# Standardized genome-wide function prediction enables comparative functional genomics: a new application area for Gene Ontologies in plants

**DOI:** 10.1093/gigascience/giac023

**Published:** 2022-04-15

**Authors:** Leila Fattel, Dennis Psaroudakis, Colleen F Yanarella, Kevin O Chiteri, Haley A Dostalik, Parnal Joshi, Dollye C Starr, Ha Vu, Kokulapalan Wimalanathan, Carolyn J Lawrence-Dill

**Affiliations:** Department of Agronomy, 2104 Agronomy Hall, 716 Farm House Lane Ames, Iowa 50011-1051, USA; Department of Plant Pathology and Microbiology, 1344 Advanced Teaching & Research Bldg, 2213 Pammel Drive, Ames, Iowa 50011, USA; Department of Agronomy, 2104 Agronomy Hall, 716 Farm House Lane Ames, Iowa 50011-1051, USA; Department of Agronomy, 2104 Agronomy Hall, 716 Farm House Lane Ames, Iowa 50011-1051, USA; Department of Agronomy, 2104 Agronomy Hall, 716 Farm House Lane Ames, Iowa 50011-1051, USA; Department of Veterinary Microbiology and Preventive Medicine, 1800 Christensen Drive, Ames, Iowa 50011-1134, USA; Department of Agronomy, 2104 Agronomy Hall, 716 Farm House Lane Ames, Iowa 50011-1051, USA; Department of Genetics, Development and Cell Biology, 1210 Molecular Biology Building, 2437 Pammel Drive, Ames, Iowa 50011-1079, USA; Department of Genetics, Development and Cell Biology, 1210 Molecular Biology Building, 2437 Pammel Drive, Ames, Iowa 50011-1079, USA; Department of Agronomy, 2104 Agronomy Hall, 716 Farm House Lane Ames, Iowa 50011-1051, USA; Department of Genetics, Development and Cell Biology, 1210 Molecular Biology Building, 2437 Pammel Drive, Ames, Iowa 50011-1079, USA

**Keywords:** gene function, ontology, plants, comparative genomics, functional genomics

## Abstract

**Background:**

Genome-wide gene function annotations are useful for hypothesis generation and for prioritizing candidate genes potentially responsible for phenotypes of interest. We functionally annotated the genes of 18 crop plant genomes across 14 species using the GOMAP pipeline.

**Results:**

By comparison to existing GO annotation datasets, GOMAP-generated datasets cover more genes, contain more GO terms, and are similar in quality (based on precision and recall metrics using existing gold standards as the basis for comparison). From there, we sought to determine whether the datasets across multiple species could be used together to carry out comparative functional genomics analyses in plants. To test the idea and as a proof of concept, we created dendrograms of functional relatedness based on terms assigned for all 18 genomes. These dendrograms were compared to well-established species-level evolutionary phylogenies to determine whether trees derived were in agreement with known evolutionary relationships, which they largely are. Where discrepancies were observed, we determined branch support based on jackknifing then removed individual annotation sets by genome to identify the annotation sets causing unexpected relationships.

**Conclusions:**

GOMAP-derived functional annotations used together across multiple species generally retain sufficient biological signal to recover known phylogenetic relationships based on genome-wide functional similarities, indicating that comparative functional genomics across species based on GO data holds promise for generating novel hypotheses about comparative gene function and traits.

## Background

Phenotypes and traits have long been the primary inspiration for biological investigation. Phenotypes are the result of a complex interplay between functions of genes and environmental cues. In an effort to organize and model gene functions, various systems of classification have been developed including systems like KEGG, which is focused on protein function including gene activities superimposed on metabolic pathways [1]. Other such systems include the various Cyc databases, MapMan, and the Gene Ontologies (GO), a vocabulary of gene functions organized as a directed acyclic graph, which makes it innately tractable for computational analysis [2–4].

GO-based gene function annotation involves the association of GO terms to individual genes. Functions may be assigned to genes on the basis of different types of evidence for the association. For example, functional predictions can be inferred from experiments (EXP), expression patterns (IEP), and more [5]. Computational pipelines are often used to generate functional predictions for newly sequenced genomes, where the genome is first sequenced and assembled, then gene structures (gene models) are predicted, then functions are associated with those gene predictions. Genome-wide gene function prediction datasets are frequently used to analyze gene expression studies, to prioritize candidate genes linked to a phenotype of interest, to design experiments aimed at characterizing functions of genes, and more [6–8]. How well a gene function prediction set models reality is influenced by how complete and correct the underlying genome assembly and gene structure annotations are, as well as by how well the software used to predict functions performs.

GOMAP (the Gene Ontology Meta Annotator for Plants) is a gene function prediction pipeline for plants that generates high-coverage and reproducible functional annotations [9]. The system uses multiple functional prediction approaches, including sequence similarity, protein domain presence, and mixed-method pipelines developed to compete in the Critical Assessment of Function Annotation (CAFA) Challenge [10], a community challenge that has advanced the performance of gene function prediction pipelines over the course of 5 organized competitions [11].

We previously annotated gene functions for the maize B73 genome and demonstrated that GOMAP’s predicted functions were closer to curated gene-term associations from the literature than those of other community functional annotation datasets, including those produced by Gramene (Ensembl pipeline) and Phytozome (Interpro2GO pipeline) [12]. Using the newly containerized GOMAP system [9], we report here the functional annotation of 18 plant genomes across the 14 crop plant species listed in Table 1 and report comparisons of performance based on comparison to gold standard gene function datasets, where possible.

**Table 1:** Functional annotation sets generated by GOMAP

Species	Germplasm/line	Assembly/annotation	Dataset DOI	Genome reference
*Arachis hypogaea*	Tifrunner	Arachis hypogaea assembly 1.0	[13]	[14]
*Brachypodium distachyon*	Bd21	Bd21.v3.1.r1	[15]	[16]
*Cannabis sativa*	Hemp	NCBI Cannabis sativa GCA_900626175.1	[17]	[18]
*Glycine max*	Williams 82	Joint Genome Institute (JGI) Wm82.a4.v1	[19]	[20]
*Gossypium raimondii*	Cotton D	Gossypium raimondii JGI v2.1	[21]	[22]
*Hordeum vulgare*	–	IBSC_PGSB_r1	[23]	[24]
*Medicago truncatula*	R108_HM340	R108: v1.0	[25]	[26]
*Medicago truncatula*	A17_HM341	Mt4.0v2	[27]	[28]
*Oryza sativa*	Japonica	IRGSP 1.0	[29]	[30]
*Phaseolus vulgaris*	G19833	DOE-JGI and USDA-NIFA annotation 2.0	[31]	[32]
*Pinus lambertiana*	Sugar Pine	TreeGenesDB sugar pine assembly v1.5	[33]	[34]
*Sorghum bicolor*	BTx623	BTx623.v3.0.1.r1	[35]	[36]
*Triticum aestivum*	Chinese Spring	IWGSC RefSeq 1.1	[37]	[38]
*Vigna unguiculata*	IT97K-499-35	JGI annotation v1.1	[39]	[40]
*Zea mays* ^a^	Mo17	Zm-Mo17-REFERENCE-CAU-1.0	[41]	[42]
*Zea mays* ^a^	PH207	Zm-PH207-REFERENCE_NS-UIUC_UMN-1.0	[43]	[44]
*Zea mays* ^a^	W22	Zm-W22-REFERENCE-NRGENE-2.0 Zm00004b.1	[45]	[46]
*Zea mays* ^a^	B73	RefGen_V4 Zm00001d.2	[47]	[48]

More information about each dataset including the source of the input to GOMAP can be found at the respective DOI. Latest overview at [49].

a Previously published in [9].

**Figure 1:**
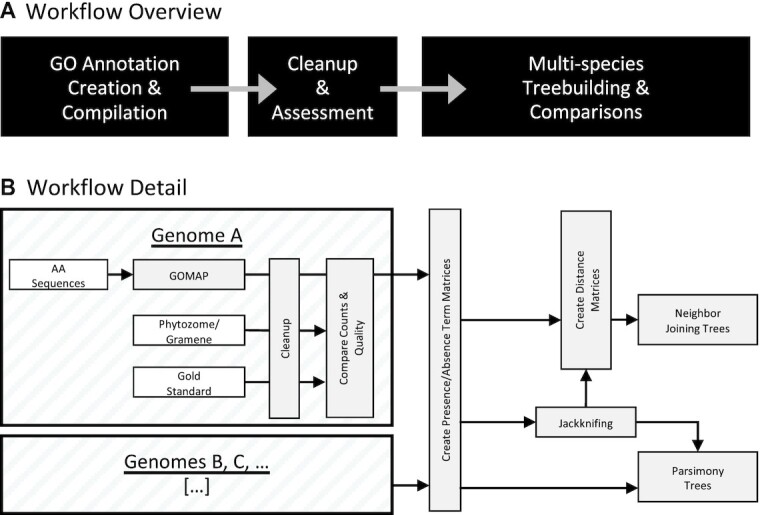
Data workflow schema. A. Workflow overview with steps represented as black boxes and the flow of information and processes indicated by arrows. B. Workflow details. The upper large hatched box shows process detail for a single genome and the lower hatched box represents additional genomes for which the details of processing are identical. White boxes represent input datasets. Arrows indicate the flow of information and processes.

**Figure 2:**
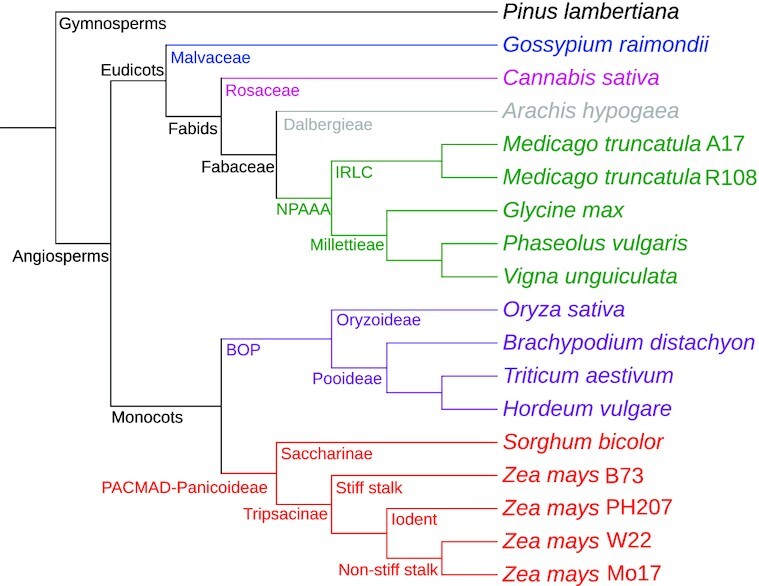
Known phylogenetic relationships among species. Cladogram is rooted by the gymnosperm *Pinus lambertiana* (black). Among angiosperms, eudicots clades include Malvaceae (blue), Rosaceae (magenta), Dalbergieae (grey), and non-protein amino acid–accumulating clade (NPAAA; green). Monocots include members of the BOP (purple) and PACMAD-Panicoideae (red) clades.

Given these multiple annotations across various plant species, we next considered whether these datasets could be used together for comparative functional genomics in plants. We describe here a simple and crude method by which we used gene function annotations to generate dendrograms of genome-level similarity in function. This idea is similar to that of Zhu et al., who determined the evolutionary relationships among microorganisms based on whole-genome functional similarity [50]. Here we expand on that approach, analyzing genome-wide GO assignments to generate parsimony and distance-based dendrograms (see Fig. 1 for process overview). We compared these with well-established species phylogenies (Fig. 2) to determine whether trees derived from gene function show any agreement with evolutionary histories, taking agreement between generated dendrograms and known evolutionary histories to be evidence that sufficient comparative biological signal exists to begin to use GO functional annotations across multiple plant genomes for comparative functional genomics investigations.

## Results of Analyses

### Overview

As shown in Fig. 1, gene function annotation sets were created and compiled for each genome. For those with existing annotation sets available on Gramene or Phytozome [51,52], the datasets were compared. From there, matrices that included genomes as rows and terms as columns were generated. These were used directly to build parsimony trees or to create distance matrices for NJ tree construction [53–55]. In subsequent analyses, jackknifing was used to remove terms (columns) or to remove genomes (rows) to map the source of signal for tree-building results [56].

### Functional annotation sets produced

Table 2 reports quantitative attributes of each of the annotation sets. In summary, GOMAP covers all annotated genomes with ≥1 annotation per gene and provides between 3.8 and 12.1 times as many annotations as Gramene or Phytozome.

**Table 2:** Quantitative metrics of the cleaned functional annotation sets

Genome	Genes	Dataset	Genes Annotated (%)				Annotations				Median annotations per gene			
			CC	MF	BP	A	CC	MF	BP	A	CC	MF	BP	A
*Arachis hypogaea*	67,124	GOMAP	85.85	84.68	100	100	150,525	132,144	493,145	775,814	2	2	6	10
*Brachypodium distachyon*	34,310	GOMAP	81.33	85.35	100	100	74,172	69,213	255,397	398,782	2	2	6	10
		Gold Standard Gramene 63 (no IEA)	21.54	19.53	18.20	26.66	10,985	10,436	11,120	32,673	1	1	1	3
		Gramene63 (IEA only)	33.12	49.29	38.29	63.60	21,658	36,372	23,899	82,026	1	1	1	3
		Phytozome12	10.25	37.21	26.86	43.11	4,186	18,597	11,070	34,060	0	1	1	2
*Cannabis sativa*	33,677	GOMAP	94.22	95.48	100	100	85,755	73,614	262,741	422,110	2	2	6	11
*Glycine max*	52,872	GOMAP	86.95	88.92	100	100	126,470	113,068	416,989	656,527	2	2	6	11
*Gossypium raimondii*	37,505	GOMAP	93.00	92.37	100	100	95,419	84,910	307,470	487,799	2	2	6	11
*Hordeum vulgare*	39,734	GOMAP	88.57	91.76	100	100	86,489	79,727	272,420	438,636	2	2	5	10
		Gold Standard Gramene 63 (no IEA)	28.23	26.30	23.43	35.64	15,734	15,391	15,267	46,414	1	1	1	3
		Gramene63 (IEA only)	36.19	50.90	41.71	65.03	29,826	44,789	29,425	104,178	1	1	1	3
*Medicago truncatula* A17	50,444	GOMAP	83.79	86.69	100	100	104,902	99,155	363,608	567,665	2	2	6	10
		Gold Standard Gramene 63 (no IEA)	25.45	23.26	21.51	32.12	17,938	18,416	18,461	54,827	1	1	1	3
		Gramene63 (IEA only)	34.25	50.84	40.26	66.14	32,753	63,470	40,441	137,001	1	1	1	3
		Phytozome12	8.87	36.05	25.83	41.07	5,315	25,950	15,576	47,098	0	1	1	2
*Medicago truncatula* R108	55,706	GOMAP	72.10	90.14	100	100	108,388	107,499	381,831	597,718	1	2	5	9
*Oryza sativa*	35,825	GOMAP	79.78	83.31	100	100	71,306	64,150	248,304	383,760	2	2	6	9
		Gold Standard Gramene 63 (no IEA)	29.95	27.29	25.33	37.57	15,492	15,176	16,536	47,339	1	1	1	3
		Gramene63 (IEA only)	32.21	45.83	36.75	60.13	21,935	37,425	24,255	83,645	1	1	1	3
		Phytozome12	10.31	40.10	29.18	46.09	4,361	20,842	12,451	37,884	0	1	1	2
*Phaseolus vulgaris*	27,433	GOMAP	94.48	93.06	100	100	70,987	64,022	229,230	364,239	2	2	6	11
*Pinus lambertiana*	31,007	GOMAP	92.67	95.91	100	100	71,247	68,315	212,248	351,810	2	2	5	10
*Sorghum bicolor*	34,129	GOMAP	82.44	85.98	100	100	75,145	69,659	259,004	403,808	2	2	6	10
		Gold Standard Gramene 63 (no IEA)	34.48	32.91	30.90	42.84	16,837	17,614	17,850	52,593	1	1	1	3
		Gramene63 (IEA only)	35.91	52.11	42.36	67.41	23,608	39,418	27,074	90,313	1	1	1	3
		Phytozome12	10.54	39.19	27.90	45.10	4,246	19,724	11,432	35,599	0	1	1	2
*Triticum aestivum*	107,891	GOMAP	88.53	90.98	100	100	259,318	217,467	785,051	1,261,836	2	2	6	10
		Gold Standard Gramene 63 (no IEA)	2.98	2.78	2.56	3.82	4,727	4,512	4,793	14,035	1	1	1	3
		Gramene63 (IEA only)	29.12	58.62	38.72	70.41	47,595	111,889	62,977	222,721	0	1	1	2
*Vigna unguiculata*	29,773	GOMAP	91.21	91.08	100	100	74,791	67,734	242,847	385,372	2	2	6	11
		Phytozome12	13.91	45.68	34.14	53.06	5,107	19,962	12,209	37,534	0	1	1	2
*Zea mays* B73.v4	39,324	GOMAP	93.16	94.92	100	100	87,648	81,665	278,305	447,618	2	2	6	10
		Gold Standard Gramene 63 (no IEA)	37.92	34.78	32.67	46.85	22,531	21,292	23,153	67,285	1	1	1	3
		Gramene63 (IEA only)	39.16	58.16	48.21	73.87	30,189	53,748	35,276	119,273	1	1	1	3
*Zea mays* Mo17	38,620	GOMAP	86.98	90.87	100	100	86,074	78,650	277,395	442,119	2	2	6	10
		Gold Standard Gramene 63 (no IEA)	27.56	25.20	23.73	33.98	16,128	15,384	16,489	48,220	1	1	1	3
*Zea mays* PH207	40,557	GOMAP	86.55	90.61	100	100	88,962	84,910	288,208	462,080	2	2	6	10
		Gold Standard Gramene 63 (no IEA)	28.18	25.82	24.26	34.66	17,370	16,580	17,791	51,984	1	1	1	3
*Zea mays* W22	40,690	GOMAP	90.77	92.58	100	100	93,622	84,450	289,364	467,436	2	2	6	10
		Gold Standard Gramene 63 (no IEA)	25.40	23.15	21.80	31.29	15,518	14,818	15,850	46,402	1	1	1	3

CC, MF, BP, and A refer to the aspects of the Gene Ontology: cellular component, molecular function, biological process, and any/all. GOMAP covers all genomes with ≥1 annotation per gene and provides substantially more annotations than Gramene63 or Phytozome, especially in the BP aspect. The total number of annotations per dataset is visualized in Supplementary Fig. S1.

^a^ How many genes in the genome have ≥1 GO term from the CC, MF, BP aspect annotated to them? A = How many ≥1 from any aspect? (A = CC∪MF∪BP).

^b^ How many annotations in the CC, MF, and BP aspect does this dataset contain? A = How many in total? A = CC + MF + BP.

^c^ Take a typical gene that is present in the annotation set. How many annotations does it have in each aspect? A = How many in total? Note that A ≠ CC + MF + BP.

Quality evaluation of gene function predictions is not trivial and is approached by different research groups in different ways. Most often datasets are assessed by comparing the set of predicted functions for a given gene to a gold standard consisting of annotations that are assumed to be correct. This assumption of correctness can be based on any number of criteria. Here we used as our gold standard dataset all annotations present in Gramene63 that had a non-IEA (non-inferred by electronic annotation) evidence code; i.e., we used only annotations that had some manual curation. This enabled us to assess 10 of the genomes described in Table 2. It is perhaps noteworthy that the IEA and non-IEA annotation sets from Gramene63 frequently contain overlaps, indicating that some of the predicted annotations were manually confirmed afterwards by a curator and that in such cases, a new annotation was asserted with the new evidence code rather than simply upgrading the evidence code from IEA to some other code, thus preserving the IEA annotations in Gramene63 that are produced by the Ensembl analysis pipeline [57], a requirement for comparing GOMAP-produced IEA datasets to the IEA datasets produced by the Ensembl pipeline.

A general limitation of using gold standards for quality evaluation is that they can never be assumed to be complete, and therefore false-positive results in the prediction cannot be distinguished from false-negative results in the gold standard. In other words, is gene X, function Y truly a wrong prediction or has it simply not yet been discovered experimentally? This problem is laid out in more detail in [58]. As a consequence, the quality of larger prediction sets will be systematically underestimated compared to smaller ones, and this effect is exacerbated the more incomplete the gold standard is.

There are many different metrics that have been used to evaluate the quality of predicted functional annotations. For the maize B73 GOMAP annotation assessment in [12], we had used a modified version of the hierarchical evaluation metrics originally introduced in [59] because they were simple, clear, and part of an earlier attempt at unifying and standardizing GO annotation comparisons [60]. In the meantime, Plyusnin et al. published an approach for evaluating different metrics showing variation among the robustness of different approaches to quality assessment [61]. On the basis of their recommendations, we use here the SimGIC2 and term-centric area under precision-recall curve (TC-AUCPCR) metrics. We also evaluated with the *F*_max_ metric, simply because it is widely used (e.g., by [10]), even though according to Plyusnin et al., it is actually a flawed metric [61]. Results of the quality assessments for the 10 genomes where a gold standard was available are reported in Table 3 and Supplementary Fig. S2. While evaluation values differ between metrics and the scores are not directly comparable, a few consistent patterns emerge: GOMAP annotations are almost always better than Gramene and Phytozome annotations in the cellular component and molecular function aspect, with the only 3 exceptions being the molecular function aspect for *T. aestivum* using the TC-AUCPCR and the *F*_max_ metric and the cellular component aspect for *M. truncatula* A17 using the *F*_max_ metric. Conversely, GOMAP predictions achieve consistently lower quality scores in the biological process aspect with the exception of *B. dystachion, O. sativa*, and *S. bicolor* with the TC-AUCPR metric. Generally, annotations that are better in 1 aspect are also better in the other 2 aspects, but the ranking of annotations does not necessarily hold across metrics. The Phytozome annotation for *O. sativa* is an outlier in terms of its comparative quality, potentially because it is based on a modified structural annotation that differs substantially from the gold standard and the other annotations under comparison.

**Table 3:** Qualitative metrics of functional annotation sets predicted by GOMAP, Gramene, and Phytozome

Genome	Dataset	SimGIC2			TC-AUCPCR			*F*_max_		
		CC	MF	BP	CC	MF	BP	CC	MF	BP
*Brachypodium distachyon*	GOMAP	0.404149	0.464127	0.223830	0.233442	0.230701	0.118526	0.741361	0.740897	0.526881
	Gramene63 (IEA only)	0.317801	0.420859	0.349406	0.129163	0.192507	0.111361	0.691016	0.738542	0.650325
	Phytozome12	0.370264	0.370521	0.352206	0.112582	0.136832	0.085628	0.717759	0.697076	0.660603
*Hordeum vulgare*	GOMAP	0.400087	0.470012	0.238177	0.237231	0.261399	0.130784	0.745272	0.750213	0.560096
	Gramene63 (IEA only)	0.306119	0.426601	0.381010	0.157352	0.228797	0.136002	0.680996	0.742638	0.665696
*Medicago truncatula* A17	GOMAP	0.371795	0.451258	0.213407	0.272809	0.282650	0.139032	0.730838	0.726991	0.531406
	Gramene63 (IEA only)	0.329600	0.437274	0.343561	0.176497	0.265887	0.133503	0.701093	0.749900	0.654297
	Phytozome12	0.358311	0.367257	0.363013	0.144247	0.170863	0.110386	0.717307	0.698429	0.661233
*Oryza sativa*	GOMAP	0.408945	0.482650	0.248207	0.298502	0.303384	0.159724	0.751121	0.757181	0.559221
	Gramene63 (IEA only)	0.328761	0.423191	0.341193	0.167619	0.265410	0.135451	0.711309	0.738732	0.643827
	Phytozome12	0.049975	0.041007	0.044279	0.000003	0.000003	0.000002	0.470134	0.266628	0.239256
*Sorghum bicolor*	GOMAP	0.404852	0.466708	0.224011	0.316873	0.337380	0.169883	0.746540	0.742001	0.534258
	Gramene63 (IEA only)	0.323037	0.400241	0.353135	0.177038	0.260198	0.154157	0.711107	0.712170	0.653591
	Phytozome12	0.356091	0.348264	0.340124	0.151947	0.177579	0.110483	0.715714	0.675147	0.641535
*Triticum aestivum*	GOMAP	0.410582	0.489881	0.229271	0.050762	0.030610	0.019360	0.736476	0.762420	0.533897
	Gramene63 (IEA only)	0.362452	0.476685	0.395112	0.040992	0.043701	0.027872	0.737769	0.762059	0.670953
*Zea mays* B73.v4	GOMAP	0.417455	0.467339	0.245373	0.302761	0.290371	0.153011	0.759504	0.746870	0.564707
	Gramene63 (IEA only)	0.303231	0.416301	0.346308	0.175735	0.250075	0.138275	0.662987	0.732860	0.647725
*Zea mays* Mo17	GOMAP	0.399521	0.464265	0.225632	0.236209	0.239598	0.125599	0.744360	0.743026	0.537489
*Zea mays* PH207	GOMAP	0.394481	0.436266	0.224226	0.221709	0.221266	0.117086	0.743111	0.718933	0.533092
*Zea mays* W22	GOMAP	0.397602	0.463499	0.223511	0.210198	0.217609	0.113262	0.743783	0.742341	0.535572

This table is visualized in Supplementary Fig. S2.

### Phylogenetic tree analyses

**Figure 3:**
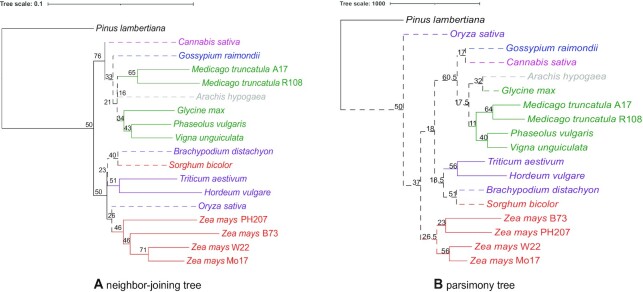
Neighbor-joining and parsimony trees. Phylograms are colored and rooted as described in Fig. 2. For both neighbor-joining (A) and parsimony (B), node values represent the jackknifing support values derived by removing 40% of GO terms in the dataset. Dashed lines mark deviations from known phylogenetic relationships. Tree scales are shown above each, with NJ showing distances and parsimony showing changes in character state.

With the comparative quality of gene function predictions in hand, we approached the question of whether the datasets could be used together for comparative functional analysis across all genomes. As a simple first step, we began to work toward understanding the degree to which trees built on the basis of gene functions agree with known, well-documented evolutionary relatedness. We constructed neighbor-joining (NJ) and parsimony trees of the 18 plant genomes and visulized them using iTOL [62]. The 2 tree topologies, rooted at *P. lambertiana*, were compared to one another and to the topology of the expected tree (Fig. 2). For both the NJ (Fig. 3A) and parsimony trees (Fig. 3B), 1 common difference is noted: *S. bicolor* is not at the base of the *Z. mays* clade as expected and is clustered with *B. distachyon* instead. Notable differences between the NJ and parsimony tree are the following: *C. sativa* appears at the base of the eudicots instead of *G. raimondii* in the NJ tree, while *G. raimondii* is grouped with *C. sativa* and *A. hypogaea* is grouped with *G. max* in the parsimony tree. Second, *O. sativa* was expected to be at the base of the Bambusoideae, Oryzoideae, and Pooideae (BOP) clade but appears at the base of *Z. mays* in the NJ tree, and at the base of all angiosperms in the parsimony tree. Differences among relationships within the *Z. mays* clade constaining B73, PH207, W22, and Mo17 were disregarded given the high degree of similarity across annotation sets and the fact that these relationships are not clear given the complex nature of within-species relationships.

Owing to differences between the function-based dendrograms and the expected tree, jackknifing analysis was carried out by removing terms (columns in underlying datasets) to determine the degree to which the underlying datasets support specific groupings based on functional term assignments. This analysis was carried out for both NJ and parsimony trees. First, trees were generated by omitting 5%–95% of the dataset in increments of 5 to determine the threshold at which the tree topologies deviated from those generated using the full dataset. That threshold was reached at 45% for both NJ and parsimony; therefore, we used trees generated with 40% of the data removed for reporting branch support values for the topology (Fig. 3). Comparing the 2 trees, the parsimony topology was not as solid as that of the NJ at jackknife values ≤40%. On the basis of this robustness for NJ tree-building in general, we carried out all subsequent analyses using NJ tree-building methods.

We considered investigating the effect of using 1 GO aspect to generate our NJ tree. In other words, we generated the NJ trees using cellular component GO terms, molecular function GO terms, and biological process GO terms separately (Supplementary Fig. S3). Of the 14,303 total GO terms, 1,524 are cellular component terms, 3,926 are molecular function terms, and 8,853 are biological process terms. Of the 3 single-aspect phylogenetic trees, the one built using molecular function terms is the closest to our NJ tree obtained using all GO terms in our datasets (Fig. 3A). The only difference is that *A. hypogaea* and *G. max* are clustered in the molecular function tree, while they are not in our NJ tree Fig. 3A. In the cellular component tree, *G. raimondii* and *C. sativa* are clustered together when they are not in the NJ tree with all GO aspects (Fig. 3A). Also, *O. sativa* is at the base of the monocots just like in the expected tree, but not in the NJ tree Fig. 3A. In the biological process tree, *O. sativa* is at the base of the angiosperms and there is no clear separation between monocots and dicots. In all 3 single-aspect phylogenetic trees and our all-aspect NJ tree, *A. hypogaea* is never placed at the base of the NPAAA clade. Also, *B. distachyon* and *S. bicolor* are always clustered together. Overall, the topologies constructed using 1 GO term aspect at a time are close to that of our NJ tree, such that not one GO term aspect alone restored the topology of the expected tree.

**Figure 4:**
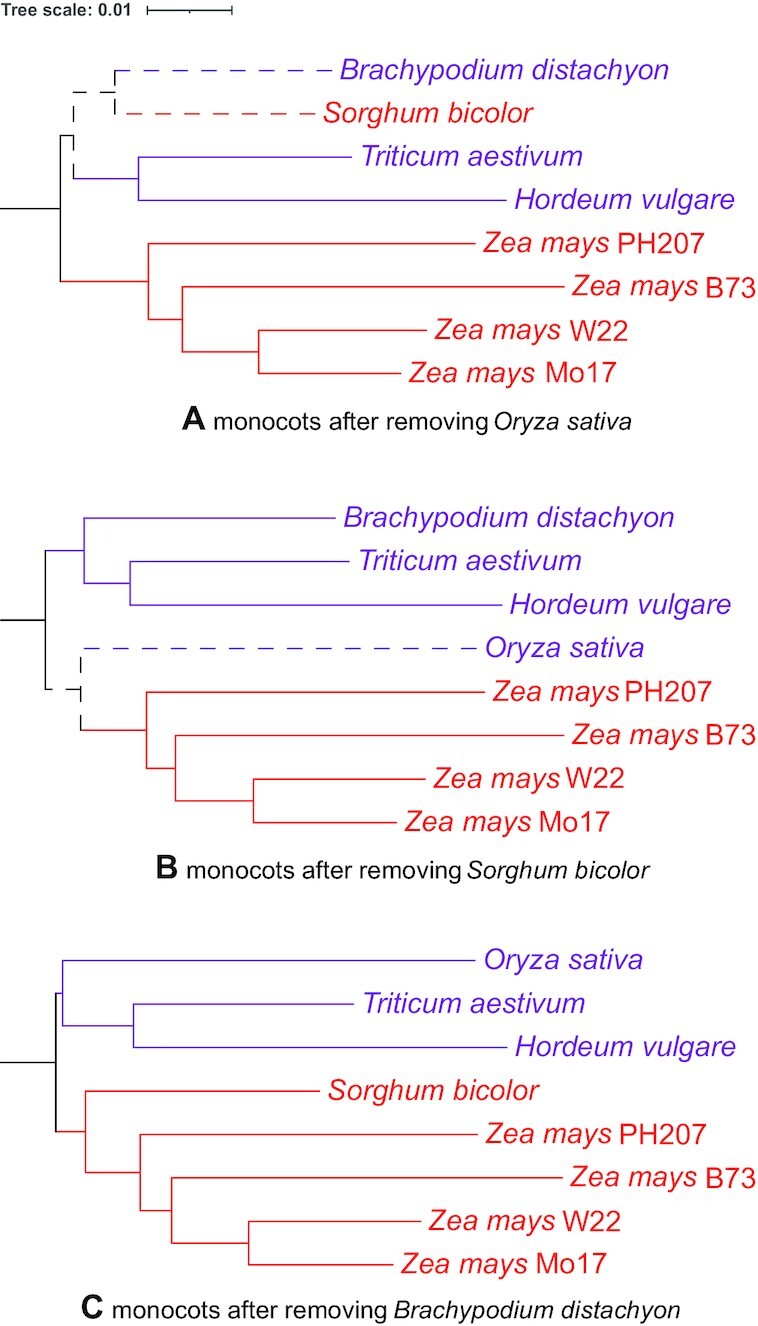
Restoring monocot relationships. Phylograms are colored and rooted as described in Fig. 2. Dashed lines mark deviations from known phylogenetic relationships. Monocot topology changes with removal of a single species: (A) *O. sativa*, (B) *S. bicolor*, and (C) *B. distachyon*. Tree scale is shown above.

To map the source of discrepancies to specific gene annotation sets, we generated various NJ trees excluding 1 genome each time, an additional tree with both *Medicago* genomes excluded simultaneously, and another with all *Z. mays* genomes excluded simultaneously. To exemplify this, see the monocot clade in Fig. 2 and the lower (monocot) clade in Fig. 3A. When the NJ tree was generated, 2 species are misplaced: *S. bicolor* and *O. sativa*. As shown in Fig. 4a, removal of *O. sativa* corrects 1 error (itself) but does not correct the errant grouping of *S. bicolor* with *B. distachyon*. In Fig. 4b, it is shown that the removal of *S. bicolor* corrects the errant grouping of itself and *B. distachyon*, but *O. sativa* placement remains incorrect. However, as shown in Fig. 4c, the removal of *B. distachyon* generates a tree where all relationships are in agreement with known species-level relationships. (Note well: all individual annotation sets were progressively removed, not just the 3 shown in the example.)

**Figure 5:**
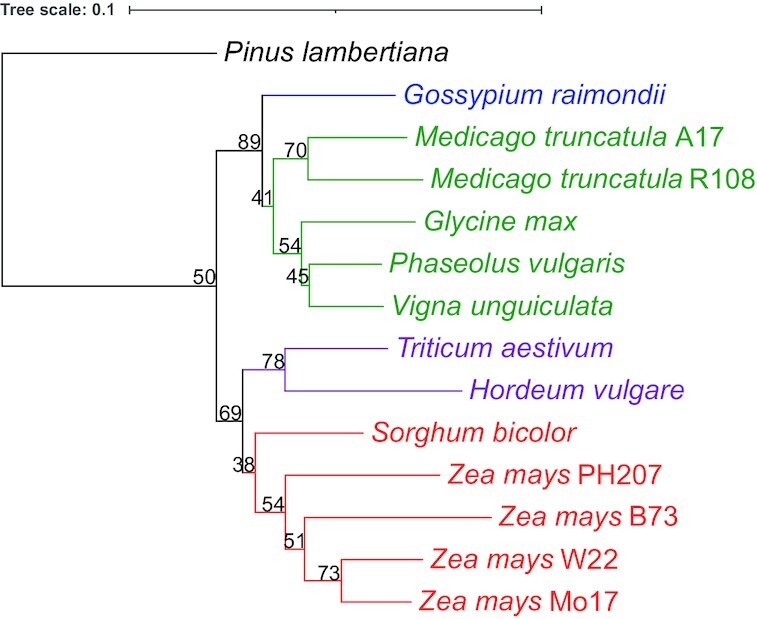
Restoring known phylogentic relationships to the NJ tree via removal of a minimal number of species. Phylograms are colored and rooted as described in Fig. 2. Node values represent the jackknifing support values derived by removing 40% of GO terms in the dataset. Four genomes have been removed: *C. sativa, O. sativa, B. distachyon*, and *A. hypogaea*. Tree scale is shown above.

With this observation in hand, we sought to determine the minimum number of genomes that could be removed to create a tree that matched the expected tree topology. All possible combinations of removing 0–5 genomes to restore the topology were tested, and 10 combinations of minimum amount of genomes to be removed were obtained. The removal of 4 genomes was required to generate function-based trees consistent with known phylogenetic relationships. Of the 10, we selected the 1 that had the genomes that were most frequently part of a solution (*O. sativa*, 8; *B. distachyon*, 7; *C. sativa*, 6; *A. hypogaea*, 5; *S. bicolor*, 4; *G. raimondii*, 4; *G. max*, 4; *T. aestivum*) to show in this article (the other combinations can be found in our publicly available dataset). To elaborate, the genomes removed here are *O. sativa, B. distachyon, C. sativa*, and *A. hypogaea* (Fig. 5). Jackknifing analysis was also carried out for this dataset with support shown. Branch support is generally higher than that for the full dataset (i.e., branch support is higher in Fig. 5 than in Fig. 3A), and removing genomes that are causing variations seems to stabilize the tree.

### Potential causes of unexpected groupings

**Figure 6:**
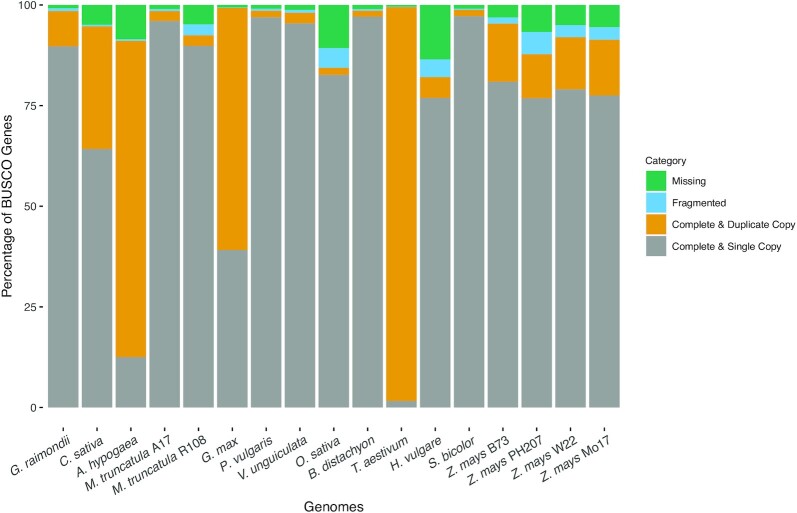
Assembly BUSCO plot generated using GenomeQC. Genomes analyzed are shown across the X-axis and are ordered to match the occurrence of species shown in Fig. 2. Percentages of BUSCO genes across 4 gene categories are stacked, with each adding up to 100% (Y-axis).

**Figure 7:**
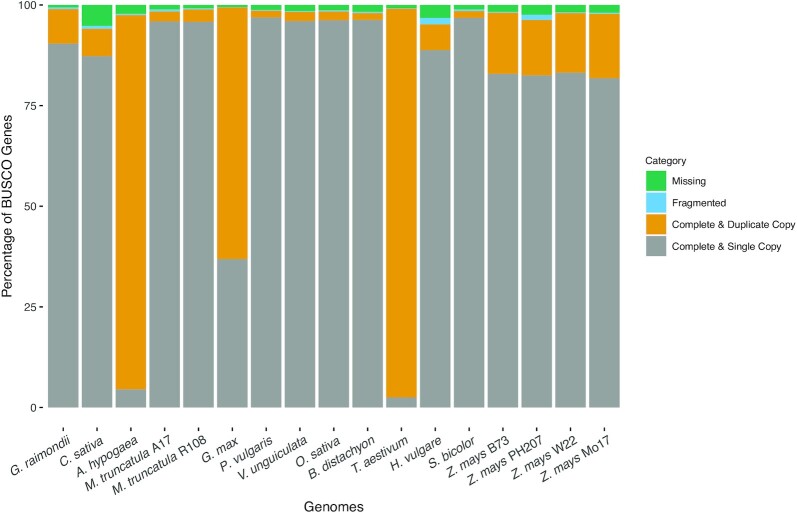
Annotation BUSCO plot generated using GenomeQC. Genomes analyzed are shown across the X-axis and are ordered to match the occurrence of species shown in Fig. 2. Percentages of BUSCO genes across 4 gene categories are stacked, with each adding up to 100% (Y-axis).

As a first step toward explaining discrepancies between known evolutionary relationships and those resulting from comparative analysis of genome-wide gene function predictions, we assessed the quality of each genome assembly and structural annotation set using GenomeQC [63]. Tables 4 and 5 and Figs. 6 and 7 represent the resulting assembly quality, structural annotation measures of quality, and proportion of single-copy BUSCOs [64] that were generated. Although these analyses make evident that the species annotated are comparatively different in both natural genome characteristics and in assembly and annotation quality aspects, it is not the case that the 4 species responsible for deviations between the functional annotation dendrograms and known phylogenetic relationships (i.e., *C. sativa, A. hypogaea, O. sativa*, and *B. distachyon*) create these discrepancies owing to issues of genome assembly and/or annotation quality. One potential for some explanation is in relation to *C. sativa*, which is the only genome that has an assembly length larger than the expected (see Table 4), and a comparatively large proportion of missing BUSCOs in the assembly (see Fig. 6). Similarly, for *A. hypogaea* and *O. sativa*, there is a large proportion of missing BUSCOs in the annotations (see Fig. 7).

**Table 4:** Assembly statistics^a^

Species	C-value		Estimated genome size (Mb)	Scaffolds	Total scaffold length (Mb)	Total scaffold length/estimated genome size (%)	Length scaffold sequences ≥25k nt (Mb)	Scaffold sequences ≥25k nt/estimated genome size (%)	%N
	(pg)	Source							
*A. hypogaea*	2.87	[65]	2,806	384	2,560	91.12	2,550	91.01	0.15
*B. distachyon*	0.32	[66]	313	11	272	87.00	272	87.00	0.19
*C. sativa*	0.84	[67]	821	221	876	106.72	875	106.62	15.93
*G. max*	1.13	[68]	1,105	282	978	88.62	977	88.48	2.65
*G. raimondii*	0.9	[69]	880	1033	761	86.52	755	85.77	1.75
*H. vulgare*	7.33	[70,71]	7,167	8	4,830	67.45	4,830	67.45	5.44
*M. truncatula* A17	0.47	[72]	460	2,186	413	89.93	403	87.77	5.53
*M. truncatula* R108	0.47	[72]	460	909	402	87.60	395	86.09	0.68
*O. sativa*	0.5	[70]	489	63	375	76.85	375	76.74	0.03
*P. vulgaris*	0.6	[73]	587	478	537	91.68	534	91.12	1.05
*S. bicolor*	1.2	[74]	1,173	870	709	60.43	705	60.08	4.72
*T. aestivum*	17.3	[70]	16,916	22	14,500	86.00	14,500	86.00	1.90
*V. unguiculata*	0.6	[75]	587	686	519	88.64	518	88.38	0.00
*Z. mays* B73	2.7	[70]	2,640	266	2,130	80.85	2,130	80.85	1.44
*Z. mays* Mo17	2.7	[70]	2,640	2,208	2,180	82.68	2,170	82.06	1.61
*Z. mays* PH207	2.7	[70]	2,640	43,291	2,160	81.67	2,090	79.25	19.61
*Z. mays* W22	2.7	[70]	2,640	11	213E0	80.83	2,130	80.83	1.90

a The extended assembly statistics are found in Supplementary Table S1.

**Table 5:** Structural annotation table directly from GenomeQC

Species	Gene models	Gene length range (bp)	Mean gene length (bp)	Gene models <200 bp	Transcripts	Mean transcripts per gene model	Exons	Mean exons per transcript	Mean exon length (bp)
*A. hypogaea*	67,128	163–342,359	3,972.3	48	84,714	1.3	423,763	4.8	296.4
*B. distachyon*	36,647	90–47,411	3,012.5	175	47,917	1.3	263,865	5.5	314.1
*C. sativa*	29,815	21–976,063	3,450.0	2,187	34,876	1.2	234,157	6.6	291.9
*G. max*	52,872	90–94,733	4,017.0	1,363	86,256	1.6			
*G. raimondii*	37,505	90–51,175	3,243.5	71	77,267	2.1	527,563	6.7	271.3
*H. vulgare*					248,270		1,715,898		278.9
*M. truncatula* A17	51,541	60–102,191	2,566.9	1,985	74,213	1.4	318,421	4.4	313.2
*M. truncatula* R108	55,706	72–62,996	2,232.4	2,404	61,019	1.1	220,904	3.6	270.6
*O. sativa*	35,825	72–304,271	3,098.7	57	42,378	1.2	192,499	4.5	350.3
*P. vulgaris*	27,433	93–90,772	3,943.8	108	36,995	1.3			
*S. bicolor*	34,129	96–88,337	3,713.2	56	47,121	1.4	266,301	5.6	350.9
*T. aestivum*	107,891	54–124,945	3,488.9	686	133,744	1.2	749,233	5.8	303.3
*V. unguiculata*	29,773	90–81,066	3,880.9	408	42,287	1.4			
*Z. mays* B73	39,324	111–128,402	4,117.8	38	151,959	3.9	1,488,381	9.7	281.5
*Z. mays* Mo17	38,620	21–146,217	4,076.8	1,225	46,530	1.2	272,323	5.9	297.1
*Z. mays* PH207	40,557	111–714,207	5,993.3	53	40,557	1.0	208,149	5.1	252.1
*Z. mays* W22	40,691	87–154,495	4,330.4	64	51,717	1.3	313,830	6.1	292.4

Missing data in *H. vulgare* is due to the absence of gene model information in the GFF file.

Missing data in *G. max, P. vulgaris*, and *V. unguiculata* is due to the absence of exon information in their GFF files.

## Discussion

In this study, we used the GOMAP pipeline to produce whole-genome GO annotations for 18 genome assembly and annotation sets from 14 plant species [9]. Assessments of the number of terms predicted, as well as the quality of predictions, indicate that GOMAP functional prediction datasets cover more genes, contain more predictions per gene, and are of similar quality to prediction datasets produced by other systems, thus supporting the notion that these high-coverage datasets are a useful addition for researchers who are interested in genome-level analyses, including efforts aimed at prioritizing candidate genes for downstream analyses. Given that we can now produce high-quality, whole-genome functional annotations for plants in a straightforward way, we intend to produce more of these over time (indeed we recently annotated *Vitis vinifera* [76], *Brassica rapa* [77], *Musa acuminata*[78], *Theobroma cacao* [79], *Coffea canephora* [80], *Vaccinium corymbosum* [81], *Solanum lycopersicum* [82], and *Solanum pennellii* [83]).

With 18 genome functional annotations in hand, we sought to determine whether and how researchers could use multispecies GO annotation datasets to perform comparative functional genomics analyses. As a proof of concept, we adapted phylogenetic tree-building methods to use the gene function terms assigned to genes represented by the genomes to build dendrograms of functional relatedness and hypothesized that if the functions were comparable across species, the resulting trees would closely match evolutionary relationships. To our delight and surprise, the NJ and parsimony trees (Fig. 3) did resemble known phylogenies, but were not exact matches to broadly accepted phylogenetic relationships.

After removing the minimum number of genomes that resulted in restoration of the expected evolutionary relationships, we found that the individual species that may be responsible for the discrepancies observed in Fig. 3 were *C. sativa, A. hypogaea, O. sativa*, and *B. distachyon*. We hypothesize that the following could account for such errant relationships:

1. Quality of sequencing and coverage assembly: genomes of similarly high sequence coverage that have excellent gene calling would be anticipated to create the best source for functional annotation. Genomes of comparatively lower, or different, character would be anticipated to mislead tree-building and other comparative genomics approaches.
2. Shared selected or natural traits: species that have been selected for, e.g., oilseeds may share genes involved in synthesis of various oils. Other shared traits would be anticipated to cause similarities for species with those shared traits.
3. Lack of good representation of diverse plant biology aspects in the GO graph: most plant-specific GO terms were derived from functional analysis of 1 model species, *Arabidopsis thaliana*. This single source for presence of plant-specific functions limits the graph from containing unique functional aspects of plant biology represented in other species’ genomes. In addition, this limitation could lead to the assignment of unknown or errant functions based on a lack of closely related and/or plant-specific terms.
4. Use of a simple method of tree-building based on the presence or absence of gene function terms: the method that we devised and describe here is not sophisticated enough to make full use of information in the GO graphs such that we recover the full detail of the species’ evolutionary histories from the simple method.

To consider the first of these, we looked at genome assembly and annotation quality metrics (see Tables 4 and 5 and Figs. 6 and 7). For *B. distachyon* we could find no compelling evidence that assembly structural or functional annotation quality differed significantly from all others, except in the case of *C. sativa*, where we noted that the assembly length exceeded the predicted genome size based on C-values for genome sizes reported previously [67]. In this case, the fact that the *C. sativa* line sequenced is not inbred [84] may be responsible for the inflated assembly size relative to what is expected. This means that in the assembly, there are likely regions where alleles between chromosomes do not align, which would inflate the overall length of the assembly. In addition, the assembly misses a large proportion of BUSCO genes compared to most other genomes included in this analysis. Indeed, the comparatively low-quality assembly for *Cannabis* genome has been noted by others [85], and our preliminary investigations indicate that the assembly length is in fact longer than expected.

In an attempt to better understand conflicting phylogenetic signals that could be caused by the second potential cause, i.e., shared selected or natural traits, we mapped all GO terms that exist in our binary matrix and traced character history (presence/absence) on the nodes and leaves of our expected evolutionary tree using the software Mesquite version 3.61 [86]. These data summarize the gain or loss of each GO term across the species described in this article and can be found in our GitHub repository. We carried out a number of simple experiments to reveal which terms could be causal for errant relationships (e.g., dropping all unique terms from the *B. distachyon* dataset, reconstructing the term states at nodes that should be where *B. distachyon* should occur) and could not identify any biologically compelling patterns. (Because these analyses were not fruitful, they were not specifically included in our Methods section, although we do include the input datasets here in section “Availability of Source Code and Supporting Data,” for others to consider and peruse independently.)

A likely limitation in this analysis is the effect of the third potential cause of discrepancies between our generated phylogenetic trees and the expected topology: a deficiency of terms that describe diverse gene functions across the diversity of plant species. Because most GO terms specific to plant biology are likely derived from *Arabidopsis*, a model dicot species, gene functions unique to other species are expected to be missing from the GO graphs [87]. This source of error will only be corrected over time as gene functions unique to diverse plant species are populated into the GO graph.

We consider the most likely explanation for observed discrepancies between the known evolutionary phylogenies and dendrograms created on the basis of GO terms describing gene function to be a result of the fourth explanation: the simplicity of the tree-building models and methods we used for these analyses. Because the tree-building and analytics described in this article were based on the presence/absence of GO terms, novel terms are highly influential on the outcomes of the analysis and the number of times a term is used does not influence the outcome at all. In contrast, plant genomes are notable for having many duplicated genes as a result of whole-genome and segmental duplications over evolutionary history, so these duplications are in fact a feature of and marker for what happened to that genome over time. Therefore, using presence/absence of GO terms where the counts of term occurrences are not weighted may be too simple to get at the genuine biological complexities represented in any given plant genome. Our simplistic demonstration of the utility of GO datasets for comparative functional genomics shows that more sophisticated methods are promising for comparative functional genomics analyses.

It should be noted that comparative analyses using gene functions are not completely absent from the literature—although they are absent for large genome comparisons in plants. An example of such existing comparative use of GO is one where a given tree topology was used to look for gains and losses of functions mapped to independently derived trees, which was reported by Schwacke et al. [88]. They report, as an example of their method, an analysis of gene loss in *Cuscuta*, a parasitic plant, based on analysis using the Mapman ontology. In their work, they showed considerable loss of genes, which is a hallmark of the parasitic lifestyle. Our efforts differ in that we used the functions directly to infer tree structures as a demonstration that sufficient biological signal is present in GO-based datasets of genome-wide function prediction to reproduce known biological relationships. The method that we used was quick and dirty, and we anticipate that refinements in approach that consider multiple copies of genes, as well as using different types of graph and network representations beyond tree structures, are logical next steps for refining the use of GO terms for comparative functional genomics analyses in plants. With that in mind, we look forward not only to developing systems to support GO-based comparative functional genomics tools but also to seeing the tools that other research groups will develop to approach the use of these datasets to formulate novel comparative functional genomics hypotheses.

## Methods

### Acquiring input datasets

For each of the 18 genomes listed in Table 1, information on how to access input annotation products is listed by DOI. For each, 1 representative translated peptide sequence per protein-coding gene was selected and used as the input for GOMAP, a gene function prediction tool for plants that is actively maintained, updated, and versioned. Details of how GOMAP annotations are derived including the specificity of component datasets and which terms are retained are described elsewhere [9, 12]. In brief, GOMAP annotations are a combination of the annotations from multiple sources. GOMAP combines the annotations from all the sources and removes the less specific annotations that could be inferred from the more specific annotations, keeping only the most specific terms for each gene that cannot be inferred from other terms (i.e., only leaf terms). Unless the authors of the genome provided a set of representative sequences designated as canonical, we chose the longest translated peptide sequence as the representative for each gene model. In general, non-IUPAC characters and trailing asterisks were removed from the sequences, and headers were simplified to contain only non-special characters. The corresponding script for each dataset can be found at the respective DOI. On the basis of this input, GOMAP yielded a functional annotation set spanning all protein-coding genes in the genome. Using the Gene Ontology version releases/2020-10-09, this functional annotation set was cleaned up by removing duplicates, annotations with qualifiers (NOT, contributes_to, colocalizes_with; column 4 in the GAF 2.1 format), and obsolete GO terms. Any terms containing alternative identifiers were merged to their respective main identifier, uncovering a few additional duplicates, which were also removed. Supplementary Table S2 shows the number of annotations removed from each dataset produced.

To compare the quality of GOMAP predictions to currently available functional predictions from Gramene and Phytozome, we downloaded IEA annotations from Gramene (version 63) [51, 89] and Phytozome (version 12) [52, 90] for each species with functional annotations of the same genome version. These datasets were cleaned as above. Similarly cleaned non-IEA annotations from Gramene63 served as the gold standard wherever they were available. More detailed information on how these datasets were accessed can be found at [91].

### Quantitative and qualitative evaluation

The number of annotations in each clean dataset was determined and related to the number of protein-coding genes (based on transcripts in the input FASTA file). This was done separately for each GO aspect as well as in total.

The ADS software version published in [61] is available from [92]. We used version b6309cb (also included in our code as a submodule) to calculate SimGIC2, TC-AUCPCR, and *F*_max_ quality scores. To provide the information content required for the SimGIC2 metric, the Arabidopsis GOA from [93] was used in version 2021-02-16.

### Cladogram construction

For clustering, we first collected all GO terms annotated to any gene in each genome into a list and removed the duplicates, yielding a 1D set of GO terms for each genome (*T*). Next, we added all parental terms for each term in this set (connected via *is_a* in the ontology), their respective parental terms and higher, recursively continuing up to the very root of the ontology. Then we once again removed the duplicates, yielding a set *S* containing the original terms from set *T* as well as all terms proximal to them in the GO directed acyclical graph. These sets with added ancestors served as a starting point of our tree-building analyses: pairwise distances between the genomes were calculated using the Jaccard distance as a metric of the dissimilarity between any 2 sets *a* and *b*.

(1) $$\begin{equation*}
d_{ab} = 1 - \frac{|S_a \cap S_b|}{|S_a \cup S_b|}
\end{equation*}
$$

An NJ tree was constructed on the basis of the generated pairwise distance matrix using PHYLIP (PHYLIP, RRID:SCR_006244) [53]. Additionally, term sets *S* of all genomes were combined into a binary matrix (with rows corresponding to genomes and columns corresponding to GO terms, values of 0 or 1 indicating whether a term is present or absent in the given set). PHYLIP pars was used to construct a parsimony tree from this binary matrix.

*P. lambertiana*, a gymnosperm, was included in the dataset as an outgroup to the angiosperms to separate between the monocot and eudicot clades. iTOL (iTOL, RRID:SCR_018174) [94] was used to visualize the trees using their Newick format, and root them at *P. lambertiana*. Moreover, a cladogram representing the known phylogeny of the included taxa was created by hand based on known evolutionary relationships [95–99]. This was used to compare the generated phylogenetic relationship based on functional similarity with the evolutionary relationships of the plant genomes.

Jackknifing analysis was carried out for both parsimony and NJ trees to assess the support for each clade on the basis of the proportion of jackknife trees showing the same clade. To this end, 40% of the terms in *T* were randomly removed, ancestors of the remaining terms were added, and trees constructed as above. The majority rule consensus tree of 100 individual trees was calculated with the jackknife values represented on each branch. The tree was then visualized using iTOL using its Newick format, and rooted again at *P. lambertiana*.

### Genome quality evaluation

Genome size was estimated from the C-values obtained from the Plant DNA C-values data resource from the Kew Database [100]. The mean C-value for a given species was used for calculating genome size estimates in base pairs using the method of [101]. In brief,

$$\begin{equation*}
\textrm {Genome~size~(bp)} = \textrm {C-value~(pg)} * 0.978 * 10^9 \frac{\textrm {bp}}{\textrm {pg}}.
\end{equation*}
$$

The estimated genome size (listed in Table 4) was used as an input for GenomeQC [63,102] to calculate quality metrics. For genomes that were too large to submit through the GenomeQC webtool or had missing exon information, modified scripts of those found in GitHub of GenomeQC (commit e6140ee [103]) were applied to calculate the assembly and structural annotation metrics in Tables 4 and 5. BUSCO version 5.2.2 (BUSCO, RRID:SCR_015008) [104] was used to calculate the assembly and annotation BUSCO scores, shown in Figs. 6 and 7. inputs for assembly BUSCO scores were chromosome sequences, whereas inputs were transcript/messenger RNA/CDS sequences for the annotation BUSCO scores. For the lineage parameter, the lineage datasets used were as follows: Eudicots for *C. sativa* and *G. raimondii*, Fabales for *A. hypogaea, M. truncatula* A17 and R108, *P. vulgaris, G. max*, and *V. unguiculata*, and Poales for *B. distachyon, O. sativa, T. aestivum, H. vulgare, S. bicolor*, and *Z. mays* B73, Mo17, W22, and PH207.

## Data Availability

All data and source code generated are freely available at [105] under the terms of the CC0 license (also archived at [106]). All software requirements and dependencies are packaged into a Singularity container (now renamed as Apptainer) so no other set-up is required to reproduce our results (container available at [107]; download and use instructions are included in the repository README).

An up-to-date list of all available annotation sets can be found at [49], and the GOMAP software used to generate them is available at [108].

## Additional Files

**Supplementary Table S1**: Additional assembly statistics from GenomeQC.

**Supplementary Table S2**: Number of removed annotations during clean-up.

**Supplementary Figure S1**: Number of total annotations in each GO IEA dataset analyzed, colored by GO aspect (cellular component in green, molecular function in orange, and biological process in blue). Species are ordered on the basis of the number of GO terms in the GOMAP dataset, with the species producing the most GO terms (*Triticum*) on top and the least (*Pinus*) on bottom.

**Supplementary Figure S2**: Quality scores of the predicted annotation visualized as a gray-scale heat map. Each row represents a single species from a single data source. Input gene model dataset origin is indicated: blue for GOMAP, yellow for Gramene63, and orange for Phytozome12. Across the top are the 3 metric types used to assess the annotation quality. Across the bottom are subgraph indicators: C is cellular component, F is molecular function, and P is biological process. Darker cells indicate a higher (better) score whereas lighter cells indicate a lower score. Note that scales are different for each metric type (meaning that comparisons across the 3 metric types are not meaningful). Rows are clustered by pairwise correlation/similarity across all metrics. The dendrogram at left retraces clustering.

**Supplementary Figure S3**: Neighbor-joining trees built on annotation subsets per GO aspect. Phylograms are colored and rooted as described in Fig. 2. For each tree, only the annotations from the respective aspect of the Gene Ontology were used.

giac023_GIGA-D-21-00269_Original_Submission

giac023_GIGA-D-21-00269_Revision_1

giac023_GIGA-D-21-00269_Revision_2

giac023_Response_to_Reviewer_Comments_Revision_1

giac023_Response_to_Reviewer_Comments_Revision_2

giac023_Reviewer_1_Report_Original_Submission Leonore Reiser -- 10/11/2021 Reviewed

giac023_Reviewer_1_Report_Revision_1 Leonore Reiser -- 1/7/2022 Reviewed

giac023_Reviewer_2_Report_Original_Submission Alexandre R. Paschoal -- 10/12/2021 Reviewed

giac023_Supplemental_Figures_and_Tables

## Abbreviations

ADS: artificial dilution series; BOP clade: Bambusoideae, Oryzoideae, and Pooideae; bp: base pairs; BUSCO: Benchmarking Universal Single-Copy Orthologs; CAFA: Critical Assessment of Functional Annotation; CDS: coding sequence; DOI: Digital Object Identifier; EXP evidence code: Inferred from Experiment; GAF: GO Annotation File; GO: Gene Ontology; GOA: Gene Ontology Annotation; GOMAP: Gene Ontology Meta Annotator for Plants; IEA evidence code: Inferred from Electronic Annotation; IEP evidence code: Inferred from Expression Pattern; iTOL: Interactive Tree of Life; IUPAC: International Union of Pure and Applied Chemistry; KEGG: Kyoto Encyclopedia of Genes and Genomes; Mb: megabase pairs; NJ: neighbor joining; NPAAA: non-protein amino acid-accumulating clade; NSF: National Science Foundation; PACMAD clade: Panicoideae, Arundinoideae, Chloridoideae, Micrairoideae, Aristidoideae, and Danthonioideae; PHYLIP: PHYLogeny Inference Package; TC-AUCPCR: term-centric area under precision-recall curve.

## Competing Interests

The authors declare that they have no competing interests.

## Funding

This work has been supported by the Iowa State University Plant Sciences Institute Faculty Scholars Program to C.J.L.D., the Predictive Plant Phenomics NSF Research Traineeship (No. DGE-1545453) to C.J.L.D. (C.F.Y. and K.O.C. are trainees), the AI Research Institutes program supported by NSF and USDA-NIFA under AI Institute: for Resilient Agriculture (Award No. 2021-67021-35329) to C.J.L.D. (supporting L.F.), and IOW04714 Hatch funding to Iowa State University.

## Authors' Contributions

L.F., D.P., C.F.Y., K.O.C., H.A.D., P.J., D.C.S., H.V., and K.W. generated annotations for plants as described in this article. D.P. and C.J.L.D. co-conceived the idea for phylogenetic analysis. D.P. wrote the code for the analyses in this article. L.F. worked with D.P. to create dendrograms and compare those to phylogenetic trees. L.F. carried out assembly and annotation metric comparisons. L.F., D.P., and C.J.L.D. wrote the manuscript. All authors read, offered suggestions to improve, and approved the final copy of the manuscript.

## Authors’ Information

K.W. created the GOMAP system during his time as a graduate student at Iowa State University. L.F., D.P., C.F.Y., K.O.C., H.V., and P.J. are currently graduate students. D.P. attended Iowa State University for 2 semesters on a Study Grant from the German-American Fulbright Commission. H.A.D. and D.C.S. are undergraduate students. Each graduate and undergraduate student annotated ≥1 genome over the course of a research rotation lasting ≤1 semester. C.J.L.D. coordinated research activities and manuscript preparation.
