# Supplementary material for: Standardized genome-wide function prediction enables comparative functional genomics: a new application area for Gene Ontologies in plants
Source: Gigascience. 2022 Apr 15;11:giac023. doi: 10.1093/gigascience/giac023 (PMC9012101; doi:10.1093/gigascience/giac023)
Supplement: giac023_GIGA-D-21-00269_Original_Submission [file giac023_giga-d-21-00269_original_submission.pdf]

GigaScience

Standardized genome-wide function prediction enables comparative functional genomics: a new application area for Gene Ontologies in plants

--Manuscript Draft--

**Manuscript Number:**	GIGA-D-21-00269
**Full Title:**	Standardized genome-wide function prediction enables comparative functional genomics: a new application area for Gene Ontologies in plants
**Article Type:**	Research
**Funding Information:**	Iowa State University Plant Sciences Institute — Dr. Carolyn J Lawrence-Dill<br>National Science Foundation (DGE-1545453) — Dr. Carolyn J Lawrence-Dill<br>Iowa State University Agricultural Experiment Station (Hatch Funds) (IOW04714) — Dr. Carolyn J Lawrence-Dill
**Abstract:**	Background: Genome-wide gene function annotations are useful for hypothesis generation and for prioritizing candidate genes potentially repsonsible for phenotypes of interest. We functionally annotated the genes of 18 crop plant genomes across 14 species using the GOMAP pipeline.<br>Results: By comparison to existing GO annotation datasets (available for a subset of these genomes), GOMAP-generated datasets cover more genes, contain more GO terms, and produce datasets similar in quality (based on precision and recall metrics using existing gold standards as the basis for comparison). From there, we sought to determine whether the datasets across multiple species could be used together to carry out comparative functional genomics analyses in plants. To test the idea and as a proof of concept, we created dendrograms of functional relatedness based on terms assigned for all 18 genomes. These dendrograms were compared to well-established species-level evolutionary phylogenies to determine whether trees derived were in agreement with known evolutionary relationships, which they largely are. Where discrepancies were observed, we determined branch support based on jack-knifing then removed individual annotation sets by genome to identify the annotation sets causing unexpected relationships.<br>Conclusions: GOMAP-derived functional annotations used together across multiple species generally retain sufficient biological signal to recover known phylogenetic relationships based on genome-wide functional similarities, indicating that comparative functional genomics across species based on GO data hold promise for generating novel hypotheses about comparative gene function and traits.
**Corresponding Author:**	Carolyn J Lawrence-Dill, Ph.D.<br>Iowa State University of Science and Technology: Iowa State University<br>Ames, IA UNITED STATES
**Corresponding Author Secondary Information:**	
**Corresponding Author's Institution:**	Iowa State University of Science and Technology: Iowa State University
**Corresponding Author's Secondary Institution:**	
**First Author:**	Leila Fattel
**First Author Secondary Information:**	
**Order of Authors:**	Leila Fattel<br>Dennis Psaroudakis<br>Colleen F. Yanarella<br>Kevin Chiteri<br>Haley A. Dostalik

Powered by Editorial Manager® and ProduXion Manager® from Aries Systems Corporation

	Parnal Joshi
	Dollye C. Starr
	Ha Vu
	Kokulapalan Wimalanathan, Ph.D.
	Carolyn J Lawrence-Dill, Ph.D.
**Order of Authors Secondary Information:**	
**Additional Information:**	
**Question**	**Response**
Are you submitting this manuscript to a special series or article collection?	No
**Experimental design and statistics**<br><br>Full details of the experimental design and statistical methods used should be given in the Methods section, as detailed in our Minimum Standards Reporting Checklist. Information essential to interpreting the data presented should be made available in the figure legends.<br><br>Have you included all the information requested in your manuscript?	Yes
**Resources**<br><br>A description of all resources used, including antibodies, cell lines, animals and software tools, with enough information to allow them to be uniquely identified, should be included in the Methods section. Authors are strongly encouraged to cite Research Resource Identifiers (RRIDs) for antibodies, model organisms and tools, where possible.<br><br>Have you included the information requested as detailed in our Minimum Standards Reporting Checklist?	Yes
**Availability of data and materials**<br><br>All datasets and code on which the conclusions of the paper rely must be	Yes

either included in your submission or deposited in publicly available repositories (where available and ethically appropriate), referencing such data using a unique identifier in the references and in the "Availability of Data and Materials" section of your manuscript.<br><br>Have you have met the above requirement as detailed in our Minimum Standards Reporting Checklist?	

Powered by Editorial Manager® and ProduXion Manager® from Aries Systems Corporation

Click here to access/download;Manuscript;Comparative_Functional_Genom

Standardized genome-wide function prediction enables comparative functional genomics: a new application area for Gene Ontologies in plants

Leila Fattel[1,*], Dennis Psaroudakis[2,*,†], Colleen F. Yanarella[1,‡], Kevin Chiteri[1,‡], Haley A. Dostalik[1,‡], Parnal Joshi[4,‡], Dollye C. Starr[1,‡], Ha Vu[5,‡], Kokulapalan Wimalanathan[5,§], and Carolyn J. Lawrence-Dill[1,5,¶]

[1] *Department of Agronomy, Iowa State University*
[2] *Department of Plant Pathology and Microbiology, Iowa State University*
[3] *Department of Ecology, Evolution and Organismal Biology, Iowa State University*
[4] *Department of Veterinary Microbiology and Preventative Medicine, Iowa State University*
[5] *Department of Genetics, Development and Cell Biology, Iowa State University*

(Dated: September 2, 2021)

Background Genome-wide gene function annotations are useful for hypothesis generation and for prioritizing candidate genes potentially repsonsible for phenotypes of interest. We functionally annotated the genes of 18 crop plant genomes across 14 species using the GOMAP pipeline.

Results By comparison to existing GO annotation datasets (available for a subset of these genomes), GOMAP-generated datasets cover more genes, contain more GO terms, and produce datasets similar in quality (based on precision and recall metrics using existing gold standards as the basis for comparison). From there, we sought to determine whether the datasets across multiple species could be used together to carry out comparative functional genomics analyses in plants. To test the idea and as a proof of concept, we created dendrograms of functional relatedness based on terms assigned for all 18 genomes. These dendrograms were compared to well-established species-level evolutionary phylogenies to determine whether trees derived were in agreement with known evolutionary relationships, which they largely are. Where discrepancies were observed, we determined branch support based on jack-knifing then removed individual annotation sets by genome to identify the annotation sets causing unexpected relationships.

Conclusions GOMAP-derived functional annotations used together across multiple species generally retain sufficient biological signal to recover known phylogenetic relationships based on genome-wide functional similarities, indicating that comparative functional genomics across species based on GO data hold promise for generating novel hypotheses about comparative gene function and traits.

Keywords: Gene function; ontology; plants; comparative genomics; functional genomics

I. BACKGROUND

Phenotypes and traits that have long been the primary inspiration for biological investigation. Phenotypes are the result of a complex interplay between functions of genes and environmental cues. In an effort to organize and model gene functions, various systems of classification have been developed including systems like KEGG (the Kyoto Encyclopedia of Genes and Genomes), which is focused on protein function including gene activities superimposed on metabolic pathways [1]. Other such systems include the various Cyc databases, MapMan, and the Gene Ontologies (GO), a vocabulary of gene functions organized as a directed acyclic graph, which makes it innately tractable for computational analysis [2, 3, 4].

GO-based gene function annotation involves the association of GO terms to individual genes. Functions may be assigned to genes based on different types of evidence for the association. For example, functional predictions can be inferred from experiments (EXP), expression patterns (IEP), and more [5]. Computational pipelines are often used to generate functional predictions for newly sequenced genomes, where the genome is first sequenced and assembled, then gene structures (gene models) are predicted, then functions are associated with those gene predictions. Genome-wide gene function prediction datasets are frequently used to analyze gene expression studies, to prioritize candidate genes linked to a phenotype of interest, to design experiments aimed at characterizing functions of genes, and more [6, 7, 8]. How well a gene function prediction set models reality is influenced by how complete and correct the underlying genome assembly and gene structure annotations are as well as by how well the software used to predict functions performs.

GOMAP (the Gene Ontology Meta Annotator for Plants) is a gene function prediction pipeline for plants that generates high-coverage and reproducible functional annotations [9]. The system employs multiple functional prediction approaches, including sequence similarity, protein domain presence, and mixed-method pipelines developed to compete in the Critical Assessment of Function Annotation (CAFA) Challenge [10], a community challenge that has advanced the performance of gene function prediction pipelines over the course of five organized competitions [11].

We previously annotated gene functions for the maize

*Contributed equally.
†Current address: Department of Molecular Biology, Leibniz Institute of Plant Genetics and Crop Plant Research (IPK), 06466 Seeland, OT Gatersleben, Germany
‡Listed alphabetically.
§kokul@bioinformapping.com
¶triffid@iastate.edu

B73 genome and demonstrated that GOMAP's predicted functions were closer to curated gene-term associations from the literature than those of other community functional annotation datasets ([12]). Using the newly containerized GOMAP system ([9]), we report here the functional annotation of 18 plant genomes across the 14 crop plant species shown in Table I.

Given these multiple annotations across various plant species, we next considered whether these datasets could be used together for comparative functional genomics in plants. We describe here a simple and crude method by which we used gene function annotations to generate dendrograms of genome-level similarity in function. This idea is similar to that of Zhu et al., who determined the evolutionary relationships among microorganisms based on whole-genome functional similarity [13]. Here we expand on that approach, analyzing genome-wide GO assignments to generate parsimony and distance-based dendrograms (see Figure 1 for process overview). We compared these with well-established species phylogenies (Figure 2) to determine whether trees derived from gene function show any agreement with evolutionary histories, taking agreement between generated dendrograms and known evolutionary histories to be evidence that sufficient comparative biological signal exists to begin to use GO functional annotations across multiple plant genomes for comparative functional genomics investigations.

II. RESULTS OF ANALYSES

A. Overview

As shown in Figure 1, gene function annotation sets were created and compiled for each genome. For those with existing annotation sets available on Gramene or Phytozome [32, 33], the datasets were compared. From there, matrices that included genomes as rows and terms as columns were generated. These were used directly to build parsimony trees or to create distance matrices for neighbor-joining tree construction [34, 35, 36]. In subsequent analyses, jackknifing was used to remove terms (columns) or to remove genomes (rows) to map the source of signal for treebuilding results [37].

B. Functional Annotation Sets Produced

Table II shows quantitative attributes of each of the annotation sets. In summary, GOMAP covers all annotated genomes with at least one annotation per gene, and provides between 3.8 and 12.1 times as many annotations as Gramene or Phytozome.

Quality evaluation of gene function predictions is not trivial and is approached by different research groups in differnet ways. Most often datasets are assessed by comparing the set of predicted functions for a given gene to a Gold Standard consisting of annotations that are assumed to be correct. This assumption of correctness can be based on any number of criteria. Here we used as our Gold Standard dataset all annotations present in Gramene that had a non-IEA (non-Inferred by Electronic Annotation) evidence code, i.e. we used only annotations that had some manual curation. This enabled us to assess the 10 genomes shown in Table II. It is perhaps noteworthy that the IEA and non-IEA annotation sets from Gramene frequently contain overlaps, indicating that some of the predicted annotations were manually confirmed afterwards by a curator and that in such cases, a new annotation was asserted with the new evidence code rather than simply upgrading the evidence code from IEA to some other code, thus preserving the IEA annotations in Gramene that are produced by the Ensembl analysis pipeline [38], a requirement for comparing GOMAP-produced IEA datasets to the IEA datasets produced by the Ensembl pipeline.

There are many different metrics that have been used to evaluate the quality of predicted functional annotations. For the maize B73 GOMAP annotation assessment in [12], we had used a modified version of the hierarchical evaluation metrics originally introduced in [39] because they were simple, clear, and part of an earlier attempt at unifying and standardizing GO annotation comparisons [40]. In the meantime, Plyusnin et al. published an approach for evaluating different metrics showing variation among the robustness of different approaches to quality assessment [41]. Based on their recommendations, we use here the SimGIC2 and Term-centric Area Under Precision-Recall Curve (TC-AUCPCR) metrics. We also evaluated with the F_{max} metric, simply because it is widely-used (e.g., by [10]), even though according to Plyusnin et al., it is actually a flawed metric [41]. Results of the quality assessments for the 10 genomes where a Gold Standard was available are shown in Table III and Figure S1. While evaluation values differ between metrics and the scores are not directly comparable, a few consistent patterns emerge: GOMAP annotations are almost always better than Gramene and Phytozome annotations in the Cellular Component and Molecular Function aspect, with the only three exceptions being the Molecular Function aspect for *T. aestivum* using the TC-AUCPCR and the F_{max} metric and the Cellular Component aspect for *M. truncatula* A17 using the F_{max} metric. Conversely, GOMAP predictions achieve consistently lower quality scores in the Biological Process aspect with the exception of *B. dystachion*, *O. sativa*, and *S. bicolor* with the TC-AUCPR metric. Generally, annotations that are better in one aspect are also better in the other two aspects (there are few cross-overs shown in Figure SI), but the ranking of annotations does not necessarily hold across metrics. The Phytozome annotation for *O. sativa* is an outlier in terms of its comparative quality, potentially because it is based on a modified structural annotation that differs substantially from the Gold Standard and the other annotations under comparison.

Table I: Functional annotation sets generated by GOMAP. More information about each dataset including the source of the input to GOMAP can be found at the respective DOI.

Species	Germplasm/Line	Assembly/Annotation	Dataset DOI
Arachis hypogaea	Tifrunner	Arachis hypogaea assembly 1.0	[14]
Brachypodium distachyon	Bd21	Bd21.v3.1.r1	[15]
Cannabis sativa	Hemp	NCBI Cannabis sativa GCA_900626175.1	[16]
Glycine max	Williams 82	Joint Genome Institute (JGI) Wm82.a4.v1	[17]
Gossypium raimondii	Cotton D	Gossypium raimondii JGI v2.1	[18]
Hordeum vulgare	–	IBSC_PGSB_r1	[19]
Medicago truncatula	R108_HM340	R108: v1.0	[20]
Medicago truncatula	A17_HM341	Mt4.0v2	[21]
Oryza sativa	japonica	IRGSP 1.0	[22]
Phaseolus vulgaris	G19833	DOE-JGI and USDA-NIFA annotation 2.0	[23]
Pinus lambertiana	Sugar Pine	TreeGenesDB sugar pine assembly v1.5	[24]
Sorghum bicolor	BTx623	BTx623.v3.0.1.r1	[25]
Triticum aestivum	Chinese Spring	IWGSC RefSeq 1.1	[26]
Vigna unguiculata	IT97K-499-35	JGI annotation v1.1	[27]
*Zea mays**	Mo17	Zm-Mo17-REFERENCE-CAU-1.0	[28]
*Zea mays**	PH207	Zm-PH207-REFERENCE_NS-UIUC_UMN-1.0	[29]
*Zea mays**	W22	Zm-W22-REFERENCE-NRGENE-2.0 Zm00004b.1	[30]
*Zea mays**	B73	RefGen_V4 Zm00001d.2	[31]

Latest overview at https://dill-picl.org/projects/gomap/gomap-datasets/
* Previously published in [9].

C. Phylogenetic Tree Analyses

With the comparative quality of gene function predictions in hand, we approached the question of whether the datasets could be used together for comparative functional analysis across all genomes. As a simple first step, we began work toward understanding the degree to which trees built based on gene functions agree with known, well-documented evolutionary relatedness. We constructed neighbor-joining and parsimony trees of the 18 plant genomes, and visulized them using iTOL [42]. The two tree topologies, rooted at *P. lambertiana*, were compared to one another and to the topology of the expected tree (Figure 2). For both the neighbor-joining (Figure 3a) and parsimony trees (Figure 3b), one common difference is noted: *S. bicolor* is not at the base of the *Z. mays* clade as expected, and is clustered with *B. distachyon* instead. Notable differences between the neighbor-joining and parsimony tree are the following: *C. sativa* appears at the base of the eudicots instead of *G. raimondii* in the neighbor-joining tree, while *G. raimondii* is grouped with *A. hypogaea* and *C. sativa* is grouped with *G. max* in the parsimony tree. Second, *O. sativa* was expected to be at the base of the BOP clade, but appears at the base of *Z. mays* in the neighbor-joining tree, but at the base of all angiosperms in the parsimony tree. Differences among relationships within the *Z. mays* clade constaining B73, PH207, W22, and Mo17 were disregarded given the high degree of similarity across annotation sets and the fact that these relationships are not clear given the complex nature of within-species relationships.

Due to differences between the function-based dendrograms and the expected tree, jackknifing analysis was carried out by removing terms (columns in underlying datasets) to determine the degree to which the underlying datasets support specific groupings based on functional term assignments. This analysis was carried out for both neighbor-joining and parsimony trees. First, trees were generated by omitting 5%, then 10%, then 15% on up to 95% of the dataset to determine the threshhold at which the tree topologies deviated from those generated using the full dataset. That threshold was reached at 45% for both neighbor-joining and parsimony; therefore we used trees generated with 40% of the data removed for reporting branch support for the topology (Figure 3). Comparing the two trees, the topologies were similar at jackknife values up to 40% but the support values for parsimony were comparatively lower. Based on this robustness for neighbor-joining treebuilding in general, we carried out all subsequent analyses using neighbor-joining treebuilding methods.

To map the source of discrepancies to specific gene annotation sets, we generated various neighbor-joining trees excluding one genome each time, an additional tree with both *Medicago* genomes excluded simultaneously, and another with all *Z. mays* genomes excluded simultaneously. To exemplify this, see the monocot clade in Figure 2 and the lower (monocot) clade in Figure 3a. When the neighbor-joining tree was generated, two species are misplaced: *S. bicolor* and *O. sativa.* As shown in Figure 4a, removal of *O. sativa* corrects one error (itself) but

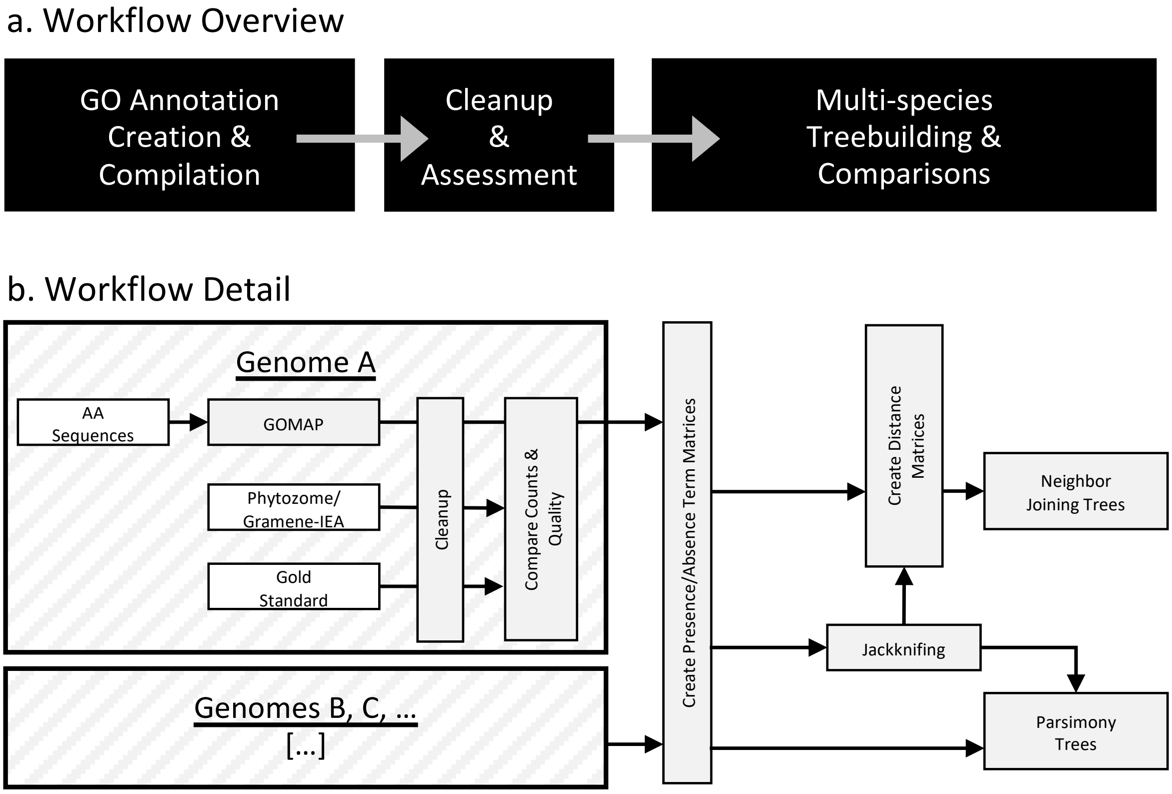

Figure 1: Data workflow schema. The workflow overview is shown in panel 'a' with steps represented as black boxes and the flow of information and processes indicated by arrows. Details are shown in panel 'b' where the upper large hatched box shows process detail for a single genome and the lower hatched box represents additional genomes for which the details of processing are identical. White boxes represent input datasets. Arrows indicate the flow of information and processes.

does not correct the errant grouping of *S. bicolor* with *B. distachyon*. In 4b, it is shown that the removal of *S. bicolor* corrects the errant grouping of itself and *B. distachyon*, but *O. sativa* placement remains incorrect. However, as shown in 4c, the removal of *B. distachyon* generates a tree where all relationships are in agreement with known species-level relationships. (Note well: all individual annotation sets were progressively removed, not just these three shown in the example.)

With this observation in hand, we sought to determine the minimum number of genomes that could be removed to create a tree that matched the expected tree topology. The removal of the three genomes was required to generate function-based trees consistent with known phylogenetic relationships. They are *C. sativa*, *G. max*, and *B. distachyon* (Figure 5). Jackknifing analysis was also carried out for this dataset with support shown. Branch support is generally higher than that for the full dataset (i.e., branch support is higher in Figure 5 than in Figure 3a).

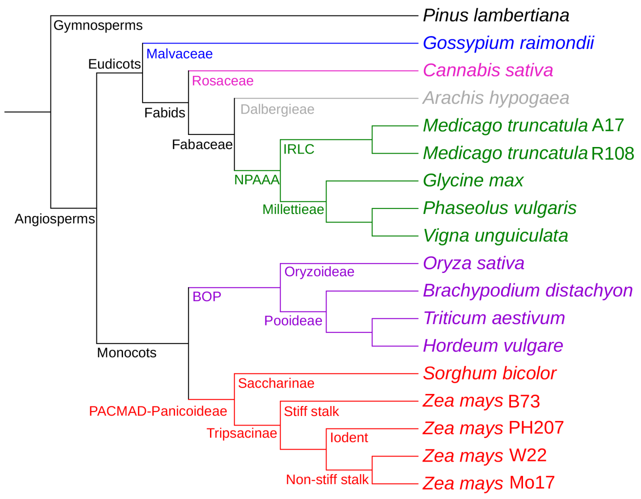

Figure 2: Known phylogenetic relationships among species. Cladogram is rooted by the gymnosperm *Pinus lambertiana* (black). Among angiosperms, eudicots clades include Malvaceae (blue), Rosaceae (magenta), Dalbergieae (grey), and NPAAA (green). Monocots include members of the BOP (purple) and PACMAD-Panicoideae (red) clades.

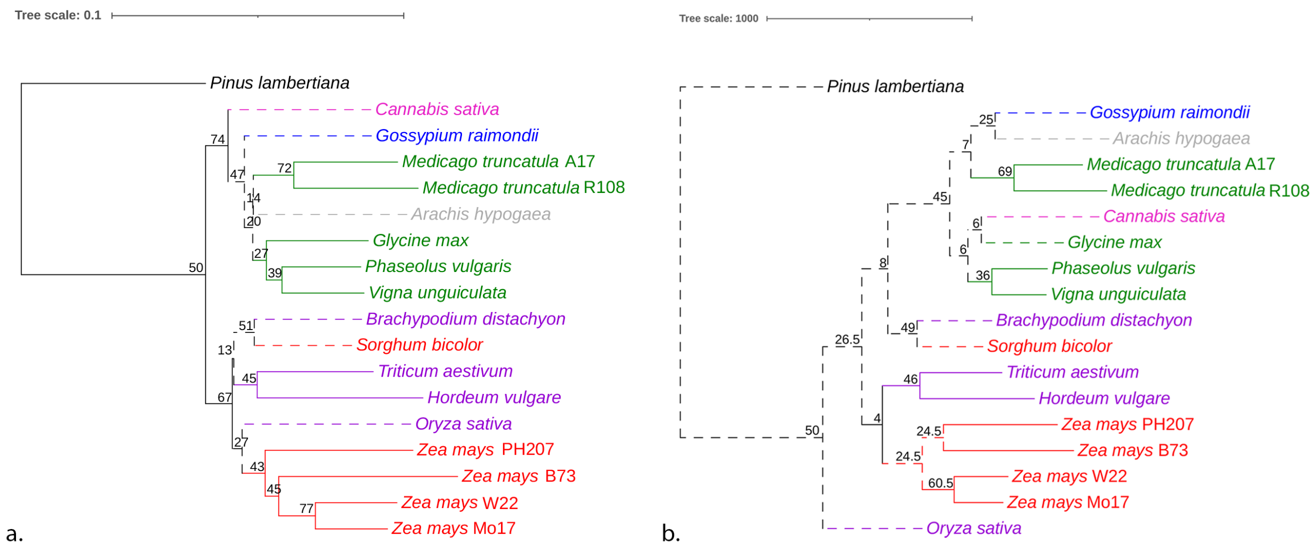

Figure 3: Neighbor-joining and parsimony trees. Phylograms are colored and rooted as described in Figure 2. For both neighbor-joining (a) and parsimony (b), node values represent the jackknifing support values derived by removing 40% of GO terms in the dataset. Dashed lines mark deviations from known phylogenetic relationships. Tree scales are shown above each, with NJ showing distances and parsimony showing changes in character state.

D. Potential Causes of Unexpected Groupings

As a first step in investigating whether and how the comparative quality of assemblies and annotation sets underlying the predicted gene function datasets could explain discrepancies between known evolutionary relationships and those resulting from comparative analysis of genome-wide gene function predictions, we assessed the quality of each genome assembly and structural annotation set using GenomeQC [43]. Tables IV-V and Figures 6-**??** represent the resulting assembly quality, structural annotation measures of quality, and proportion of single-copy BUSCOs (Benchmarking Universal Single-Copy genes) [44] that were generated. Although these analyses make evident that the species annotated are comparatively different in both natural genome characteristics and in assembly in annotation quality aspects, it is not the case that the three species responsible for deviations between the functional annotation dendrograms and known phylogenetic relationships (i.e., *C. sativa*, *G. max*, and *B. distachyon*) create these discrepancies due to issues of genome assembly and/or annotation quality. The one potential for some explanation is in relation to *C. sativa*, which is the only genome that has an assembly length larger than the expected (see IV), and a comparatively large proportion of missing BUSCOS in the assembly (see **??**).

III. DISCUSSION

In this study, we used the GOMAP pipeline to produce whole-genome GO annotations for eighteen genome assembly and annotation sets from fourteen plant species [9]. Assessments of the number of terms predicted as well as the quality of predictions indicate that GOMAP functional prediction datasets cover more genes, contain more predictions per gene, and are of similar quality to prediction datasets produced by other systems, thus supporting the notion that these high-coverage datasets are a useful addition for researchers who are interested in genome-level analyses, including efforts aimed at prioritizing candidate genes for downstream analyses. Given that we can now produce high-quality, whole genome functional annotations for plants in a straightforward way, we intend to produce more of these over time (indeed we recently annotated *Vitis vinifera* Pinot Noir grape [45], *Brassica rapa* [46], *Musa acuminata*[47], *Theobroma cacao* [48], *Coffea canephora* [49], *Vaccinium corymbosum* [50] *Solanum lycopersicum* [51], and *Solanum pennellii* [52]).

With eighteen genome functional annotations in hand, we sought to determine whether and how researchers could use multispecies GO annotation datasets to perform comparative functional genomics analyses. As a proof of concept, we adapted phylogenetic tree-building methods to use the gene function terms assigned to genes represented by the genomes to build dendrograms of functional relatedness and hypothesized that if the functions were comparable across species, the resulting trees would closely match evolutionary relationships. To our

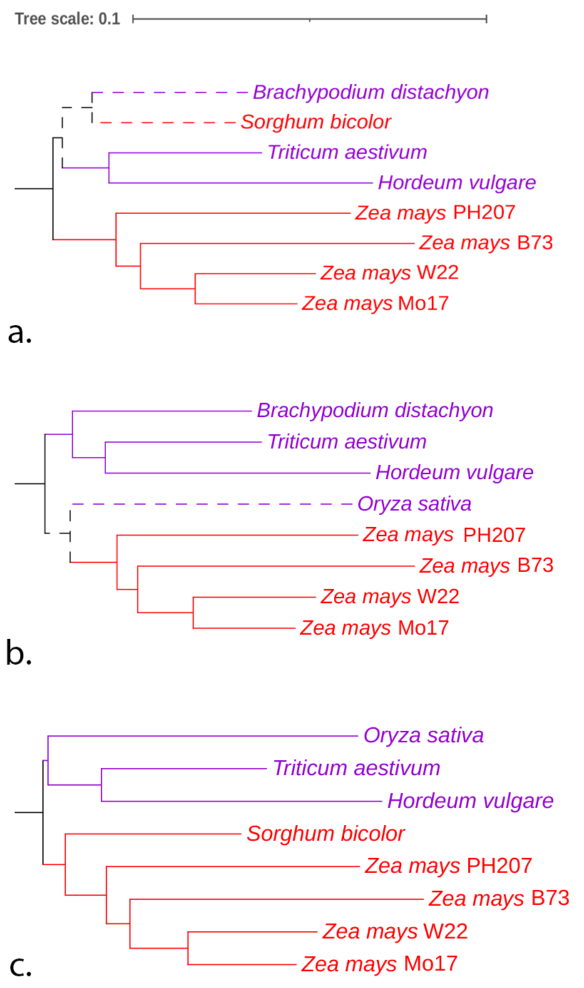

Figure 4: Restoring monocot relationships. Phylograms are colored and rooted as described in Figure 2. Dashed lines mark deviations from known phylogenetic relationships. Monocot topology changes with removal of a single species: a) *O. sativa*, b) *S. bicolor*, and c) *B. distachyon*. Tree scale is shown above.

delight and surprise, the neighbor-joining and parsimony trees (Figure 3) did resemble known phylogenies, but were not exact matches to broadly accepted phylogenetic relationships.

After removing the minimum number of genomes that resulted in restoration of the expected evolutionary relationships, we found that the individual species that may be responsible for the discrepancies observed in Figure 3 were *C. sativa*, *G. max*, and *B. distachyon*. We hypothesize that the following could account for such errant relationships:

1) Quality of sequencing and coverage assembly: genomes of similarly high sequence coverage that have excellent gene calling would be anticipated to create the best source for functional annotation. Genomes of comparatively lower, or different, character would be anticipated to mislead treebuilding and other comparative ge-

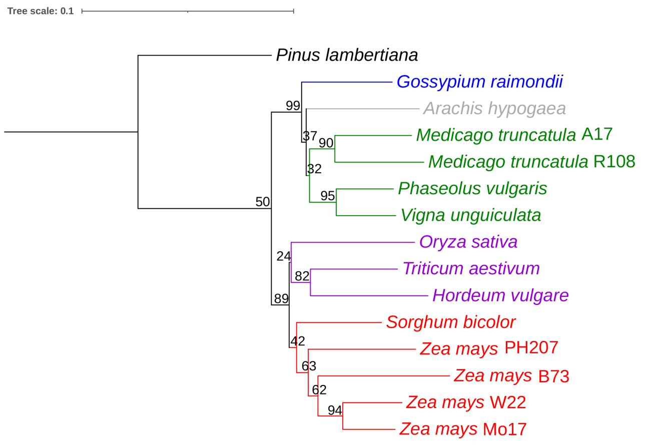

Figure 5: Restoring known phylogentic relationships to the NJ tree via removal of a minimal number of species. Phylograms are colored and rooted as described in Figure 2. Node values represent the jackknifing support values derived by removing 40% of GO terms in the dataset. 3 genomes have been removed: *C. sativa*, *G. max*, and *B. distachyon*. Tree scale is shown above.

nomics approaches.

2) Shared selected or natural traits: species that have been selected for, e.g., oilseeds may share genes involved in synthesis of various oils. Other shared traits would be anticipated to cause similarities for species with those shared traits.

3) The simple method of treebuilding based on the presence or absence of gene function terms is not sophisticated enough to recover the full detail of the species' evolutionary histories.

To consider the first of these, we looked at genome assembly and annotation quality metrics (see Tables IV-V and Figures 6-**??**). For *G. max* and *B. distachyon* we could find no compelling evidence that assembly structural or functional annotation quality differed significantly from all others, except in the case of *C. sativa*, where we noted that the assembly length exceeded the predicted genome size based on C-values for genome sizes reported previously [53]. In this case, the fact that the *C. sativa* line sequenced is not inbred [54] may be responsible for the inflated assembly size relative to what is expected. This means that in the assembly, there are likely regions where alleles between chromosomes do not aligned, which would inflate the overall length of the assembly. In addition, the assembly misses a large proportion of BUSCO genes compared to most other genomes included in this analysis. Indeed, the comparatively low-quality assembly for *Cannabis* genome has been noted by others [55], and our preliminary investigations indicate that the assembly length is in fact longer than expected .

In an attempt to better understand conflicting phylogenetic signals that could be caused by the second potential cause, i.e., shared selected or natural traits, we

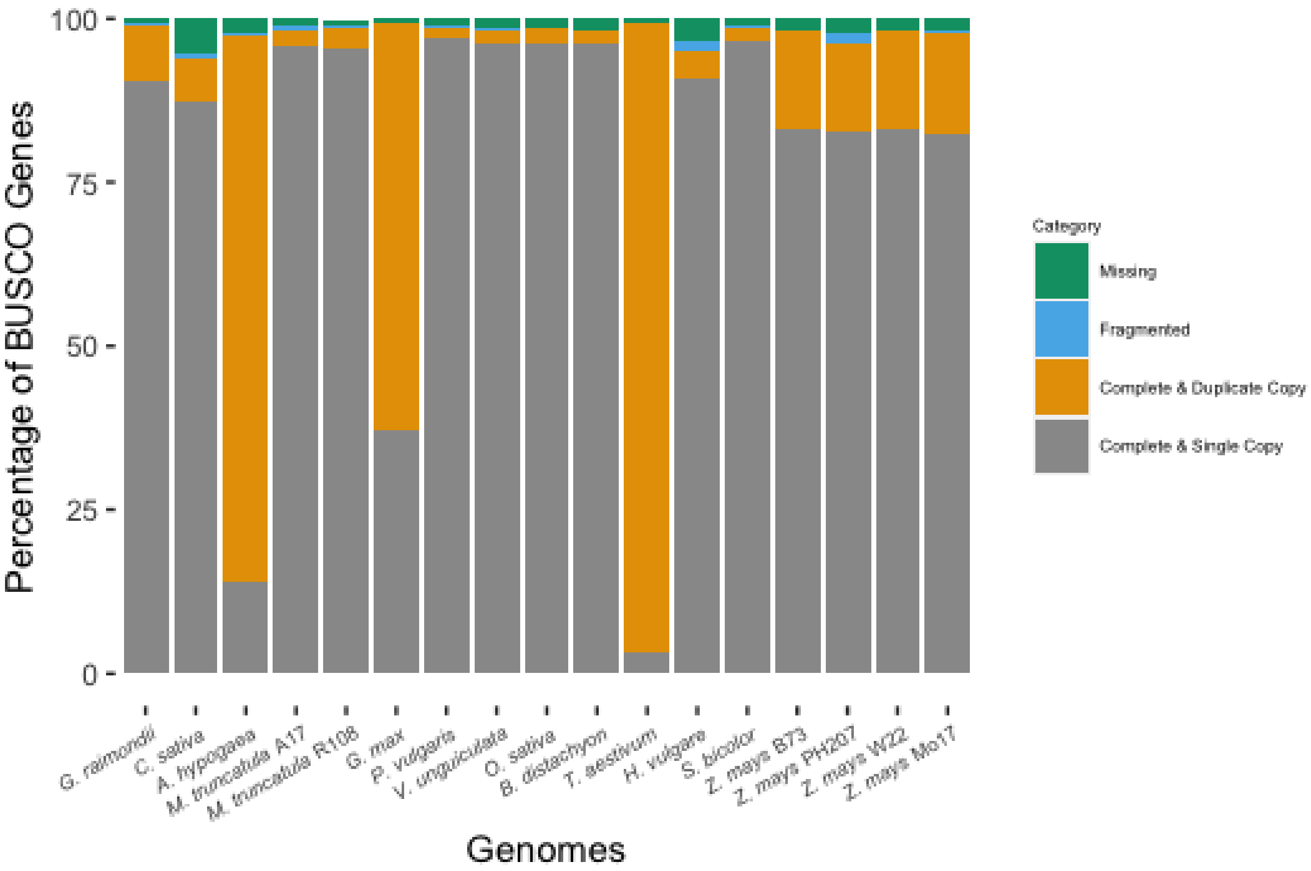

Figure 6: Assembly BUSCO plot generated using GenomeQC. Genomes analyzed are shown across the X-axis, and are ordered to match the occurrence of species shown in Figure 2. Percentage of BUSCO genes across four gene categories are stacked, with each adding up to 100 percent (Y-axis). Complete and single-copy genes are shown in grey, complete and duplicated copies in orange, fragmented copies in blue, and missing are shown copies in green.

mapped all GO terms that exist in our binary matrix and traced character history (presence/absence) on the nodes and leaves of our expected evolutionary tree using the software Mesquite version 3.61 [56]. These data summarize the gain or loss of each GO term across the species described in this paper and can be found in our Github repository. We carried out a number of simple experiments to reveal which terms could be causal for errant relationships (e.g., dropping all unique terms from the *B. distachyon* or *G. max* datasets, reconstructing the term states at nodes that should be where *B. distachyon* and *G. max* should occur, etc., and could not identify any biologically compelling patterns. (Because these analyses were not fruitful, they were not specifically included in our materials and methods, though we do include the input datasets here for others to consider and peruse independently.)

We consider the most likely explanation for discrepancies between the known evolutionary phylogenies and dendrograms created based on GO terms describing gene function to be the third explanation: the simplicity of the treebuilding models and methods we used for these analyses. Because the treebuilding and analytics described in this paper were based on the presence/absence of GO terms, novel terms are highly influential to the outcomes of the analysis. In contrast, plant genomes are notable for having many duplicated genes as a result of whole-genome and segmental duplications. Therefore, using presence/absence of GO terms where the count of term occurrences are not weighted may be too simple a method to get at the genuine biological complexities represented in any given plant genome, with this demonstration of the utility of GO datasets for comparative functional genomics showing great promise for more sophisticated comparative methods as they are developed and applied.

It should be noted that such comparative analyses using gene functions are not absent from the literature - though they are absent for large genome comparisons in plants. An example of such comparative studies where a given tree topology is used to look for gains and loss of functions that are in agreement with known biology was reported by Schwacke et al. [57]. They analyzed

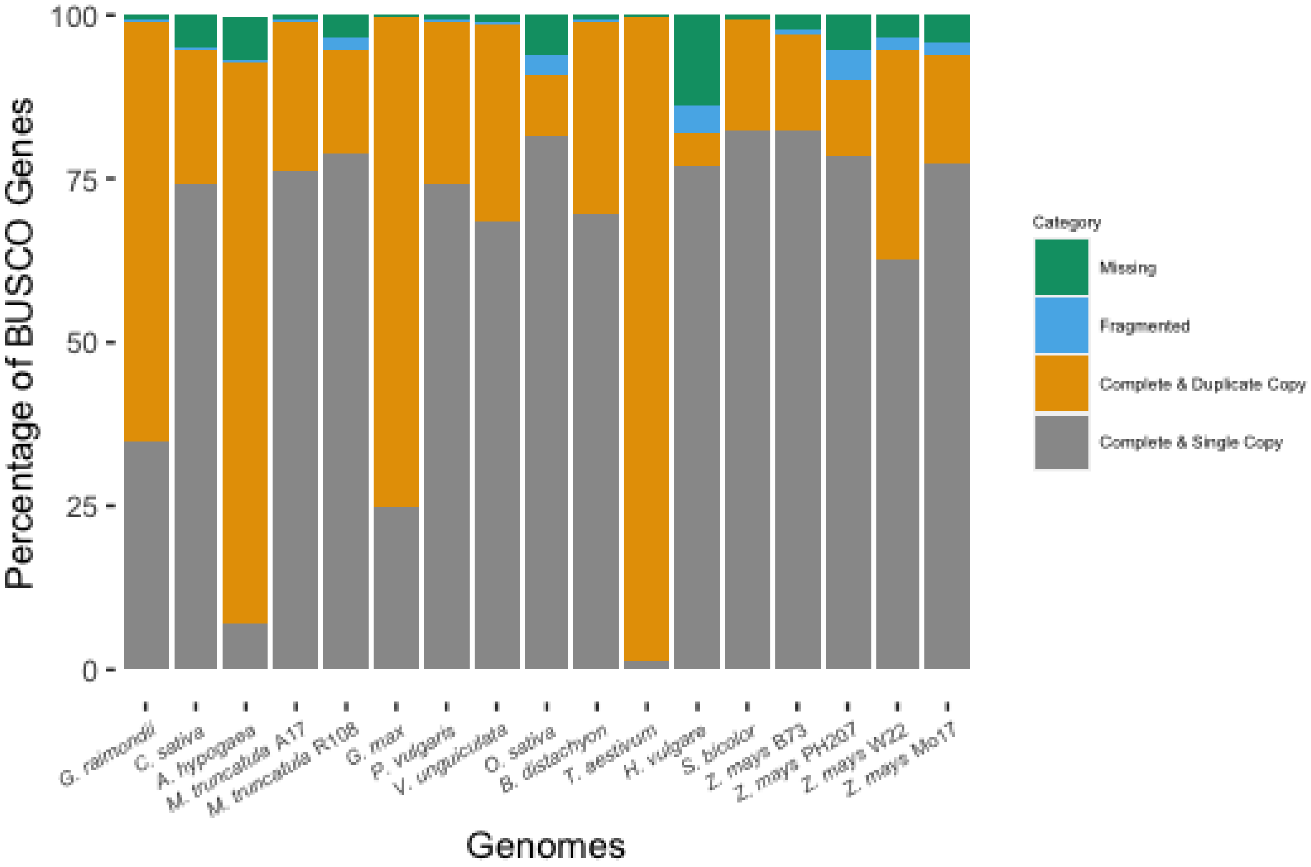

Figure 7: Annotation BUSCO plot generated using GenomeQC. Genomes analyzed are shown across the X-axis, and are ordered to match the occurrence of species shown in Figure 2. Percentage of BUSCO genes across four gene categories are stacked, with each adding up to 100 percent (Y-axis). Complete and single-copy genes are shown in grey, complete and duplicated copies in orange, fragmented copies in blue, and missing are shown copies in green.

gene loss in *Cuscuta*, a parasitic plant based on analysis using the Mapman ontology. In their work, they could demonstrate considerable loss of genes, which is a hallmark of the parasitic lifestyle. Our efforts differ in that we used the functions directly to infer tree structures as a demonstration that sufficient biological signal is present in GO-based datasets of genome-wide function prediction to reproduce known biological relationships. The method we used was quick and dirty, and we anticipate that refinements in approach that consider multiple copies of genes, as well as using different types of graph and network representations beyond tree structures, are logical next steps for refining the use of GO terms for comparative functional genomics analyses in plants. With that in mind, we look forward not only to developing systems to support comparative functional genomics tools, but also to seeing the tools other research groups will develop to approach these datasets for use to formulate novel comparative functional genomics hypotheses.

IV. METHODS

A. Acquiring Input Datasets

For each of the 18 genomes listed in Table I, information on how to access input annotation products are listed by DOI. For each, one representative translated peptide sequence per protein coding gene was selected and used as the input for GOMAP. Unless the authors of the genome provided a set of representative sequences designated as canonical, we chose the longest translated peptide sequence as the representative for each gene model. Non-IUPAC characters and trailing asterisks (*) were removed from the sequences, and headers were simplified to contain only non-special characters. The corresponding script for each dataset can be found at the respective DOI. Based on this input, GOMAP yielded a functional annotation set spanning all protein-coding genes in the genome. Using the Gene Ontology version releases/2020-10-09, this functional annotation set was cleaned up by removing duplicates, annotations with modifiers, and ob-

solete GO terms. Any terms containing alternative identifiers were merged to their respective main identifier, uncovering a few additional duplicates, which were also removed. Table SII shows the number of annotations removed from each dataset produced.

To compare the quality of GOMAP predictions to currently available functional predictions from Gramene and Phytozome, we downloaded IEA annotations from Gramene (version 63, [32], https://www.gramene.org/) and Phytozome (version 12, [33], https://phytozome.jgi.doe.gov/) for each species with functional annotations of the same genome version. These datasets were cleaned as above. Similarly cleaned non-IEA annotations from Gramene served as the Gold Standard wherever they were available. More detailed information on how these datasets were accessed can be found at https://github.com/Dill-PICL/GOMAP-Paper-2019.1/blob/master/data/go_annotation_sets/README.md.

B. Quantitative and Qualitative Evaluation

The number of annotations in each clean dataset was determined and related to the number of protein coding genes (based on transcripts in the input FASTA file). This was done for separately for each GO aspect as well as in total.

The ADS software version published in [41] is available from https://bitbucket.org/plyusnin/ads/. We used version b6309cb (also included in our code as a submodule) to calculate SimGIC2, TC-AUCPCR, and F_{max} quality scores. To provide the information content required for the SimGIC2 metric, the Arabidopsis GOA from https://www.ebi.ac.uk/GOA/arabidopsis_release was used in version 2021-02-16.

C. Cladogram Construction

For clustering, we first collected all GO terms annotated to any gene in each genome into a list and removed the duplicates, yielding a one-dimensional set of GO terms for each genome (T). Next, we added all ancestor terms for each term in this set T and once again removed the duplicates, yielding a set S. These sets with added ancestors served as a starting point of our tree-building analyses: pairwise distances between the genomes were calculated using the Jaccard distance as a metric of the dissimilarity between any two sets a and b.

$$d_{ab} = 1 - \frac{|S_a \cap S_b|}{|S_a \cup S_b|} \qquad (1)$$

A neighbor-joining tree was constructed based on the generated pairwise distance matrix using PHYLIP [34]. Additionally, term sets S of all genomes were combined into a binary matrix (with rows corresponding to genomes and columns corresponding to GO terms, values of 0 or 1 indicating whether a term is present or absent in the given set). PHYLIP pars was used to construct a parsimony tree from this binary matrix.

P. lambertiana, a gymnosperm, was included in the dataset as an outgroup to the angiosperms to separate between the monocot and eudicot clades. iTOL [58] was used to visualize the trees using their Newick format, and root them at *P. lambertiana*. Moreover, a cladogram representing the known phylogeny of the included taxa was created by hand based on known evolutionary relationships [59, 60, 61, 62, 63]. This was used to compare the generated phylogenetic relationship based on functional similarity with the evolutionary relationships of the plant genomes.

Jackknifing analysis was carried out for both parsimony and neighbor-joining trees to assess the support for each clade based on the proportion of jackknife trees showing the same clade. To this end, 40% of the terms in T were randomly removed, ancestors of the remaining terms were added and trees constructed as above. The majority rule consensus tree of 100 individual trees was calculated with the jackknife values represented on each branch. The tree was then visualized using iTOL using its Newick format, and rooted again at *P. lambertiana*.

D. Genome Quality Evaluation

Genome size was estimated from the C-values obtained from the Plant DNA C-values data resource from the Kew Database (https://cvalues.science.kew.org). The mean C-value for a given species was used for calculating genome size estimates in base pairs (bp) using the method of [64]. In brief,

$$\text{Genome size (bp)} = \text{C-value (pg)} * 0.978\text{x}10^9 \frac{\text{bp}}{\text{pg}}$$

The estimated genome size (listed in Table IV) was used as an input for GenomeQC (https://genomeqc.maizegdb.org/) [43] to calculate quality metrics. For genomes that were too large to submit through the GenomeQC webtool or had missing exon information, modified scripts of those found in GitHub of GenomeQC (https://github.com/HuffordLab/GenomeQC, commit e6140ee) were applied to calculate the assembly and structural annotation metrics in Table IV and V. BUSCO version 5.0.0 [65] was used to calculate the assembly and annotation BUSCO scores, shown in Figures 6 and 7. The input for assembly BUSCO scores were chromosome sequences, whereas inputs were transcript/mRNA/CDS sequences for the annotation BUSCO scores. For the lineage parameter, the lineage datasets used were as follows: Eudicots for *C. sativa* and *G. raimondii*, Fabales for *A. hypogaea*, *M. truncatula* A17 and R108, *P. vulgaris*, *G. max*, and *V. unguiculata*, and Poales for *B. distachyon*, *O. sativa*, *T. aestivum*, *H.*

vulgare, *S. bicolor* and *Z. mays* B73, Mo17, W22 and PH207.

V. AVAILABILITY OF SOURCE CODE AND SUPPORTING DATA

All data and source code generated are freely available at https://github.com/Dill-PICL/GOMAP-Paper-2019.1 under the terms of the MIT license. All software requirements and dependencies are packaged into a Singularity container so no other setup is required to reproduce our results. We will provide a DOI through Zenodo for the final version of the manuscript after reviews and corrections are incorporated.

An up-to-date list of all available annotation sets can be found at https://dill-picl.org/projects/gomap/gomap-datasets/.

VI. DECLARATIONS

A. Competing Interests

The author(s) declare that they have no competing interests.

B. Funding

This work has been supported by the Iowa State University Plant Sciences Institute Faculty Scholars Program to CJLD, the Predictive Plant Phenomics NSF Research Traineeship (#DGE-1545453) to CJLD (CFY is a trainee), and IOW04714 Hatch funding to Iowa State University.

C. Author's Contributions

LF, DP, DFY, KC, HAD, PJ, DCS, HV, and KW generated annotations for plants as described in this paper. DP and CJLD co-conceived the idea for phylogenetic analysis. LF worked with DP to create dendrograms and compare those to phylogenetic trees. LF carried out assembly and annotation metric comparisons. LF, DP, and CJLD wrote the manuscript. All authors read, offered suggestions to improve, and approved the final copy of the manuscript.

VII. ACKNOWLEDGEMENTS

Thanks to Steven Cannon for help to understand phylogenetic relationships among eudicots and helpful discussions and to Toby Kellogg and Jedrzej Szymanski for discussions and ideas on how to consider the data. Thanks to Nancy Manchanda for reviewing documentation and for checking genome versions used as input for GenomeQC. Thanks to Darwin Campbell who guided the deposition of datasets with CyVerse for release and DOI assignment.

VIII. AUTHORS' INFORMATION

KW created the GOMAP system during his time as a graduate student at Iowa State University. LF, DP, CFY, KC, HV, and PJ are currently graduate students. HAD and DCS are undergraduate students. Each graduate and undergraduate student annotated at least one genome over the course of a research rotation lasting no more than one semester. CJLD coordinated research activities and manuscript preparation.

References

[1] Minoru Kanehisa et al. "KEGG: integrating viruses and cellular organisms". In: *Nucleic Acids Research* 49.D1 (Oct. 2020), pp. D545–D551. DOI: 10.1093/nar/gkaa970. URL: https://doi.org/10.1093/nar/gkaa970.

[2] Michael Ashburner et al. "Gene Ontology: tool for the unification of biology". In: *Nature genetics* 25.1 (May 2000), pp. 25–29. ISSN: 1061-4036. DOI: 10.1038/75556. URL: https://www.ncbi.nlm.nih.gov/pmc/articles/PMC3037419/ (visited on 03/12/2021).

[3] The Gene Ontology Consortium et al. "The Gene Ontology resource: enriching a GOld mine". en. In: *Nucleic Acids Research* 49.D1 (Jan. 2021), pp. D325–D334. ISSN: 0305-1048, 1362-4962. DOI: 10.1093/nar/gkaa1113. URL: https://academic.oup.com/nar/article/49/D1/D325/6027811 (visited on 03/12/2021).

[4] Oliver Thimm et al. "mapman: a user-driven tool to display genomics data sets onto diagrams of metabolic pathways and other biological processes". In: *The Plant Journal* 37.6 (Mar. 2004), pp. 914–939. DOI: 10.1111/j.1365-313x.2004.02016.x. URL: https://doi.org/10.1111/j.1365-313x.2004.02016.x.

[5] D. Binns et al. "QuickGO: a web-based tool for Gene Ontology searching". In: *Bioinformatics* 25.22 (Sept. 2009), pp. 3045–3046. DOI: 10.1093/bioinformatics/btp536. URL: https://doi.org/10.1093/bioinformatics/btp536.

[6] Iris Tzafrir et al. "Identification of Genes Required for Embryo Development in Arabidopsis". en. In: *Plant Physiology* 135.3 (July 2004). Publisher: American Society of Plant Biologists Section: GENOME ANALYSIS, pp. 1206–1220. ISSN: 0032-0889, 1532-2548. DOI: 10.1104/pp.104.045179. URL: http://www.plantphysiol.org/content/135/3/1206 (visited on 03/12/2021).

[7] Ana Conesa and Stefan Götz. "Blast2GO: A Comprehensive Suite for Functional Analysis in Plant Genomics". en. In: *International Journal of Plant Genomics* 2008 (Apr. 2008), pp. 1–12. ISSN: 1687-5370, 1687-5389. DOI: 10.1155/2008/619832. URL: https://www.hindawi.com/journals/ijpg/2008/619832/ (visited on 03/12/2021).

[8] Seung Yon Rhee and Marek Mutwil. "Towards revealing the functions of all genes in plants". In: *Trends in Plant Science* 19.4 (Apr. 2014), pp. 212–221. ISSN: 1360-1385. DOI: 10.1016/j.tplants.2013.10.006. URL: https://www.sciencedirect.com/science/article/pii/S1360138513002343 (visited on 03/12/2021).

[9] Kokulapalan Wimalanathan and Carolyn J. Lawrence-Dill. "Gene Ontology Meta Annotator for Plants (GOMAP)". In: *Plant Methods* 17.1 (May 2021). DOI: 10.1186/s13007-021-00754-1. URL: https://doi.org/10.1186/s13007-021-00754-1.

[10] Naihui Zhou et al. "The CAFA challenge reports improved protein function prediction and new functional annotations for hundreds of genes through experimental screens". In: *Genome Biology* 20.1 (Nov. 2019). DOI: 10.1186/s13059-019-1835-8. URL: https://doi.org/10.1186/s13059-019-1835-8.

[11] URL: https://www.biofunctionprediction.org.

[12] Kokulapalan Wimalanathan et al. "Maize GO Annotation-Methods, Evaluation, and Review (maize-GAMER)". In: *Plant Direct* 2.4 (Apr. 2018), e00052. ISSN: 24754455. DOI: 10.1002/pld3.52. URL: http://doi.wiley.com/10.1002/pld3.52.

[13] Chengsheng Zhu et al. "Functional Basis of Microorganism Classification". en. In: *PLOS Computational Biology* 11.8 (Aug. 2015). Publisher: Public Library of Science, e1004472. ISSN: 1553-7358. DOI: 10.1371/journal.pcbi.1004472. URL: https://journals.plos.org/ploscompbiol/article?id=10.1371/journal.pcbi.1004472 (visited on 03/12/2021).

[14] Dennis Psaroudakis and Carolyn Lawrence-Dill. *GOMAP Peanut IPGI 1.0*. 2019. DOI: 10.25739/CHAB-0E35. URL: http://datacommons.cyverse.org/browse/iplant/home/shared/commons_repo/curated/Carolyn_Lawrence_Dill_GOMAP_Peanut_Tifrunner.IPGI.1.0_August_2019.r1.

[15] Kokulapalan Wimalanathan and Carolyn Lawrence-Dill. *GOMAP Bdistachyon.Bd21.v3.1.r1*. 2019. DOI: 10.25739/DW2T-3G82. URL: http://datacommons.cyverse.org/browse/iplant/home/shared/commons_repo/curated/Carolyn_Lawrence_Dill_GOMAP_Bdistachyon.Bd21.v3.1_November_2019.r1.

[16] Kevin Chiteri and Carolyn Lawrence-Dill. *GOMAP_Cannabis_sativa_NCBI-cs10_January_2020*. 2020. DOI: 10.25739/AB9Z-2Z86. URL: http://datacommons.cyverse.org/browse/iplant/home/shared/commons_repo/curated/Carolyn_Lawrence_Dill_GOMAP_Cannabis_NCBI-cs10_January_2020.r1.

[17] Dennis Psaroudakis and Carolyn Lawrence-Dill. *GOMAP Soybean JGI-Wm82.a4.v1*. 2019. DOI: 10.25739/59EC-1719. URL: http://datacommons.cyverse.org/browse/iplant/home/shared/commons_repo/curated/Carolyn_Lawrence_Dill_GOMAP_Soybean_JGI-Wm82.a4.v1_April_2019.r1.

[18] Parnal Joshi and Carolyn Lawrence-Dill. *GOMAP Gossypium raimondii JGI v2.1*. 2020. DOI: 10.25739/A13T-ZH47. URL: http://datacommons.cyverse.org/browse/iplant/home/shared/commons_repo/curated/Carolyn_Lawrence_Dill_GOMAP_Gossypium_raimondii_JGI_v2.1_January_2020.r1.

[19] Colleen Yanarella and Carolyn Lawrence-Dill. *GOMAP Barley Reference Sequences IBSC_PGSB_r1*. 2019. DOI: 10.25739/ZVGV-8E37. URL: http://datacommons.cyverse.org/browse/iplant/home/shared/commons_repo/curated/Carolyn_Lawrence_Dill_GOMAP_Barley_IBSC_PGSB-1.0_May_2019.r1.

[20] Dennis Psaroudakis and Carolyn Lawrence-Dill. *GOMAP Barrel Clover R108_HM340 v1.0*. 2019. DOI: 10.25739/2SQC-J140. URL: http://datacommons.cyverse.org/browse/iplant/home/shared/commons_repo/curated/Carolyn_Lawrence_Dill_GOMAP_BarrelClover_R108_HM340_v1.0_August_2019.r1.

[21] Dennis Psaroudakis and Carolyn Lawrence-Dill. *GOMAP Barrel Clover A17_HM341 Mt4.0v2*. 2019. DOI: 10.25739/PY38-YB08. URL: http://datacommons.cyverse.org/browse/iplant/home/shared/commons_repo/curated/Carolyn_Lawrence_Dill_GOMAP_BarrelClover_A17_HM341_Mt4.0v2_August_2019.r1.

[22] Ha Vu and Carolyn Lawrence-Dill. *GOMAP Rice Reference Sequences 2.0*. 2019. DOI: 10.25739/53G0-J859. URL: http://datacommons.cyverse.org/browse/iplant/home/shared/commons_repo/curated/Carolyn_Lawrence_Dill_GOMAP_Rice_IRGSP-1.0_April_2019.r2.

[23] Dennis Psaroudakis and Carolyn Lawrence-Dill. *GOMAP Common Bean DOE-JGI and USDA-NIFA v2.0*. 2019. DOI: 10.25739/1YWE-EW96. URL: http://datacommons.cyverse.org/browse/iplant/home/shared/commons_repo/curated/Carolyn_Lawrence_Dill_GOMAP_CommonBean_DOE-JGI-USDA-NIFA.2.0_August_2019.r1.

[24] Colleen Yanarella and Carolyn Lawrence-Dill. *GOMAP TreeGenesDB sugar pine assembly v1.5*. 2020. DOI: 10.25739/JVS4-XR88. URL: http://datacommons.cyverse.org/browse/iplant/home/shared/commons_repo/curated/Carolyn_Lawrence_Dill_GOMAP_SugarPine_TreeGenesDB-1.5_January_2020.r1.

[25] Kokulapalan Wimalanathan and Carolyn Lawrence-Dill. *GOMAP Sbicolor.BTx623.v3.0.1.r1*. 2019. DOI: 10.25739/4TY0-YE98. URL: http://datacommons.cyverse.org/browse/iplant/home/shared/commons_repo/curated/Carolyn_Lawrence_Dill_GOMAP_Sbicolor.BTx623.v3.0.1_November_2019.r1.

[26] Dennis Psaroudakis and Carolyn Lawrence-Dill. *GOMAP Wheat Reference Sequences 1.1*. 2019. DOI: 10.25739/65KF-JZ20. URL: http://datacommons.cyverse.org/browse/iplant/home/shared/commons_repo/curated/Carolyn_Lawrence_Dill_GOMAP_Wheat_RefSeq1.1_HC_December_2018.r1.

[27] Dennis Psaroudakis and Carolyn Lawrence-Dill. *GOMAP Cowpea IT97K-499-35 JGI annotation v1.1*. 2019. DOI: 10.25739/CDX9-WR97. URL: http://datacommons.cyverse.org/browse/iplant/home/shared/commons_repo/curated/Carolyn_Lawrence_Dill_GOMAP_Cowpea_JGI.v1.1_August_2019.r1.

[28] Kokulapalan Wimalanathan and Carolyn Lawrence-Dill. *GOMAP Maize Zm-Mo17-REFERENCE-CAU-1.0 Zm00014a.1*. 2019. DOI: 10.25739/M634-CN58. URL: http://datacommons.cyverse.org/browse/iplant/home/shared/commons_repo/curated/Carolyn_Lawrence_Dill_GOMAP_maize.Mo17.AGPv1_April_2019.r1.

[29] Kokulapalan Wimalanathan and Carolyn Lawrence-Dill. *GOMAP Maize Zm-PH207-REFERENCE_NS-UIUC_UMN-1.0 Zm00008a.1*. 2019. DOI: 10.25739/DM9S-AA15. URL: http://datacommons.cyverse.org/browse/iplant/home/shared/commons_repo/curated/

Carolyn_Lawrence_Dill_GOMAP_maize.PH207.UIUC_UMN-1.0_April_2019.r1.

[30] Kokulapalan Wimalanathan and Carolyn Lawrence-Dill. *GOMAP Maize Zm-W22-REFERENCE-NRGENE-2.0 Zm00004b.1*. 2019. DOI: 10.25739/E4VA-9F09. URL: http://datacommons.cyverse.org/browse/iplant/home/shared/commons_repo/curated/Carolyn_Lawrence_Dill_GOMAP_maize.W22.AGPv2_April_2019.r1.

[31] Kokulapalan Wimalanathan and Carolyn Lawrence-Dill. *maize-GAMER Annotaions for maize.B73.AGPv4.r1*. 2017. DOI: 10.7946/P2M925. URL: http://datacommons.cyverse.org/browse/iplant/home/shared/commons_repo/curated/Carolyn_Lawrence-Dill_maize-GAMER_maize.B73_RefGen_v4_Zm00001d.2_Oct_2017.r1.

[32] Marcela K Tello-Ruiz et al. "Gramene 2021: harnessing the power of comparative genomics and pathways for plant research". In: *Nucleic Acids Research* 49.D1 (Nov. 2020), pp. D1452–D1463. DOI: 10.1093/nar/gkaa979. URL: https://doi.org/10.1093/nar/gkaa979.

[33] David M. Goodstein et al. "Phytozome: a comparative platform for green plant genomics". In: *Nucleic Acids Research* 40.D1 (Nov. 2011), pp. D1178–D1186. DOI: 10.1093/nar/gkr944. URL: https://doi.org/10.1093/nar/gkr944.

[34] Joseph Felsenstein. *PHYLIP (phylogeny inference package), version 3.5 c.* Joseph Felsenstein., 1993.

[35] Walter M. Fitch. "Toward Defining the Course of Evolution: Minimum Change for a Specific Tree Topology". In: *Systematic Zoology* 20.4 (Dec. 1971), p. 406. DOI: 10.2307/2412116. URL: https://doi.org/10.2307/2412116.

[36] N Saitou and M Nei. "The neighbor-joining method: a new method for reconstructing phylogenetic trees." In: *Molecular Biology and Evolution* (July 1987). DOI: 10.1093/oxfordjournals.molbev.a040454. URL: https://doi.org/10.1093/oxfordjournals.molbev.a040454.

[37] C. F. J. Wu. "Jackknife, Bootstrap and Other Resampling Methods in Regression Analysis". In: *The Annals of Statistics* 14.4 (Dec. 1986). DOI: 10.1214/aos/1176350142. URL: https://doi.org/10.1214/aos/1176350142.

[38] S. C. Potter. "The Ensembl Analysis Pipeline". In: *Genome Research* 14.5 (May 2004), pp. 934–941. DOI: 10.1101/gr.1859804. URL: https://doi.org/10.1101/gr.1859804.

[39] Karin Verspoor et al. "A categorization approach to automated ontological function annotation". In: *Protein Science* (2006). ISSN: 09618368. DOI: 10.1110/ps.062184006. URL: https://dx.doi.org/10.1110%7B%5C%%7D2Fps.062184006.

[40] Michael Defoin-Platel et al. "AIGO: Towards a unified framework for the Analysis and the Inter-comparison of GO functional annotations". In: *BMC Bioinformatics* (2011). ISSN: 14712105. DOI: 10.1186/1471-2105-12-431. URL: https://doi.org/10.1186/1471-2105-12-431.

[41] Ilya Plyusnin, Liisa Holm, and Petri Törönen. "Novel comparison of evaluation metrics for gene ontology classifiers reveals drastic performance differences". In: *PLOS Computational Biology* 15.11 (Nov. 2019). Ed. by Predrag Radivojac, e1007419. ISSN: 1553-7358. DOI: 10.1371/journal.pcbi.1007419. URL: http://dx.plos.org/10.1371/journal.pcbi.1007419.

[42] Ivica Letunic and Peer Bork. "Interactive Tree Of Life (iTOL) v5: an online tool for phylogenetic tree display and annotation". In: *Nucleic Acids Research* (Apr. 2021). DOI: 10.1093/nar/gkab301. URL: https://doi.org/10.1093/nar/gkab301.

[43] Nancy Manchanda et al. "GenomeQC: a quality assessment tool for genome assemblies and gene structure annotations". In: *BMC Genomics* 21.1 (Mar. 2020), p. 193. ISSN: 1471-2164. DOI: 10.1186/s12864-020-6568-2. URL: https://doi.org/10.1186/s12864-020-6568-2 (visited on 03/12/2021).

[44] Felipe A. Simão et al. "BUSCO: assessing genome assembly and annotation completeness with single-copy orthologs". en. In: *Bioinformatics* 31.19 (Oct. 2015), pp. 3210–3212. ISSN: 1367-4803, 1460-2059. DOI: 10.1093/bioinformatics/btv351. URL: https://academic.oup.com/bioinformatics/article-lookup/doi/10.1093/bioinformatics/btv351 (visited on 03/12/2021).

[45] Haley Dostalik and Carolyn Lawrence-Dill. *Carolyn_Lawrence_Dill_GOMAP_Grape_Genoscope_12x_January_202* 2021. DOI: 10.25739/JTFK-Q888. URL: http://datacommons.cyverse.org/browse/iplant/home/shared/commons_repo/curated/Carolyn_Lawrence_Dill_GOMAP_Grape_Genoscope_12x_January_2021.r1.

[46] Carolyn Lawrence-Dill. $Carolyn_{L}awrence_{D}ill_{G}OMAP_{C}anola_{B}nPIR_{Z}S11_{M}arch_{2}021.r$ 2021. DOI: 10.25739/XGMR-HR31. URL: http://datacommons.cyverse.org/browse/iplant/home/shared/commons_repo/curated/Carolyn_Lawrence_Dill_GOMAP_Canola_BnPIR_ZS11_March_2021.r1.

[47] Leila Fattel and Carolyn Lawrence-Dill. *Carolyn_Lawrence_Dill_GOMAP_Banana_NCBI_ASM31385v2_Febru* 2021. DOI: 10.25739/YT7W-GS55. URL: http://datacommons.cyverse.org/browse/iplant/home/shared/commons_repo/curated/Carolyn_Lawrence_Dill_GOMAP_Banana_NCBI_ASM31385v2_February_2021.r1.

[48] Leila Fattel and Carolyn Lawrence-Dill. *Carolyn_Lawrence_Dill_GOMAP_Cacao_NCBI_CriolloV2_March_202* 2021. DOI: 10.25739/9QC0-N310. URL: http://datacommons.cyverse.org/browse/iplant/home/shared/commons_repo/curated/Carolyn_Lawrence_Dill_GOMAP_Cacao_NCBI_CriolloV2_March_2021.r1.

[49] Carolyn Lawrence-Dill. $Carolyn_{L}awrence_{D}ill_{G}OMAP_{C}offee_{C}GH_{v}1.0_{J}une_{2}021.r1.$ 2021. DOI: 10.25739/RM4J-3580. URL: http://datacommons.cyverse.org/browse/iplant/home/shared/commons_repo/curated/Carolyn_Lawrence_Dill_GOMAP_Coffee_CGH_v1.0_June_2021.r1.

[50] Carolyn Lawrence-Dill. $Carolyn_{L}awrence_{D}ill_{G}OMAP_{B}lueberry_{G}igaDB_{v}1.0_{J}une_{2}021.r$ 2021. DOI: 10.25739/Q7RQ-E992. URL: http://datacommons.cyverse.org/browse/iplant/home/shared/commons_repo/curated/Carolyn_Lawrence_Dill_GOMAP_Blueberry_GigaDB_v1.0_June_2021.r1.

[51] Dennis Psaroudakis and Carolyn Lawrence-Dill. *Carolyn_Lawrence_Dill_GOMAP_Solanum_lycopersicum_ITAG4.1.v1* 2021. DOI: 10.25739/ZH2V-4P15. URL: http://datacommons.cyverse.org/browse/iplant/home/shared/commons_repo/curated/Carolyn_Lawrence_

Dill_GOMAP_Solanum_lycopersicum_ITAG4.1.v1_April_2021.r1.

[52] Dennis Psaroudakis and Carolyn Lawrence-Dill. *Carolyn_Lawrence_Dill_GOMAP_Solanum_pennellii_Bolger2014.v1_April_2021.r1*. 2021. DOI: 10.25739/FHR4-CX67. URL: http://datacommons.cyverse.org/browse/iplant/home/shared/commons_repo/curated/Carolyn_Lawrence_Dill_GOMAP_Solanum_pennellii_Bolger2014.v1_April_2021.r1.

[53] Koichi Sakamoto et al. "Characterization Genome Sizes and Morphology of Sex Chromosomes in Hemp (Cannabis sativa L.)." In: *CYTOLOGIA* 63.4 (1998), pp. 459–464. DOI: 10.1508/cytologia.63.459. URL: https://doi.org/10.1508/cytologia.63.459.

[54] Harm van Bakel et al. "The draft genome and transcriptome of Cannabis sativa". In: *Genome Biology* 12.10 (2011), R102. DOI: 10.1186/gb-2011-12-10-r102. URL: https://doi.org/10.1186/gb-2011-12-10-r102.

[55] Shan Gao et al. "A high-quality reference genome of wild Cannabis sativa". en. In: *Horticulture Research* 7.1 (May 2020). Number: 1 Publisher: Nature Publishing Group, pp. 1–11. ISSN: 2052-7276. DOI: 10.1038/s41438-020-0295-3. URL: https://www.nature.com/articles/s41438-020-0295-3 (visited on 03/12/2021).

[56] W.P. Maddison and D.R. Maddison. *Mesquite: a modular system for evolutionary analysis. Version 3.61*. 2021. URL: http://www.mesquiteproject.org/ (visited on 07/14/2021).

[57] Rainer Schwacke et al. "MapMan4: A Refined Protein Classification and Annotation Framework Applicable to Multi-Omics Data Analysis". In: *Molecular Plant* 12.6 (June 2019), pp. 879–892. DOI: 10.1016/j.molp.2019.01.003. URL: https://doi.org/10.1016/j.molp.2019.01.003.

[58] Ivica Letunic and Peer Bork. "Interactive Tree Of Life (iTOL) v4: recent updates and new developments". en. In: *Nucleic Acids Research* 47.W1 (July 2019), W256–W259. ISSN: 0305-1048, 1362-4962. DOI: 10.1093/nar/gkz239. URL: https://academic.oup.com/nar/article/47/W1/W256/5424068 (visited on 03/12/2021).

[59] Elizabeth A. Kellogg. "Evolutionary History of the Grasses". en. In: *Plant Physiology* 125.3 (Mar. 2001), pp. 1198–1205. ISSN: 0032-0889, 1532-2548. DOI: 10.1104/pp.125.3.1198. URL: https://academic.oup.com/plphys/article/125/3/1198-1205/6109905 (visited on 03/12/2021).

[60] Steven B. Cannon and Randy C. Shoemaker. "Evolutionary and comparative analyses of the soybean genome". eng. In: *Breeding Science* 61.5 (Jan. 2012), pp. 437–444. ISSN: 1344-7610. DOI: 10.1270/jsbbs.61.437.

[61] Candice N. Hansey et al. "Maize (Zea mays L.) genome diversity as revealed by RNA-sequencing". eng. In: *PloS One* 7.3 (2012), e33071. ISSN: 1932-6203. DOI: 10.1371/journal.pone.0033071.

[62] Nasim Azani et al. "A new subfamily classification of the Leguminosae based on a taxonomically comprehensive phylogeny: The Legume Phylogeny Working Group (LPWG)". en. In: *TAXON* 66.1 (2017). _eprint: https://onlinelibrary.wiley.com/doi/pdf/10.12705/661.3, pp. 44–77. ISSN: 1996-8175. DOI: https://doi.org/10.12705/661.3. URL: https://onlinelibrary.wiley.com/doi/abs/10.12705/661.3 (visited on 03/12/2021).

[63] Mark N. Puttick et al. "The Interrelationships of Land Plants and the Nature of the Ancestral Embryophyte". English. In: *Current Biology* 28.5 (Mar. 2018). Publisher: Elsevier, 733–745.e2. ISSN: 0960-9822. DOI: 10.1016/j.cub.2018.01.063. URL: https://www.cell.com/current-biology/abstract/S0960-9822(18)30096-4 (visited on 03/12/2021).

[64] J. Doležel et al. "Letter to the editor". In: *Cytometry* 51A.2 (Jan. 2003), pp. 127–128. DOI: 10.1002/cyto.a.10013. URL: https://doi.org/10.1002/cyto.a.10013.

[65] Mathieu Seppey, Mosè Manni, and Evgeny M. Zdobnov. "BUSCO: Assessing Genome Assembly and Annotation Completeness". In: *Methods in Molecular Biology*. Springer New York, 2019, pp. 227–245. DOI: 10.1007/978-1-4939-9173-0_14. URL: https://doi.org/10.1007/978-1-4939-9173-0_14.

[66] Eva M Temsch and Johann Greilhuber. "Genome size variation in Arachis hypogaea and A. monticola re-evaluated". In: *Genome* 43.3 (June 2000), pp. 449–451. DOI: 10.1139/g99-130. URL: https://doi.org/10.1139/g99-130.

[67] Pilar Catalán et al. "Evolution and taxonomic split of the model grass Brachypodium distachyon". In: *Annals of Botany* 109.2 (Jan. 2012), pp. 385–405. DOI: 10.1093/aob/mcr294. URL: https://doi.org/10.1093/aob/mcr294.

[68] Johann Greilhuber and Renate Obermayer. "Genome size and maturity group in Glycine max (soybean)". In: *Heredity* 78.5 (1997), pp. 547–551.

[69] B. HENDRIX. "Estimation of the Nuclear DNA Content of Gossypium Species". In: *Annals of Botany* 95.5 (Feb. 2005), pp. 789–797. DOI: 10.1093/aob/mci078. URL: https://doi.org/10.1093/aob/mci078.

[70] Michael David Bennett and JB Smith. "Nuclear DNA amounts in angiosperms". In: *Philosophical Transactions: Biological Sciences* (1991), pp. 309–345.

[71] Fatima Pustahija et al. "Small genomes dominate in plants growing on serpentine soils in West Balkans, an exhaustive study of 8 habitats covering 308 taxa". In: *Plant and soil* 373.1 (2013), pp. 427–453.

[72] Ka Arumuganathan and ED Earle. "Nuclear DNA content of some important plant species". In: *Plant molecular biology reporter* 9.3 (1991), pp. 208–218.

[73] AY Kenton, SJ Owens, and D Langton. "The origin of ringformation and self-compatibility in Gibasis pulchella (Commelinaceae)". In: *Kew Chromosome Conference*. Vol. 3. 1988, pp. 75–84.

[74] DA Laurie and MD Bennett. "Nuclear DNA content in the genera Zea and Sorghum. Intergeneric, interspecific and intraspecific variation". In: *Heredity* 55.3 (1985), pp. 307–313.

[75] A Parida, SN Raina, and RKJ Narayan. "Quantitative DNA variation between and within chromosome complements of Vigna species (Fabaceae)". In: *Genetica* 82.2 (1990), pp. 125–133.

Table II: Quantitative metrics of the cleaned functional annotation sets. CC, MF, BP, and A refer to the aspects of the Gene Ontology: Cellular Component, Molecular Function, Biological Process, and Any/All. GOMAP covers all genomes with at least one annotation per gene and provides substantially more annotations than Gramene63 or Phytozome, especially in the BP aspect.

			Genes Annotated[%][a]				Annotations[b]				Median Ann. per G.[c]			
Genome	Genes	Dataset	CC	MF	BP	**A**	CC	MF	BP	**A**	CC	MF	BP	**A**
Arachis hypogaea	67,124	GOMAP	85.85	84.68	100.00	**100.00**	150,525	132,144	493,145	**775,814**	2	2	6	**10**
Brachypodium distachyon	34,310	GOMAP	81.33	85.35	100.00	**100.00**	74,172	69,213	255,397	**398,782**	2	2	6	**10**
		GoldStandard	21.54	19.53	18.20	**26.66**	10,985	10,436	11,120	**32,673**	1	1	1	**3**
		Gramene63-IEA	33.12	49.29	38.29	**63.60**	21,658	36,372	23,899	**82,026**	1	1	1	**3**
		Phytozome12	10.25	37.21	26.86	**43.11**	4,186	18,597	11,070	**34,060**	0	1	1	**2**
Cannabis sativa	33,677	GOMAP	94.22	95.48	100.00	**100.00**	85,755	73,614	262,741	**422,110**	2	2	6	**11**
Glycine max	52,872	GOMAP	86.95	88.92	100.00	**100.00**	126,470	113,068	416,989	**656,527**	2	2	6	**11**
Gossypium raimondii	37,505	GOMAP	93.00	92.37	100.00	**100.00**	95,419	84,910	307,470	**487,799**	2	2	6	**11**
Hordeum vulgare	39,734	GOMAP	88.57	91.76	100.00	**100.00**	86,489	79,727	272,420	**438,636**	2	2	5	**10**
		GoldStandard	28.23	26.30	23.43	**35.64**	15,734	15,391	15,267	**46,414**	1	1	1	**3**
		Gramene63-IEA	36.19	50.90	41.71	**65.03**	29,826	44,789	29,425	**104,178**	1	1	1	**3**
Medicago truncatula A17	50,444	GOMAP	83.79	86.69	100.00	**100.00**	104,902	99,155	363,608	**567,665**	2	2	6	**10**
		GoldStandard	25.45	23.26	21.51	**32.12**	17,938	18,416	18,461	**54,827**	1	1	1	**3**
		Gramene63-IEA	34.25	50.84	40.26	**66.14**	32,753	63,470	40,441	**137,001**	1	1	1	**3**
		Phytozome12	8.87	36.05	25.83	**41.07**	5,315	25,950	15,576	**47,098**	0	1	1	**2**
Medicago truncatula R108	55,706	GOMAP	72.10	90.14	100.00	**100.00**	108,388	107,499	381,831	**597,718**	1	2	5	**9**
Oryza sativa	35,825	GOMAP	79.78	83.31	100.00	**100.00**	71,306	64,150	248,304	**383,760**	2	2	6	**9**
		GoldStandard	29.95	27.29	25.33	**37.57**	15,492	15,176	16,536	**47,339**	1	1	1	**3**
		Gramene63-IEA	32.21	45.83	36.75	**60.13**	21,935	37,425	24,255	**83,645**	1	1	1	**3**
		Phytozome12	10.31	40.10	29.18	**46.09**	4,361	20,842	12,451	**37,884**	0	1	1	**2**
Phaseolus vulgaris	27,433	GOMAP	94.48	93.06	100.00	**100.00**	70,987	64,022	229,230	**364,239**	2	2	6	**11**
Pinus lambertiana	31,007	GOMAP	92.67	95.91	100.00	**100.00**	71,247	68,315	212,248	**351,810**	2	2	5	**10**
Sorghum bicolor	34,129	GOMAP	82.44	85.98	100.00	**100.00**	75,145	69,659	259,004	**403,808**	2	2	6	**10**
		GoldStandard	34.48	32.91	30.90	**42.84**	16,837	17,614	17,850	**52,593**	1	1	1	**3**
		Gramene63-IEA	35.91	52.11	42.36	**67.41**	23,608	39,418	27,074	**90,313**	1	1	1	**3**
		Phytozome12	10.54	39.19	27.90	**45.10**	4,246	19,724	11,432	**35,599**	0	1	1	**2**
Triticum aestivum	107,891	GOMAP	88.53	90.98	100.00	**100.00**	259,318	217,467	785,051	**1,261,836**	2	2	6	**10**
		GoldStandard	2.98	2.78	2.56	**3.82**	4,727	4,512	4,793	**14,035**	1	1	1	**3**
		Gramene63-IEA	29.12	58.62	38.72	**70.41**	47,595	111,889	62,977	**222,721**	0	1	1	**2**
Vigna unguiculata	29,773	GOMAP	91.21	91.08	100.00	**100.00**	74,791	67,734	242,847	**385,372**	2	2	6	**11**
		Phytozome12	13.91	45.68	34.14	**53.06**	5,107	19,962	12,209	**37,534**	0	1	1	**2**
Zea mays B73.v4	39,324	GOMAP	93.16	94.92	100.00	**100.00**	87,648	81,665	278,305	**447,618**	2	2	6	**10**
		GoldStandard	37.92	34.78	32.67	**46.85**	22,531	21,292	23,153	**67,285**	1	1	1	**3**
		Gramene63-IEA	39.16	58.16	48.21	**73.87**	30,189	53,748	35,276	**119,273**	1	1	1	**3**
Zea mays Mo17	38,620	GOMAP	86.98	90.87	100.00	**100.00**	86,074	78,650	277,395	**442,119**	2	2	6	**10**
		GoldStandard	27.56	25.20	23.73	**33.98**	16,128	15,384	16,489	**48,220**	1	1	1	**3**
Zea mays PH207	40,557	GOMAP	86.55	90.61	100.00	**100.00**	88,962	84,910	288,208	**462,080**	2	2	6	**10**
		GoldStandard	28.18	25.82	24.26	**34.66**	17,370	16,580	17,791	**51,984**	1	1	1	**3**
Zea mays W22	40,690	GOMAP	90.77	92.58	100.00	**100.00**	93,622	84,450	289,364	**467,436**	2	2	6	**10**
		GoldStandard	25.40	23.15	21.80	**31.29**	15,518	14,818	15,850	**46,402**	1	1	1	**3**

Download this table (CSV)

[a] How many genes in the genome have at least one GO term from the CC, MF, BP aspect annotated to them? A = How many at least one from any aspect? ($A = CC \cup MF \cup BP$)

[b] How many annotations in the CC, MF, and BP aspect does this dataset contain? A = How many in total? $A = CC + MF + BP$

[c] Take a typical gene that is present in the annotation set. How many annotations does it have in each aspect? A = How many in total? Please note that $A \neq CC + MF + BP$

Table III: Qualitative metrics of functional annotation sets predicted by GOMAP, Gramene, and Phytozome.

		SimGIC2			TC-AUCPCR			Fmax		
Genome	Dataset	CC	MF	BP	CC	MF	BP	CC	MF	BP
Brachypodium distachyon	GOMAP	0.404149	0.464127	0.223830	0.233442	0.230701	0.118526	0.741361	0.740897	0.526881
	Gramene63-IEA	0.317801	0.420859	0.349406	0.129163	0.192507	0.111361	0.691016	0.738542	0.650325
	Phytozome12	0.370264	0.370521	0.352206	0.112582	0.136832	0.085628	0.717759	0.697076	0.660603
Hordeum vulgare	GOMAP	0.400087	0.470012	0.238177	0.237231	0.261399	0.130784	0.745272	0.750213	0.560096
	Gramene63-IEA	0.306119	0.426601	0.381010	0.157352	0.228797	0.136002	0.680996	0.742638	0.665696
Medicago truncatula A17	GOMAP	0.371795	0.451258	0.213407	0.272809	0.282650	0.139032	0.730838	0.726991	0.531406
	Gramene63-IEA	0.329600	0.437274	0.343561	0.176497	0.265887	0.133503	0.701093	0.749900	0.654297
	Phytozome12	0.358311	0.367257	0.363013	0.144247	0.170863	0.110386	0.717307	0.698429	0.661233
Oryza sativa	GOMAP	0.408945	0.482650	0.248207	0.298502	0.303384	0.159724	0.751121	0.757181	0.559221
	Gramene63-IEA	0.328761	0.423191	0.341193	0.167619	0.265410	0.135451	0.711309	0.738732	0.643827
	Phytozome12	0.049975	0.041007	0.044279	0.000003	0.000003	0.000002	0.470134	0.266628	0.239256
Sorghum bicolor	GOMAP	0.404852	0.466708	0.224011	0.316873	0.337380	0.169883	0.746540	0.742001	0.534258
	Gramene63-IEA	0.323037	0.400241	0.353135	0.177038	0.260198	0.154157	0.711107	0.712170	0.653591
	Phytozome12	0.356091	0.348264	0.340124	0.151947	0.177579	0.110483	0.715714	0.675147	0.641535
Triticum aestivum	GOMAP	0.410582	0.489881	0.229271	0.050762	0.030610	0.019360	0.736476	0.762420	0.533897
	Gramene63-IEA	0.362452	0.476685	0.395112	0.040992	0.043701	0.027872	0.737769	0.762059	0.670953
Zea mays B73.v4	GOMAP	0.417455	0.467339	0.245373	0.302761	0.290371	0.153011	0.759504	0.746870	0.564707
	Gramene63-IEA	0.303231	0.416301	0.346308	0.175735	0.250075	0.138275	0.662987	0.732860	0.647725
Zea mays Mo17	GOMAP	0.399521	0.464265	0.225632	0.236209	0.239598	0.125599	0.744360	0.743026	0.537489
Zea mays PH207	GOMAP	0.394481	0.436266	0.224226	0.221709	0.221266	0.117086	0.743111	0.718933	0.533092
Zea mays W22	GOMAP	0.397602	0.463499	0.223511	0.210198	0.217609	0.113262	0.743783	0.742341	0.535572

Download this table (CSV)

Table IV: Assembly statistics*

	C-value (pg)	C-value Ref.	Estimated Genome Size (bp)	Scaffolds	Total Scaffold Length (bp)	Total Scaffold Length / Estimated Genome Size (%)	Scaffold Sequences >=25K nt	Scaffold Sequences >=25K nt / Estimated Genome Size (%)	%N
A. hypogaea	2.87	[66]	2806	384	2.56E+09	91.12	2.55E+09	91.01	0.15
B. distachyon	0.32	[67]	313	11	2.72E+08	87.00	2.72E+08	87.00	0.19
C. sativa	0.84	[53]	821	221	8.76E+08	106.72	8.75E+08	106.62	15.93
G. max	1.13	[68]	1105	282	9.78E+08	88.62	9.77E+08	88.48	2.65
G. raimondii	0.9	[69]	880	1033	7.61E+08	86.52	7.55E+08	85.77	1.75
H. vulgare	7.33	[70, 71]	7167	8	4.83E+09	67.45	4.83E+09	67.45	5.44
M. truncatula A17	0.47	[72]	460	2186	4.13E+08	89.93	4.03E+08	87.77	5.53
M. truncatula R108	0.47	[72]	460	909	4.02E+08	87.60	3.95E+08	86.09	0.68
O. sativa	0.5	[70]	489	63	3.75E+08	76.85	3.75E+08	76.74	0.03
P. vulgaris	0.6	[73]	587	478	5.37E+08	91.68	5.34E+08	91.12	1.05
S. bicolor	1.2	[74]	1173	870	7.09E+08	60.43	7.05E+08	60.08	4.72
T. aestivum	17.3	[70]	16916	22	1.45E+10	86.00	1.45E+10	86.00	1.90
V. unguiculata	0.6	[75]	587	686	5.19E+08	88.64	5.18E+08	88.38	0.00
Z. mays B73	2.7	[70]	2640	266	2.13E+09	80.85	2.13E+09	80.85	1.44
Z. mays Mo17	2.7	[70]	2640	2208	2.18E+09	82.68	2.17E+09	82.06	1.61
Z. mays PH207	2.7	[70]	2640	43291	2.16E+09	81.67	2.09E+09	79.25	19.61
Z. mays W22	2.7	[70]	2640	11	2.13E+09	80.83	2.13E+09	80.83	1.90

Download this table (CSV)

* The extended assembly statistics are found in Table S1.

Table V: Structural annotation table directly from GenomeQC.

	Gene Models	Min. Gene Length (bp)	Max. Gene Length (bp)	Avg. Gene Length (bp)	Gene Models <200 bp	Transcripts	Avg. Transcripts per Gene Model	Exons	Avg. Exons per Transcript	Avg. Exon Length (bp)
A. hypogaea	67128	163	342359	3972.3	48	84714	1.3	423763	4.8	296.4
B. distachyon	36647	90	47411	3012.5	175	47917	1.3	263865	5.5	314.1
C. sativa	29815	21	976063	3450.0	2187	34876	1.2	234157	6.6	291.9
G. max	52872	90	94733	4017.0	1363	86256	1.6	-	-	-
G. raimondii	37505	90	51175	3243.5	71	77267	2.1	527563	6.7	271.3
H. vulgare	-	-	-	-	-	248270	-	1715898	-	278.9
M. truncatula A17	51541	60	102191	2566.9	1985	74213	1.4	318421	4.4	313.2
M. truncatula R108	55706	72	62996	2232.4	2404	61019	1.1	220904	3.6	270.6
O. sativa	35825	72	304271	3098.7	57	42378	1.2	192499	4.5	350.3
P. vulgaris	27433	93	90772	3943.8	108	36995	1.3	-	-	-
S. bicolor	34129	96	88337	3713.2	56	47121	1.4	266301	5.6	350.9
T. aestivum	107891	54	124945	3488.9	686	133744	1.2	749233	5.8	303.3
V. unguiculata	29773	90	81066	3880.9	408	42287	1.4	-	-	-
Z. mays B73	39324	111	128402	4117.8	38	151959	3.9	1488381	9.7	281.5
Z. mays Mo17	38620	21	146217	4076.8	1225	46530	1.2	272323	5.9	297.1
Z. mays PH207	40557	111	714207	5993.3	53	40557	1.0	208149	5.1	252.1
Z. mays W22	40691	87	154495	4330.4	64	51717	1.3	313830	6.1	292.4

Download this table (CSV)
Dashes (-) represent missing data.
Missing data in *H. vulgare* is due to the absence of gene model information in the GFF file.
Missing data in *G. max*, *P. vulgaris*, and *V. unguiculata* is due to the absence of exon information in their GFF files.

IX. SUPPLEMENTARY

Supplementary Table SI: Additional assembly statistics from GenomeQC.

	Longest Scaffold (bp)	Shortest Scaffold (bp)	Scaffolds >1K nt	Scaffolds >10K nt	Scaffolds >100K nt	Scaffolds >1M nt	Scaffolds >10M nt	N50	L50	NG50	LG50	%A	%C	%G	%T
A. hypogaea	1.61E+08	1000	382	299	42	23	20	1.35E+08	9	1.35E+08	10	31.79	18.14	18.13	31.79
B. distachyon	7.55E+07	2.88E+04	11	11	5	5	5	5.89E+07	3	5.89E+07	3	26.76	23.16	23.14	26.76
C. sativa	1.05E+08	6741	221	218	52	15	10	9.19E+07	5	9.19E+07	5	27.94	14.07	14.11	27.95
G. max	5.83E+07	885	281	271	63	20	20	4.99E+07	10	4.78E+07	11	31.77	16.91	16.91	31.77
G. raimondii	7.07E+07	1001	1033	200	20	14	13	6.22E+07	6	6.10E+07	7	32.81	16.32	16.32	32.81
H. vulgare	7.68E+08	2.50E+08	8	8	8	8	8	6.57E+08	4	5.83E+08	6	26.27	21.02	21.01	26.26
M. truncatula A17	5.66E+07	1004	2186	605	51	8	8	4.92E+07	4	4.57E+07	5	31.60	15.66	15.65	31.57
M. truncatula R108	3.23E+07	1007	909	481	72	49	18	1.28E+07	12	1.23E+07	14	33.25	16.41	16.41	33.25
O. sativa	4.33E+07	4236	63	51	17	12	12	3.00E+07	6	2.90E+07	8	28.21	21.77	21.78	28.21
P. vulgaris	6.30E+07	1005	478	290	61	13	11	4.97E+07	5	4.80E+07	6	31.84	17.61	17.64	31.86
S. bicolor	8.09E+07	1005	870	232	69	12	10	6.87E+07	5	6.87E+07	9	26.74	20.90	20.91	26.73
T. aestivum	8.31E+08	4.74E+08	22	22	22	22	22	7.10E+08	10	6.74E+08	12	26.46	22.59	22.59	26.46
V. unguiculata	6.53E+07	2922	686	679	103	13	11	4.17E+07	6	4.13E+07	7	33.31	16.40	16.42	33.34
Z. mays B73	3.07E+08	5568	266	261	48	12	10	2.24E+08	5	1.82E+08	6	26.18	23.09	23.10	26.19
Z. mays Mo17	3.06E+08	1007	2208	1864	129	11	10	2.20E+08	5	1.83E+08	6	26.14	23.03	23.05	26.17
Z. mays PH207	3.03E+08	500	16840	1115	61	11	10	2.15E+08	5	1.76E+08	6	21.34	18.40	18.40	21.36
Z. mays W22	3.11E+08	1.25E+07	11	11	11	11	11	2.23E+08	5	1.83E+08	6	26.11	22.92	22.94	26.13

Download this table (CSV)

N50: The length of the shortest scaffold/contig in the list of L50 sequences.
L50: The number of sequences whose sum of lengths make up 50% or more of the total assembly length.
NG50: The length of the shortest scaffold/contig calculated in the same manner as N50 but based on estimated genome size rather than total assembly length.
LG50: The number of sequences whose sum of lengths make up 50% or more of the estimated genome size.

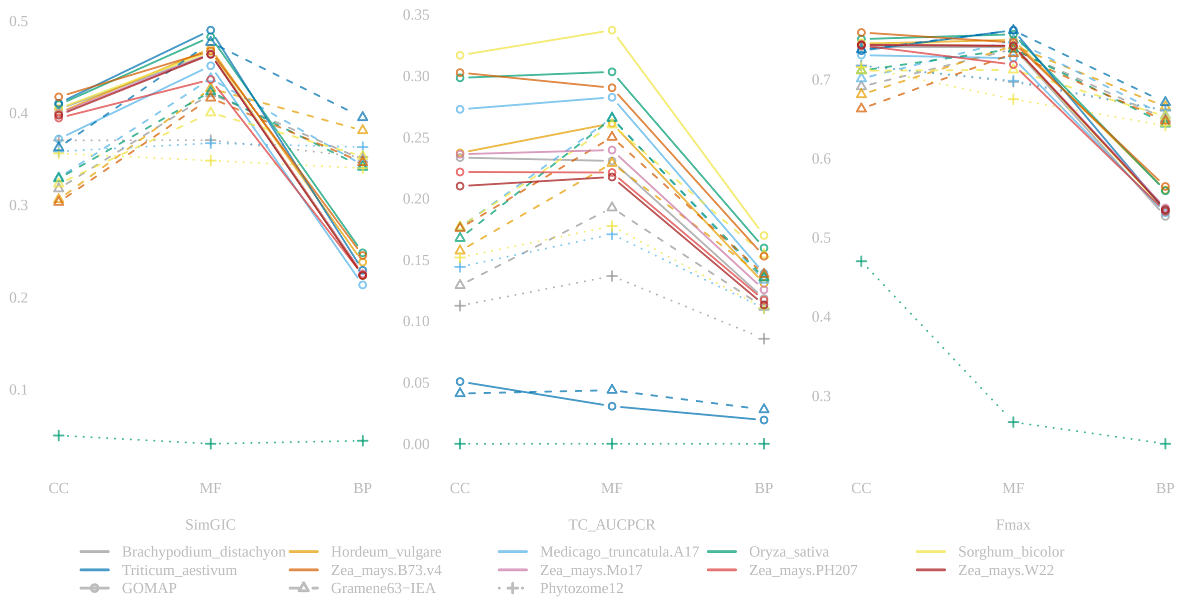

Supplementary Figure S1: Quality scores of the predicted annotation visualized. Line and symbol color designate the genome while line and symbol type designate prediction method/dataset. Please note that scales are different for each metric (values are not directly comparable).

Supplementary Table SII: Number of removed annotations during cleanup.

Genome	Dataset	Obsolete Annotations	Duplicates	Annotations with Modifiers
Arachis hypogaea	GOMAP	3437	13	912
Brachypodium distachyon	GOMAP	2512	49	789
	GoldStandard	21	204	0
	Gramene63-IEA	166	114	0
	Phytozome12	99	18	0
Cannabis sativa	GOMAP	1714	6	757
Glycine max	GOMAP	3333	10	930
Gossypium raimondii	GOMAP	1781	7	822
Hordeum vulgare	GOMAP	1877	8	815
	GoldStandard	1	9	0
	Gramene63-IEA	282	147	0
Medicago truncatula A17	GOMAP	2673	10	798
	GoldStandard	2	23	0
	Gramene62-IEA	429	251	0
	Gramene63-IEA	309	243	0
	Phytozome12	132	17	0
Medicago truncatula R108	GOMAP	4168	7	803
Oryza sativa	GOMAP	1642	7	869
	GoldStandard	37	833	0
	Gramene61-IEA	242	28	0
	Gramene63-IEA	238	64	0
	Phytozome12	119	19	0
Phaseolus vulgaris	GOMAP	1190	6	783
Pinus lambertiana	GOMAP	1839	4	587
Sorghum bicolor	GOMAP	2384	66	783
	GoldStandard	178	219	0
	Gramene63-IEA	278	198	0
	Phytozome12	131	12	0
Triticum aestivum	GOMAP	9624	17	1132
	GoldStandard	1	5	0
	Gramene61-IEA	706	88	0
	Gramene63-IEA	584	319	0
Vigna unguiculata	GOMAP	1269	6	811
	Phytozome12	122	27	0
Zea mays B73.v4	GOMAP	2077	89	848
	GoldStandard	50	633	0
	Gramene63-IEA	306	140	0
Zea mays Mo17	GOMAP	2346	83	823
	GoldStandard	36	1489	0
Zea mays PH207	GOMAP	2676	82	830
	GoldStandard	37	2702	0
Zea mays W22	GOMAP	2681	88	840
	GoldStandard	30	499	0

Download this table (CSV)
