# Supplementary material for: Standardized genome-wide function prediction enables comparative functional genomics: a new application area for Gene Ontologies in plants
Source: Gigascience. 2022 Apr 15;11:giac023. doi: 10.1093/gigascience/giac023 (PMC9012101; doi:10.1093/gigascience/giac023)
Supplement: giac023_GIGA-D-21-00269_Revision_1 [file giac023_giga-d-21-00269_revision_1.pdf]

GigaScience

Standardized genome-wide function prediction enables comparative functional genomics: a new application area for Gene Ontologies in plants

--Manuscript Draft--

Manuscript Number:	GIGA-D-21-00269R1
Full Title:	Standardized genome-wide function prediction enables comparative functional genomics: a new application area for Gene Ontologies in plants
Article Type:	Research
Funding Information:	Iowa State University Plant Sciences Institute — Dr. Carolyn J Lawrence-Dill<br>National Science Foundation (DGE-1545453) — Dr. Carolyn J Lawrence-Dill<br>Iowa State University Agricultural Experiment Station (Hatch Funds) (IOW04714) — Dr. Carolyn J Lawrence-Dill
Abstract:	Background: Genome-wide gene function annotations are useful for hypothesis generation and for prioritizing candidate genes potentially repsponsible for phenotypes of interest. We functionally annotated the genes of 18 crop plant genomes across 14 species using the GOMAP pipeline.<br>Results: By comparison to existing GO annotation datasets (available for a subset of these genomes), GOMAP-generated datasets cover more genes, contain more GO terms, and produce datasets similar in quality (based on precision and recall metrics using existing gold standards as the basis for comparison). From there, we sought to determine whether the datasets across multiple species could be used together to carry out comparative functional genomics analyses in plants. To test the idea and as a proof of concept, we created dendrograms of functional relatedness based on terms assigned for all 18 genomes. These dendrograms were compared to well-established species-level evolutionary phylogenies to determine whether trees derived were in agreement with known evolutionary relationships, which they largely are. Where discrepancies were observed, we determined branch support based on jack-knifing then removed individual annotation sets by genome to identify the annotation sets causing unexpected relationships.<br>Conclusions: GOMAP-derived functional annotations used together across multiple species generally retain sufficient biological signal to recover known phylogenetic relationships based on genome-wide functional similarities, indicating that comparative functional genomics across species based on GO data hold promise for generating novel hypotheses about comparative gene function and traits.
Corresponding Author:	Carolyn J Lawrence-Dill, Ph.D.<br>Iowa State University of Science and Technology: Iowa State University<br>Ames, IA UNITED STATES
Corresponding Author Secondary Information:	
Corresponding Author's Institution:	Iowa State University of Science and Technology: Iowa State University
Corresponding Author's Secondary Institution:	
First Author:	Leila Fattel
First Author Secondary Information:	
Order of Authors:	Leila Fattel<br>Dennis Psaroudakis<br>Colleen F. Yanarella<br>Kevin Chiteri<br>Haley A. Dostalik

Powered by Editorial Manager® and ProduXion Manager® from Aries Systems Corporation

	Parnal Joshi
	Dollye C. Starr
	Ha Vu
	Kokulapalan Wimalanathan, Ph.D.
	Carolyn J Lawrence-Dill, Ph.D.
**Order of Authors Secondary Information:**	
**Response to Reviewers:**	Please see cover file entitled, Response to Reviewers at end of file group.
**Additional Information:**	
**Question**	**Response**
Are you submitting this manuscript to a special series or article collection?	No
**Experimental design and statistics**<br><br>Full details of the experimental design and statistical methods used should be given in the Methods section, as detailed in our Minimum Standards Reporting Checklist. Information essential to interpreting the data presented should be made available in the figure legends.<br><br>Have you included all the information requested in your manuscript?	Yes
**Resources**<br><br>A description of all resources used, including antibodies, cell lines, animals and software tools, with enough information to allow them to be uniquely identified, should be included in the Methods section. Authors are strongly encouraged to cite Research Resource Identifiers (RRIDs) for antibodies, model organisms and tools, where possible.<br><br>Have you included the information requested as detailed in our Minimum Standards Reporting Checklist?	Yes
**Availability of data and materials**<br><br>All datasets and code on which the	Yes

conclusions of the paper rely must be either included in your submission or deposited in publicly available repositories (where available and ethically appropriate), referencing such data using a unique identifier in the references and in the “Availability of Data and Materials” section of your manuscript.<br><br>Have you have met the above requirement as detailed in our Minimum Standards Reporting Checklist?	

Powered by Editorial Manager® and ProduXion Manager® from Aries Systems Corporation

Manuscript and supplement - changes highlighted as describe in response to reviewers

Click here to access/download;Manuscript;Review_comparison.pdf

Standardized genome-wide function prediction enables comparative functional genomics: a new application area for Gene Ontologies in plants

Leila Fattel[1,*], Dennis Psaroudakis[2,*,†], Colleen F. Yanarella[1,], Kevin O. Chiteri[1,‡], Haley A. Dostalik[1,‡], Parnal Joshi[4,‡], Dollye C. Starr[1,‡], Ha Vu[5,‡], Kokulapalan Wimalanathan[5,§¶], and Carolyn J. Lawrence-Dill[1,5,**]

[1] *Department of Agronomy, Iowa State University*

[2] *Department of Plant Pathology and Microbiology, Iowa State University*

[3] *Department of Ecology, Evolution and Organismal Biology, Iowa State University*

[4] *Department of Veterinary Microbiology and Preventative Medicine, Iowa State University*

[5] *Department of Genetics, Development and Cell Biology, Iowa State University*

(Dated: December 28, 2021)

Background Genome-wide gene function annotations are useful for hypothesis generation and for prioritizing candidate genes potentially responsible for phenotypes of interest. We functionally annotated the genes of 18 crop plant genomes across 14 species using the GOMAP pipeline.

Results By comparison to existing GO annotation datasets, GOMAP-generated datasets cover more genes, contain more GO terms, and produce datasets similar in quality (based on precision and recall metrics using existing gold standards as the basis for comparison). From there, we sought to determine whether the datasets across multiple species could be used together to carry out comparative functional genomics analyses in plants. To test the idea and as a proof of concept, we created dendrograms of functional relatedness based on terms assigned for all 18 genomes. These dendrograms were compared to well-established species-level evolutionary phylogenies to determine whether trees derived were in agreement with known evolutionary relationships, which they largely are. Where discrepancies were observed, we determined branch support based on jack-knifing then removed individual annotation sets by genome to identify the annotation sets causing unexpected relationships.

Conclusions GOMAP-derived functional annotations used together across multiple species generally retain sufficient biological signal to recover known phylogenetic relationships based on genome-wide functional similarities, indicating that comparative functional genomics across species based on GO data hold promise for generating novel hypotheses about comparative gene function and traits.

Keywords: Gene function; ontology; plants; comparative genomics; functional genomics

I. BACKGROUND

Phenotypes and traits have long been the primary inspiration for biological investigation. Phenotypes are the result of a complex interplay between functions of genes and environmental cues. In an effort to organize and model gene functions, various systems of classification have been developed including systems like KEGG (the Kyoto Encyclopedia of Genes and Genomes), which is focused on protein function including gene activities superimposed on metabolic pathways [1]. Other such systems include the various Cyc databases, MapMan, and the Gene Ontologies (GO), a vocabulary of gene functions organized as a directed acyclic graph, which makes it innately tractable for computational analysis [2, 3, 4].

GO-based gene function annotation involves the association of GO terms to individual genes. Functions may be assigned to genes based on different types of evidence for the association. For example, functional predictions can be inferred from experiments (EXP), expression patterns (IEP), and more [5]. Computational pipelines are often used to generate functional predictions for newly sequenced genomes, where the genome is first sequenced and assembled, then gene structures (gene models) are predicted, then functions are associated with those gene predictions. Genome-wide gene function prediction datasets are frequently used to analyze gene expression studies, to prioritize candidate genes linked to a phenotype of interest, to design experiments aimed at characterizing functions of genes, and more [6, 7, 8]. How well a gene function prediction set models reality is influenced by how complete and correct the underlying genome assembly and gene structure annotations are as well as by how well the software used to predict functions performs.

GOMAP (the Gene Ontology Meta Annotator for Plants) is a gene function prediction pipeline for plants that generates high-coverage and reproducible functional annotations [9]. The system employs multiple functional prediction approaches, including sequence similarity, protein domain presence, and mixed-method pipelines developed to compete in the Critical Assessment of Function Annotation (CAFA) Challenge [10], a community challenge that has advanced the performance of gene function prediction pipelines over the course of five organized competitions [11].

*Contributed equally.
†Current address: Department of Molecular Biology, Leibniz Institute of Plant Genetics and Crop Plant Research (IPK), 06466 Seeland, OT Gatersleben, Germany
‡Listed alphabetically.
§kokul@bioinformapping.com
¶Current address: Greenlight Biosciences Inc., Medford, MA, 02155, USA
**triffid@iastate.edu

We previously annotated gene functions for the maize B73 genome and demonstrated that GOMAP's predicted functions were closer to curated gene-term associations from the literature than those of other community functional annotation datasets, including those produced by Gramene (Ensembl pipeline) and Phytozome (Inter-pro2GO pipeline) [12]. Using the newly containerized GOMAP system [9], we report here the functional annotation of 18 plant genomes across the 14 crop plant species shown in Table I and report comparisons of performance based on comparison to a Gold Standard gene function datasets, where possible.

Given these multiple annotations across various plant species, we next considered whether these datasets could be used together for comparative functional genomics in plants. We describe here a simple and crude method by which we used gene function annotations to generate dendrograms of genome-level similarity in function. This idea is similar to that of Zhu et al., who determined the evolutionary relationships among microorganisms based on whole-genome functional similarity [13]. Here we expand on that approach, analyzing genome-wide GO assignments to generate parsimony and distance-based dendrograms (see Figure 1 for process overview). We compared these with well-established species phylogenies (Figure 2) to determine whether trees derived from gene function show any agreement with evolutionary histories, taking agreement between generated dendrograms and known evolutionary histories to be evidence that sufficient comparative biological signal exists to begin to use GO functional annotations across multiple plant genomes for comparative functional genomics investigations.

II. RESULTS OF ANALYSES

A. Overview

As shown in Figure 1, gene function annotation sets were created and compiled for each genome. For those with existing annotation sets available on Gramene or Phytozome [50, 51], the datasets were compared. From there, matrices that included genomes as rows and terms as columns were generated. These were used directly to build parsimony trees or to create distance matrices for neighbor-joining tree construction [52, 53, 54]. In subsequent analyses, jackknifing was used to remove terms (columns) or to remove genomes (rows) to map the source of signal for treebuilding results [55].

B. Functional Annotation Sets Produced

Table II shows quantitative attributes of each of the annotation sets. In summary, GOMAP covers all annotated genomes with at least one annotation per gene, and provides between 3.8 and 12.1 times as many annotations as Gramene or Phytozome.

Quality evaluation of gene function predictions is not trivial and is approached by different research groups in different ways. Most often datasets are assessed by comparing the set of predicted functions for a given gene to a Gold Standard consisting of annotations that are assumed to be correct. This assumption of correctness can be based on any number of criteria. Here we used as our Gold Standard dataset all annotations present in Gramene63 that had a non-IEA (non-Inferred by Electronic Annotation) evidence code, i.e. we used only annotations that had some manual curation. This enabled us to assess the 10 genomes shown in Table II. It is perhaps noteworthy that the IEA and non-IEA annotation sets from Gramene63 frequently contain overlaps, indicating that some of the predicted annotations were manually confirmed afterwards by a curator and that in such cases, a new annotation was asserted with the new evidence code rather than simply upgrading the evidence code from IEA to some other code, thus preserving the IEA annotations in Gramene63 that are produced by the Ensembl analysis pipeline [56], a requirement for comparing GOMAP-produced IEA datasets to the IEA datasets produced by the Ensembl pipeline.

A general limitation of using Gold Standards for quality evaluation is that they can never be assumed to be complete, and therefore false positives in the prediction cannot be distinguished from false negatives in the Gold Standard. In other words, is gene X, function Y truly a wrong prediction or has it simply not yet been discovered experimentally? This problem is laid out in more detail in [57]. As a consequence, the quality of larger prediction sets will be systematically underestimated compared to smaller ones, and this effect is exacerbated the more incomplete the Gold Standard is.

There are many different metrics that have been used to evaluate the quality of predicted functional annotations. For the maize B73 GOMAP annotation assessment in [12], we had used a modified version of the hierarchical evaluation metrics originally introduced in [58] because they were simple, clear, and part of an earlier attempt at unifying and standardizing GO annotation comparisons [59]. In the meantime, Plyusnin et al. published an approach for evaluating different metrics showing variation among the robustness of different approaches to quality assessment [60]. Based on their recommendations, we use here the SimGIC2 and Term-centric Area Under Precision-Recall Curve (TC-AUCPCR) metrics. We also evaluated with the F_{max} metric, simply because it is widely-used (e.g., by [10]), even though according to Plyusnin et al., it is actually a flawed metric [60]. Results of the quality assessments for the 10 genomes where a Gold Standard was available are shown in Table III and Figure S2. While evaluation values differ between metrics and the scores are not directly comparable, a few consistent patterns emerge: GOMAP annotations are almost always better than Gramene and Phytozome annotations in the Cellular Component and Molecular Function aspect, with the only three exceptions being the Molecular

Table I: Functional annotation sets generated by GOMAP. More information about each dataset including the source of the input to GOMAP can be found at the respective DOI.

Species	Germplasm/Line	Assembly/Annotation	Dataset DOI	Genome Reference
Arachis hypogaea	Tifrunner	Arachis hypogaea assembly 1.0	[14]	[15]
Brachypodium distachyon	Bd21	Bd21.v3.1.r1	[16]	[17]
Cannabis sativa	Hemp	NCBI Cannabis sativa GCA_900626175.1	[18]	[19]
Glycine max	Williams 82	Joint Genome Institute (JGI) Wm82.a4.v1	[20]	[21]
Gossypium raimondii	Cotton D	Gossypium raimondii JGI v2.1	[22]	[23]
Hordeum vulgare	–	IBSC_PGSB_r1	[24]	[25]
Medicago truncatula	R108_HM340	R108: v1.0	[26]	[27]
Medicago truncatula	A17_HM341	Mt4.0v2	[28]	[29]
Oryza sativa	Japonica	IRGSP 1.0	[30]	[31]
Phaseolus vulgaris	G19833	DOE-JGI and USDA-NIFA annotation 2.0	[32]	[33]
Pinus lambertiana	Sugar Pine	TreeGenesDB sugar pine assembly v1.5	[34]	[35]
Sorghum bicolor	BTx623	BTx623.v3.0.1.r1	[36]	[37]
Triticum aestivum	Chinese Spring	IWGSC RefSeq 1.1	[38]	[39]
Vigna unguiculata	IT97K-499-35	JGI annotation v1.1	[40]	[41]
Zea mays *	Mo17	Zm-Mo17-REFERENCE-CAU-1.0	[42]	[43]
Zea mays *	PH207	Zm-PH207-REFERENCE_NS-UIUC_UMN-1.0	[44]	[45]
Zea mays *	W22	Zm-W22-REFERENCE-NRGENE-2.0 Zm00004b.1	[46]	[47]
Zea mays *	B73	RefGen_V4 Zm00001d.2	[48]	[49]

Latest overview at https://dill-picl.org/projects/gomap/gomap-datasets/
* Previously published in [9].

Function aspect for *T. aestivum* using the TC-AUCPCR and the F_{max} metric and the Cellular Component aspect for *M. truncatula* A17 using the F_{max} metric. Conversely, GOMAP predictions achieve consistently lower quality scores in the Biological Process aspect with the exception of *B. dystachion*, *O. sativa*, and *S. bicolor* with the TC-AUCPR metric. Generally, annotations that are better in one aspect are also better in the other two aspects but the ranking of annotations does not necessarily hold across metrics. The Phytozome annotation for *O. sativa* is an outlier in terms of its comparative quality, potentially because it is based on a modified structural annotation that differs substantially from the Gold Standard and the other annotations under comparison.

C. Phylogenetic Tree Analyses

With the comparative quality of gene function predictions in hand, we approached the question of whether the datasets could be used together for comparative functional analysis across all genomes. As a simple first step, we began to work toward understanding the degree to which trees built based on gene functions agree with known, well-documented evolutionary relatedness. We constructed neighbor-joining and parsimony trees of the 18 plant genomes, and visulized them using iTOL [61]. The two tree topologies, rooted at *P. lambertiana*, were compared to one another and to the topology of the expected tree (Figure 2). For both the neighbor-joining (Figure 3a) and parsimony trees (Figure 3b), one common difference is noted: *S. bicolor* is not at the base of the *Z. mays* clade as expected, and is clustered with *B. distachyon* instead. Notable differences between the neighbor-joining and parsimony tree are the following: *C. sativa* appears at the base of the eudicots instead of *G. raimondii* in the neighbor-joining tree, while *G. raimondii* is grouped with *C. sativa* and *A. hypogaea* is grouped with *G. max* in the parsimony tree. Second, *O. sativa* was expected to be at the base of the BOP clade, but appears at the base of *Z. mays* in the neighbor-joining tree, and at the base of all angiosperms in the parsimony tree. Differences among relationships within the *Z. mays* clade constaining B73, PH207, W22, and Mo17 were disregarded given the high degree of similarity across annotation sets and the fact that these relationships are not clear given the complex nature of within-species relationships.

Due to differences between the function-based dendrograms and the expected tree, jackknifing analysis was carried out by removing terms (columns in underlying datasets) to determine the degree to which the underlying datasets support specific groupings based on functional term assignments. This analysis was carried out for both neighbor-joining and parsimony trees. First, trees were generated by omitting 5% to 95% of the dataset in increments of 5 to determine the threshold at which the tree topologies deviated from those generated using the full dataset. That threshold was reached at 45% for both neighbor-joining and parsimony; therefore, we used trees generated with 40% of the data removed for reporting branch support values for the topology (Figure 3). Comparing the two trees, the parsimony topology was not as solid as that of the neighbor-joining at jackknife values up to 40%. Based on this robustness for neighbor-joining treebuilding in general, we carried out all subsequent analyses using neighbor-joining treebuilding methods.

We considered investigating the effect of using one

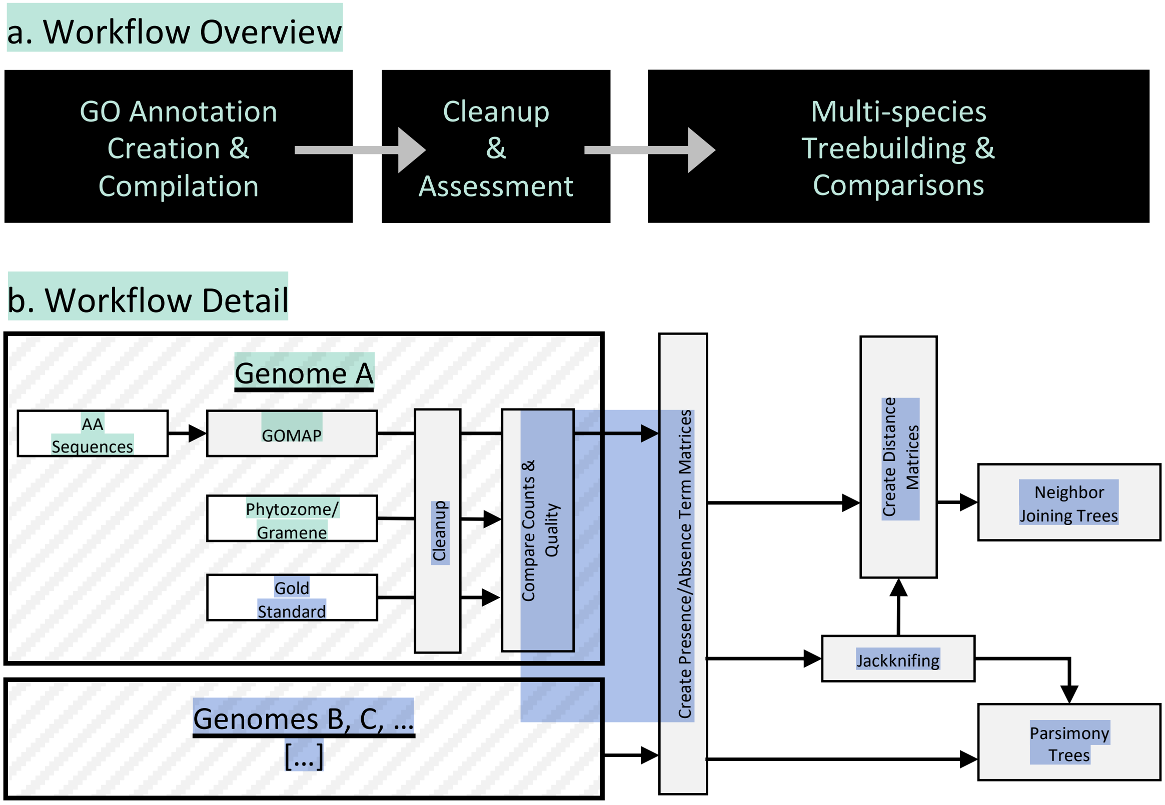

Figure 1: Data workflow schema. The workflow overview is shown in panel 'a' with steps represented as black boxes and the flow of information and processes indicated by arrows. Details are shown in panel 'b' where the upper large hatched box shows process detail for a single genome and the lower hatched box represents additional genomes for which the details of processing are identical. White boxes represent input datasets. Arrows indicate the flow of information and processes.

GO aspect to generate our neighbor-joining tree. In other words, we generated the neighbor-joining trees using cellular component GO terms, molecular function GO terms, and biological process GO terms separately (Supplementary Figure 3). Of the 14,303 total GO terms, 1,524 are cellular component terms, 3,926 are molecular function terms, and 8,853 are biological process terms. Out of the three single aspect phylogenetic trees, the one built using molecular function terms is the closest to our neighbor-joining tree obtained using all GO terms in our datasets Figure 3a. The only difference is that *A. hypogaea* and *G. max* are clustered in the molecular function tree, while they are not in our neighbor-joining tree Figure 3a. In the cellular component tree, *G. raimondii* and *C. sativa* are clustered together when they are not in the neighbor-joining tree with all GO aspects Figure 3a. Also, *O. sativa* is at the base of the monocots just like in the expected tree, but not in the neighbor-joining tree Figure 3a. In the biological process tree, *O. sativa* is at the base of the angiosperms and there is no clear separation between monocots and dicots. In all the three single aspect phylogenetic trees and our all-aspect neighbor-joining tree, *A. hypogaea* is never placed at the base of the NPAAA clade. Also, *B. distachyon* and *S. bicolor* are always clustered together. Overall, the topologies constructed using one GO term aspect at a time are close to that of our neighbor-joining tree, such that not one GO term aspect alone restored the topology of the expected tree.

To map the source of discrepancies to specific gene annotation sets, we generated various neighbor-joining trees excluding one genome each time, an additional tree with both *Medicago* genomes excluded simultaneously, and another with all *Z. mays* genomes excluded simultaneously. To exemplify this, see the monocot clade in Figure 2 and the lower (monocot) clade in Figure 3a. When the neighbor-joining tree was generated, two species are misplaced: *S. bicolor* and *O. sativa*. As shown in Figure 5a, removal of *O. sativa* corrects one error (itself) but does not correct the errant grouping of *S. bicolor* with *B. distachyon*. In Figure 5b, it is shown that the removal of *S. bicolor* corrects the errant grouping of itself and *B. distachyon*, but *O. sativa* placement remains incorrect. However, as shown in Figure 5c, the removal of *B. distachyon* generates a tree where all relationships are in agreement with known species-level relationships. (Note well: all individual annotation sets were progressively removed, not just the three shown in the example.)

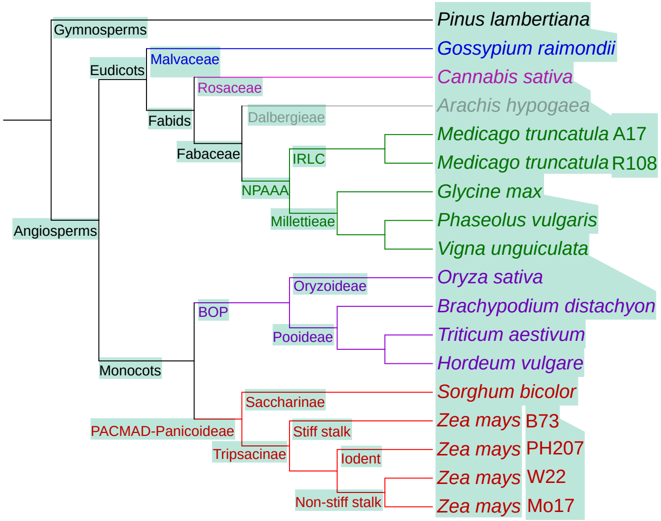

Figure 2: Known phylogenetic relationships among species. Cladogram is rooted by the gymnosperm *Pinus lambertiana* (black). Among angiosperms, eudicots clades include Malvaceae (blue), Rosaceae (magenta), Dalbergieae (grey), and NPAAA (green). Monocots include members of the BOP (purple) and PACMAD-Panicoideae (red) clades.

With this observation in hand, we sought to determine the minimum number of genomes that could be removed to create a tree that matched the expected tree topology. All possible combinations of removing 0-5 genomes to restore the topology were tested, and 10 combinations of minimum amount of genomes to be removed were obtained. The removal of four genomes was required to generate function-based trees consistent with known phylogenetic relationships. Of the 10, we selected the one that had the genomes that were most frequently part of a solution (*O. sativa*, 8; *B. distachyon*, 7; *C. sativa*, 6; *A. hypogaea*, 5; *S. bicolor*, 4; *G. raimondii*, 4; *G. max*, 4; *T. aestivum*) to show in this paper (the other combinations can be found in our publicly available dataset). To elaborate, the genomes removed here are *O. sativa*, *B. distachyon*, *C. sativa*, and *A. hypogaea* (Figure 4). Jackknifing analysis was also carried out for this dataset with support shown. Branch support is generally higher than that for the full dataset (i.e., branch support is higher in Figure 4 than in Figure 3a), and removing genomes that are causing variations seem to stabilize the tree.

D. Potential Causes of Unexpected Groupings

As a first step toward explaining discrepancies between known evolutionary relationships and those resulting from comparative analysis of genome-wide gene function predictions, we assessed the quality of each genome assembly and structural annotation set using GenomeQC [62]. Tables IV-V and Figures 6-7 represent the resulting assembly quality, structural annotation measures of quality, and proportion of single-copy BUSCOs (Benchmarking Universal Single-Copy genes) [63] that were generated. Although these analyses make evident that the species annotated are comparatively different in both natural genome characteristics and in assembly and annotation quality aspects, it is not the case that the four species responsible for deviations between the functional annotation dendrograms and known phylogenetic relationships (i.e., *C. sativa*, *A. hypogaea*, *O. sativa* and *B. distachyon*) create these discrepancies due to issues of genome assembly and/or annotation quality. One potential for some explanation is in relation to *C. sativa*, which is the only genome that has an assembly length larger than the expected (see IV), and a comparatively large proportion of missing BUSCOs in the assembly (see Figure 6). Similarly, for *A. hypogaea* and *O. sativa*, there is a large proportion of missing BUSCOs in the annotations (see Figure 7).

III. DISCUSSION

In this study, we used the GOMAP pipeline to produce whole-genome GO annotations for 18 genome assembly and annotation sets from 14 plant species [9]. Assessments of the number of terms predicted as well as the quality of predictions indicate that GOMAP functional prediction datasets cover more genes, contain more predictions per gene, and are of similar quality to prediction datasets produced by other systems, thus supporting the notion that these high-coverage datasets are a useful addition for researchers who are interested in genome-level analyses, including efforts aimed at prioritizing candidate genes for downstream analyses. Given that we can now produce high-quality, whole genome functional annotations for plants in a straightforward way, we intend to produce more of these over time (indeed we recently annotated *Vitis vinifera* [64], *Brassica rapa* [65], *Musa acuminata*[66], *Theobroma cacao* [67], *Coffea canephora* [68], *Vaccinium corymbosum* [69] *Solanum lycopersicum* [70], and *Solanum pennellii* [71]).

With 18 genome functional annotations in hand, we sought to determine whether and how researchers could use multispecies GO annotation datasets to perform comparative functional genomics analyses. As a proof of concept, we adapted phylogenetic tree-building methods to use the gene function terms assigned to genes represented by the genomes to build dendrograms of functional relatedness and hypothesized that if the functions were comparable across species, the resulting trees would closely match evolutionary relationships. To our delight and surprise, the neighbor-joining and parsimony trees (Figure 3) did resemble known phylogenies, but were not exact matches to broadly accepted phylogenetic relationships.

After removing the minimum number of genomes that resulted in restoration of the expected evolutionary relationships, we found that the individual species that may be responsible for the discrepancies observed in Figure 3

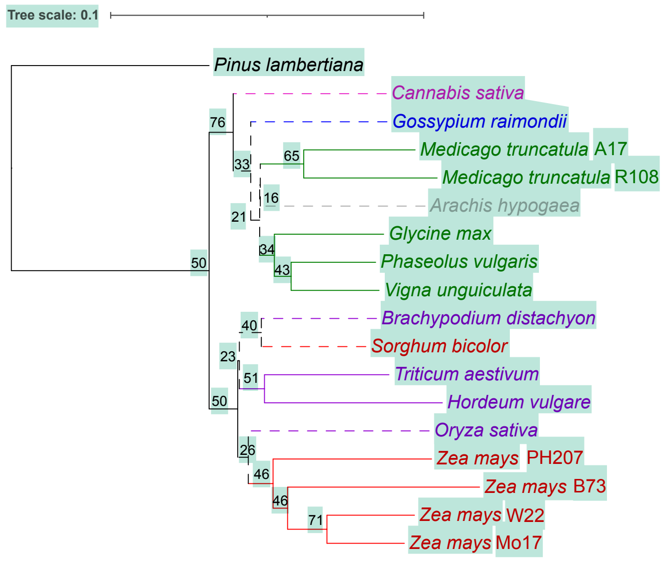

(a) neighbor-joining tree

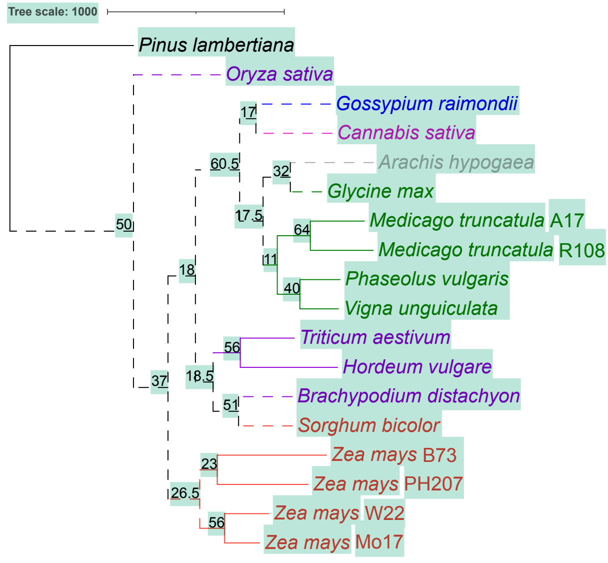

(b) parsimony tree

Figure 3: Neighbor-joining and parsimony trees. Phylograms are colored and rooted as described in Figure 2. For both neighbor-joining (a) and parsimony (b), node values represent the jackknifing support values derived by removing 40% of GO terms in the dataset. Dashed lines mark deviations from known phylogenetic relationships. Tree scales are shown above each, with NJ showing distances and parsimony showing changes in character state.

were *C. sativa*, *A. hypogaea*, *O. sativa* and *B. distachyon*. We hypothesize that the following could account for such errant relationships:

1) Quality of sequencing and coverage assembly: genomes of similarly high sequence coverage that have excellent gene calling would be anticipated to create the best source for functional annotation. Genomes of comparatively lower, or different, character would be anticipated to mislead treebuilding and other comparative genomics approaches.

2) Shared selected or natural traits: species that have been selected for, e.g., oilseeds may share genes involved in synthesis of various oils. Other shared traits would be anticipated to cause similarities for species with those shared traits.

3) Lack of good representation of diverse plant biology aspects in the GO graph: most plant-specific GO terms were derived from functional analysis of one model species, *Arabidopsis thaliana*. This single-source for presence of plant-specific functions limits the graph from containing unique functional aspects of plant biology represented in other species' genomes.

4) Use of a simple method of treebuilding based on the presence or absence of gene function terms: the method we devised and describe here is not sophisticated enough to make full use of information in the GO graphs such that we recover the full detail of the species' evolutionary histories from the simple method.

To consider the first of these, we looked at genome assembly and annotation quality metrics (see Tables IV-V and Figures 6-7). For *B. distachyon* we could find no compelling evidence that assembly structural or functional annotation quality differed significantly from all others, except in the case of *C. sativa*, where we noted that the assembly length exceeded the predicted genome size based on C-values for genome sizes reported previously [72]. In this case, the fact that the *C. sativa* line sequenced is not inbred [73] may be responsible for the inflated assembly size relative to what is expected. This means that in the assembly, there are likely regions where alleles between chromosomes do not align, which would inflate the overall length of the assembly. In addition, the assembly misses a large proportion of BUSCO genes compared to most other genomes included in this analysis. Indeed, the comparatively low-quality assembly for *Cannabis* genome has been noted by others [74], and our preliminary investigations indicate that the assembly length is in fact longer than expected.

In an attempt to better understand conflicting phylogenetic signals that could be caused by the second potential cause, i.e., shared selected or natural traits, we mapped all GO terms that exist in our binary matrix and traced character history (presence/absence) on the nodes and leaves of our expected evolutionary tree using the software Mesquite version 3.61 [75]. These data summarize the gain or loss of each GO term across the species described in this paper and can be found in our Github repository. We carried out a number of simple experiments to reveal which terms could be causal for errant relationships (e.g., dropping all unique terms from the *B. distachyon* dataset, reconstructing the term states at nodes that should be where *B. distachyon* should occur, etc., and could not identify any biologically compelling patterns. (Because these analyses were not fruitful, they

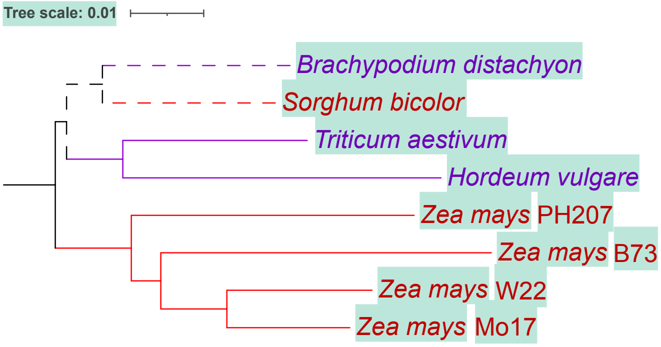

(a) monocots after removing *Oryza sativa*

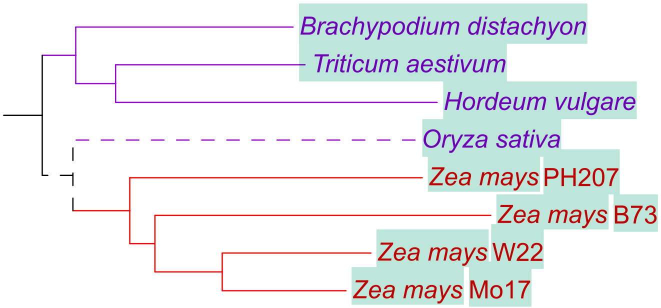

(b) monocots after removing *Sorghum bicolor*

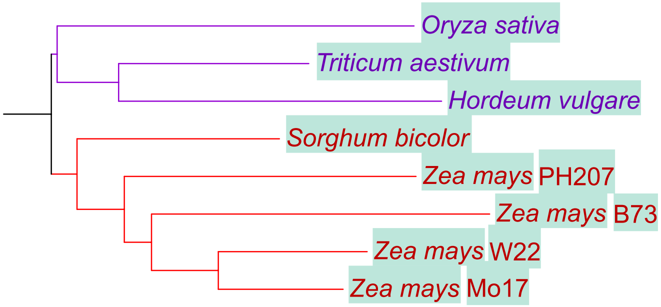

(c) monocots after removing *Brachypodium distachyon*

Figure 4: Restoring monocot relationships. Phylograms are colored and rooted as described in Figure 2. Dashed lines mark deviations from known phylogenetic relationships. Monocot topology changes with removal of a single species: (a) *O. sativa*, (b) *S. bicolor*, and (c) *B. distachyon*. Tree scale is shown above

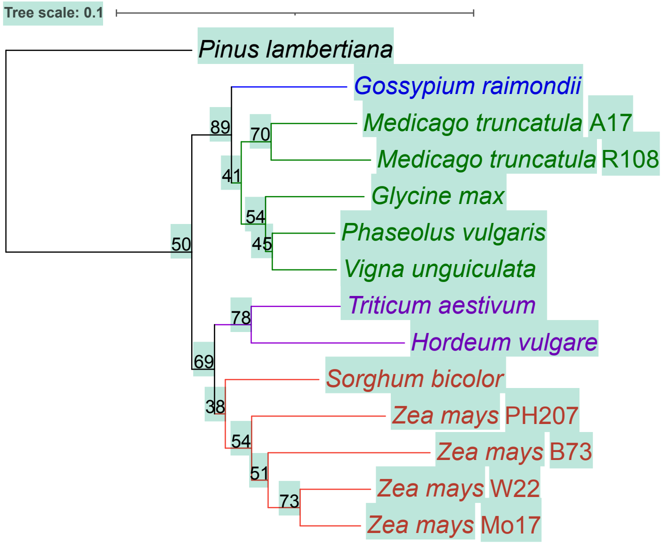

Figure 5: Restoring known phylogentic relationships to the NJ tree via removal of a minimal number of species. Phylograms are colored and rooted as described in Figure 2. Node values represent the jackknifing support values derived by removing 40% of GO terms in the dataset. 4 genomes have been removed: *C. sativa*, *O. sativa*, *B. distachyon*, and *A. hypogaea*. Tree scale is shown above.

were not specifically included in our materials and methods, though we do include the input datasets here, at the link provided in section V "Availability of Source Code and Supporting Data" of the paper, for others to consider and peruse independently.)

An important limitation that must be mentioned is the effect of the third potential cause of errant on our generated phylogenetic trees: a deficiency of terms describing plant biology. Because most GO terms specific to plant biology are likely derived from *Arabidopsis*, a model dicot species, gene functions unique to other species are expected to be missing from the GO graphs [76]. This source of error will only be corrected over time as gene functions unique to diverse plant species are populated into the GO graph.

We consider the most likely explanation for observed discrepancies between the known evolutionary phylogenies and dendrograms created based on GO terms describing gene function to be a result of the fourth explanation: the simplicity of the treebuilding models and methods we used for these analyses. Because the treebuilding and analytics described in this paper were based on the presence/absence of GO terms, novel terms are highly influential to the outcomes of the analysis and the number of times a term is used does not influence the outcome at all. In contrast, plant genomes are notable for having many duplicated genes as a result of whole-genome and segmental duplications over evolutionary history, so these duplications are in fact a feature of and marker for what happened to that genome over time. Therefore, using presence/absence of GO terms where the count of term occurrences are not weighted may be too simple to get at the genuine biological complexities represented in any given plant genome. Our simplistic demonstration of the utility of GO datasets for comparative functional genomics shows that more sophisticated methods are very promising for comparative functional genomics analyses.

It should be noted that comparative analyses using gene functions are not completely absent from the literature - though they are absent for large genome comparisons in plants. An example of such existing comparative use of GO is one where a given tree topology was

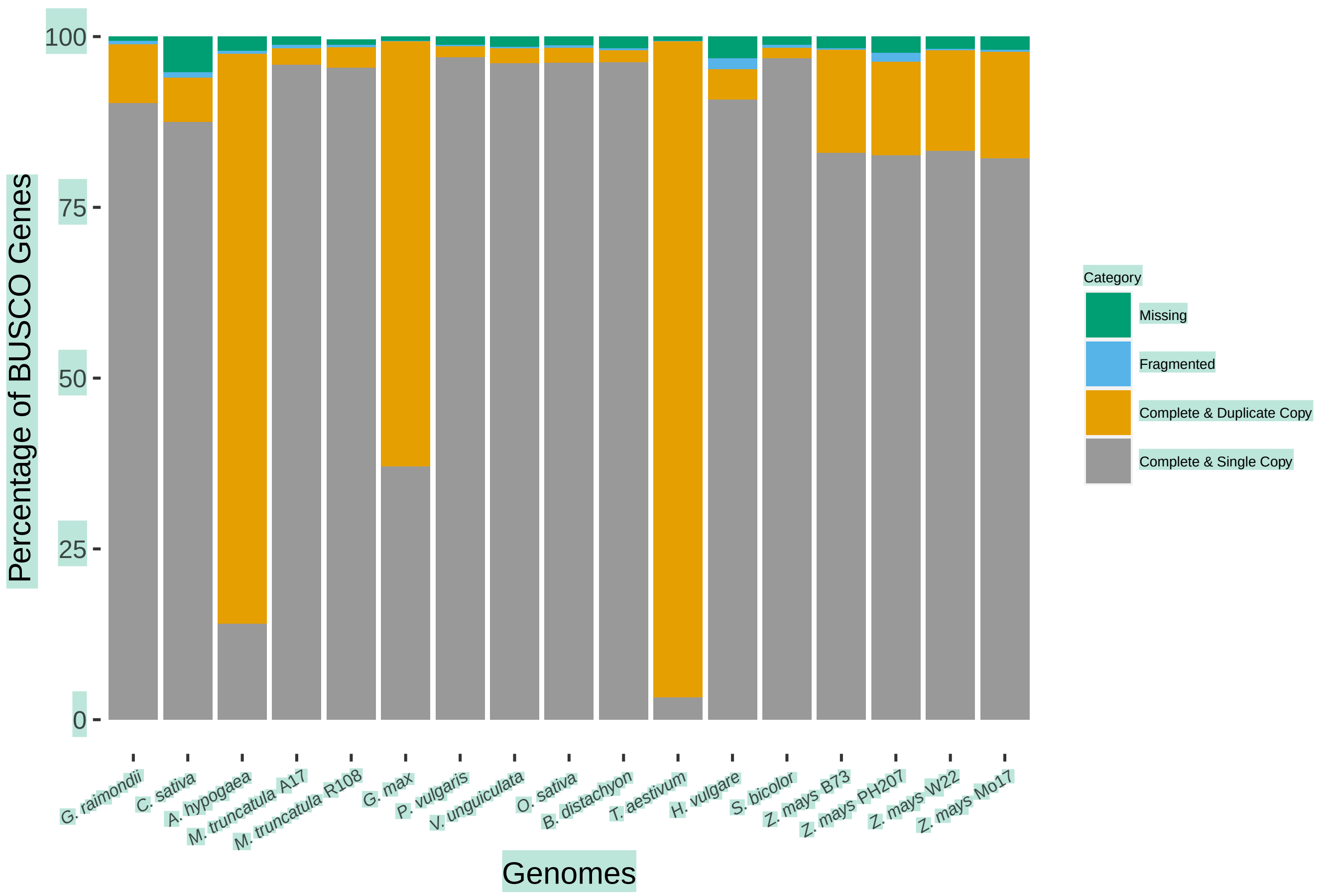

Figure 6: Assembly BUSCO plot generated using GenomeQC. Genomes analyzed are shown across the X-axis, and are ordered to match the occurrence of species shown in Figure 2. Percentage of BUSCO genes across four gene categories are stacked, with each adding up to 100 percent (Y-axis). Complete and single-copy genes are shown in grey, complete and duplicated copies in orange, fragmented copies in blue, and missing are shown copies in green.

used to look for gains and losses of functions mapped to independently derived trees, which was reported by Schwacke et al. [77]. They report, as an example of their method, an analysis of gene loss in *Cuscuta*, a parasitic plant based on analysis using the Mapman ontology. In their work, they showed considerable loss of genes, which is a hallmark of the parasitic lifestyle. Our efforts differ in that we used the functions directly to infer tree structures as a demonstration that sufficient biological signal is present in GO-based datasets of genome-wide function prediction to reproduce known biological relationships. The method we used was quick and dirty, and we anticipate that refinements in approach that consider multiple copies of genes, as well as using different types of graph and network representations beyond tree structures, are logical next steps for refining the use of GO terms for comparative functional genomics analyses in plants. With that in mind, we look forward not only to developing systems to support GO-based comparative functional genomics tools, but also to seeing the tools other research groups will develop to approach the use of these datasets to formulate novel comparative functional genomics hypotheses.

IV. METHODS

A. Acquiring Input Datasets

For each of the 18 genomes listed in Table I, information on how to access input annotation products are listed by DOI. For each, one representative translated peptide sequence per protein coding gene was selected and used as the input for GOMAP, a gene function prediction tool for plants that is actively maintained, updated, and versioned. Details of how GOMAP annotations are derived including the specificity of component datasets and which terms are retained are described elsewhere [12] [9]. In brief, GOMAP annotations are a combination of the annotations from multiple sources. GOMAP combines the annotations from all the sources and removes the less specific annotations that could be

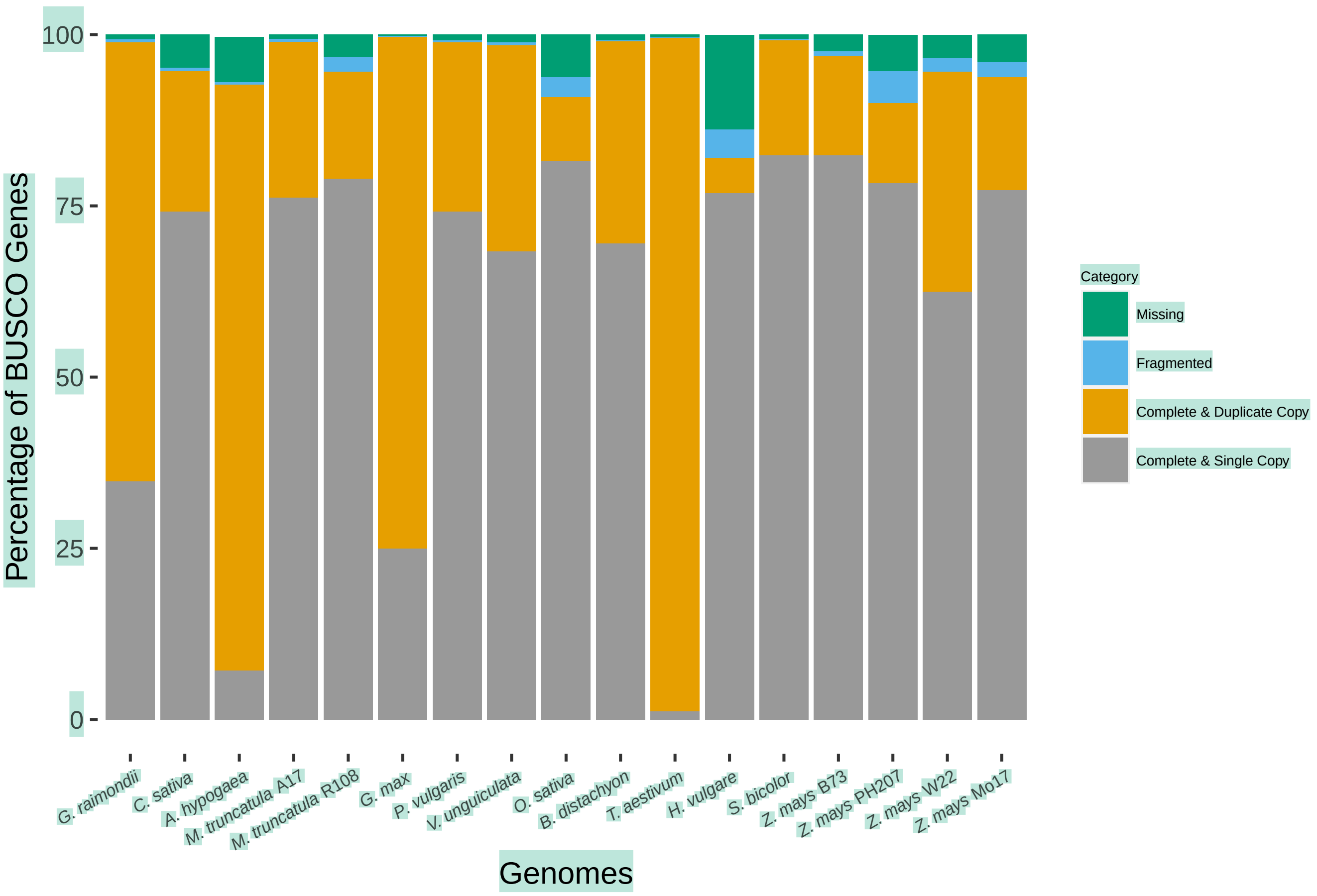

Figure 7: Annotation BUSCO plot generated using GenomeQC. Genomes analyzed are shown across the X-axis, and are ordered to match the occurrence of species shown in Figure 2. Percentage of BUSCO genes across four gene categories are stacked, with each adding up to 100 percent (Y-axis). Complete and single-copy genes are shown in grey, complete and duplicated copies in orange, fragmented copies in blue, and missing are shown copies in green.

inferred from the more specific annotations, keeping only the most specific terms for each gene that cannot be inferred from other terms (i.e., only leaf terms). Unless the authors of the genome provided a set of representative sequences designated as canonical, we chose the longest translated peptide sequence as the representative for each gene model. In general, non-IUPAC characters and trailing asterisks (*) were removed from the sequences, and headers were simplified to contain only non-special characters. The corresponding script for each dataset can be found at the respective DOI. Based on this input, GOMAP yielded a functional annotation set spanning all protein-coding genes in the genome. Using the Gene Ontology version releases/2020-10-09, this functional annotation set was cleaned up by removing duplicates, annotations with qualifiers (NOT, contributes_to, colocalizes_with; column 4 in the GAF 2.1 format), and obsolete GO terms. Any terms containing alternative identifiers were merged to their respective main identifier, uncovering a few additional duplicates, which were also removed. Table SII shows the number of annotations removed from each dataset produced.

To compare the quality of GOMAP predictions to currently available functional predictions from Gramene and Phytozome, we downloaded IEA annotations from Gramene (version 63, [50], https://www.gramene.org/) and Phytozome (version 12, [51], https://phytozome.jgi.doe.gov/) for each species with functional annotations of the same genome version. These datasets were cleaned as above. Similarly cleaned non-IEA annotations from Gramene63 served as the Gold Standard wherever they were available. More detailed information on how these datasets were accessed can be found at https://github.com/Dill-PICL/GOMAP-Paper-2019.1/blob/master/data/go_annotation_sets/README.md.

B. Quantitative and Qualitative Evaluation

The number of annotations in each clean dataset was determined and related to the number of protein coding

genes (based on transcripts in the input FASTA file). This was done for separately for each GO aspect as well as in total.

The ADS software version published in [60] is available from https://bitbucket.org/plyusnin/ads/. We used version b6309cb (also included in our code as a submodule) to calculate SimGIC2, TC-AUCPCR, and F_{max} quality scores. To provide the information content required for the SimGIC2 metric, the Arabidopsis GOA from https://www.ebi.ac.uk/GOA/arabidopsis_release was used in version 2021-02-16.

C. Cladogram Construction

For clustering, we first collected all GO terms annotated to any gene in each genome into a list and removed the duplicates, yielding a one-dimensional set of GO terms for each genome (T). Next, we added all parental terms for each term in this set (connected via *is_a* in the ontology), their respective parental terms and higher, recursively continuing up to the very root of the ontology. Then we once again removed the duplicates, yielding a set S containing the original terms from set T as well as all terms proximal to them in the Gene Ontology directed acyclical graph. These sets with added ancestors served as a starting point of our tree-building analyses: pairwise distances between the genomes were calculated using the Jaccard distance as a metric of the dissimilarity between any two sets a and b.

$$d_{ab} = 1 - \frac{|S_a \cap S_b|}{|S_a \cup S_b|} \quad (1)$$

A neighbor-joining tree was constructed based on the generated pairwise distance matrix using PHYLIP [52]. Additionally, term sets S of all genomes were combined into a binary matrix (with rows corresponding to genomes and columns corresponding to GO terms, values of 0 or 1 indicating whether a term is present or absent in the given set). PHYLIP pars was used to construct a parsimony tree from this binary matrix.

P. lambertiana, a gymnosperm, was included in the dataset as an outgroup to the angiosperms to separate between the monocot and eudicot clades. iTOL [78] was used to visualize the trees using their Newick format, and root them at *P. lambertiana*. Moreover, a cladogram representing the known phylogeny of the included taxa was created by hand based on known evolutionary relationships [79, 80, 81, 82, 83]. This was used to compare the generated phylogenetic relationship based on functional similarity with the evolutionary relationships of the plant genomes.

Jackknifing analysis was carried out for both parsimony and neighbor-joining trees to assess the support for each clade based on the proportion of jackknife trees showing the same clade. To this end, 40% of the terms in T were randomly removed, ancestors of the remaining terms were added and trees constructed as above. The majority rule consensus tree of 100 individual trees was calculated with the jackknife values represented on each branch. The tree was then visualized using iTOL using its Newick format, and rooted again at *P. lambertiana*.

D. Genome Quality Evaluation

Genome size was estimated from the C-values obtained from the Plant DNA C-values data resource from the Kew Database (https://cvalues.science.kew.org). The mean C-value for a given species was used for calculating genome size estimates in base pairs (bp) using the method of [84]. In brief,

$$\text{Genome size (bp)} = \text{C-value (pg)} * 0.978\text{x}10^9 \frac{\text{bp}}{\text{pg}}$$

The estimated genome size (listed in Table IV) was used as an input for GenomeQC (https://genomeqc.maizegdb.org/) [62] to calculate quality metrics. For genomes that were too large to submit through the GenomeQC webtool or had missing exon information, modified scripts of those found in GitHub of GenomeQC (https://github.com/HuffordLab/GenomeQC, commit e6140ee) were applied to calculate the assembly and structural annotation metrics in Table IV and V. BUSCO version 5.0.0 [85] was used to calculate the assembly and annotation BUSCO scores, shown in Figures 6 and 7. The input for assembly BUSCO scores were chromosome sequences, whereas inputs were transcript/mRNA/CDS sequences for the annotation BUSCO scores. For the lineage parameter, the lineage datasets used were as follows: Eudicots for *C. sativa* and *G. raimondii*, Fabales for *A. hypogaea*, *M. truncatula* A17 and R108, *P. vulgaris*, *G. max*, and *V. unguiculata*, and Poales for *B. distachyon*, *O. sativa*, *T. aestivum*, *H. vulgare*, *S. bicolor* and *Z. mays* B73, Mo17, W22 and PH207.

V. AVAILABILITY OF SOURCE CODE AND SUPPORTING DATA

All data and source code generated are freely available at https://github.com/Dill-PICL/GOMAP-Paper-2019.1 under the terms of the MIT license. All software requirements and dependencies are packaged into a Singularity container so no other setup is required to reproduce our results. We will provide a DOI through Zenodo for the final version of the manuscript after reviews and corrections are incorporated.

An up-to-date list of all available annotation sets can be found at https://dill-picl.org/projects/gomap/gomap-datasets/.

VI. DECLARATIONS

A. Competing Interests

The author(s) declare that they have no competing interests.

B. Funding

This work has been supported by the Iowa State University Plant Sciences Institute Faculty Scholars Program to CJLD, the Predictive Plant Phenomics NSF Research Traineeship (#DGE-1545453) to CJLD (CFY and KOC are a trainees), and IOW04714 Hatch funding to Iowa State University.

C. Author's Contributions

LF, DP, CFY, KOC, HAD, PJ, DCS, HV, and KW generated annotations for plants as described in this paper. DP and CJLD co-conceived the idea for phylogenetic analysis. DP wrote the code for the analyses in this paper. LF worked with DP to create dendrograms and compare those to phylogenetic trees. LF carried out assembly and annotation metric comparisons. LF, DP, and CJLD wrote the manuscript. All authors read, offered suggestions to improve, and approved the final copy of the manuscript.

VII. ACKNOWLEDGEMENTS

Thanks to Steven Cannon for help to understand phylogenetic relationships among eudicots and helpful discussions and to Toby Kellogg and Jedrzej Szymanski for discussions and ideas on how to consider the data. Thanks to Nancy Manchanda for reviewing documentation and for checking genome versions used as input for GenomeQC. Thanks to Darwin Campbell who guided the deposition of datasets with CyVerse for release and DOI assignment. Thanks to the reviewers for their suggestions which have improved this manuscript.

VIII. AUTHORS' INFORMATION

KW created the GOMAP system during his time as a graduate student at Iowa State University. LF, DP, CFY, KOC, HV, and PJ are currently graduate students. HAD and DCS are undergraduate students. Each graduate and undergraduate student annotated at least one genome over the course of a research rotation lasting no more than one semester. CJLD coordinated research activities and manuscript preparation.

References

[1] Minoru Kanehisa et al. "KEGG: integrating viruses and cellular organisms". In: *Nucleic Acids Research* 49.D1 (Oct. 2020), pp. D545–D551. DOI: 10.1093/nar/gkaa970. URL: https://doi.org/10.1093/nar/gkaa970.

[2] Michael Ashburner et al. "Gene Ontology: tool for the unification of biology". In: *Nature genetics* 25.1 (May 2000), pp. 25–29. ISSN: 1061-4036. DOI: 10.1038/75556. URL: https://www.ncbi.nlm.nih.gov/pmc/articles/PMC3037419/ (visited on 03/12/2021).

[3] The Gene Ontology Consortium et al. "The Gene Ontology resource: enriching a GOld mine". en. In: *Nucleic Acids Research* 49.D1 (Jan. 2021), pp. D325–D334. ISSN: 0305-1048, 1362-4962. DOI: 10.1093/nar/gkaa1113. URL: https://academic.oup.com/nar/article/49/D1/D325/6027811 (visited on 03/12/2021).

[4] Oliver Thimm et al. "mapman: a user-driven tool to display genomics data sets onto diagrams of metabolic pathways and other biological processes". In: *The Plant Journal* 37.6 (Mar. 2004), pp. 914–939. DOI: 10.1111/j.1365-313x.2004.02016.x. URL: https://doi.org/10.1111/j.1365-313x.2004.02016.x.

[5] D. Binns et al. "QuickGO: a web-based tool for Gene Ontology searching". In: *Bioinformatics* 25.22 (Sept. 2009), pp. 3045–3046. DOI: 10.1093/bioinformatics/btp536. URL: https://doi.org/10.1093/bioinformatics/btp536.

[6] Iris Tzafrir et al. "Identification of Genes Required for Embryo Development in Arabidopsis". en. In: *Plant Physiology* 135.3 (July 2004). Publisher: American Society of Plant Biologists Section: GENOME ANALYSIS, pp. 1206–1220. ISSN: 0032-0889, 1532-2548. DOI: 10.1104/pp.104.045179. URL: http://www.plantphysiol.org/content/135/3/1206 (visited on 03/12/2021).

[7] Ana Conesa and Stefan Götz. "Blast2GO: A Comprehensive Suite for Functional Analysis in Plant Genomics". en. In: *International Journal of Plant Genomics* 2008 (Apr. 2008), pp. 1–12. ISSN: 1687-5370, 1687-5389. DOI: 10.1155/2008/619832. URL: https://www.hindawi.com/journals/ijpg/2008/619832/ (visited on 03/12/2021).

[8] Seung Yon Rhee and Marek Mutwil. "Towards revealing the functions of all genes in plants". In: *Trends in Plant Science* 19.4 (Apr. 2014), pp. 212–221. ISSN: 1360-1385. DOI: 10.1016/j.tplants.2013.10.006. URL: https://www.sciencedirect.com/science/article/pii/S1360138513002343 (visited on 03/12/2021).

[9] Kokulapalan Wimalanathan and Carolyn J. Lawrence-Dill. "Gene Ontology Meta Annotator for Plants (GOMAP)". In: *Plant Methods* 17.1 (May 2021). DOI: 10.1186/s13007-021-00754-1. URL: https://doi.org/10.1186/s13007-021-00754-1.

[10] Naihui Zhou et al. "The CAFA challenge reports improved protein function prediction and new functional annotations for hundreds of genes through experimental screens". In: *Genome Biology* 20.1 (Nov. 2019). DOI: 10.1186/s13059-019-1835-8. URL: https://doi.org/10.1186/s13059-019-1835-8.

[11] URL: https://www.biofunctionprediction.org.

[12] Kokulapalan Wimalanathan et al. "Maize GO Annotation-Methods, Evaluation, and Review (maize-

GAMER)". In: *Plant Direct* 2.4 (Apr. 2018), e00052. ISSN: 24754455. DOI: 10.1002/pld3.52. URL: http://doi.wiley.com/10.1002/pld3.52.

[13] Chengsheng Zhu et al. "Functional Basis of Microorganism Classification". en. In: *PLOS Computational Biology* 11.8 (Aug. 2015). Publisher: Public Library of Science, e1004472. ISSN: 1553-7358. DOI: 10.1371/journal.pcbi.1004472. URL: https://journals.plos.org/ploscompbiol/article?id=10.1371/journal.pcbi.1004472 (visited on 03/12/2021).

[14] Dennis Psaroudakis and Carolyn Lawrence-Dill. *GOMAP Peanut IPGI 1.0*. 2019. DOI: 10.25739/CHAB-0E35. URL: http://datacommons.cyverse.org/browse/iplant/home/shared/commons_repo/curated/Carolyn_Lawrence_Dill_GOMAP_Peanut_Tifrunner.IPGI.1.0_August_2019.r1.

[15] David J. Bertioli et al. "The genome sequence of segmental allotetraploid peanut Arachis hypogaea". In: *Nature Genetics* 51.5 (May 2019), pp. 877–884. DOI: 10.1038/s41588-019-0405-z. URL: https://doi.org/10.1038/s41588-019-0405-z.

[16] Kokulapalan Wimalanathan and Carolyn Lawrence-Dill. *GOMAP Bdistachyon.Bd21.v3.1.r1*. 2019. DOI: 10.25739/DW2T-3G82. URL: http://datacommons.cyverse.org/browse/iplant/home/shared/commons_repo/curated/Carolyn_Lawrence_Dill_GOMAP_Bdistachyon.Bd21.v3.1_November_2019.r1.

[17] "Genome sequencing and analysis of the model grass Brachypodium distachyon". In: *Nature* 463.7282 (Feb. 2010), pp. 763–768. DOI: 10.1038/nature08747. URL: https://doi.org/10.1038/nature08747.

[18] Kevin Chiteri and Carolyn Lawrence-Dill. *GOMAP_Cannabis_sativa_NCBI-cs10_January_2020*. 2020. DOI: 10.25739/AB9Z-2Z86. URL: http://datacommons.cyverse.org/browse/iplant/home/shared/commons_repo/curated/Carolyn_Lawrence_Dill_GOMAP_Cannabis_NCBI-cs10_January_2020.r1.

[19] Kaitlin U. Laverty et al. "A physical and genetic map of Cannabis sativa identifies extensive rearrangements at the THC/CBD acid synthase loci". In: *Genome Research* 29.1 (Nov. 2018), pp. 146–156. DOI: 10.1101/gr.242594.118. URL: https://doi.org/10.1101/gr.242594.118.

[20] Dennis Psaroudakis and Carolyn Lawrence-Dill. *GOMAP Soybean JGI-Wm82.a4.v1*. 2019. DOI: 10.25739/59EC-1719. URL: http://datacommons.cyverse.org/browse/iplant/home/shared/commons_repo/curated/Carolyn_Lawrence_Dill_GOMAP_Soybean_JGI-Wm82.a4.v1_April_2019.r1.

[21] Jeremy Schmutz et al. "Genome sequence of the palaeopolyploid soybean". In: *Nature* 463.7278 (Jan. 2010), pp. 178–183. DOI: 10.1038/nature08670. URL: https://doi.org/10.1038/nature08670.

[22] Parnal Joshi and Carolyn Lawrence-Dill. *GOMAP Gossypium raimondii JGI v2.1*. 2020. DOI: 10.25739/A13T-ZH47. URL: http://datacommons.cyverse.org/browse/iplant/home/shared/commons_repo/curated/Carolyn_Lawrence_Dill_GOMAP_Gossypium_raimondii_JGI_v2.1_January_2020.r1.

[23] Andrew H. Paterson et al. "Repeated polyploidization of Gossypium genomes and the evolution of spinnable cotton fibres". In: *Nature* 492.7429 (Dec. 2012), pp. 423–427. DOI: 10.1038/nature11798. URL: https://doi.org/10.1038/nature11798.

[24] Colleen Yanarella and Carolyn Lawrence-Dill. *GOMAP Barley Reference Sequences IBSC_PGSB_r1*. 2019. DOI: 10.25739/ZVGV-8E37. URL: http://datacommons.cyverse.org/browse/iplant/home/shared/commons_repo/curated/Carolyn_Lawrence_Dill_GOMAP_Barley_IBSC_PGSB-1.0_May_2019.r1.

[25] Martin Mascher et al. "A chromosome conformation capture ordered sequence of the barley genome". In: *Nature* 544.7651 (Apr. 2017), pp. 427–433. DOI: 10.1038/nature22043. URL: https://doi.org/10.1038/nature22043.

[26] Dennis Psaroudakis and Carolyn Lawrence-Dill. *GOMAP Barrel Clover R108_HM340 v1.0*. 2019. DOI: 10.25739/2SQC-J140. URL: http://datacommons.cyverse.org/browse/iplant/home/shared/commons_repo/curated/Carolyn_Lawrence_Dill_GOMAP_BarrelClover_R108_HM340_v1.0_August_2019.r1.

[27] Karen M. Moll et al. "Strategies for optimizing BioNano and Dovetail explored through a second reference quality assembly for the legume model, Medicago truncatula". In: *BMC Genomics* 18.1 (Aug. 2017). DOI: 10.1186/s12864-017-3971-4. URL: https://doi.org/10.1186/s12864-017-3971-4.

[28] Dennis Psaroudakis and Carolyn Lawrence-Dill. *GOMAP Barrel Clover A17_HM341 Mt4.0v2*. 2019. DOI: 10.25739/PY38-YB08. URL: http://datacommons.cyverse.org/browse/iplant/home/shared/commons_repo/curated/Carolyn_Lawrence_Dill_GOMAP_BarrelClover_A17_HM341_Mt4.0v2_August_2019.r1.

[29] Haibao Tang et al. "An improved genome release (version Mt4.0) for the model legume Medicago truncatula". In: *BMC Genomics* 15.1 (2014), p. 312. DOI: 10.1186/1471-2164-15-312. URL: https://doi.org/10.1186/1471-2164-15-312.

[30] Ha Vu and Carolyn Lawrence-Dill. *GOMAP Rice Reference Sequences 2.0*. 2019. DOI: 10.25739/53G0-J859. URL: http://datacommons.cyverse.org/browse/iplant/home/shared/commons_repo/curated/Carolyn_Lawrence_Dill_GOMAP_Rice_IRGSP-1.0_April_2019.r2.

[31] Marcela K Tello-Ruiz et al. "Gramene 2018: unifying comparative genomics and pathway resources for plant research". In: *Nucleic Acids Research* 46.D1 (Nov. 2017), pp. D1181–D1189. DOI: 10.1093/nar/gkx1111. URL: https://doi.org/10.1093/nar/gkx1111.

[32] Dennis Psaroudakis and Carolyn Lawrence-Dill. *GOMAP Common Bean DOE-JGI and USDA-NIFA v2.0*. 2019. DOI: 10.25739/1YWE-EW96. URL: http://datacommons.cyverse.org/browse/iplant/home/shared/commons_repo/curated/Carolyn_Lawrence_Dill_GOMAP_CommonBean_DOE-JGI-USDA-NIFA.2.0_August_2019.r1.

[33] Oscar P Hurtado-Gonzales, Thiago AS Gilio, and MA Pastor-Corrales. "Resistant Reaction of Andean Common Bean Landrace G19833, Reference Genome, to 13 Races of Uromyces appendiculatus Suggests Broad Spectrum Rust Resistance". In: (2017).

[34] Colleen Yanarella and Carolyn Lawrence-Dill. *GOMAP TreeGenesDB sugar pine assembly v1.5*. 2020. DOI: 10.25739/JVS4-XR88. URL: http://datacommons.cyverse.org/browse/iplant/home/shared/commons_repo/curated/Carolyn_Lawrence_Dill_GOMAP_SugarPine_TreeGenesDB-1.5_January_2020.r1.

[35] Marc W Crepeau, Charles H Langley, and Kristian A Stevens. "From Pine Cones to Read Clouds: Rescaffolding the Megagenome of Sugar Pine (Pinus lambertiana)". In: *G3 Genes|Genomes|Genetics* 7.5 (May 2017), pp. 1563–1568. DOI: 10.1534/g3.117.040055. URL: https://doi.org/10.1534/g3.117.040055.

[36] Kokulapalan Wimalanathan and Carolyn Lawrence-Dill. *GOMAP Sbicolor.BTx623.v3.0.1.r1*. 2019. DOI: 10.25739/4TY0-YE98. URL: http://datacommons.cyverse.org/browse/iplant/home/shared/commons_repo/curated/Carolyn_Lawrence_Dill_GOMAP_Sbicolor.BTx623.v3.0.1_November_2019.r1.

[37] Ryan F. McCormick et al. "The Sorghum bicolor reference genome: improved assembly, gene annotations, a transcriptome atlas, and signatures of genome organization". In: *The Plant Journal* 93.2 (Dec. 2017), pp. 338–354. DOI: 10.1111/tpj.13781. URL: https://doi.org/10.1111/tpj.13781.

[38] Dennis Psaroudakis and Carolyn Lawrence-Dill. *GOMAP Wheat Reference Sequences 1.1*. 2019. DOI: 10.25739/65KF-JZ20. URL: http://datacommons.cyverse.org/browse/iplant/home/shared/commons_repo/curated/Carolyn_Lawrence_Dill_GOMAP_Wheat_RefSeq1.1_HC_December_2018.r1.

[39] Michael Alaux et al. "Linking the International Wheat Genome Sequencing Consortium bread wheat reference genome sequence to wheat genetic and phenomic data". In: *Genome Biology* 19.1 (Aug. 2018). DOI: 10.1186/s13059-018-1491-4. URL: https://doi.org/10.1186/s13059-018-1491-4.

[40] Dennis Psaroudakis and Carolyn Lawrence-Dill. *GOMAP Cowpea IT97K-499-35 JGI annotation v1.1*. 2019. DOI: 10.25739/CDX9-WR97. URL: http://datacommons.cyverse.org/browse/iplant/home/shared/commons_repo/curated/Carolyn_Lawrence_Dill_GOMAP_Cowpea_JGI.v1.1_August_2019.r1.

[41] Stefano Lonardi et al. "The genome of cowpea (Vigna unguiculata [L.] Walp.)" In: (Jan. 2019). DOI: 10.1101/518969. URL: https://doi.org/10.1101/518969.

[42] Kokulapalan Wimalanathan and Carolyn Lawrence-Dill. *GOMAP Maize Zm-Mo17-REFERENCE-CAU-1.0 Zm00014a.1*. 2019. DOI: 10.25739/M634-CN58. URL: http://datacommons.cyverse.org/browse/iplant/home/shared/commons_repo/curated/Carolyn_Lawrence_Dill_GOMAP_maize.Mo17.AGPv1_April_2019.r1.

[43] Silong Sun et al. "Extensive intraspecific gene order and gene structural variations between Mo17 and other maize genomes". In: *Nature Genetics* 50.9 (July 2018), pp. 1289–1295. DOI: 10.1038/s41588-018-0182-0. URL: https://doi.org/10.1038/s41588-018-0182-0.

[44] Kokulapalan Wimalanathan and Carolyn Lawrence-Dill. *GOMAP Maize Zm-PH207-REFERENCE_NS-UIUC_UMN-1.0 Zm00008a.1*. 2019. DOI: 10.25739/DM9S-AA15. URL: http://datacommons.cyverse.org/browse/iplant/home/shared/commons_repo/curated/Carolyn_Lawrence_Dill_GOMAP_maize.PH207.UIUC_UMN-1.0_April_2019.r1.

[45] Candice N. Hirsch et al. "Draft Assembly of Elite Inbred Line PH207 Provides Insights into Genomic and Transcriptome Diversity in Maize". In: *The Plant Cell* 28.11 (Nov. 2016), pp. 2700–2714. DOI: 10.1105/tpc.16.00353. URL: https://doi.org/10.1105/tpc.16.00353.

[46] Kokulapalan Wimalanathan and Carolyn Lawrence-Dill. *GOMAP Maize Zm-W22-REFERENCE-NRGENE-2.0 Zm00004b.1*. 2019. DOI: 10.25739/E4VA-9F09. URL: http://datacommons.cyverse.org/browse/iplant/home/shared/commons_repo/curated/Carolyn_Lawrence_Dill_GOMAP_maize.W22.AGPv2_April_2019.r1.

[47] Nathan M. Springer et al. "The maize W22 genome provides a foundation for functional genomics and transposon biology". In: *Nature Genetics* 50.9 (July 2018), pp. 1282–1288. DOI: 10.1038/s41588-018-0158-0. URL: https://doi.org/10.1038/s41588-018-0158-0.

[48] Kokulapalan Wimalanathan and Carolyn Lawrence-Dill. *maize-GAMER Annotaions for maize.B73.AGPv4.r1*. 2017. DOI: 10.7946/P2M925. URL: http://datacommons.cyverse.org/browse/iplant/home/shared/commons_repo/curated/Carolyn_Lawrence-Dill_maize-GAMER_maize.B73_RefGen_v4_Zm00001d.2_Oct_2017.r1.

[49] Yinping Jiao et al. "Improved maize reference genome with single-molecule technologies". In: *Nature* 546.7659 (June 2017), pp. 524–527. DOI: 10.1038/nature22971. URL: https://doi.org/10.1038/nature22971.

[50] Marcela K Tello-Ruiz et al. "Gramene 2021: harnessing the power of comparative genomics and pathways for plant research". In: *Nucleic Acids Research* 49.D1 (Nov. 2020), pp. D1452–D1463. DOI: 10.1093/nar/gkaa979. URL: https://doi.org/10.1093/nar/gkaa979.

[51] David M. Goodstein et al. "Phytozome: a comparative platform for green plant genomics". In: *Nucleic Acids Research* 40.D1 (Nov. 2011), pp. D1178–D1186. DOI: 10.1093/nar/gkr944. URL: https://doi.org/10.1093/nar/gkr944.

[52] Joseph Felsenstein. *PHYLIP (phylogeny inference package), version 3.5 c.* Joseph Felsenstein., 1993.

[53] Walter M. Fitch. "Toward Defining the Course of Evolution: Minimum Change for a Specific Tree Topology". In: *Systematic Zoology* 20.4 (Dec. 1971), p. 406. DOI: 10.2307/2412116. URL: https://doi.org/10.2307/2412116.

[54] N Saitou and M Nei. "The neighbor-joining method: a new method for reconstructing phylogenetic trees." In: *Molecular Biology and Evolution* (July 1987). DOI: 10.1093/oxfordjournals.molbev.a040454. URL: https://doi.org/10.1093/oxfordjournals.molbev.a040454.

[55] C. F. J. Wu. "Jackknife, Bootstrap and Other Resampling Methods in Regression Analysis". In: *The Annals of Statistics* 14.4 (Dec. 1986). DOI: 10.1214/aos/1176350142. URL: https://doi.org/10.1214/aos/1176350142.

[56] S. C. Potter. "The Ensembl Analysis Pipeline". In: *Genome Research* 14.5 (May 2004), pp. 934–941. DOI: 10.1101/gr.1859804. URL: https://doi.org/10.1101/gr.1859804.

[57] Christophe Dessimoz, Nives Škunca, and Paul D. Thomas. "CAFA and the Open World of protein function predictions". In: *Trends in Genetics* 29.11 (Nov. 2013), pp. 609–610. DOI: 10.1016/j.tig.2013.09.005. URL: https://doi.org/10.1016/j.tig.2013.09.005.

[58] Karin Verspoor et al. "A categorization approach to automated ontological function annotation". In: *Protein Science* (2006). ISSN: 09618368. DOI: 10.1110/ps.062184006. URL: https://dx.doi.org/10.1110%7B%5C%%7D2Fps.062184006.

[59] Michael Defoin-Platel et al. "AIGO: Towards a unified framework for the Analysis and the Inter-comparison of GO functional annotations". In: *BMC Bioinformatics* (2011). ISSN: 14712105. DOI: 10.1186/1471-2105-12-431. URL: https://doi.org/10.1186/1471-2105-12-431.

[60] Ilya Plyusnin, Liisa Holm, and Petri Törönen. "Novel comparison of evaluation metrics for gene ontology classifiers reveals drastic performance differences". In: *PLOS Computational Biology* 15.11 (Nov. 2019). Ed. by Predrag Radivojac, e1007419. ISSN: 1553-7358. DOI: 10.1371/journal.pcbi.1007419. URL: http://dx.plos.org/10.1371/journal.pcbi.1007419.

[61] Ivica Letunic and Peer Bork. "Interactive Tree Of Life (iTOL) v5: an online tool for phylogenetic tree display and annotation". In: *Nucleic Acids Research* (Apr. 2021). DOI: 10.1093/nar/gkab301. URL: https://doi.org/10.1093/nar/gkab301.

[62] Nancy Manchanda et al. "GenomeQC: a quality assessment tool for genome assemblies and gene structure annotations". In: *BMC Genomics* 21.1 (Mar. 2020), p. 193. ISSN: 1471-2164. DOI: 10.1186/s12864-020-6568-2. URL: https://doi.org/10.1186/s12864-020-6568-2 (visited on 03/12/2021).

[63] Felipe A. Simão et al. "BUSCO: assessing genome assembly and annotation completeness with single-copy orthologs". en. In: *Bioinformatics* 31.19 (Oct. 2015), pp. 3210–3212. ISSN: 1367-4803, 1460-2059. DOI: 10.1093/bioinformatics/btv351. URL: https://academic.oup.com/bioinformatics/article-lookup/doi/10.1093/bioinformatics/btv351 (visited on 03/12/2021).

[64] Haley Dostalik and Carolyn Lawrence-Dill. *Carolyn_Lawrence_Dill_GOMAP_Grape_Genoscope_12x_January_2021.r1*. 2021. DOI: 10.25739/JTFK-Q888. URL: http://datacommons.cyverse.org/browse/iplant/home/shared/commons_repo/curated/Carolyn_Lawrence_Dill_GOMAP_Grape_Genoscope_12x_January_2021.r1.

[65] Carolyn Lawrence-Dill. *Carolyn_Lawrence_Dill_GOMAP_Canola_BnPIR_ZS11_March_2021.r1*. 2021. DOI: 10.25739/XGMR-HR31. URL: http://datacommons.cyverse.org/browse/iplant/home/shared/commons_repo/curated/Carolyn_Lawrence_Dill_GOMAP_Canola_BnPIR_ZS11_March_2021.r1.

[66] Leila Fattel and Carolyn Lawrence-Dill. *Carolyn_Lawrence_Dill_GOMAP_Banana_NCBI_ASM31385v2_February_2021.r1*. 2021. DOI: 10.25739/YT7W-GS55. URL: http://datacommons.cyverse.org/browse/iplant/home/shared/commons_repo/curated/Carolyn_Lawrence_Dill_GOMAP_Banana_NCBI_ASM31385v2_February_2021.r1.

[67] Leila Fattel and Carolyn Lawrence-Dill. *Carolyn_Lawrence_Dill_GOMAP_Cacao_NCBI_CriolloV2_March_2021.r1*. 2021. DOI: 10.25739/9QC0-N310. URL: http://datacommons.cyverse.org/browse/iplant/home/shared/commons_repo/curated/Carolyn_Lawrence_Dill_GOMAP_Cacao_NCBI_CriolloV2_March_2021.r1.

[68] Carolyn Lawrence-Dill. *Carolyn_Lawrence_Dill_GOMAP_Coffee_CGH_v1.0_June_2021.r1*. 2021. DOI: 10.25739/RM4J-3580. URL: http://datacommons.cyverse.org/browse/iplant/home/shared/commons_repo/curated/Carolyn_Lawrence_Dill_GOMAP_Coffee_CGH_v1.0_June_2021.r1.

[69] Carolyn Lawrence-Dill. *Carolyn_Lawrence_Dill_GOMAP_Blueberry_GigaDB_v1.0_June*[illegible] 2021. DOI: 10.25739/Q7RQ-E992. URL: http://datacommons.cyverse.org/browse/iplant/home/shared/commons_repo/curated/Carolyn_Lawrence_Dill_GOMAP_Blueberry_GigaDB_v1.0_June_2021.r1.

[70] Dennis Psaroudakis and Carolyn Lawrence-Dill. *Carolyn_Lawrence_Dill_GOMAP_Solanum_lycopersicum_ITAG4.1.v1*[illegible] 2021. DOI: 10.25739/ZH2V-4P15. URL: http://datacommons.cyverse.org/browse/iplant/home/shared/commons_repo/curated/Carolyn_Lawrence_Dill_GOMAP_Solanum_lycopersicum_ITAG4.1.v1_April_2021.r1.

[71] Dennis Psaroudakis and Carolyn Lawrence-Dill. *Carolyn_Lawrence_Dill_GOMAP_Solanum_pennellii_Bolger2014.v1_A*[illegible] 2021. DOI: 10.25739/FHR4-CX67. URL: http://datacommons.cyverse.org/browse/iplant/home/shared/commons_repo/curated/Carolyn_Lawrence_Dill_GOMAP_Solanum_pennellii_Bolger2014.v1_April_2021.r1.

[72] Koichi Sakamoto et al. "Characterization Genome Sizes and Morphology of Sex Chromosomes in Hemp (Cannabis sativa L.)." In: *CYTOLOGIA* 63.4 (1998), pp. 459–464. DOI: 10.1508/cytologia.63.459. URL: https://doi.org/10.1508/cytologia.63.459.

[73] Harm van Bakel et al. "The draft genome and transcriptome of Cannabis sativa". In: *Genome Biology* 12.10 (2011), R102. DOI: 10.1186/gb-2011-12-10-r102. URL: https://doi.org/10.1186/gb-2011-12-10-r102.

[74] Shan Gao et al. "A high-quality reference genome of wild Cannabis sativa". en. In: *Horticulture Research* 7.1 (May 2020). Number: 1 Publisher: Nature Publishing Group, pp. 1–11. ISSN: 2052-7276. DOI: 10.1038/s41438-020-0295-3. URL: https://www.nature.com/articles/s41438-020-0295-3 (visited on 03/12/2021).

[75] W.P. Maddison and D.R. Maddison. *Mesquite: a modular system for evolutionary analysis. Version 3.61*. 2021. URL: http://www.mesquiteproject.org/ (visited on 07/14/2021).

[76] Peifen Zhang et al. "PhyloGenes: An online phylogenetics and functional genomics resource for plant gene function inference". In: *Plant Direct* 4.12 (Dec. 2020). DOI: 10.1002/pld3.293. URL: https://doi.org/10.1002/pld3.293.

[77] Rainer Schwacke et al. "MapMan4: A Refined Protein Classification and Annotation Framework Applicable to Multi-Omics Data Analysis". In: *Molecular Plant* 12.6 (June 2019), pp. 879–892. DOI: 10.1016/j.molp.2019.01.003. URL: https://doi.org/10.1016/j.molp.2019.01.003.

[78] Ivica Letunic and Peer Bork. "Interactive Tree Of Life (iTOL) v4: recent updates and new developments". en. In: *Nucleic Acids Research* 47.W1 (July 2019), W256–W259. ISSN: 0305-1048, 1362-4962. DOI: 10.1093/nar/gkz239. URL: https://academic.oup.com/nar/article/47/W1/W256/5424068 (visited on 03/12/2021).

[79] Elizabeth A. Kellogg. "Evolutionary History of the Grasses". en. In: *Plant Physiology* 125.3 (Mar. 2001), pp. 1198–1205. ISSN: 0032-0889, 1532-2548. DOI: 10.1104/pp.125.3.1198. URL: https://academic.oup.com/plphys/article/125/3/1198-1205/6109905 (visited on 03/12/2021).

[80] Steven B. Cannon and Randy C. Shoemaker. "Evolutionary and comparative analyses of the soybean

genome". eng. In: *Breeding Science* 61.5 (Jan. 2012), pp. 437–444. ISSN: 1344-7610. DOI: 10.1270/jsbbs.61.437.

[81] Candice N. Hansey et al. "Maize (Zea mays L.) genome diversity as revealed by RNA-sequencing". eng. In: *PloS One* 7.3 (2012), e33071. ISSN: 1932-6203. DOI: 10.1371/journal.pone.0033071.

[82] Nasim Azani et al. "A new subfamily classification of the Leguminosae based on a taxonomically comprehensive phylogeny: The Legume Phylogeny Working Group (LPWG)". en. In: *TAXON* 66.1 (2017). _eprint: https://onlinelibrary.wiley.com/doi/pdf/10.12705/661.3, pp. 44–77. ISSN: 1996-8175. DOI: https://doi.org/10.12705/661.3. URL: https://onlinelibrary.wiley.com/doi/abs/10.12705/661.3 (visited on 03/12/2021).

[83] Mark N. Puttick et al. "The Interrelationships of Land Plants and the Nature of the Ancestral Embryophyte". English. In: *Current Biology* 28.5 (Mar. 2018). Publisher: Elsevier, 733–745.e2. ISSN: 0960-9822. DOI: 10.1016/j.cub.2018.01.063. URL: https://www.cell.com/current-biology/abstract/S0960-9822(18)30096-4 (visited on 03/12/2021).

[84] J. Doležel et al. "Letter to the editor". In: *Cytometry* 51A.2 (Jan. 2003), pp. 127–128. DOI: 10.1002/cyto.a.10013. URL: https://doi.org/10.1002/cyto.a.10013.

[85] Mathieu Seppey, Mosè Manni, and Evgeny M. Zdobnov. "BUSCO: Assessing Genome Assembly and Annotation Completeness". In: *Methods in Molecular Biology*. Springer New York, 2019, pp. 227–245. DOI: 10.1007/978-1-4939-9173-0_14. URL: https://doi.org/10.1007/978-1-4939-9173-0_14.

[86] Eva M Temsch and Johann Greilhuber. "Genome size variation in Arachis hypogaea and A. monticola re-evaluated". In: *Genome* 43.3 (June 2000), pp. 449–451. DOI: 10.1139/g99-130. URL: https://doi.org/10.1139/g99-130.

[87] Pilar Catalán et al. "Evolution and taxonomic split of the model grass Brachypodium distachyon". In: *Annals of Botany* 109.2 (Jan. 2012), pp. 385–405. DOI: 10.1093/aob/mcr294. URL: https://doi.org/10.1093/aob/mcr294.

[88] Johann Greilhuber and Renate Obermayer. "Genome size and maturity group in Glycine max (soybean)". In: *Heredity* 78.5 (1997), pp. 547–551. DOI: 10.1038/hdy.1997.85. URL: https://doi.org/10.1038/hdy.1997.85.

[89] B. HENDRIX. "Estimation of the Nuclear DNA Content of Gossypium Species". In: *Annals of Botany* 95.5 (Feb. 2005), pp. 789–797. DOI: 10.1093/aob/mci078. URL: https://doi.org/10.1093/aob/mci078.

[90] Michael David Bennett and JB Smith. "Nuclear DNA amounts in angiosperms". In: *Philosophical Transactions: Biological Sciences* (1991), pp. 309–345. DOI: 10.1098/rstb.1991.0120. URL: https://doi.org/10.1098/rstb.1991.0120.

[91] Fatima Pustahija et al. "Small genomes dominate in plants growing on serpentine soils in West Balkans, an exhaustive study of 8 habitats covering 308 taxa". In: *Plant and soil* 373.1 (2013), pp. 427–453. DOI: 10.1007/s11104-013-1794-x. URL: https://doi.org/10.1007/s11104-013-1794-x.

[92] Ka Arumuganathan and ED Earle. "Nuclear DNA content of some important plant species". In: *Plant molecular biology reporter* 9.3 (1991), pp. 208–218. DOI: 10.1007/BF02672069. URL: https://doi.org/10.1007/BF02672069.

[93] AY Kenton, SJ Owens, and D Langton. "The origin of ringformation and self-compatibility in Gibasis pulchella (Commelinaceae)". In: *Kew Chromosome Conference*. Vol. 3. 1988, pp. 75–84.

[94] DA Laurie and MD Bennett. "Nuclear DNA content in the genera Zea and Sorghum. Intergeneric, interspecific and intraspecific variation". In: *Heredity* 55.3 (1985), pp. 307–313. DOI: 10.1038/hdy.1985.112. URL: https://doi.org/10.1038/hdy.1985.112.

[95] A Parida, SN Raina, and RKJ Narayan. "Quantitative DNA variation between and within chromosome complements of Vigna species (Fabaceae)". In: *Genetica* 82.2 (1990), pp. 125–133. DOI: 10.1007/BF00124642. URL: https://doi.org/10.1007/BF00124642.

Table II: Quantitative metrics of the cleaned functional annotation sets. CC, MF, BP, and A refer to the aspects of the Gene Ontology: Cellular Component, Molecular Function, Biological Process, and Any/All. GOMAP covers all genomes with at least one annotation per gene and provides substantially more annotations than Gramene63 or Phytozome, especially in the BP aspect. The total number of annotations per dataset is visualized in Figure S1.

			Genes Annotated[%][a]				Annotations[b]				Median Ann. per G.[c]			
Genome	Genes	Dataset	CC	MF	BP	**A**	CC	MF	BP	**A**	CC	MF	BP	**A**
Arachis hypogaea	67,124	GOMAP	85.85	84.68	100.00	**100.00**	150,525	132,144	493,145	**775,814**	2	2	6	**10**
Brachypodium distachyon	34,310	GOMAP	81.33	85.35	100.00	**100.00**	74,172	69,213	255,397	**398,782**	2	2	6	**10**
		Gold Standard Gramene 63 (no IEA)	21.54	19.53	18.20	**26.66**	10,985	10,436	11,120	**32,673**	1	1	1	**3**
		Gramene63 (IEA only)	33.12	49.29	38.29	**63.60**	21,658	36,372	23,899	**82,026**	1	1	1	**3**
		Phytozome12	10.25	37.21	26.86	**43.11**	4,186	18,597	11,070	**34,060**	0	1	1	**2**
Cannabis sativa	33,677	GOMAP	94.22	95.48	100.00	**100.00**	85,755	73,614	262,741	**422,110**	2	2	6	**11**
Glycine max	52,872	GOMAP	86.95	88.92	100.00	**100.00**	126,470	113,068	416,989	**656,527**	2	2	6	**11**
Gossypium raimondii	37,505	GOMAP	93.00	92.37	100.00	**100.00**	95,419	84,910	307,470	**487,799**	2	2	6	**11**
Hordeum vulgare	39,734	GOMAP	88.57	91.76	100.00	**100.00**	86,489	79,727	272,420	**438,636**	2	2	5	**10**
		Gold Standard Gramene 63 (no IEA)	28.23	26.30	23.43	**35.64**	15,734	15,391	15,267	**46,414**	1	1	1	**3**
		Gramene63 (IEA only)	36.19	50.90	41.71	**65.03**	29,826	44,789	29,425	**104,178**	1	1	1	**3**
Medicago truncatula A17	50,444	GOMAP	83.79	86.69	100.00	**100.00**	104,902	99,155	363,608	**567,665**	2	2	6	**10**
		Gold Standard Gramene 63 (no IEA)	25.45	23.26	21.51	**32.12**	17,938	18,416	18,461	**54,827**	1	1	1	**3**
		Gramene63 (IEA only)	34.25	50.84	40.26	**66.14**	32,753	63,470	40,441	**137,001**	1	1	1	**3**
		Phytozome12	8.87	36.05	25.83	**41.07**	5,315	25,950	15,576	**47,098**	0	1	1	**2**
Medicago truncatula R108	55,706	GOMAP	72.10	90.14	100.00	**100.00**	108,388	107,499	381,831	**597,718**	1	2	5	**9**
Oryza sativa	35,825	GOMAP	79.78	83.31	100.00	**100.00**	71,306	64,150	248,304	**383,760**	2	2	6	**9**
		Gold Standard Gramene 63 (no IEA)	29.95	27.29	25.33	**37.57**	15,492	15,176	16,536	**47,339**	1	1	1	**3**
		Gramene63 (IEA only)	32.21	45.83	36.75	**60.13**	21,935	37,425	24,255	**83,645**	1	1	1	**3**
		Phytozome12	10.31	40.10	29.18	**46.09**	4,361	20,842	12,451	**37,884**	0	1	1	**2**
Phaseolus vulgaris	27,433	GOMAP	94.48	93.06	100.00	**100.00**	70,987	64,022	229,230	**364,239**	2	2	6	**11**
Pinus lambertiana	31,007	GOMAP	92.67	95.91	100.00	**100.00**	71,247	68,315	212,248	**351,810**	2	2	5	**10**
Sorghum bicolor	34,129	GOMAP	82.44	85.98	100.00	**100.00**	75,145	69,659	259,004	**403,808**	2	2	6	**10**
		Gold Standard Gramene 63 (no IEA)	34.48	32.91	30.90	**42.84**	16,837	17,614	17,850	**52,593**	1	1	1	**3**
		Gramene63 (IEA only)	35.91	52.11	42.36	**67.41**	23,608	39,418	27,074	**90,313**	1	1	1	**3**
		Phytozome12	10.54	39.19	27.90	**45.10**	4,246	19,724	11,432	**35,599**	0	1	1	**2**
Triticum aestivum	107,891	GOMAP	88.53	90.98	100.00	**100.00**	259,318	217,467	785,051	**1,261,836**	2	2	6	**10**
		Gold Standard Gramene 63 (no IEA)	2.98	2.78	2.56	**3.82**	4,727	4,512	4,793	**14,035**	1	1	1	**3**
		Gramene63 (IEA only)	29.12	58.62	38.72	**70.41**	47,595	111,889	62,977	**222,721**	0	1	1	**2**
Vigna unguiculata	29,773	GOMAP	91.21	91.08	100.00	**100.00**	74,791	67,734	242,847	**385,372**	2	2	6	**11**
		Phytozome12	13.91	45.68	34.14	**53.06**	5,107	19,962	12,209	**37,534**	0	1	1	**2**
Zea mays B73.v4	39,324	GOMAP	93.16	94.92	100.00	**100.00**	87,648	81,665	278,305	**447,618**	2	2	6	**10**
		Gold Standard Gramene 63 (no IEA)	37.92	34.78	32.67	**46.85**	22,531	21,292	23,153	**67,285**	1	1	1	**3**
		Gramene63 (IEA only)	39.16	58.16	48.21	**73.87**	30,189	53,748	35,276	**119,273**	1	1	1	**3**
Zea mays Mo17	38,620	GOMAP	86.98	90.87	100.00	**100.00**	86,074	78,650	277,395	**442,119**	2	2	6	**10**
		Gold Standard Gramene 63 (no IEA)	27.56	25.20	23.73	**33.98**	16,128	15,384	16,489	**48,220**	1	1	1	**3**
Zea mays PH207	40,557	GOMAP	86.55	90.61	100.00	**100.00**	88,962	84,910	288,208	**462,080**	2	2	6	**10**
		Gold Standard Gramene 63 (no IEA)	28.18	25.82	24.26	**34.66**	17,370	16,580	17,791	**51,984**	1	1	1	**3**
Zea mays W22	40,690	GOMAP	90.77	92.58	100.00	**100.00**	93,622	84,450	289,364	**467,436**	2	2	6	**10**
		Gold Standard Gramene 63 (no IEA)	25.40	23.15	21.80	**31.29**	15,518	14,818	15,850	**46,402**	1	1	1	**3**

Download this table (CSV)

[a] How many genes in the genome have at least one GO term from the CC, MF, BP aspect annotated to them? A = How many at least one from any aspect? ($A = CC \cup MF \cup BP$)

[b] How many annotations in the CC, MF, and BP aspect does this dataset contain? A = How many in total? $A = CC + MF + BP$

[c] Take a typical gene that is present in the annotation set. How many annotations does it have in each aspect? A = How many in total? Please note that $A \neq CC + MF + BP$

Table III: Qualitative metrics of functional annotation sets predicted by GOMAP, Gramene, and Phytozome. This table is visualized in Figure S2.

		SimGIC2			TC-AUCPCR			Fmax		
Genome	Dataset	CC	MF	BP	CC	MF	BP	CC	MF	BP
Brachypodium distachyon	GOMAP	0.404149	0.464127	0.223830	0.233442	0.230701	0.118526	0.741361	0.740897	0.526881
	Gramene63 (IEA only)	0.317801	0.420859	0.349406	0.129163	0.192507	0.111361	0.691016	0.738542	0.650325
	Phytozome12	0.370264	0.370521	0.352206	0.112582	0.136832	0.085628	0.717759	0.697076	0.660603
Hordeum vulgare	GOMAP	0.400087	0.470012	0.238177	0.237231	0.261399	0.130784	0.745272	0.750213	0.560096
	Gramene63 (IEA only)	0.306119	0.426601	0.381010	0.157352	0.228797	0.136002	0.680996	0.742638	0.665696
Medicago truncatula A17	GOMAP	0.371795	0.451258	0.213407	0.272809	0.282650	0.139032	0.730838	0.726991	0.531406
	Gramene63 (IEA only)	0.329600	0.437274	0.343561	0.176497	0.265887	0.133503	0.701093	0.749900	0.654297
	Phytozome12	0.358311	0.367257	0.363013	0.144247	0.170863	0.110386	0.717307	0.698429	0.661233
Oryza sativa	GOMAP	0.408945	0.482650	0.248207	0.298502	0.303384	0.159724	0.751121	0.757181	0.559221
	Gramene63 (IEA only)	0.328761	0.423191	0.341193	0.167619	0.265410	0.135451	0.711309	0.738732	0.643827
	Phytozome12	0.049975	0.041007	0.044279	0.000003	0.000003	0.000002	0.470134	0.266628	0.239256
Sorghum bicolor	GOMAP	0.404852	0.466708	0.224011	0.316873	0.337380	0.169883	0.746540	0.742001	0.534258
	Gramene63 (IEA only)	0.323037	0.400241	0.353135	0.177038	0.260198	0.154157	0.711107	0.712170	0.653591
	Phytozome12	0.356091	0.348264	0.340124	0.151947	0.177579	0.110483	0.715714	0.675147	0.641535
Triticum aestivum	GOMAP	0.410582	0.489881	0.229271	0.050762	0.030610	0.019360	0.736476	0.762420	0.533897
	Gramene63 (IEA only)	0.362452	0.476685	0.395112	0.040992	0.043701	0.027872	0.737769	0.762059	0.670953
Zea mays B73.v4	GOMAP	0.417455	0.467339	0.245373	0.302761	0.290371	0.153011	0.759504	0.746870	0.564707
	Gramene63 (IEA only)	0.303231	0.416301	0.346308	0.175735	0.250075	0.138275	0.662987	0.732860	0.647725
Zea mays Mo17	GOMAP	0.399521	0.464265	0.225632	0.236209	0.239598	0.125599	0.744360	0.743026	0.537489
Zea mays PH207	GOMAP	0.394481	0.436266	0.224226	0.221709	0.221266	0.117086	0.743111	0.718933	0.533092
Zea mays W22	GOMAP	0.397602	0.463499	0.223511	0.210198	0.217609	0.113262	0.743783	0.742341	0.535572

Download this table (CSV)

Table IV: Assembly statistics*

	C-value (pg)	C-value Ref.	Estimated Genome Size (Mb)	Scaffolds	Total Scaffold Length (Mb)	Total Scaffold Length / Estimated Genome Size (%)	Length Scaffold Sequences >=25K nt (Mb)	Scaffold Sequences >=25K nt / Estimated Genome Size (%)	%N
A. hypogaea	2.87	[86]	2806	384	2560	91.12	2550	91.01	0.15
B. distachyon	0.32	[87]	313	11	272	87.00	272	87.00	0.19
C. sativa	0.84	[72]	821	221	876	106.72	875	106.62	15.93
G. max	1.13	[88]	1105	282	978	88.62	977	88.48	2.65
G. raimondii	0.9	[89]	880	1033	761	86.52	755	85.77	1.75
H. vulgare	7.33	[90, 91]	7167	8	4830	67.45	4830	67.45	5.44
M. truncatula A17	0.47	[92]	460	2186	413	89.93	403	87.77	5.53
M. truncatula R108	0.47	[92]	460	909	402	87.60	395	86.09	0.68
O. sativa	0.5	[90]	489	63	375	76.85	375	76.74	0.03
P. vulgaris	0.6	[93]	587	478	537	91.68	534	91.12	1.05
S. bicolor	1.2	[94]	1173	870	709	60.43	705	60.08	4.72
T. aestivum	17.3	[90]	16916	22	14500	86.00	14500	86.00	1.90
V. unguiculata	0.6	[95]	587	686	519	88.64	518	88.38	0.00
Z. mays B73	2.7	[90]	2640	266	2130	80.85	2130	80.85	1.44
Z. mays Mo17	2.7	[90]	2640	2208	2180	82.68	2170	82.06	1.61
Z. mays PH207	2.7	[90]	2640	43291	2160	81.67	2090	79.25	19.61
Z. mays W22	2.7	[90]	2640	11	213E0	80.83	2130	80.83	1.90

Download this table (CSV)

* The extended assembly statistics are found in Table S1.

Table V: Structural annotation table directly from GenomeQC.

	Gene Models	Min. Gene Length (bp)	Max. Gene Length (bp)	Avg. Gene Length (bp)	Gene Models <200 bp	Transcripts	Avg. Transcripts per Gene Model	Exons	Avg. Exons per Transcript	Avg. Exon Length (bp)
A. hypogaea	67128	163	342359	3972.3	48	84714	1.3	423763	4.8	296.4
B. distachyon	36647	90	47411	3012.5	175	47917	1.3	263865	5.5	314.1
C. sativa	29815	21	976063	3450.0	2187	34876	1.2	234157	6.6	291.9
G. max	52872	90	94733	4017.0	1363	86256	1.6	-	-	-
G. raimondii	37505	90	51175	3243.5	71	77267	2.1	527563	6.7	271.3
H. vulgare	-	-	-	-	-	248270	-	1715898	-	278.9
M. truncatula A17	51541	60	102191	2566.9	1985	74213	1.4	318421	4.4	313.2
M. truncatula R108	55706	72	62996	2232.4	2404	61019	1.1	220904	3.6	270.6
O. sativa	35825	72	304271	3098.7	57	42378	1.2	192499	4.5	350.3
P. vulgaris	27433	93	90772	3943.8	108	36995	1.3	-	-	-
S. bicolor	34129	96	88337	3713.2	56	47121	1.4	266301	5.6	350.9
T. aestivum	107891	54	124945	3488.9	686	133744	1.2	749233	5.8	303.3
V. unguiculata	29773	90	81066	3880.9	408	42287	1.4	-	-	-
Z. mays B73	39324	111	128402	4117.8	38	151959	3.9	1488381	9.7	281.5
Z. mays Mo17	38620	21	146217	4076.8	1225	46530	1.2	272323	5.9	297.1
Z. mays PH207	40557	111	714207	5993.3	53	40557	1.0	208149	5.1	252.1
Z. mays W22	40691	87	154495	4330.4	64	51717	1.3	313830	6.1	292.4

Download this table (CSV)

Dashes (-) represent missing data.

Missing data in *H. vulgare* is due to the absence of gene model information in the GFF file.

Missing data in *G. max*, *P. vulgaris*, and *V. unguiculata* is due to the absence of exon information in their GFF files.

IX. SUPPLEMENTARY

Supplementary Table SI: Additional assembly statistics from GenomeQC.

	Longest Scaffold (bp)	Shortest Scaffold (bp)	Scaffolds >1K nt	Scaffolds >10K nt	Scaffolds >100K nt	Scaffolds >1M nt	Scaffolds >10M nt	N50	L50	NG50	LG50	%A	%C	%G	%T
A. hypogaea	1.61E+08	1000	382	299	42	23	20	1.35E+08	9	1.35E+08	10	31.79	18.14	18.13	31.79
B. distachyon	7.55E+07	2.88E+04	11	11	5	5	5	5.89E+07	3	5.89E+07	3	26.76	23.16	23.14	26.76
C. sativa	1.05E+08	6741	221	218	52	15	10	9.19E+07	5	9.19E+07	5	27.94	14.07	14.11	27.95
G. max	5.83E+07	885	281	271	63	20	20	4.99E+07	10	4.78E+07	11	31.77	16.91	16.91	31.77
G. raimondii	7.07E+07	1001	1033	200	20	14	13	6.22E+07	6	6.10E+07	7	32.81	16.32	16.32	32.81
H. vulgare	7.68E+08	2.50E+08	8	8	8	8	8	6.57E+08	4	5.83E+08	6	26.27	21.02	21.01	26.26
M. truncatula A17	5.66E+07	1004	2186	605	51	8	8	4.92E+07	4	4.57E+07	5	31.60	15.66	15.65	31.57
M. truncatula R108	3.23E+07	1007	909	481	72	49	18	1.28E+07	12	1.23E+07	14	33.25	16.41	16.41	33.25
O. sativa	4.33E+07	4236	63	51	17	12	12	3.00E+07	6	2.90E+07	8	28.21	21.77	21.78	28.21
P. vulgaris	6.30E+07	1005	478	290	61	13	11	4.97E+07	5	4.80E+07	6	31.84	17.61	17.64	31.86
S. bicolor	8.09E+07	1005	870	232	69	12	10	6.87E+07	5	6.87E+07	9	26.74	20.90	20.91	26.73
T. aestivum	8.31E+08	4.74E+08	22	22	22	22	22	7.10E+08	10	6.74E+08	12	26.46	22.59	22.59	26.46
V. unguiculata	6.53E+07	2922	686	679	103	13	11	4.17E+07	6	4.13E+07	7	33.31	16.40	16.42	33.34
Z. mays B73	3.07E+08	5568	266	261	48	12	10	2.24E+08	5	1.82E+08	6	26.18	23.09	23.10	26.19
Z. mays Mo17	3.06E+08	1007	2208	1864	129	11	10	2.20E+08	5	1.83E+08	6	26.14	23.03	23.05	26.17
Z. mays PH207	3.03E+08	500	16840	1115	61	11	10	2.15E+08	5	1.76E+08	6	21.34	18.40	18.40	21.36
Z. mays W22	3.11E+08	1.25E+07	11	11	11	11	11	2.23E+08	5	1.83E+08	6	26.11	22.92	22.94	26.13

Download this table (CSV)

N50: The length of the shortest scaffold/contig in the list of L50 sequences.

L50: The number of sequences whose sum of lengths make up 50% or more of the total assembly length.

NG50: The length of the shortest scaffold/contig calculated in the same manner as N50 but based on estimated genome size rather than total assembly length.

LG50: The number of sequences whose sum of lengths make up 50% or more of the estimated genome size.

Supplementary Table SII: Number of removed annotations during cleanup.

Genome	Dataset	Obsolete Annotations	Duplicates	Annotations with Modifiers
Arachis hypogaea	GOMAP	3437	13	912
Brachypodium distachyon	GOMAP	2512	49	789
	Gold Standard Gramene 63 (no IEA)	21	204	0
	Gramene63 (IEA only)	166	114	0
	Phytozome12	99	18	0
Cannabis sativa	GOMAP	1714	6	757
Glycine max	GOMAP	3333	10	930
Gossypium raimondii	GOMAP	1781	7	822
Hordeum vulgare	GOMAP	1877	8	815
	Gold Standard Gramene 63 (no IEA)	1	9	0
	Gramene63 (IEA only)	282	147	0
Medicago truncatula A17	GOMAP	2673	10	798
	Gold Standard Gramene 63 (no IEA)	2	23	0
	Gramene63 (IEA only)	309	243	0
	Phytozome12	132	17	0
Medicago truncatula R108	GOMAP	4168	7	803
Oryza sativa	GOMAP	1642	7	869
	Gold Standard Gramene 63 (no IEA)	37	833	0
	Gramene63 (IEA only)	238	64	0
	Phytozome12	119	19	0
Phaseolus vulgaris	GOMAP	1190	6	783
Pinus lambertiana	GOMAP	1839	4	587
Sorghum bicolor	GOMAP	2384	66	783
	Gold Standard Gramene 63 (no IEA)	178	219	0
	Gramene63 (IEA only)	278	198	0
	Phytozome12	131	12	0
Triticum aestivum	GOMAP	9624	17	1132
	Gold Standard Gramene 63 (no IEA)	1	5	0
	Gramene63 (IEA only)	584	319	0
Vigna unguiculata	GOMAP	1269	6	811
	Phytozome12	122	27	0
Zea mays B73.v4	GOMAP	2077	89	848
	Gold Standard Gramene 63 (no IEA)	50	633	0
	Gramene63 (IEA only)	306	140	0
Zea mays Mo17	GOMAP	2346	83	823
	Gold Standard Gramene 63 (no IEA)	36	1489	0
Zea mays PH207	GOMAP	2676	82	830
	Gold Standard Gramene 63 (no IEA)	37	2702	0
Zea mays W22	GOMAP	2681	88	840
	Gold Standard Gramene 63 (no IEA)	30	499	0

Download this table (CSV)

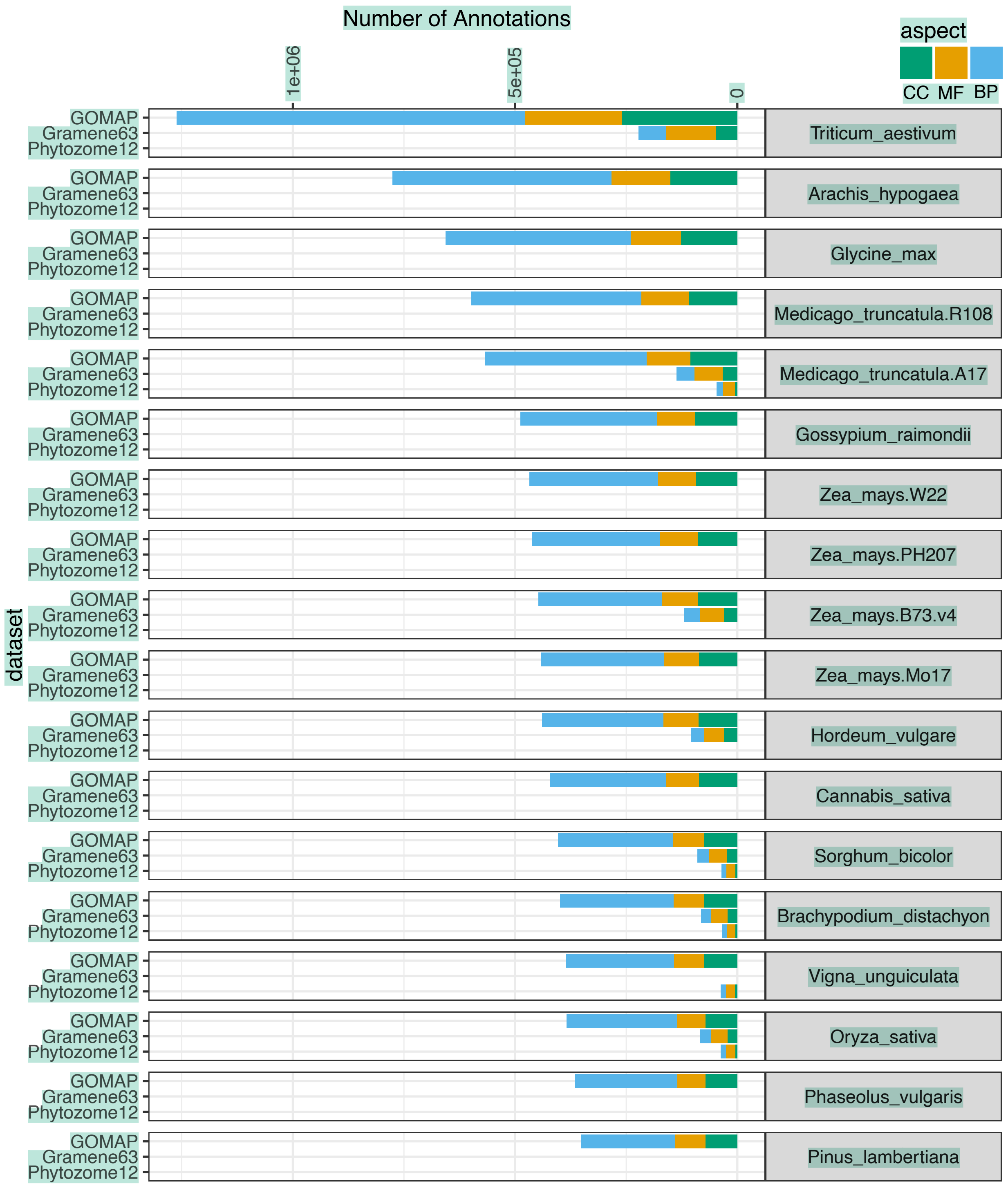

Supplementary Figure S1: Number of total annotations in each GO IEA dataset analyzed, colored by GO aspect (Cellular Component in green, Molecular Function in orange, and Biological Process in blue). Species are ordered based on the number of GO terms in the GOMAP dataset, with the species producing the most GO terms (*Triticum*) on top and the least (*Pinus*) on bottom.

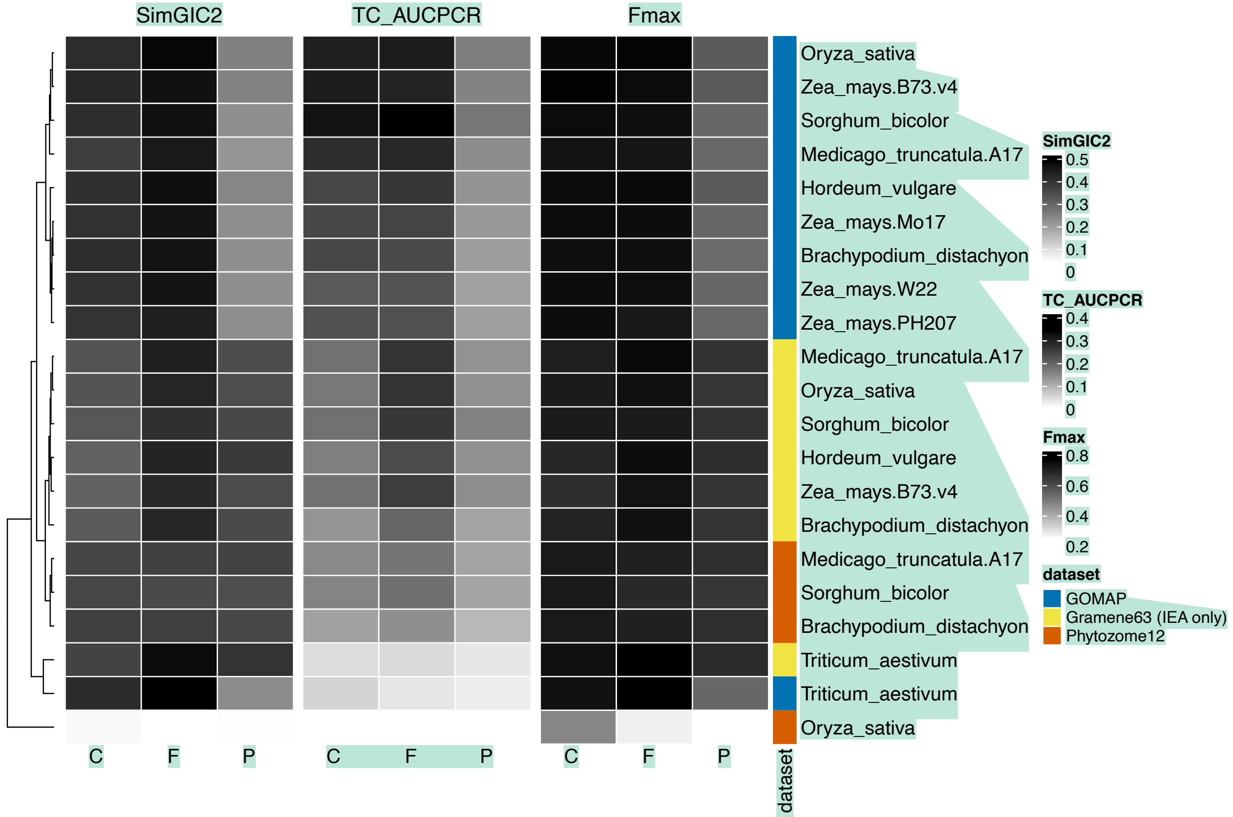

Supplementary Figure S2: Quality scores of the predicted annotation visualized as a grayscale heatmap. Each row represents a single species from a single data source. Input gene model dataset origin is indicated - blue for GOMAP, yellow for Gramene63, and orange for Phytozome12. Across the top are the three metric types used to assess the annotation quality. Across the bottom are subgraph indicators: C is Cellular Component, F is Molecular Function, and P is Biological Process. Darker cells indicate a higher (better) score whereas lighter cells indicate a lower score. Note that scales are different for each metric type (meaning that comparisons across the three metric types are not meaningful). Rows are clustered by pairwise correlation/similarity across all metrics. The dendrogram at left retraces clustering.

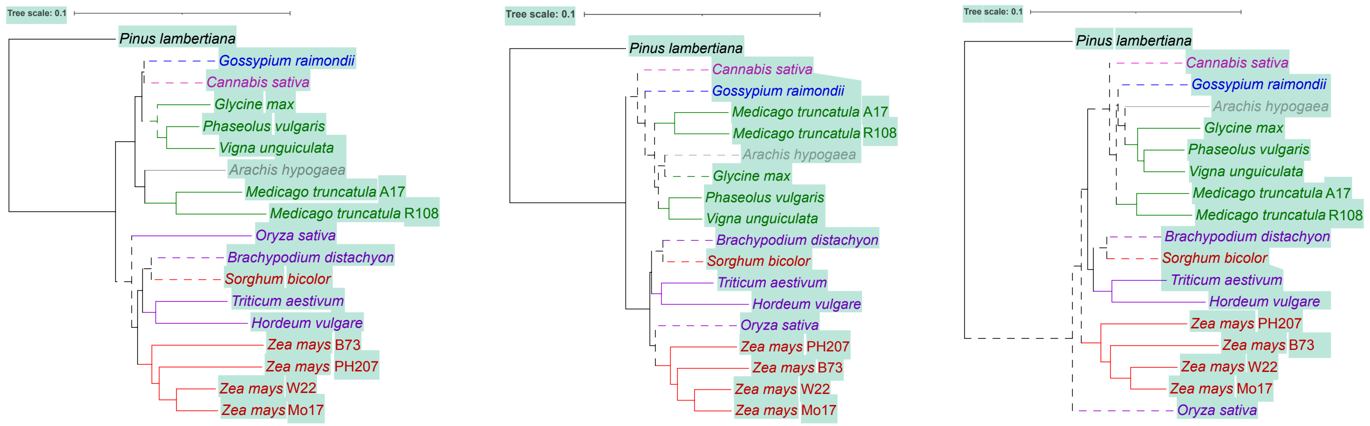

(a) Cellular Component (b) Molecular Function (c) Biological Process

Supplementary Figure S3: Neighbor-joining trees built on annotation subsets per GO aspect. Phylograms are colored and rooted as described in Figure 2. For each tree, only the annotations from the respective aspect of the Gene Ontology were used.

Clean pdf of the revised manuscript

Click here to access/download;Manuscript;Comparative_Functional_Genom

Standardized genome-wide function prediction enables comparative functional genomics: a new application area for Gene Ontologies in plants

Leila Fattel[1,*], Dennis Psaroudakis[2,*,†], Colleen F. Yanarella[1], Kevin O. Chiteri[1,‡], Haley A. Dostalik[1,‡], Parnal Joshi[4,‡], Dollye C. Starr[1,‡], Ha Vu[5,‡], Kokulapalan Wimalanathan[5,§¶], and Carolyn J. Lawrence-Dill[1,5,**]

[1] *Department of Agronomy, Iowa State University*

[2] *Department of Plant Pathology and Microbiology, Iowa State University*

[3] *Department of Ecology, Evolution and Organismal Biology, Iowa State University*

[4] *Department of Veterinary Microbiology and Preventative Medicine, Iowa State University*

[5] *Department of Genetics, Development and Cell Biology, Iowa State University*

(Dated: December 28, 2021)

Background Genome-wide gene function annotations are useful for hypothesis generation and for prioritizing candidate genes potentially responsible for phenotypes of interest. We functionally annotated the genes of 18 crop plant genomes across 14 species using the GOMAP pipeline.

Results By comparison to existing GO annotation datasets, GOMAP-generated datasets cover more genes, contain more GO terms, and produce datasets similar in quality (based on precision and recall metrics using existing gold standards as the basis for comparison). From there, we sought to determine whether the datasets across multiple species could be used together to carry out comparative functional genomics analyses in plants. To test the idea and as a proof of concept, we created dendrograms of functional relatedness based on terms assigned for all 18 genomes. These dendrograms were compared to well-established species-level evolutionary phylogenies to determine whether trees derived were in agreement with known evolutionary relationships, which they largely are. Where discrepancies were observed, we determined branch support based on jack-knifing then removed individual annotation sets by genome to identify the annotation sets causing unexpected relationships.

Conclusions GOMAP-derived functional annotations used together across multiple species generally retain sufficient biological signal to recover known phylogenetic relationships based on genome-wide functional similarities, indicating that comparative functional genomics across species based on GO data hold promise for generating novel hypotheses about comparative gene function and traits.

Keywords: Gene function; ontology; plants; comparative genomics; functional genomics

I. BACKGROUND

Phenotypes and traits have long been the primary inspiration for biological investigation. Phenotypes are the result of a complex interplay between functions of genes and environmental cues. In an effort to organize and model gene functions, various systems of classification have been developed including systems like KEGG (the Kyoto Encyclopedia of Genes and Genomes), which is focused on protein function including gene activities superimposed on metabolic pathways [1]. Other such systems include the various Cyc databases, MapMan, and the Gene Ontologies (GO), a vocabulary of gene functions organized as a directed acyclic graph, which makes it innately tractable for computational analysis [2, 3, 4].

GO-based gene function annotation involves the association of GO terms to individual genes. Functions may be assigned to genes based on different types of evidence for the association. For example, functional predictions can be inferred from experiments (EXP), expression patterns (IEP), and more [5]. Computational pipelines are often used to generate functional predictions for newly sequenced genomes, where the genome is first sequenced and assembled, then gene structures (gene models) are predicted, then functions are associated with those gene predictions. Genome-wide gene function prediction datasets are frequently used to analyze gene expression studies, to prioritize candidate genes linked to a phenotype of interest, to design experiments aimed at characterizing functions of genes, and more [6, 7, 8]. How well a gene function prediction set models reality is influenced by how complete and correct the underlying genome assembly and gene structure annotations are as well as by how well the software used to predict functions performs.

GOMAP (the Gene Ontology Meta Annotator for Plants) is a gene function prediction pipeline for plants that generates high-coverage and reproducible functional annotations [9]. The system employs multiple functional prediction approaches, including sequence similarity, protein domain presence, and mixed-method pipelines developed to compete in the Critical Assessment of Function Annotation (CAFA) Challenge [10], a community challenge that has advanced the performance of gene function prediction pipelines over the course of five organized competitions [11].

*Contributed equally.

†Current address: Department of Molecular Biology, Leibniz Institute of Plant Genetics and Crop Plant Research (IPK), 06466 Seeland, OT Gatersleben, Germany

‡Listed alphabetically.

§kokul@bioinformapping.com

¶Current address: Greenlight Biosciences Inc., Medford, MA, 02155, USA

**triffid@iastate.edu

We previously annotated gene functions for the maize B73 genome and demonstrated that GOMAP's predicted functions were closer to curated gene-term associations from the literature than those of other community functional annotation datasets, including those produced by Gramene (Ensembl pipeline) and Phytozome (Interpro2GO pipeline) [12]. Using the newly containerized GOMAP system [9], we report here the functional annotation of 18 plant genomes across the 14 crop plant species shown in Table I and report comparisons of performance based on comparison to a Gold Standard gene function datasets, where possible.

Given these multiple annotations across various plant species, we next considered whether these datasets could be used together for comparative functional genomics in plants. We describe here a simple and crude method by which we used gene function annotations to generate dendrograms of genome-level similarity in function. This idea is similar to that of Zhu et al., who determined the evolutionary relationships among microorganisms based on whole-genome functional similarity [13]. Here we expand on that approach, analyzing genome-wide GO assignments to generate parsimony and distance-based dendrograms (see Figure 1 for process overview). We compared these with well-established species phylogenies (Figure 2) to determine whether trees derived from gene function show any agreement with evolutionary histories, taking agreement between generated dendrograms and known evolutionary histories to be evidence that sufficient comparative biological signal exists to begin to use GO functional annotations across multiple plant genomes for comparative functional genomics investigations.

II. RESULTS OF ANALYSES

A. Overview

As shown in Figure 1, gene function annotation sets were created and compiled for each genome. For those with existing annotation sets available on Gramene or Phytozome [50, 51], the datasets were compared. From there, matrices that included genomes as rows and terms as columns were generated. These were used directly to build parsimony trees or to create distance matrices for neighbor-joining tree construction [52, 53, 54]. In subsequent analyses, jackknifing was used to remove terms (columns) or to remove genomes (rows) to map the source of signal for treebuilding results [55].

B. Functional Annotation Sets Produced

Table II shows quantitative attributes of each of the annotation sets. In summary, GOMAP covers all annotated genomes with at least one annotation per gene, and provides between 3.8 and 12.1 times as many annotations as Gramene or Phytozome.

Quality evaluation of gene function predictions is not trivial and is approached by different research groups in different ways. Most often datasets are assessed by comparing the set of predicted functions for a given gene to a Gold Standard consisting of annotations that are assumed to be correct. This assumption of correctness can be based on any number of criteria. Here we used as our Gold Standard dataset all annotations present in Gramene63 that had a non-IEA (non-Inferred by Electronic Annotation) evidence code, i.e. we used only annotations that had some manual curation. This enabled us to assess the 10 genomes shown in Table II. It is perhaps noteworthy that the IEA and non-IEA annotation sets from Gramene63 frequently contain overlaps, indicating that some of the predicted annotations were manually confirmed afterwards by a curator and that in such cases, a new annotation was asserted with the new evidence code rather than simply upgrading the evidence code from IEA to some other code, thus preserving the IEA annotations in Gramene63 that are produced by the Ensembl analysis pipeline [56], a requirement for comparing GOMAP-produced IEA datasets to the IEA datasets produced by the Ensembl pipeline.

A general limitation of using Gold Standards for quality evaluation is that they can never be assumed to be complete, and therefore false positives in the prediction cannot be distinguished from false negatives in the Gold Standard. In other words, is gene X, function Y truly a wrong prediction or has it simply not yet been discovered experimentally? This problem is laid out in more detail in [57]. As a consequence, the quality of larger prediction sets will be systematically underestimated compared to smaller ones, and this effect is exacerbated the more incomplete the Gold Standard is.

There are many different metrics that have been used to evaluate the quality of predicted functional annotations. For the maize B73 GOMAP annotation assessment in [12], we had used a modified version of the hierarchical evaluation metrics originally introduced in [58] because they were simple, clear, and part of an earlier attempt at unifying and standardizing GO annotation comparisons [59]. In the meantime, Plyusnin et al. published an approach for evaluating different metrics showing variation among the robustness of different approaches to quality assessment [60]. Based on their recommendations, we use here the SimGIC2 and Term-centric Area Under Precision-Recall Curve (TC-AUCPCR) metrics. We also evaluated with the F_{max} metric, simply because it is widely-used (e.g., by [10]), even though according to Plyusnin et al., it is actually a flawed metric [60]. Results of the quality assessments for the 10 genomes where a Gold Standard was available are shown in Table III and Figure S2. While evaluation values differ between metrics and the scores are not directly comparable, a few consistent patterns emerge: GOMAP annotations are almost always better than Gramene and Phytozome annotations in the Cellular Component and Molecular Function aspect, with the only three exceptions being the Molecular

Table I: Functional annotation sets generated by GOMAP. More information about each dataset including the source of the input to GOMAP can be found at the respective DOI.

Species	Germplasm/Line	Assembly/Annotation	Dataset DOI	Genome Reference
Arachis hypogaea	Tifrunner	Arachis hypogaea assembly 1.0	[14]	[15]
Brachypodium distachyon	Bd21	Bd21.v3.1.r1	[16]	[17]
Cannabis sativa	Hemp	NCBI Cannabis sativa GCA_900626175.1	[18]	[19]
Glycine max	Williams 82	Joint Genome Institute (JGI) Wm82.a4.v1	[20]	[21]
Gossypium raimondii	Cotton D	Gossypium raimondii JGI v2.1	[22]	[23]
Hordeum vulgare	–	IBSC_PGSB_r1	[24]	[25]
Medicago truncatula	R108_HM340	R108: v1.0	[26]	[27]
Medicago truncatula	A17_HM341	Mt4.0v2	[28]	[29]
Oryza sativa	Japonica	IRGSP 1.0	[30]	[31]
Phaseolus vulgaris	G19833	DOE-JGI and USDA-NIFA annotation 2.0	[32]	[33]
Pinus lambertiana	Sugar Pine	TreeGenesDB sugar pine assembly v1.5	[34]	[35]
Sorghum bicolor	BTx623	BTx623.v3.0.1.r1	[36]	[37]
Triticum aestivum	Chinese Spring	IWGSC RefSeq 1.1	[38]	[39]
Vigna unguiculata	IT97K-499-35	JGI annotation v1.1	[40]	[41]
Zea mays *	Mo17	Zm-Mo17-REFERENCE-CAU-1.0	[42]	[43]
Zea mays *	PH207	Zm-PH207-REFERENCE_NS-UIUC_UMN-1.0	[44]	[45]
Zea mays *	W22	Zm-W22-REFERENCE-NRGENE-2.0 Zm00004b.1	[46]	[47]
Zea mays *	B73	RefGen_V4 Zm00001d.2	[48]	[49]

Latest overview at https://dill-picl.org/projects/gomap/gomap-datasets/
* Previously published in [9].

Function aspect for *T. aestivum* using the TC-AUCPCR and the F_{max} metric and the Cellular Component aspect for *M. truncatula* A17 using the F_{max} metric. Conversely, GOMAP predictions achieve consistently lower quality scores in the Biological Process aspect with the exception of *B. dystachion*, *O. sativa*, and *S. bicolor* with the TC-AUCPR metric. Generally, annotations that are better in one aspect are also better in the other two aspects but the ranking of annotations does not necessarily hold across metrics. The Phytozome annotation for *O. sativa* is an outlier in terms of its comparative quality, potentially because it is based on a modified structural annotation that differs substantially from the Gold Standard and the other annotations under comparison.

C. Phylogenetic Tree Analyses

With the comparative quality of gene function predictions in hand, we approached the question of whether the datasets could be used together for comparative functional analysis across all genomes. As a simple first step, we began to work toward understanding the degree to which trees built based on gene functions agree with known, well-documented evolutionary relatedness. We constructed neighbor-joining and parsimony trees of the 18 plant genomes, and visilized them using iTOL [61]. The two tree topologies, rooted at *P. lambertiana*, were compared to one another and to the topology of the expected tree (Figure 2). For both the neighbor-joining (Figure 3a) and parsimony trees (Figure 3b), one common difference is noted: *S. bicolor* is not at the base of the *Z. mays* clade as expected, and is clustered with *B. distachyon* instead. Notable differences between the neighbor-joining and parsimony tree are the following: *C. sativa* appears at the base of the eudicots instead of *G. raimondii* in the neighbor-joining tree, while *G. raimondii* is grouped with *C. sativa* and *A. hypogaea* is grouped with *G. max* in the parsimony tree. Second, *O. sativa* was expected to be at the base of the BOP clade, but appears at the base of *Z. mays* in the neighbor-joining tree, and at the base of all angiosperms in the parsimony tree. Differences among relationships within the *Z. mays* clade constaining B73, PH207, W22, and Mo17 were disregarded given the high degree of similarity across annotation sets and the fact that these relationships are not clear given the complex nature of within-species relationships.

Due to differences between the function-based dendrograms and the expected tree, jackknifing analysis was carried out by removing terms (columns in underlying datasets) to determine the degree to which the underlying datasets support specific groupings based on functional term assignments. This analysis was carried out for both neighbor-joining and parsimony trees. First, trees were generated by omitting 5% to 95% of the dataset in increments of 5 to determine the threshold at which the tree topologies deviated from those generated using the full dataset. That threshold was reached at 45% for both neighbor-joining and parsimony; therefore, we used trees generated with 40% of the data removed for reporting branch support values for the topology (Figure 3). Comparing the two trees, the parsimony topology was not as solid as that of the neighbor-joining at jackknife values up to 40%. Based on this robustness for neighbor-joining treebuilding in general, we carried out all subsequent analyses using neighbor-joining treebuilding methods.

We considered investigating the effect of using one

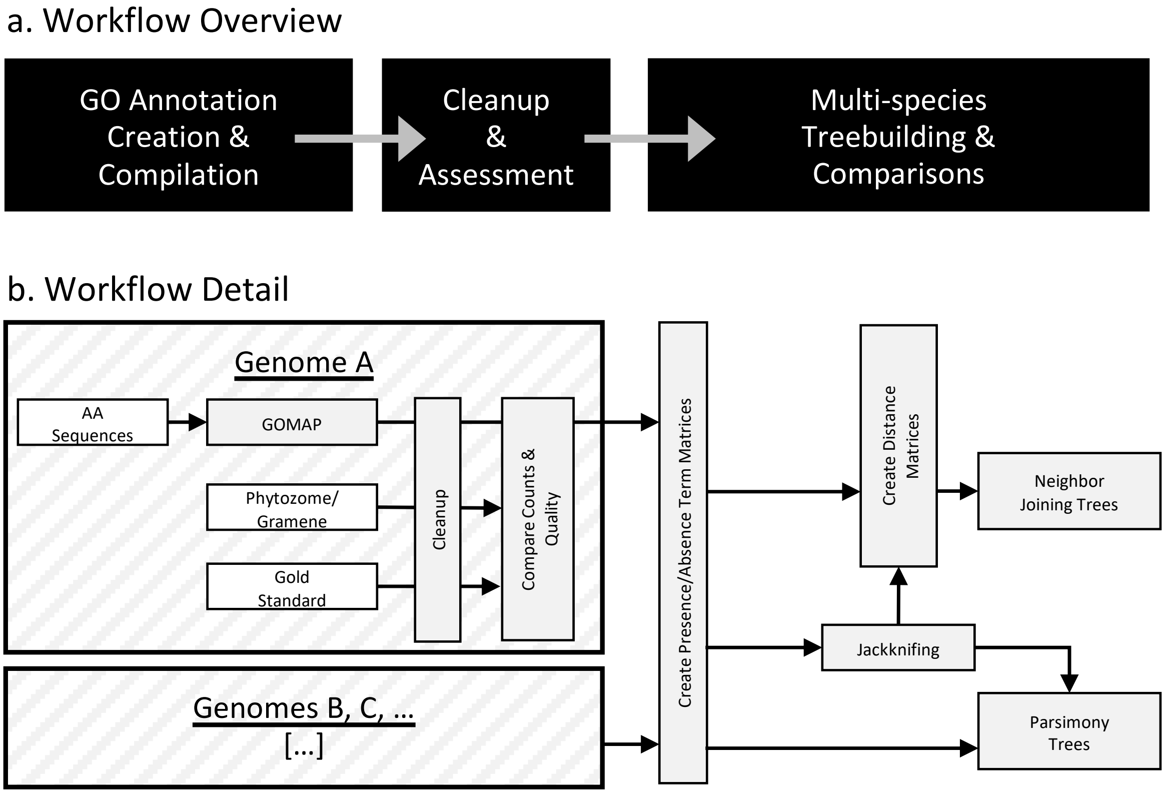

Figure 1: Data workflow schema. The workflow overview is shown in panel ‘a’ with steps represented as black boxes and the flow of information and processes indicated by arrows. Details are shown in panel ‘b’ where the upper large hatched box shows process detail for a single genome and the lower hatched box represents additional genomes for which the details of processing are identical. White boxes represent input datasets. Arrows indicate the flow of information and processes.

GO aspect to generate our neighbor-joining tree. In other words, we generated the neighbor-joining trees using cellular component GO terms, molecular function GO terms, and biological process GO terms separately (Supplementary Figure 3). Of the 14,303 total GO terms, 1,524 are cellular component terms, 3,926 are molecular function terms, and 8,853 are biological process terms. Out of the three single aspect phylogenetic trees, the one built using molecular function terms is the closest to our neighbor-joining tree obtained using all GO terms in our datasets Figure 3a. The only difference is that *A. hypogaea* and *G. max* are clustered in the molecular function tree, while they are not in our neighbor-joining tree Figure 3a. In the cellular component tree, *G. raimondii* and *C. sativa* are clustered together when they are not in the neighbor-joining tree with all GO aspects Figure 3a. Also, *O. sativa* is at the base of the monocots just like in the expected tree, but not in the neighbor-joining tree Figure 3a. In the biological process tree, *O. sativa* is at the base of the angiosperms and there is no clear separation between monocots and dicots. In all the three single aspect phylogenetic trees and our all-aspect neighbor-joining tree, *A. hypogaea* is never placed at the base of the NPAAA clade. Also, *B. distachyon* and *S. bicolor* are always clustered together. Overall, the topologies constructed using one GO term aspect at a time are close to that of our neighbor-joining tree, such that not one GO term aspect alone restored the topology of the expected tree.

To map the source of discrepancies to specific gene annotation sets, we generated various neighbor-joining trees excluding one genome each time, an additional tree with both *Medicago* genomes excluded simultaneously, and another with all *Z. mays* genomes excluded simultaneously. To exemplify this, see the monocot clade in Figure 2 and the lower (monocot) clade in Figure 3a. When the neighbor-joining tree was generated, two species are misplaced: *S. bicolor* and *O. sativa*. As shown in Figure 5a, removal of *O. sativa* corrects one error (itself) but does not correct the errant grouping of *S. bicolor* with *B. distachyon*. In Figure 5b, it is shown that the removal of *S. bicolor* corrects the errant grouping of itself and *B. distachyon*, but *O. sativa* placement remains incorrect. However, as shown in Figure 5c, the removal of *B. distachyon* generates a tree where all relationships are in agreement with known species-level relationships. (Note well: all individual annotation sets were progressively removed, not just the three shown in the example.)

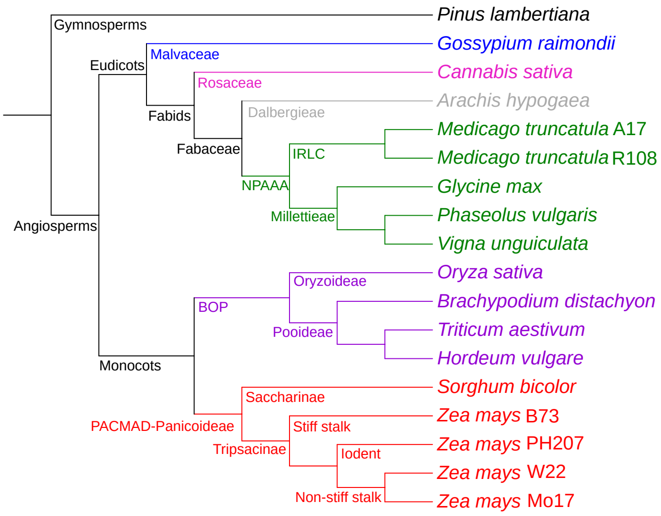

Figure 2: Known phylogenetic relationships among species. Cladogram is rooted by the gymnosperm *Pinus lambertiana* (black). Among angiosperms, eudicots clades include Malvaceae (blue), Rosaceae (magenta), Dalbergieae (grey), and NPAAA (green). Monocots include members of the BOP (purple) and PACMAD-Panicoideae (red) clades.

With this observation in hand, we sought to determine the minimum number of genomes that could be removed to create a tree that matched the expected tree topology. All possible combinations of removing 0-5 genomes to restore the topology were tested, and 10 combinations of minimum amount of genomes to be removed were obtained. The removal of four genomes was required to generate function-based trees consistent with known phylogenetic relationships. Of the 10, we selected the one that had the genomes that were most frequently part of a solution (*O. sativa*, 8; *B. distachyon*, 7; *C. sativa*, 6; *A. hypogaea*, 5; *S. bicolor*, 4; *G. raimondii*, 4; *G. max*, 4; *T. aestivum*) to show in this paper (the other combinations can be found in our publicly available dataset). To elaborate, the genomes removed here are *O. sativa*, *B. distachyon*, *C. sativa*, and *A. hypogaea* (Figure 4). Jackknifing analysis was also carried out for this dataset with support shown. Branch support is generally higher than that for the full dataset (i.e., branch support is higher in Figure 4 than in Figure 3a), and removing genomes that are causing variations seem to stabilize the tree.

D. Potential Causes of Unexpected Groupings

As a first step toward explaining discrepancies between known evolutionary relationships and those resulting from comparative analysis of genome-wide gene function predictions, we assessed the quality of each genome assembly and structural annotation set using GenomeQC [62]. Tables IV-V and Figures 6-7 represent the resulting assembly quality, structural annotation measures of quality, and proportion of single-copy BUSCOs (Benchmarking Universal Single-Copy genes) [63] that were generated. Although these analyses make evident that the species annotated are comparatively different in both natural genome characteristics and in assembly and annotation quality aspects, it is not the case that the four species responsible for deviations between the functional annotation dendrograms and known phylogenetic relationships (i.e., *C. sativa*, *A. hypogaea*, *O. sativa* and *B. distachyon*) create these discrepancies due to issues of genome assembly and/or annotation quality. One potential for some explanation is in relation to *C. sativa*, which is the only genome that has an assembly length larger than the expected (see IV), and a comparatively large proportion of missing BUSCOs in the assembly (see Figure 6). Similarly, for *A. hypogaea* and *O. sativa*, there is a large proportion of missing BUSCOs in the annotations (see Figure 7).

III. DISCUSSION

In this study, we used the GOMAP pipeline to produce whole-genome GO annotations for 18 genome assembly and annotation sets from 14 plant species [9]. Assessments of the number of terms predicted as well as the quality of predictions indicate that GOMAP functional prediction datasets cover more genes, contain more predictions per gene, and are of similar quality to prediction datasets produced by other systems, thus supporting the notion that these high-coverage datasets are a useful addition for researchers who are interested in genome-level analyses, including efforts aimed at prioritizing candidate genes for downstream analyses. Given that we can now produce high-quality, whole genome functional annotations for plants in a straightforward way, we intend to produce more of these over time (indeed we recently annotated *Vitis vinifera* [64], *Brassica rapa* [65], *Musa acuminata*[66], *Theobroma cacao* [67], *Coffea canephora* [68], *Vaccinium corymbosum* [69] *Solanum lycopersicum* [70], and *Solanum pennellii* [71]).

With 18 genome functional annotations in hand, we sought to determine whether and how researchers could use multispecies GO annotation datasets to perform comparative functional genomics analyses. As a proof of concept, we adapted phylogenetic tree-building methods to use the gene function terms assigned to genes represented by the genomes to build dendrograms of functional relatedness and hypothesized that if the functions were comparable across species, the resulting trees would closely match evolutionary relationships. To our delight and surprise, the neighbor-joining and parsimony trees (Figure 3) did resemble known phylogenies, but were not exact matches to broadly accepted phylogenetic relationships.

After removing the minimum number of genomes that resulted in restoration of the expected evolutionary relationships, we found that the individual species that may be responsible for the discrepancies observed in Figure 3

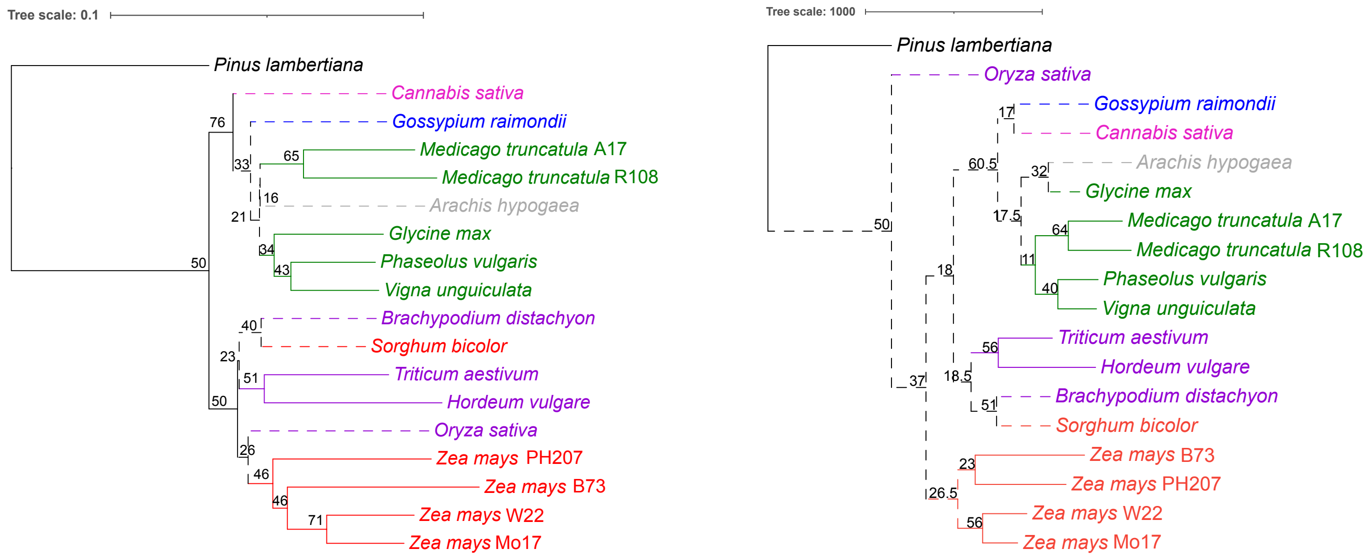

(a) neighbor-joining tree (b) parsimony tree

Figure 3: Neighbor-joining and parsimony trees. Phylograms are colored and rooted as described in Figure 2. For both neighbor-joining (a) and parsimony (b), node values represent the jackknifing support values derived by removing 40% of GO terms in the dataset. Dashed lines mark deviations from known phylogenetic relationships. Tree scales are shown above each, with NJ showing distances and parsimony showing changes in character state.

were *C. sativa*, *A. hypogaea*, *O. sativa* and *B. distachyon*. We hypothesize that the following could account for such errant relationships:

1) Quality of sequencing and coverage assembly: genomes of similarly high sequence coverage that have excellent gene calling would be anticipated to create the best source for functional annotation. Genomes of comparatively lower, or different, character would be anticipated to mislead treebuilding and other comparative genomics approaches.

2) Shared selected or natural traits: species that have been selected for, e.g., oilseeds may share genes involved in synthesis of various oils. Other shared traits would be anticipated to cause similarities for species with those shared traits.

3) Lack of good representation of diverse plant biology aspects in the GO graph: most plant-specific GO terms were derived from functional analysis of one model species, *Arabidopsis thaliana*. This single-source for presence of plant-specific functions limits the graph from containing unique functional aspects of plant biology represented in other species' genomes.

4) Use of a simple method of treebuilding based on the presence or absence of gene function terms: the method we devised and describe here is not sophisticated enough to make full use of information in the GO graphs such that we recover the full detail of the species' evolutionary histories from the simple method.

To consider the first of these, we looked at genome assembly and annotation quality metrics (see Tables IV-V and Figures 6-7). For *B. distachyon* we could find no compelling evidence that assembly structural or functional annotation quality differed significantly from all others, except in the case of *C. sativa*, where we noted that the assembly length exceeded the predicted genome size based on C-values for genome sizes reported previously [72]. In this case, the fact that the *C. sativa* line sequenced is not inbred [73] may be responsible for the inflated assembly size relative to what is expected. This means that in the assembly, there are likely regions where alleles between chromosomes do not align, which would inflate the overall length of the assembly. In addition, the assembly misses a large proportion of BUSCO genes compared to most other genomes included in this analysis. Indeed, the comparatively low-quality assembly for *Cannabis* genome has been noted by others [74], and our preliminary investigations indicate that the assembly length is in fact longer than expected.

In an attempt to better understand conflicting phylogenetic signals that could be caused by the second potential cause, i.e., shared selected or natural traits, we mapped all GO terms that exist in our binary matrix and traced character history (presence/absence) on the nodes and leaves of our expected evolutionary tree using the software Mesquite version 3.61 [75]. These data summarize the gain or loss of each GO term across the species described in this paper and can be found in our Github repository. We carried out a number of simple experiments to reveal which terms could be causal for errant relationships (e.g., dropping all unique terms from the *B. distachyon* dataset, reconstructing the term states at nodes that should be where *B. distachyon* should occur, etc., and could not identify any biologically compelling patterns. (Because these analyses were not fruitful, they

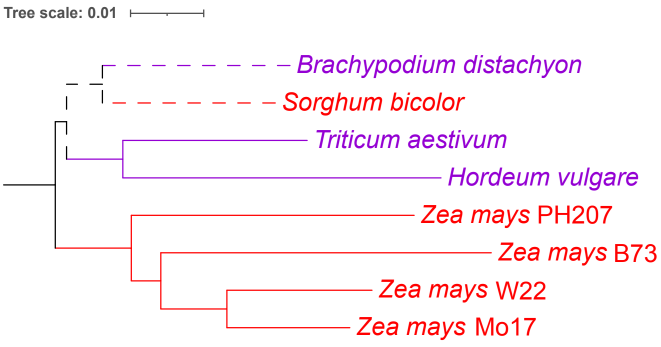

(a) monocots after removing *Oryza sativa*

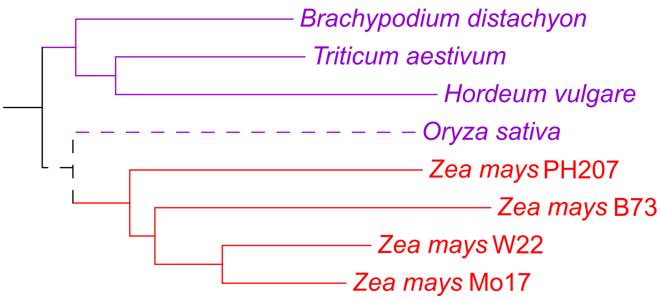

(b) monocots after removing *Sorghum bicolor*

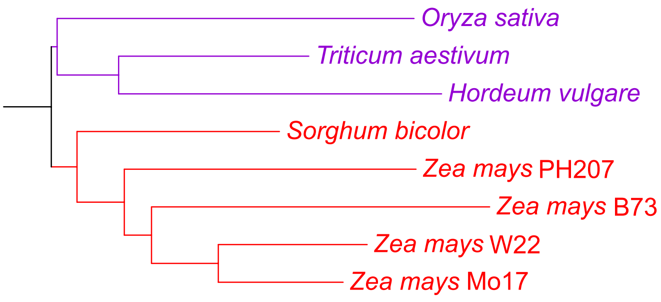

(c) monocots after removing *Brachypodium distachyon*

Figure 4: Restoring monocot relationships. Phylograms are colored and rooted as described in Figure 2. Dashed lines mark deviations from known phylogenetic relationships. Monocot topology changes with removal of a single species: (a) *O. sativa*, (b) *S. bicolor*, and (c) *B. distachyon*. Tree scale is shown above

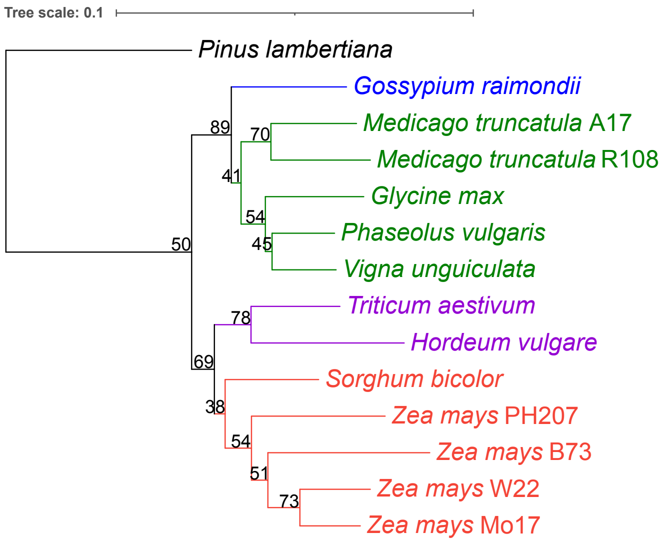

Figure 5: Restoring known phylogentic relationships to the NJ tree via removal of a minimal number of species. Phylograms are colored and rooted as described in Figure 2. Node values represent the jackknifing support values derived by removing 40% of GO terms in the dataset. 4 genomes have been removed: *C. sativa*, *O. sativa*, *B. distachyon*, and *A. hypogaea*. Tree scale is shown above.

were not specifically included in our materials and methods, though we do include the input datasets here, at the link provided in section V "Availability of Source Code and Supporting Data" of the paper, for others to consider and peruse independently.)

An important limitation that must be mentioned is the effect of the third potential cause of errant on our generated phylogenetic trees: a deficiency of terms describing plant biology. Because most GO terms specific to plant biology are likely derived from *Arabidopsis*, a model dicot species, gene functions unique to other species are expected to be missing from the GO graphs [76]. This source of error will only be corrected over time as gene functions unique to diverse plant species are populated into the GO graph.

We consider the most likely explanation for observed discrepancies between the known evolutionary phylogenies and dendrograms created based on GO terms describing gene function to be a result of the fourth explanation: the simplicity of the treebuilding models and methods we used for these analyses. Because the treebuilding and analytics described in this paper were based on the presence/absence of GO terms, novel terms are highly influential to the outcomes of the analysis and the number of times a term is used does not influence the outcome at all. In contrast, plant genomes are notable for having many duplicated genes as a result of whole-genome and segmental duplications over evolutionary history, so these duplications are in fact a feature of and marker for what happened to that genome over time. Therefore, using presence/absence of GO terms where the count of term occurrences are not weighted may be too simple to get at the genuine biological complexities represented in any given plant genome. Our simplistic demonstration of the utility of GO datasets for comparative functional genomics shows that more sophisticated methods are very promising for comparative functional genomics analyses.

It should be noted that comparative analyses using gene functions are not completely absent from the literature - though they are absent for large genome comparisons in plants. An example of such existing comparative use of GO is one where a given tree topology was

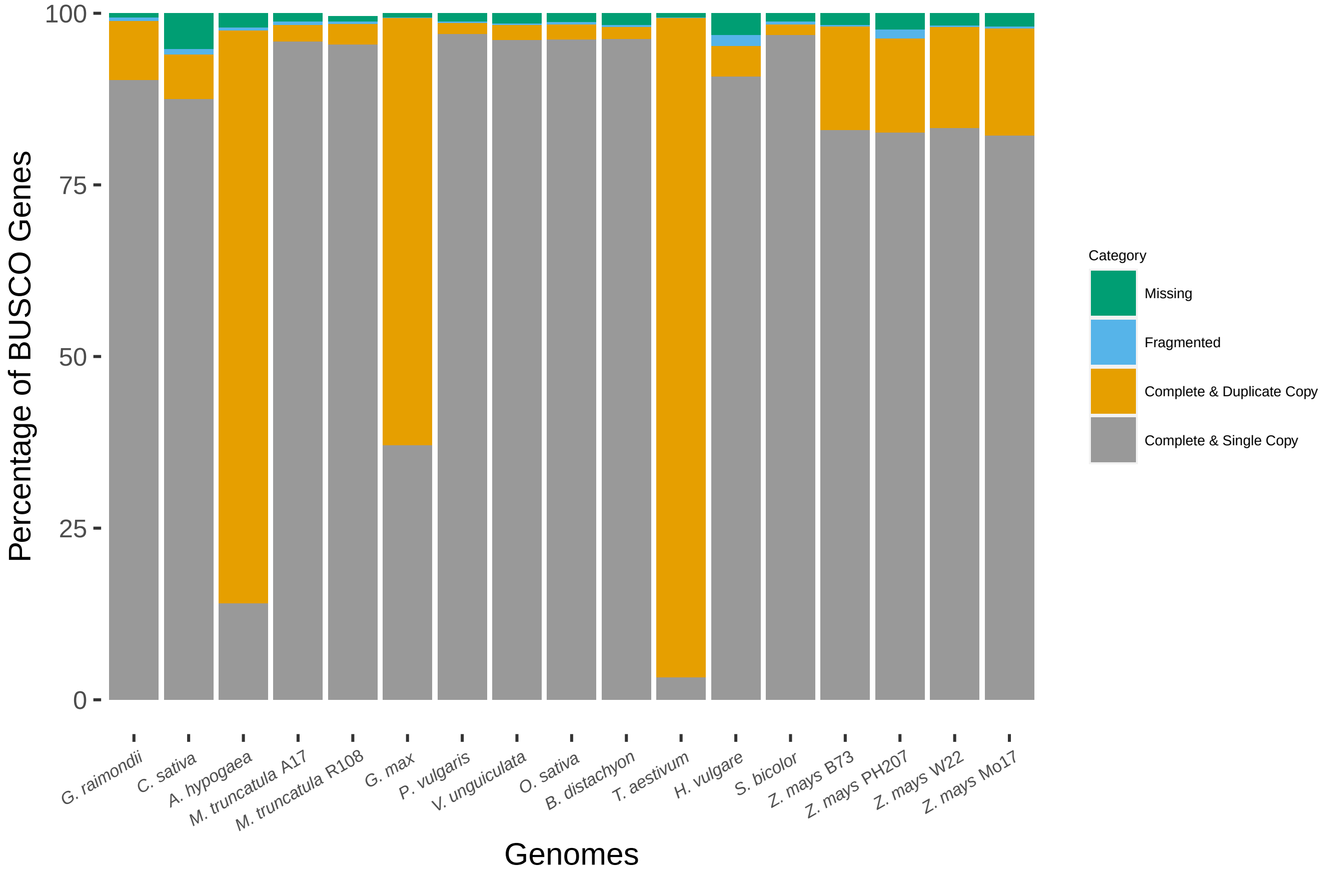

Figure 6: Assembly BUSCO plot generated using GenomeQC. Genomes analyzed are shown across the X-axis, and are ordered to match the occurrence of species shown in Figure 2. Percentage of BUSCO genes across four gene categories are stacked, with each adding up to 100 percent (Y-axis). Complete and single-copy genes are shown in grey, complete and duplicated copies in orange, fragmented copies in blue, and missing are shown copies in green.

used to look for gains and losses of functions mapped to independently derived trees, which was reported by Schwacke et al. [77]. They report, as an example of their method, an analysis of gene loss in *Cuscuta*, a parasitic plant based on analysis using the Mapman ontology. In their work, they showed considerable loss of genes, which is a hallmark of the parasitic lifestyle. Our efforts differ in that we used the functions directly to infer tree structures as a demonstration that sufficient biological signal is present in GO-based datasets of genome-wide function prediction to reproduce known biological relationships. The method we used was quick and dirty, and we anticipate that refinements in approach that consider multiple copies of genes, as well as using different types of graph and network representations beyond tree structures, are logical next steps for refining the use of GO terms for comparative functional genomics analyses in plants. With that in mind, we look forward not only to developing systems to support GO-based comparative functional genomics tools, but also to seeing the tools other research groups will develop to approach the use of these datasets to formulate novel comparative functional genomics hypotheses.

IV. METHODS

A. Acquiring Input Datasets

For each of the 18 genomes listed in Table I, information on how to access input annotation products are listed by DOI. For each, one representative translated peptide sequence per protein coding gene was selected and used as the input for GOMAP, a gene function prediction tool for plants that is actively maintained, updated, and versioned. Details of how GOMAP annotations are derived including the specificity of component datasets and which terms are retained are described elsewhere [12] [9]. In brief, GOMAP annotations are a combination of the annotations from multiple sources. GOMAP combines the annotations from all the sources and removes the less specific annotations that could be

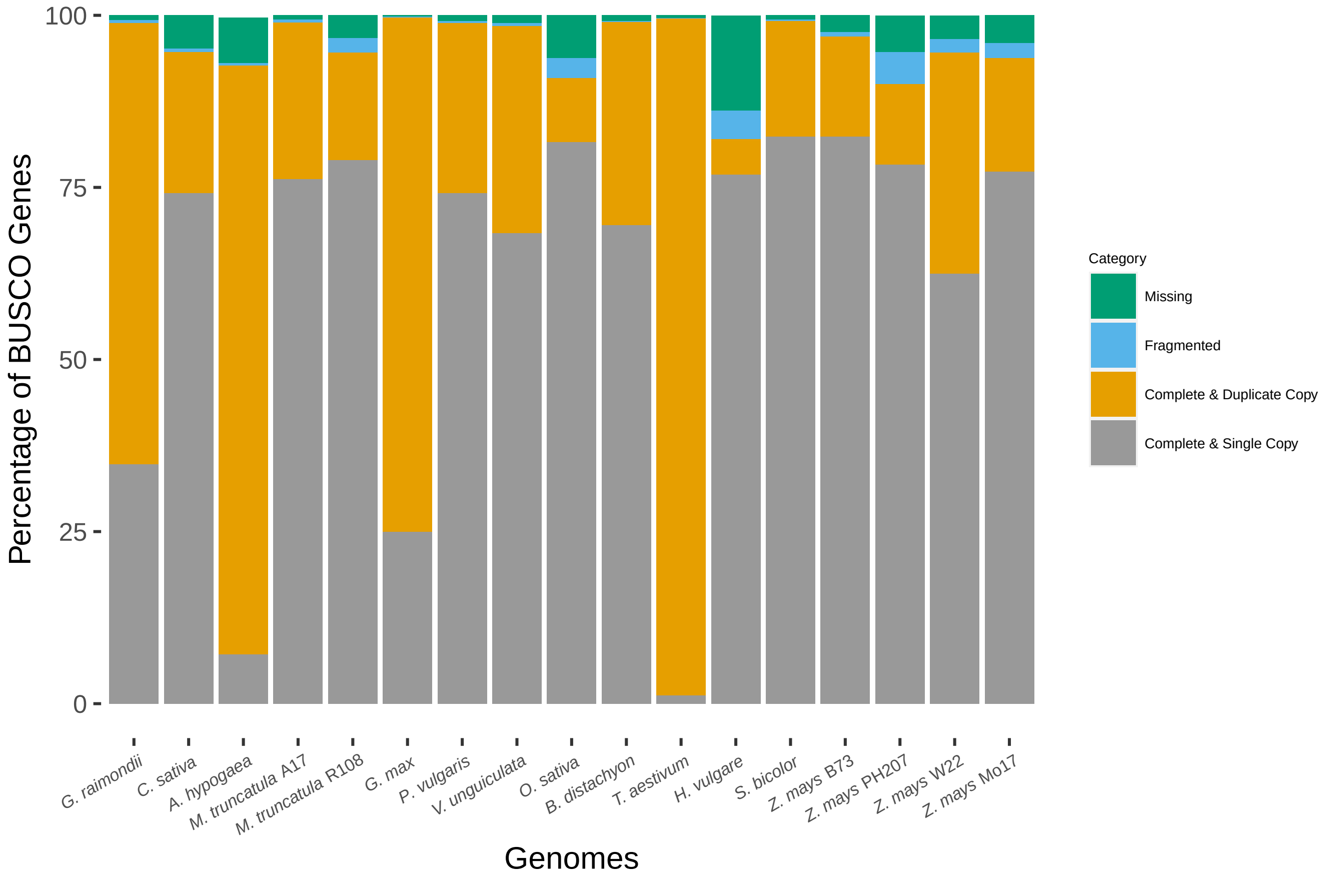

Figure 7: Annotation BUSCO plot generated using GenomeQC. Genomes analyzed are shown across the X-axis, and are ordered to match the occurrence of species shown in Figure 2. Percentage of BUSCO genes across four gene categories are stacked, with each adding up to 100 percent (Y-axis). Complete and single-copy genes are shown in grey, complete and duplicated copies in orange, fragmented copies in blue, and missing are shown copies in green.

inferred from the more specific annotations, keeping only the most specific terms for each gene that cannot be inferred from other terms (i.e., only leaf terms). Unless the authors of the genome provided a set of representative sequences designated as canonical, we chose the longest translated peptide sequence as the representative for each gene model. In general, non-IUPAC characters and trailing asterisks (*) were removed from the sequences, and headers were simplified to contain only non-special characters. The corresponding script for each dataset can be found at the respective DOI. Based on this input, GOMAP yielded a functional annotation set spanning all protein-coding genes in the genome. Using the Gene Ontology version releases/2020-10-09, this functional annotation set was cleaned up by removing duplicates, annotations with qualifiers (NOT, contributes_to, colocalizes_with; column 4 in the GAF 2.1 format), and obsolete GO terms. Any terms containing alternative identifiers were merged to their respective main identifier, uncovering a few additional duplicates, which were also removed. Table SII shows the number of annotations removed from each dataset produced.

To compare the quality of GOMAP predictions to currently available functional predictions from Gramene and Phytozome, we downloaded IEA annotations from Gramene (version 63, [50], https://www.gramene.org/) and Phytozome (version 12, [51], https://phytozome.jgi.doe.gov/) for each species with functional annotations of the same genome version. These datasets were cleaned as above. Similarly cleaned non-IEA annotations from Gramene63 served as the Gold Standard wherever they were available. More detailed information on how these datasets were accessed can be found at https://github.com/Dill-PICL/GOMAP-Paper-2019.1/blob/master/data/go_annotation_sets/README.md.

B. Quantitative and Qualitative Evaluation

The number of annotations in each clean dataset was determined and related to the number of protein coding

genes (based on transcripts in the input FASTA file). This was done for separately for each GO aspect as well as in total.

The ADS software version published in [60] is available from https://bitbucket.org/plyusnin/ads/. We used version b6309cb (also included in our code as a submodule) to calculate SimGIC2, TC-AUCPCR, and F_{max} quality scores. To provide the information content required for the SimGIC2 metric, the Arabidopsis GOA from https://www.ebi.ac.uk/GOA/arabidopsis_release was used in version 2021-02-16.

C. Cladogram Construction

For clustering, we first collected all GO terms annotated to any gene in each genome into a list and removed the duplicates, yielding a one-dimensional set of GO terms for each genome (T). Next, we added all parental terms for each term in this set (connected via *is_a* in the ontology), their respective parental terms and higher, recursively continuing up to the very root of the ontology. Then we once again removed the duplicates, yielding a set S containing the original terms from set T as well as all terms proximal to them in the Gene Ontology directed acyclical graph. These sets with added ancestors served as a starting point of our tree-building analyses: pairwise distances between the genomes were calculated using the Jaccard distance as a metric of the dissimilarity between any two sets a and b.

$$d_{ab} = 1 - \frac{|S_a \cap S_b|}{|S_a \cup S_b|} \quad (1)$$

A neighbor-joining tree was constructed based on the generated pairwise distance matrix using PHYLIP [52]. Additionally, term sets S of all genomes were combined into a binary matrix (with rows corresponding to genomes and columns corresponding to GO terms, values of 0 or 1 indicating whether a term is present or absent in the given set). PHYLIP pars was used to construct a parsimony tree from this binary matrix.

P. lambertiana, a gymnosperm, was included in the dataset as an outgroup to the angiosperms to separate between the monocot and eudicot clades. iTOL [78] was used to visualize the trees using their Newick format, and root them at *P. lambertiana*. Moreover, a cladogram representing the known phylogeny of the included taxa was created by hand based on known evolutionary relationships [79, 80, 81, 82, 83]. This was used to compare the generated phylogenetic relationship based on functional similarity with the evolutionary relationships of the plant genomes.

Jackknifing analysis was carried out for both parsimony and neighbor-joining trees to assess the support for each clade based on the proportion of jackknife trees showing the same clade. To this end, 40% of the terms in T were randomly removed, ancestors of the remaining terms were added and trees constructed as above. The majority rule consensus tree of 100 individual trees was calculated with the jackknife values represented on each branch. The tree was then visualized using iTOL using its Newick format, and rooted again at *P. lambertiana*.

D. Genome Quality Evaluation

Genome size was estimated from the C-values obtained from the Plant DNA C-values data resource from the Kew Database (https://cvalues.science.kew.org). The mean C-value for a given species was used for calculating genome size estimates in base pairs (bp) using the method of [84]. In brief,

$$\text{Genome size (bp)} = \text{C-value (pg)} * 0.978\text{x}10^9 \frac{\text{bp}}{\text{pg}}$$

The estimated genome size (listed in Table IV) was used as an input for GenomeQC (https://genomeqc.maizegdb.org/) [62] to calculate quality metrics. For genomes that were too large to submit through the GenomeQC webtool or had missing exon information, modified scripts of those found in GitHub of GenomeQC (https://github.com/HuffordLab/GenomeQC, commit e6140ee) were applied to calculate the assembly and structural annotation metrics in Table IV and V. BUSCO version 5.0.0 [85] was used to calculate the assembly and annotation BUSCO scores, shown in Figures 6 and 7. The input for assembly BUSCO scores were chromosome sequences, whereas inputs were transcript/mRNA/CDS sequences for the annotation BUSCO scores. For the lineage parameter, the lineage datasets used were as follows: Eudicots for *C. sativa* and *G. raimondii*, Fabales for *A. hypogaea*, *M. truncatula* A17 and R108, *P. vulgaris*, *G. max*, and *V. unguiculata*, and Poales for *B. distachyon*, *O. sativa*, *T. aestivum*, *H. vulgare*, *S. bicolor* and *Z. mays* B73, Mo17, W22 and PH207.

V. AVAILABILITY OF SOURCE CODE AND SUPPORTING DATA

All data and source code generated are freely available at https://github.com/Dill-PICL/GOMAP-Paper-2019.1 under the terms of the MIT license. All software requirements and dependencies are packaged into a Singularity container so no other setup is required to reproduce our results. We will provide a DOI through Zenodo for the final version of the manuscript after reviews and corrections are incorporated.

An up-to-date list of all available annotation sets can be found at https://dill-picl.org/projects/gomap/gomap-datasets/.

VI. DECLARATIONS

A. Competing Interests

The author(s) declare that they have no competing interests.

B. Funding

This work has been supported by the Iowa State University Plant Sciences Institute Faculty Scholars Program to CJLD, the Predictive Plant Phenomics NSF Research Traineeship (#DGE-1545453) to CJLD (CFY and KOC are a trainees), and IOW04714 Hatch funding to Iowa State University.

C. Author's Contributions

LF, DP, CFY, KOC, HAD, PJ, DCS, HV, and KW generated annotations for plants as described in this paper. DP and CJLD co-conceived the idea for phylogenetic analysis. DP wrote the code for the analyses in this paper. LF worked with DP to create dendrograms and compare those to phylogenetic trees. LF carried out assembly and annotation metric comparisons. LF, DP, and CJLD wrote the manuscript. All authors read, offered suggestions to improve, and approved the final copy of the manuscript.

VII. ACKNOWLEDGEMENTS

Thanks to Steven Cannon for help to understand phylogenetic relationships among eudicots and helpful discussions and to Toby Kellogg and Jedrzej Szymanski for discussions and ideas on how to consider the data. Thanks to Nancy Manchanda for reviewing documentation and for checking genome versions used as input for GenomeQC. Thanks to Darwin Campbell who guided the deposition of datasets with CyVerse for release and DOI assignment. Thanks to the reviewers for their suggestions which have improved this manuscript.

VIII. AUTHORS' INFORMATION

KW created the GOMAP system during his time as a graduate student at Iowa State University. LF, DP, CFY, KOC, HV, and PJ are currently graduate students. HAD and DCS are undergraduate students. Each graduate and undergraduate student annotated at least one genome over the course of a research rotation lasting no more than one semester. CJLD coordinated research activities and manuscript preparation.

References

[1] Minoru Kanehisa et al. "KEGG: integrating viruses and cellular organisms". In: *Nucleic Acids Research* 49.D1 (Oct. 2020), pp. D545–D551. DOI: 10.1093/nar/gkaa970. URL: https://doi.org/10.1093/nar/gkaa970.

[2] Michael Ashburner et al. "Gene Ontology: tool for the unification of biology". In: *Nature genetics* 25.1 (May 2000), pp. 25–29. ISSN: 1061-4036. DOI: 10.1038/75556. URL: https://www.ncbi.nlm.nih.gov/pmc/articles/PMC3037419/ (visited on 03/12/2021).

[3] The Gene Ontology Consortium et al. "The Gene Ontology resource: enriching a GOld mine". en. In: *Nucleic Acids Research* 49.D1 (Jan. 2021), pp. D325–D334. ISSN: 0305-1048, 1362-4962. DOI: 10.1093/nar/gkaa1113. URL: https://academic.oup.com/nar/article/49/D1/D325/6027811 (visited on 03/12/2021).

[4] Oliver Thimm et al. "mapman: a user-driven tool to display genomics data sets onto diagrams of metabolic pathways and other biological processes". In: *The Plant Journal* 37.6 (Mar. 2004), pp. 914–939. DOI: 10.1111/j.1365-313x.2004.02016.x. URL: https://doi.org/10.1111/j.1365-313x.2004.02016.x.

[5] D. Binns et al. "QuickGO: a web-based tool for Gene Ontology searching". In: *Bioinformatics* 25.22 (Sept. 2009), pp. 3045–3046. DOI: 10.1093/bioinformatics/btp536. URL: https://doi.org/10.1093/bioinformatics/btp536.

[6] Iris Tzafrir et al. "Identification of Genes Required for Embryo Development in Arabidopsis". en. In: *Plant Physiology* 135.3 (July 2004). Publisher: American Society of Plant Biologists Section: GENOME ANALYSIS, pp. 1206–1220. ISSN: 0032-0889, 1532-2548. DOI: 10.1104/pp.104.045179. URL: http://www.plantphysiol.org/content/135/3/1206 (visited on 03/12/2021).

[7] Ana Conesa and Stefan Götz. "Blast2GO: A Comprehensive Suite for Functional Analysis in Plant Genomics". en. In: *International Journal of Plant Genomics* 2008 (Apr. 2008), pp. 1–12. ISSN: 1687-5370, 1687-5389. DOI: 10.1155/2008/619832. URL: https://www.hindawi.com/journals/ijpg/2008/619832/ (visited on 03/12/2021).

[8] Seung Yon Rhee and Marek Mutwil. "Towards revealing the functions of all genes in plants". In: *Trends in Plant Science* 19.4 (Apr. 2014), pp. 212–221. ISSN: 1360-1385. DOI: 10.1016/j.tplants.2013.10.006. URL: https://www.sciencedirect.com/science/article/pii/S1360138513002343 (visited on 03/12/2021).

[9] Kokulapalan Wimalanathan and Carolyn J. Lawrence-Dill. "Gene Ontology Meta Annotator for Plants (GOMAP)". In: *Plant Methods* 17.1 (May 2021). DOI: 10.1186/s13007-021-00754-1. URL: https://doi.org/10.1186/s13007-021-00754-1.

[10] Naihui Zhou et al. "The CAFA challenge reports improved protein function prediction and new functional annotations for hundreds of genes through experimental screens". In: *Genome Biology* 20.1 (Nov. 2019). DOI: 10.1186/s13059-019-1835-8. URL: https://doi.org/10.1186/s13059-019-1835-8.

[11] URL: https://www.biofunctionprediction.org.

[12] Kokulapalan Wimalanathan et al. "Maize GO Annotation-Methods, Evaluation, and Review (maize-

GAMER)". In: *Plant Direct* 2.4 (Apr. 2018), e00052. ISSN: 24754455. DOI: 10.1002/pld3.52. URL: http://doi.wiley.com/10.1002/pld3.52.

[13] Chengsheng Zhu et al. "Functional Basis of Microorganism Classification". en. In: *PLOS Computational Biology* 11.8 (Aug. 2015). Publisher: Public Library of Science, e1004472. ISSN: 1553-7358. DOI: 10.1371/journal.pcbi.1004472. URL: https://journals.plos.org/ploscompbiol/article?id=10.1371/journal.pcbi.1004472 (visited on 03/12/2021).

[14] Dennis Psaroudakis and Carolyn Lawrence-Dill. *GOMAP Peanut IPGI 1.0*. 2019. DOI: 10.25739/CHAB-0E35. URL: http://datacommons.cyverse.org/browse/iplant/home/shared/commons_repo/curated/Carolyn_Lawrence_Dill_GOMAP_Peanut_Tifrunner.IPGI.1.0_August_2019.r1.

[15] David J. Bertioli et al. "The genome sequence of segmental allotetraploid peanut Arachis hypogaea". In: *Nature Genetics* 51.5 (May 2019), pp. 877–884. DOI: 10.1038/s41588-019-0405-z. URL: https://doi.org/10.1038/s41588-019-0405-z.

[16] Kokulapalan Wimalanathan and Carolyn Lawrence-Dill. *GOMAP Bdistachyon.Bd21.v3.1.r1*. 2019. DOI: 10.25739/DW2T-3G82. URL: http://datacommons.cyverse.org/browse/iplant/home/shared/commons_repo/curated/Carolyn_Lawrence_Dill_GOMAP_Bdistachyon.Bd21.v3.1_November_2019.r1.

[17] "Genome sequencing and analysis of the model grass Brachypodium distachyon". In: *Nature* 463.7282 (Feb. 2010), pp. 763–768. DOI: 10.1038/nature08747. URL: https://doi.org/10.1038/nature08747.

[18] Kevin Chiteri and Carolyn Lawrence-Dill. *GOMAP_Cannabis_sativa_NCBI-cs10_January_2020*. 2020. DOI: 10.25739/AB9Z-2Z86. URL: http://datacommons.cyverse.org/browse/iplant/home/shared/commons_repo/curated/Carolyn_Lawrence_Dill_GOMAP_Cannabis_NCBI-cs10_January_2020.r1.

[19] Kaitlin U. Laverty et al. "A physical and genetic map of Cannabis sativa identifies extensive rearrangements at the THC/CBD acid synthase loci". In: *Genome Research* 29.1 (Nov. 2018), pp. 146–156. DOI: 10.1101/gr.242594.118. URL: https://doi.org/10.1101/gr.242594.118.

[20] Dennis Psaroudakis and Carolyn Lawrence-Dill. *GOMAP Soybean JGI-Wm82.a4.v1*. 2019. DOI: 10.25739/59EC-1719. URL: http://datacommons.cyverse.org/browse/iplant/home/shared/commons_repo/curated/Carolyn_Lawrence_Dill_GOMAP_Soybean_JGI-Wm82.a4.v1_April_2019.r1.

[21] Jeremy Schmutz et al. "Genome sequence of the palaeopolyploid soybean". In: *Nature* 463.7278 (Jan. 2010), pp. 178–183. DOI: 10.1038/nature08670. URL: https://doi.org/10.1038/nature08670.

[22] Parnal Joshi and Carolyn Lawrence-Dill. *GOMAP Gossypium raimondii JGI v2.1*. 2020. DOI: 10.25739/A13T-ZH47. URL: http://datacommons.cyverse.org/browse/iplant/home/shared/commons_repo/curated/Carolyn_Lawrence_Dill_GOMAP_Gossypium_raimondii_JGI_v2.1_January_2020.r1.

[23] Andrew H. Paterson et al. "Repeated polyploidization of Gossypium genomes and the evolution of spinnable cotton fibres". In: *Nature* 492.7429 (Dec. 2012), pp. 423–427. DOI: 10.1038/nature11798. URL: https://doi.org/10.1038/nature11798.

[24] Colleen Yanarella and Carolyn Lawrence-Dill. *GOMAP Barley Reference Sequences IBSC_PGSB_r1*. 2019. DOI: 10.25739/ZVGV-8E37. URL: http://datacommons.cyverse.org/browse/iplant/home/shared/commons_repo/curated/Carolyn_Lawrence_Dill_GOMAP_Barley_IBSC_PGSB-1.0_May_2019.r1.

[25] Martin Mascher et al. "A chromosome conformation capture ordered sequence of the barley genome". In: *Nature* 544.7651 (Apr. 2017), pp. 427–433. DOI: 10.1038/nature22043. URL: https://doi.org/10.1038/nature22043.

[26] Dennis Psaroudakis and Carolyn Lawrence-Dill. *GOMAP Barrel Clover R108_HM340 v1.0*. 2019. DOI: 10.25739/2SQC-J140. URL: http://datacommons.cyverse.org/browse/iplant/home/shared/commons_repo/curated/Carolyn_Lawrence_Dill_GOMAP_BarrelClover_R108_HM340_v1.0_August_2019.r1.

[27] Karen M. Moll et al. "Strategies for optimizing BioNano and Dovetail explored through a second reference quality assembly for the legume model, Medicago truncatula". In: *BMC Genomics* 18.1 (Aug. 2017). DOI: 10.1186/s12864-017-3971-4. URL: https://doi.org/10.1186/s12864-017-3971-4.

[28] Dennis Psaroudakis and Carolyn Lawrence-Dill. *GOMAP Barrel Clover A17_HM341 Mt4.0v2*. 2019. DOI: 10.25739/PY38-YB08. URL: http://datacommons.cyverse.org/browse/iplant/home/shared/commons_repo/curated/Carolyn_Lawrence_Dill_GOMAP_BarrelClover_A17_HM341_Mt4.0v2_August_2019.r1.

[29] Haibao Tang et al. "An improved genome release (version Mt4.0) for the model legume Medicago truncatula". In: *BMC Genomics* 15.1 (2014), p. 312. DOI: 10.1186/1471-2164-15-312. URL: https://doi.org/10.1186/1471-2164-15-312.

[30] Ha Vu and Carolyn Lawrence-Dill. *GOMAP Rice Reference Sequences 2.0*. 2019. DOI: 10.25739/53G0-J859. URL: http://datacommons.cyverse.org/browse/iplant/home/shared/commons_repo/curated/Carolyn_Lawrence_Dill_GOMAP_Rice_IRGSP-1.0_April_2019.r2.

[31] Marcela K Tello-Ruiz et al. "Gramene 2018: unifying comparative genomics and pathway resources for plant research". In: *Nucleic Acids Research* 46.D1 (Nov. 2017), pp. D1181–D1189. DOI: 10.1093/nar/gkx1111. URL: https://doi.org/10.1093/nar/gkx1111.

[32] Dennis Psaroudakis and Carolyn Lawrence-Dill. *GOMAP Common Bean DOE-JGI and USDA-NIFA v2.0*. 2019. DOI: 10.25739/1YWE-EW96. URL: http://datacommons.cyverse.org/browse/iplant/home/shared/commons_repo/curated/Carolyn_Lawrence_Dill_GOMAP_CommonBean_DOE-JGI-USDA-NIFA.2.0_August_2019.r1.

[33] Oscar P Hurtado-Gonzales, Thiago AS Gilio, and MA Pastor-Corrales. "Resistant Reaction of Andean Common Bean Landrace G19833, Reference Genome, to 13 Races of Uromyces appendiculatus Suggests Broad Spectrum Rust Resistance". In: (2017).

[34] Colleen Yanarella and Carolyn Lawrence-Dill. *GOMAP TreeGenesDB sugar pine assembly v1.5*. 2020. DOI: 10.25739/JVS4-XR88. URL: http://datacommons.cyverse.org/browse/iplant/home/shared/commons_repo/curated/Carolyn_Lawrence_Dill_GOMAP_SugarPine_TreeGenesDB-1.5_January_2020.r1.

[35] Marc W Crepeau, Charles H Langley, and Kristian A Stevens. "From Pine Cones to Read Clouds: Rescaffolding the Megagenome of Sugar Pine (Pinus lambertiana)". In: *G3 Genes|Genomes|Genetics* 7.5 (May 2017), pp. 1563–1568. DOI: 10.1534/g3.117.040055. URL: https://doi.org/10.1534/g3.117.040055.

[36] Kokulapalan Wimalanathan and Carolyn Lawrence-Dill. *GOMAP Sbicolor.BTx623.v3.0.1.r1*. 2019. DOI: 10.25739/4TY0-YE98. URL: http://datacommons.cyverse.org/browse/iplant/home/shared/commons_repo/curated/Carolyn_Lawrence_Dill_GOMAP_Sbicolor.BTx623.v3.0.1_November_2019.r1.

[37] Ryan F. McCormick et al. "The Sorghum bicolor reference genome: improved assembly, gene annotations, a transcriptome atlas, and signatures of genome organization". In: *The Plant Journal* 93.2 (Dec. 2017), pp. 338–354. DOI: 10.1111/tpj.13781. URL: https://doi.org/10.1111/tpj.13781.

[38] Dennis Psaroudakis and Carolyn Lawrence-Dill. *GOMAP Wheat Reference Sequences 1.1*. 2019. DOI: 10.25739/65KF-JZ20. URL: http://datacommons.cyverse.org/browse/iplant/home/shared/commons_repo/curated/Carolyn_Lawrence_Dill_GOMAP_Wheat_RefSeq1.1_HC_December_2018.r1.

[39] Michael Alaux et al. "Linking the International Wheat Genome Sequencing Consortium bread wheat reference genome sequence to wheat genetic and phenomic data". In: *Genome Biology* 19.1 (Aug. 2018). DOI: 10.1186/s13059-018-1491-4. URL: https://doi.org/10.1186/s13059-018-1491-4.

[40] Dennis Psaroudakis and Carolyn Lawrence-Dill. *GOMAP Cowpea IT97K-499-35 JGI annotation v1.1*. 2019. DOI: 10.25739/CDX9-WR97. URL: http://datacommons.cyverse.org/browse/iplant/home/shared/commons_repo/curated/Carolyn_Lawrence_Dill_GOMAP_Cowpea_JGI.v1.1_August_2019.r1.

[41] Stefano Lonardi et al. "The genome of cowpea (Vigna unguiculata [L.] Walp.)" In: (Jan. 2019). DOI: 10.1101/518969. URL: https://doi.org/10.1101/518969.

[42] Kokulapalan Wimalanathan and Carolyn Lawrence-Dill. *GOMAP Maize Zm-Mo17-REFERENCE-CAU-1.0 Zm00014a.1*. 2019. DOI: 10.25739/M634-CN58. URL: http://datacommons.cyverse.org/browse/iplant/home/shared/commons_repo/curated/Carolyn_Lawrence_Dill_GOMAP_maize.Mo17.AGPv1_April_2019.r1.

[43] Silong Sun et al. "Extensive intraspecific gene order and gene structural variations between Mo17 and other maize genomes". In: *Nature Genetics* 50.9 (July 2018), pp. 1289–1295. DOI: 10.1038/s41588-018-0182-0. URL: https://doi.org/10.1038/s41588-018-0182-0.

[44] Kokulapalan Wimalanathan and Carolyn Lawrence-Dill. *GOMAP Maize Zm-PH207-REFERENCE_NS-UIUC_UMN-1.0 Zm00008a.1*. 2019. DOI: 10.25739/DM9S-AA15. URL: http://datacommons.cyverse.org/browse/iplant/home/shared/commons_repo/curated/Carolyn_Lawrence_Dill_GOMAP_maize.PH207.UIUC_UMN-1.0_April_2019.r1.

[45] Candice N. Hirsch et al. "Draft Assembly of Elite Inbred Line PH207 Provides Insights into Genomic and Transcriptome Diversity in Maize". In: *The Plant Cell* 28.11 (Nov. 2016), pp. 2700–2714. DOI: 10.1105/tpc.16.00353. URL: https://doi.org/10.1105/tpc.16.00353.

[46] Kokulapalan Wimalanathan and Carolyn Lawrence-Dill. *GOMAP Maize Zm-W22-REFERENCE-NRGENE-2.0 Zm00004b.1*. 2019. DOI: 10.25739/E4VA-9F09. URL: http://datacommons.cyverse.org/browse/iplant/home/shared/commons_repo/curated/Carolyn_Lawrence_Dill_GOMAP_maize.W22.AGPv2_April_2019.r1.

[47] Nathan M. Springer et al. "The maize W22 genome provides a foundation for functional genomics and transposon biology". In: *Nature Genetics* 50.9 (July 2018), pp. 1282–1288. DOI: 10.1038/s41588-018-0158-0. URL: https://doi.org/10.1038/s41588-018-0158-0.

[48] Kokulapalan Wimalanathan and Carolyn Lawrence-Dill. *maize-GAMER Annotaions for maize.B73.AGPv4.r1*. 2017. DOI: 10.7946/P2M925. URL: http://datacommons.cyverse.org/browse/iplant/home/shared/commons_repo/curated/Carolyn_Lawrence-Dill_maize-GAMER_maize.B73_RefGen_v4_Zm00001d.2_Oct_2017.r1.

[49] Yinping Jiao et al. "Improved maize reference genome with single-molecule technologies". In: *Nature* 546.7659 (June 2017), pp. 524–527. DOI: 10.1038/nature22971. URL: https://doi.org/10.1038/nature22971.

[50] Marcela K Tello-Ruiz et al. "Gramene 2021: harnessing the power of comparative genomics and pathways for plant research". In: *Nucleic Acids Research* 49.D1 (Nov. 2020), pp. D1452–D1463. DOI: 10.1093/nar/gkaa979. URL: https://doi.org/10.1093/nar/gkaa979.

[51] David M. Goodstein et al. "Phytozome: a comparative platform for green plant genomics". In: *Nucleic Acids Research* 40.D1 (Nov. 2011), pp. D1178–D1186. DOI: 10.1093/nar/gkr944. URL: https://doi.org/10.1093/nar/gkr944.

[52] Joseph Felsenstein. *PHYLIP (phylogeny inference package), version 3.5 c.* Joseph Felsenstein., 1993.

[53] Walter M. Fitch. "Toward Defining the Course of Evolution: Minimum Change for a Specific Tree Topology". In: *Systematic Zoology* 20.4 (Dec. 1971), p. 406. DOI: 10.2307/2412116. URL: https://doi.org/10.2307/2412116.

[54] N Saitou and M Nei. "The neighbor-joining method: a new method for reconstructing phylogenetic trees." In: *Molecular Biology and Evolution* (July 1987). DOI: 10.1093/oxfordjournals.molbev.a040454. URL: https://doi.org/10.1093/oxfordjournals.molbev.a040454.

[55] C. F. J. Wu. "Jackknife, Bootstrap and Other Resampling Methods in Regression Analysis". In: *The Annals of Statistics* 14.4 (Dec. 1986). DOI: 10.1214/aos/1176350142. URL: https://doi.org/10.1214/aos/1176350142.

[56] S. C. Potter. "The Ensembl Analysis Pipeline". In: *Genome Research* 14.5 (May 2004), pp. 934–941. DOI: 10.1101/gr.1859804. URL: https://doi.org/10.1101/gr.1859804.

[57] Christophe Dessimoz, Nives Škunca, and Paul D. Thomas. "CAFA and the Open World of protein function predictions". In: *Trends in Genetics* 29.11 (Nov. 2013), pp. 609–610. DOI: 10.1016/j.tig.2013.09.005. URL: https://doi.org/10.1016/j.tig.2013.09.005.

[58] Karin Verspoor et al. "A categorization approach to automated ontological function annotation". In: *Protein Science* (2006). ISSN: 09618368. DOI: 10.1110/ps.062184006. URL: https://dx.doi.org/10.1110%7B%5C%%7D2Fps.062184006.

[59] Michael Defoin-Platel et al. "AIGO: Towards a unified framework for the Analysis and the Inter-comparison of GO functional annotations". In: *BMC Bioinformatics* (2011). ISSN: 14712105. DOI: 10.1186/1471-2105-12-431. URL: https://doi.org/10.1186/1471-2105-12-431.

[60] Ilya Plyusnin, Liisa Holm, and Petri Törönen. "Novel comparison of evaluation metrics for gene ontology classifiers reveals drastic performance differences". In: *PLOS Computational Biology* 15.11 (Nov. 2019). Ed. by Predrag Radivojac, e1007419. ISSN: 1553-7358. DOI: 10.1371/journal.pcbi.1007419. URL: http://dx.plos.org/10.1371/journal.pcbi.1007419.

[61] Ivica Letunic and Peer Bork. "Interactive Tree Of Life (iTOL) v5: an online tool for phylogenetic tree display and annotation". In: *Nucleic Acids Research* (Apr. 2021). DOI: 10.1093/nar/gkab301. URL: https://doi.org/10.1093/nar/gkab301.

[62] Nancy Manchanda et al. "GenomeQC: a quality assessment tool for genome assemblies and gene structure annotations". In: *BMC Genomics* 21.1 (Mar. 2020), p. 193. ISSN: 1471-2164. DOI: 10.1186/s12864-020-6568-2. URL: https://doi.org/10.1186/s12864-020-6568-2 (visited on 03/12/2021).

[63] Felipe A. Simão et al. "BUSCO: assessing genome assembly and annotation completeness with single-copy orthologs". en. In: *Bioinformatics* 31.19 (Oct. 2015), pp. 3210–3212. ISSN: 1367-4803, 1460-2059. DOI: 10.1093/bioinformatics/btv351. URL: https://academic.oup.com/bioinformatics/article-lookup/doi/10.1093/bioinformatics/btv351 (visited on 03/12/2021).

[64] Haley Dostalik and Carolyn Lawrence-Dill. *Carolyn_Lawrence_Dill_GOMAP_Grape_Genoscope_12x_January_2021.r1*. 2021. DOI: 10.25739/JTFK-Q888. URL: http://datacommons.cyverse.org/browse/iplant/home/shared/commons_repo/curated/Carolyn_Lawrence_Dill_GOMAP_Grape_Genoscope_12x_January_2021.r1.

[65] Carolyn Lawrence-Dill. *Carolyn_Lawrence_Dill_GOMAP_Canola_BnPIR_ZS11_March_2021.r1*. 2021. DOI: 10.25739/XGMR-HR31. URL: http://datacommons.cyverse.org/browse/iplant/home/shared/commons_repo/curated/Carolyn_Lawrence_Dill_GOMAP_Canola_BnPIR_ZS11_March_2021.r1.

[66] Leila Fattel and Carolyn Lawrence-Dill. *Carolyn_Lawrence_Dill_GOMAP_Banana_NCBI_ASM31385v2_February_2021.r1*. 2021. DOI: 10.25739/YT7W-GS55. URL: http://datacommons.cyverse.org/browse/iplant/home/shared/commons_repo/curated/Carolyn_Lawrence_Dill_GOMAP_Banana_NCBI_ASM31385v2_February_2021.r1.

[67] Leila Fattel and Carolyn Lawrence-Dill. *Carolyn_Lawrence_Dill_GOMAP_Cacao_NCBI_CrilloV2_March_2021.r1*. 2021. DOI: 10.25739/9QC0-N310. URL: http://datacommons.cyverse.org/browse/iplant/home/shared/commons_repo/curated/Carolyn_Lawrence_Dill_GOMAP_Cacao_NCBI_CriolloV2_March_2021.r1.

[68] Carolyn Lawrence-Dill. *Carolyn_Lawrence_Dill_GOMAP_Coffee_CGH_v1.0_June_2021.r1*. 2021. DOI: 10.25739/RM4J-3580. URL: http://datacommons.cyverse.org/browse/iplant/home/shared/commons_repo/curated/Carolyn_Lawrence_Dill_GOMAP_Coffee_CGH_v1.0_June_2021.r1.

[69] Carolyn Lawrence-Dill. *Carolyn_Lawrence_Dill_GOMAP_Blueberry_GigaDB_v1.0_June* 2021. DOI: 10.25739/Q7RQ-E992. URL: http://datacommons.cyverse.org/browse/iplant/home/shared/commons_repo/curated/Carolyn_Lawrence_Dill_GOMAP_Blueberry_GigaDB_v1.0_June_2021.r1.

[70] Dennis Psaroudakis and Carolyn Lawrence-Dill. *Carolyn_Lawrence_Dill_GOMAP_Solanum_lycopersicum_ITAG4.1.v1* 2021. DOI: 10.25739/ZH2V-4P15. URL: http://datacommons.cyverse.org/browse/iplant/home/shared/commons_repo/curated/Carolyn_Lawrence_Dill_GOMAP_Solanum_lycopersicum_ITAG4.1.v1_April_2021.r1.

[71] Dennis Psaroudakis and Carolyn Lawrence-Dill. *Carolyn_Lawrence_Dill_GOMAP_Solanum_pennellii_Bolger2014.v1_A* 2021. DOI: 10.25739/FHR4-CX67. URL: http://datacommons.cyverse.org/browse/iplant/home/shared/commons_repo/curated/Carolyn_Lawrence_Dill_GOMAP_Solanum_pennellii_Bolger2014.v1_April_2021.r1.

[72] Koichi Sakamoto et al. "Characterization Genome Sizes and Morphology of Sex Chromosomes in Hemp (Cannabis sativa L.)." In: *CYTOLOGIA* 63.4 (1998), pp. 459–464. DOI: 10.1508/cytologia.63.459. URL: https://doi.org/10.1508/cytologia.63.459.

[73] Harm van Bakel et al. "The draft genome and transcriptome of Cannabis sativa". In: *Genome Biology* 12.10 (2011), R102. DOI: 10.1186/gb-2011-12-10-r102. URL: https://doi.org/10.1186/gb-2011-12-10-r102.

[74] Shan Gao et al. "A high-quality reference genome of wild Cannabis sativa". en. In: *Horticulture Research* 7.1 (May 2020). Number: 1 Publisher: Nature Publishing Group, pp. 1–11. ISSN: 2052-7276. DOI: 10.1038/s41438-020-0295-3. URL: https://www.nature.com/articles/s41438-020-0295-3 (visited on 03/12/2021).

[75] W.P. Maddison and D.R. Maddison. *Mesquite: a modular system for evolutionary analysis. Version 3.61*. 2021. URL: http://www.mesquiteproject.org/ (visited on 07/14/2021).

[76] Peifen Zhang et al. "PhyloGenes: An online phylogenetics and functional genomics resource for plant gene function inference". In: *Plant Direct* 4.12 (Dec. 2020). DOI: 10.1002/pld3.293. URL: https://doi.org/10.1002/pld3.293.

[77] Rainer Schwacke et al. "MapMan4: A Refined Protein Classification and Annotation Framework Applicable to Multi-Omics Data Analysis". In: *Molecular Plant* 12.6 (June 2019), pp. 879–892. DOI: 10.1016/j.molp.2019.01.003. URL: https://doi.org/10.1016/j.molp.2019.01.003.

[78] Ivica Letunic and Peer Bork. "Interactive Tree Of Life (iTOL) v4: recent updates and new developments". en. In: *Nucleic Acids Research* 47.W1 (July 2019), W256–W259. ISSN: 0305-1048, 1362-4962. DOI: 10.1093/nar/gkz239. URL: https://academic.oup.com/nar/article/47/W1/W256/5424068 (visited on 03/12/2021).

[79] Elizabeth A. Kellogg. "Evolutionary History of the Grasses". en. In: *Plant Physiology* 125.3 (Mar. 2001), pp. 1198–1205. ISSN: 0032-0889, 1532-2548. DOI: 10.1104/pp.125.3.1198. URL: https://academic.oup.com/plphys/article/125/3/1198-1205/6109905 (visited on 03/12/2021).

[80] Steven B. Cannon and Randy C. Shoemaker. "Evolutionary and comparative analyses of the soybean

genome". eng. In: *Breeding Science* 61.5 (Jan. 2012), pp. 437–444. ISSN: 1344-7610. DOI: 10.1270/jsbbs.61.437.

[81] Candice N. Hansey et al. "Maize (Zea mays L.) genome diversity as revealed by RNA-sequencing". eng. In: *PloS One* 7.3 (2012), e33071. ISSN: 1932-6203. DOI: 10.1371/journal.pone.0033071.

[82] Nasim Azani et al. "A new subfamily classification of the Leguminosae based on a taxonomically comprehensive phylogeny: The Legume Phylogeny Working Group (LPWG)". en. In: *TAXON* 66.1 (2017). _eprint: https://onlinelibrary.wiley.com/doi/pdf/10.12705/661.3, pp. 44–77. ISSN: 1996-8175. DOI: https://doi.org/10.12705/661.3. URL: https://onlinelibrary.wiley.com/doi/abs/10.12705/661.3 (visited on 03/12/2021).

[83] Mark N. Puttick et al. "The Interrelationships of Land Plants and the Nature of the Ancestral Embryophyte". English. In: *Current Biology* 28.5 (Mar. 2018). Publisher: Elsevier, 733–745.e2. ISSN: 0960-9822. DOI: 10.1016/j.cub.2018.01.063. URL: https://www.cell.com/current-biology/abstract/S0960-9822(18)30096-4 (visited on 03/12/2021).

[84] J. Doležel et al. "Letter to the editor". In: *Cytometry* 51A.2 (Jan. 2003), pp. 127–128. DOI: 10.1002/cyto.a.10013. URL: https://doi.org/10.1002/cyto.a.10013.

[85] Mathieu Seppey, Mosè Manni, and Evgeny M. Zdobnov. "BUSCO: Assessing Genome Assembly and Annotation Completeness". In: *Methods in Molecular Biology*. Springer New York, 2019, pp. 227–245. DOI: 10.1007/978-1-4939-9173-0_14. URL: https://doi.org/10.1007/978-1-4939-9173-0_14.

[86] Eva M Temsch and Johann Greilhuber. "Genome size variation in Arachis hypogaea and A. monticola re-evaluated". In: *Genome* 43.3 (June 2000), pp. 449–451. DOI: 10.1139/g99-130. URL: https://doi.org/10.1139/g99-130.

[87] Pilar Catalán et al. "Evolution and taxonomic split of the model grass Brachypodium distachyon". In: *Annals of Botany* 109.2 (Jan. 2012), pp. 385–405. DOI: 10.1093/aob/mcr294. URL: https://doi.org/10.1093/aob/mcr294.

[88] Johann Greilhuber and Renate Obermayer. "Genome size and maturity group in Glycine max (soybean)". In: *Heredity* 78.5 (1997), pp. 547–551. DOI: 10.1038/hdy.1997.85. URL: https://doi.org/10.1038/hdy.1997.85.

[89] B. HENDRIX. "Estimation of the Nuclear DNA Content of Gossypium Species". In: *Annals of Botany* 95.5 (Feb. 2005), pp. 789–797. DOI: 10.1093/aob/mci078. URL: https://doi.org/10.1093/aob/mci078.

[90] Michael David Bennett and JB Smith. "Nuclear DNA amounts in angiosperms". In: *Philosophical Transactions: Biological Sciences* (1991), pp. 309–345. DOI: 10.1098/rstb.1991.0120. URL: https://doi.org/10.1098/rstb.1991.0120.

[91] Fatima Pustahija et al. "Small genomes dominate in plants growing on serpentine soils in West Balkans, an exhaustive study of 8 habitats covering 308 taxa". In: *Plant and soil* 373.1 (2013), pp. 427–453. DOI: 10.1007/s11104-013-1794-x. URL: https://doi.org/10.1007/s11104-013-1794-x.

[92] Ka Arumuganathan and ED Earle. "Nuclear DNA content of some important plant species". In: *Plant molecular biology reporter* 9.3 (1991), pp. 208–218. DOI: 10.1007/BF02672069. URL: https://doi.org/10.1007/BF02672069.

[93] AY Kenton, SJ Owens, and D Langton. "The origin of ringformation and self-compatibility in Gibasis pulchella (Commelinaceae)". In: *Kew Chromosome Conference*. Vol. 3. 1988, pp. 75–84.

[94] DA Laurie and MD Bennett. "Nuclear DNA content in the genera Zea and Sorghum. Intergeneric, interspecific and intraspecific variation". In: *Heredity* 55.3 (1985), pp. 307–313. DOI: 10.1038/hdy.1985.112. URL: https://doi.org/10.1038/hdy.1985.112.

[95] A Parida, SN Raina, and RKJ Narayan. "Quantitative DNA variation between and within chromosome complements of Vigna species (Fabaceae)". In: *Genetica* 82.2 (1990), pp. 125–133. DOI: 10.1007/BF00124642. URL: https://doi.org/10.1007/BF00124642.

Table II: Quantitative metrics of the cleaned functional annotation sets. CC, MF, BP, and A refer to the aspects of the Gene Ontology: Cellular Component, Molecular Function, Biological Process, and Any/All. GOMAP covers all genomes with at least one annotation per gene and provides substantially more annotations than Gramene63 or Phytozome, especially in the BP aspect. The total number of annotations per dataset is visualized in Figure S1.

			Genes Annotated[%][a]				Annotations[b]				Median Ann. per G.[c]			
Genome	Genes	Dataset	CC	MF	BP	**A**	CC	MF	BP	**A**	CC	MF	BP	**A**
Arachis hypogaea	67,124	GOMAP	85.85	84.68	100.00	**100.00**	150,525	132,144	493,145	**775,814**	2	2	6	**10**
Brachypodium distachyon	34,310	GOMAP	81.33	85.35	100.00	**100.00**	74,172	69,213	255,397	**398,782**	2	2	6	**10**
		Gold Standard Gramene 63 (no IEA)	21.54	19.53	18.20	**26.66**	10,985	10,436	11,120	**32,673**	1	1	1	**3**
		Gramene63 (IEA only)	33.12	49.29	38.29	**63.60**	21,658	36,372	23,899	**82,026**	1	1	1	**3**
		Phytozome12	10.25	37.21	26.86	**43.11**	4,186	18,597	11,070	**34,060**	0	1	1	**2**
Cannabis sativa	33,677	GOMAP	94.22	95.48	100.00	**100.00**	85,755	73,614	262,741	**422,110**	2	2	6	**11**
Glycine max	52,872	GOMAP	86.95	88.92	100.00	**100.00**	126,470	113,068	416,989	**656,527**	2	2	6	**11**
Gossypium raimondii	37,505	GOMAP	93.00	92.37	100.00	**100.00**	95,419	84,910	307,470	**487,799**	2	2	6	**11**
Hordeum vulgare	39,734	GOMAP	88.57	91.76	100.00	**100.00**	86,489	79,727	272,420	**438,636**	2	2	5	**10**
		Gold Standard Gramene 63 (no IEA)	28.23	26.30	23.43	**35.64**	15,734	15,391	15,267	**46,414**	1	1	1	**3**
		Gramene63 (IEA only)	36.19	50.90	41.71	**65.03**	29,826	44,789	29,425	**104,178**	1	1	1	**3**
Medicago truncatula A17	50,444	GOMAP	83.79	86.69	100.00	**100.00**	104,902	99,155	363,608	**567,665**	2	2	6	**10**
		Gold Standard Gramene 63 (no IEA)	25.45	23.26	21.51	**32.12**	17,938	18,416	18,461	**54,827**	1	1	1	**3**
		Gramene63 (IEA only)	34.25	50.84	40.26	**66.14**	32,753	63,470	40,441	**137,001**	1	1	1	**3**
		Phytozome12	8.87	36.05	25.83	**41.07**	5,315	25,950	15,576	**47,098**	0	1	1	**2**
Medicago truncatula R108	55,706	GOMAP	72.10	90.14	100.00	**100.00**	108,388	107,499	381,831	**597,718**	1	2	5	**9**
Oryza sativa	35,825	GOMAP	79.78	83.31	100.00	**100.00**	71,306	64,150	248,304	**383,760**	2	2	6	**9**
		Gold Standard Gramene 63 (no IEA)	29.95	27.29	25.33	**37.57**	15,492	15,176	16,536	**47,339**	1	1	1	**3**
		Gramene63 (IEA only)	32.21	45.83	36.75	**60.13**	21,935	37,425	24,255	**83,645**	1	1	1	**3**
		Phytozome12	10.31	40.10	29.18	**46.09**	4,361	20,842	12,451	**37,884**	0	1	1	**2**
Phaseolus vulgaris	27,433	GOMAP	94.48	93.06	100.00	**100.00**	70,987	64,022	229,230	**364,239**	2	2	6	**11**
Pinus lambertiana	31,007	GOMAP	92.67	95.91	100.00	**100.00**	71,247	68,315	212,248	**351,810**	2	2	5	**10**
Sorghum bicolor	34,129	GOMAP	82.44	85.98	100.00	**100.00**	75,145	69,659	259,004	**403,808**	2	2	6	**10**
		Gold Standard Gramene 63 (no IEA)	34.48	32.91	30.90	**42.84**	16,837	17,614	17,850	**52,593**	1	1	1	**3**
		Gramene63 (IEA only)	35.91	52.11	42.36	**67.41**	23,608	39,418	27,074	**90,313**	1	1	1	**3**
		Phytozome12	10.54	39.19	27.90	**45.10**	4,246	19,724	11,432	**35,599**	0	1	1	**2**
Triticum aestivum	107,891	GOMAP	88.53	90.98	100.00	**100.00**	259,318	217,467	785,051	**1,261,836**	2	2	6	**10**
		Gold Standard Gramene 63 (no IEA)	2.98	2.78	2.56	**3.82**	4,727	4,512	4,793	**14,035**	1	1	1	**3**
		Gramene63 (IEA only)	29.12	58.62	38.72	**70.41**	47,595	111,889	62,977	**222,721**	0	1	1	**2**
Vigna unguiculata	29,773	GOMAP	91.21	91.08	100.00	**100.00**	74,791	67,734	242,847	**385,372**	2	2	6	**11**
		Phytozome12	13.91	45.68	34.14	**53.06**	5,107	19,962	12,209	**37,534**	0	1	1	**2**
Zea mays B73.v4	39,324	GOMAP	93.16	94.92	100.00	**100.00**	87,648	81,665	278,305	**447,618**	2	2	6	**10**
		Gold Standard Gramene 63 (no IEA)	37.92	34.78	32.67	**46.85**	22,531	21,292	23,153	**67,285**	1	1	1	**3**
		Gramene63 (IEA only)	39.16	58.16	48.21	**73.87**	30,189	53,748	35,276	**119,273**	1	1	1	**3**
Zea mays Mo17	38,620	GOMAP	86.98	90.87	100.00	**100.00**	86,074	78,650	277,395	**442,119**	2	2	6	**10**
		Gold Standard Gramene 63 (no IEA)	27.56	25.20	23.73	**33.98**	16,128	15,384	16,489	**48,220**	1	1	1	**3**
Zea mays PH207	40,557	GOMAP	86.55	90.61	100.00	**100.00**	88,962	84,910	288,208	**462,080**	2	2	6	**10**
		Gold Standard Gramene 63 (no IEA)	28.18	25.82	24.26	**34.66**	17,370	16,580	17,791	**51,984**	1	1	1	**3**
Zea mays W22	40,690	GOMAP	90.77	92.58	100.00	**100.00**	93,622	84,450	289,364	**467,436**	2	2	6	**10**
		Gold Standard Gramene 63 (no IEA)	25.40	23.15	21.80	**31.29**	15,518	14,818	15,850	**46,402**	1	1	1	**3**

Download this table (CSV)

[a] How many genes in the genome have at least one GO term from the CC, MF, BP aspect annotated to them? A = How many at least one from any aspect? (A = CC ∪ MF ∪ BP)

[b] How many annotations in the CC, MF, and BP aspect does this dataset contain? A = How many in total? A = CC + MF + BP

[c] Take a typical gene that is present in the annotation set. How many annotations does it have in each aspect? A = How many in total? Please note that A ≠ CC + MF + BP

Table III: Qualitative metrics of functional annotation sets predicted by GOMAP, Gramene, and Phytozome. This table is visualized in Figure S2.

Genome	Dataset	SimGIC2 CC	SimGIC2 MF	SimGIC2 BP	TC-AUCPCR CC	TC-AUCPCR MF	TC-AUCPCR BP	Fmax CC	Fmax MF	Fmax BP
Brachypodium distachyon	GOMAP	0.404149	0.464127	0.223830	0.233442	0.230701	0.118526	0.741361	0.740897	0.526881
	Gramene63 (IEA only)	0.317801	0.420859	0.349406	0.129163	0.192507	0.111361	0.691016	0.738542	0.650325
	Phytozome12	0.370264	0.370521	0.352206	0.112582	0.136832	0.085628	0.717759	0.697076	0.660603
Hordeum vulgare	GOMAP	0.400087	0.470012	0.238177	0.237231	0.261399	0.130784	0.745272	0.750213	0.560096
	Gramene63 (IEA only)	0.306119	0.426601	0.381010	0.157352	0.228797	0.136002	0.680996	0.742638	0.665696
Medicago truncatula A17	GOMAP	0.371795	0.451258	0.213407	0.272809	0.282650	0.139032	0.730838	0.726991	0.531406
	Gramene63 (IEA only)	0.329600	0.437274	0.343561	0.176497	0.265887	0.133503	0.701093	0.749900	0.654297
	Phytozome12	0.358311	0.367257	0.363013	0.144247	0.170863	0.110386	0.717307	0.698429	0.661233
Oryza sativa	GOMAP	0.408945	0.482650	0.248207	0.298502	0.303384	0.159724	0.751121	0.757181	0.559221
	Gramene63 (IEA only)	0.328761	0.423191	0.341193	0.167619	0.265410	0.135451	0.711309	0.738732	0.643827
	Phytozome12	0.049975	0.041007	0.044279	0.000003	0.000003	0.000002	0.470134	0.266628	0.239256
Sorghum bicolor	GOMAP	0.404852	0.466708	0.224011	0.316873	0.337380	0.169883	0.746540	0.742001	0.534258
	Gramene63 (IEA only)	0.323037	0.400241	0.353135	0.177038	0.260198	0.154157	0.711107	0.712170	0.653591
	Phytozome12	0.356091	0.348264	0.340124	0.151947	0.177579	0.110483	0.715714	0.675147	0.641535
Triticum aestivum	GOMAP	0.410582	0.489881	0.229271	0.050762	0.030610	0.019360	0.736476	0.762420	0.533897
	Gramene63 (IEA only)	0.362452	0.476685	0.395112	0.040992	0.043701	0.027872	0.737769	0.762059	0.670953
Zea mays B73.v4	GOMAP	0.417455	0.467339	0.245373	0.302761	0.290371	0.153011	0.759504	0.746870	0.564707
	Gramene63 (IEA only)	0.303231	0.416301	0.346308	0.175735	0.250075	0.138275	0.662987	0.732860	0.647725
Zea mays Mo17	GOMAP	0.399521	0.464265	0.225632	0.236209	0.239598	0.125599	0.744360	0.743026	0.537489
Zea mays PH207	GOMAP	0.394481	0.436266	0.224226	0.221709	0.221266	0.117086	0.743111	0.718933	0.533092
Zea mays W22	GOMAP	0.397602	0.463499	0.223511	0.210198	0.217609	0.113262	0.743783	0.742341	0.535572

Download this table (CSV)

Table IV: Assembly statistics*

	C-value (pg)	C-value Ref.	Estimated Genome Size (Mb)	Scaffolds	Total Scaffold Length (Mb)	Total Scaffold Length / Estimated Genome Size (%)	Length Scaffold Sequences >=25K nt (Mb)	Scaffold Sequences >=25K nt / Estimated Genome Size (%)	%N
A. hypogaea	2.87	[86]	2806	384	2560	91.12	2550	91.01	0.15
B. distachyon	0.32	[87]	313	11	272	87.00	272	87.00	0.19
C. sativa	0.84	[72]	821	221	876	106.72	875	106.62	15.93
G. max	1.13	[88]	1105	282	978	88.62	977	88.48	2.65
G. raimondii	0.9	[89]	880	1033	761	86.52	755	85.77	1.75
H. vulgare	7.33	[90, 91]	7167	8	4830	67.45	4830	67.45	5.44
M. truncatula A17	0.47	[92]	460	2186	413	89.93	403	87.77	5.53
M. truncatula R108	0.47	[92]	460	909	402	87.60	395	86.09	0.68
O. sativa	0.5	[90]	489	63	375	76.85	375	76.74	0.03
P. vulgaris	0.6	[93]	587	478	537	91.68	534	91.12	1.05
S. bicolor	1.2	[94]	1173	870	709	60.43	705	60.08	4.72
T. aestivum	17.3	[90]	16916	22	14500	86.00	14500	86.00	1.90
V. unguiculata	0.6	[95]	587	686	519	88.64	518	88.38	0.00
Z. mays B73	2.7	[90]	2640	266	2130	80.85	2130	80.85	1.44
Z. mays Mo17	2.7	[90]	2640	2208	2180	82.68	2170	82.06	1.61
Z. mays PH207	2.7	[90]	2640	43291	2160	81.67	2090	79.25	19.61
Z. mays W22	2.7	[90]	2640	11	213E0	80.83	2130	80.83	1.90

Download this table (CSV)

* The extended assembly statistics are found in Table S1.

Table V: Structural annotation table directly from GenomeQC.

	Gene Models	Min. Gene Length (bp)	Max. Gene Length (bp)	Avg. Gene Length (bp)	Gene Models <200 bp	Transcripts	Avg. Transcripts per Gene Model	Exons	Avg. Exons per Transcript	Avg. Exon Length (bp)
A. hypogaea	67128	163	342359	3972.3	48	84714	1.3	423763	4.8	296.4
B. distachyon	36647	90	47411	3012.5	175	47917	1.3	263865	5.5	314.1
C. sativa	29815	21	976063	3450.0	2187	34876	1.2	234157	6.6	291.9
G. max	52872	90	94733	4017.0	1363	86256	1.6	-	-	-
G. raimondii	37505	90	51175	3243.5	71	77267	2.1	527563	6.7	271.3
H. vulgare	-	-	-	-	-	248270	-	1715898	-	278.9
M. truncatula A17	51541	60	102191	2566.9	1985	74213	1.4	318421	4.4	313.2
M. truncatula R108	55706	72	62996	2232.4	2404	61019	1.1	220904	3.6	270.6
O. sativa	35825	72	304271	3098.7	57	42378	1.2	192499	4.5	350.3
P. vulgaris	27433	93	90772	3943.8	108	36995	1.3	-	-	-
S. bicolor	34129	96	88337	3713.2	56	47121	1.4	266301	5.6	350.9
T. aestivum	107891	54	124945	3488.9	686	133744	1.2	749233	5.8	303.3
V. unguiculata	29773	90	81066	3880.9	408	42287	1.4	-	-	-
Z. mays B73	39324	111	128402	4117.8	38	151959	3.9	1488381	9.7	281.5
Z. mays Mo17	38620	21	146217	4076.8	1225	46530	1.2	272323	5.9	297.1
Z. mays PH207	40557	111	714207	5993.3	53	40557	1.0	208149	5.1	252.1
Z. mays W22	40691	87	154495	4330.4	64	51717	1.3	313830	6.1	292.4

Download this table (CSV)
Dashes (-) represent missing data.
Missing data in *H. vulgare* is due to the absence of gene model information in the GFF file.
Missing data in *G. max*, *P. vulgaris*, and *V. unguiculata* is due to the absence of exon information in their GFF files.

IX. SUPPLEMENTARY

Supplementary Table SI: Additional assembly statistics from GenomeQC.

	Longest Scaffold (bp)	Shortest Scaffold (bp)	Scaffolds >1K nt	Scaffolds >10K nt	Scaffolds >100K nt	Scaffolds >1M nt	Scaffolds >10M nt	N50	L50	NG50	LG50	%A	%C	%G	%T
A. hypogaea	1.61E+08	1000	382	299	42	23	20	1.35E+08	9	1.35E+08	10	31.79	18.14	18.13	31.79
B. distachyon	7.55E+07	2.88E+04	11	11	5	5	5	5.89E+07	3	5.89E+07	3	26.76	23.16	23.14	26.76
C. sativa	1.05E+08	6741	221	218	52	15	10	9.19E+07	5	9.19E+07	5	27.94	14.07	14.11	27.95
G. max	5.83E+07	885	281	271	63	20	20	4.99E+07	10	4.78E+07	11	31.77	16.91	16.91	31.77
G. raimondii	7.07E+07	1001	1033	200	20	14	13	6.22E+07	6	6.10E+07	7	32.81	16.32	16.32	32.81
H. vulgare	7.68E+08	2.50E+08	8	8	8	8	8	6.57E+08	4	5.83E+08	6	26.27	21.02	21.01	26.26
M. truncatula A17	5.66E+07	1004	2186	605	51	8	8	4.92E+07	4	4.57E+07	5	31.60	15.66	15.65	31.57
M. truncatula R108	3.23E+07	1007	909	481	72	49	18	1.28E+07	12	1.23E+07	14	33.25	16.41	16.41	33.25
O. sativa	4.33E+07	4236	63	51	17	12	12	3.00E+07	6	2.90E+07	8	28.21	21.77	21.78	28.21
P. vulgaris	6.30E+07	1005	478	290	61	13	11	4.97E+07	5	4.80E+07	6	31.84	17.61	17.64	31.86
S. bicolor	8.09E+07	1005	870	232	69	12	10	6.87E+07	5	6.87E+07	9	26.74	20.90	20.91	26.73
T. aestivum	8.31E+08	4.74E+08	22	22	22	22	22	7.10E+08	10	6.74E+08	12	26.46	22.59	22.59	26.46
V. unguiculata	6.53E+07	2922	686	679	103	13	11	4.17E+07	6	4.13E+07	7	33.31	16.40	16.42	33.34
Z. mays B73	3.07E+08	5568	266	261	48	12	10	2.24E+08	5	1.82E+08	6	26.18	23.09	23.10	26.19
Z. mays Mo17	3.06E+08	1007	2208	1864	129	11	10	2.20E+08	5	1.83E+08	6	26.14	23.03	23.05	26.17
Z. mays PH207	3.03E+08	500	16840	1115	61	11	10	2.15E+08	5	1.76E+08	6	21.34	18.40	18.40	21.36
Z. mays W22	3.11E+08	1.25E+07	11	11	11	11	11	2.23E+08	5	1.83E+08	6	26.11	22.92	22.94	26.13

Download this table (CSV)

N50: The length of the shortest scaffold/contig in the list of L50 sequences.

L50: The number of sequences whose sum of lengths make up 50% or more of the total assembly length.

NG50: The length of the shortest scaffold/contig calculated in the same manner as N50 but based on estimated genome size rather than total assembly length.

LG50: The number of sequences whose sum of lengths make up 50% or more of the estimated genome size.

Supplementary Table SII: Number of removed annotations during cleanup.

Genome	Dataset	Obsolete Annotations	Duplicates	Annotations with Modifiers
Arachis hypogaea	GOMAP	3437	13	912
Brachypodium distachyon	GOMAP	2512	49	789
	Gold Standard Gramene 63 (no IEA)	21	204	0
	Gramene63 (IEA only)	166	114	0
	Phytozome12	99	18	0
Cannabis sativa	GOMAP	1714	6	757
Glycine max	GOMAP	3333	10	930
Gossypium raimondii	GOMAP	1781	7	822
Hordeum vulgare	GOMAP	1877	8	815
	Gold Standard Gramene 63 (no IEA)	1	9	0
	Gramene63 (IEA only)	282	147	0
Medicago truncatula A17	GOMAP	2673	10	798
	Gold Standard Gramene 63 (no IEA)	2	23	0
	Gramene63 (IEA only)	309	243	0
	Phytozome12	132	17	0
Medicago truncatula R108	GOMAP	4168	7	803
Oryza sativa	GOMAP	1642	7	869
	Gold Standard Gramene 63 (no IEA)	37	833	0
	Gramene63 (IEA only)	238	64	0
	Phytozome12	119	19	0
Phaseolus vulgaris	GOMAP	1190	6	783
Pinus lambertiana	GOMAP	1839	4	587
Sorghum bicolor	GOMAP	2384	66	783
	Gold Standard Gramene 63 (no IEA)	178	219	0
	Gramene63 (IEA only)	278	198	0
	Phytozome12	131	12	0
Triticum aestivum	GOMAP	9624	17	1132
	Gold Standard Gramene 63 (no IEA)	1	5	0
	Gramene63 (IEA only)	584	319	0
Vigna unguiculata	GOMAP	1269	6	811
	Phytozome12	122	27	0
Zea mays B73.v4	GOMAP	2077	89	848
	Gold Standard Gramene 63 (no IEA)	50	633	0
	Gramene63 (IEA only)	306	140	0
Zea mays Mo17	GOMAP	2346	83	823
	Gold Standard Gramene 63 (no IEA)	36	1489	0
Zea mays PH207	GOMAP	2676	82	830
	Gold Standard Gramene 63 (no IEA)	37	2702	0
Zea mays W22	GOMAP	2681	88	840
	Gold Standard Gramene 63 (no IEA)	30	499	0

Download this table (CSV)

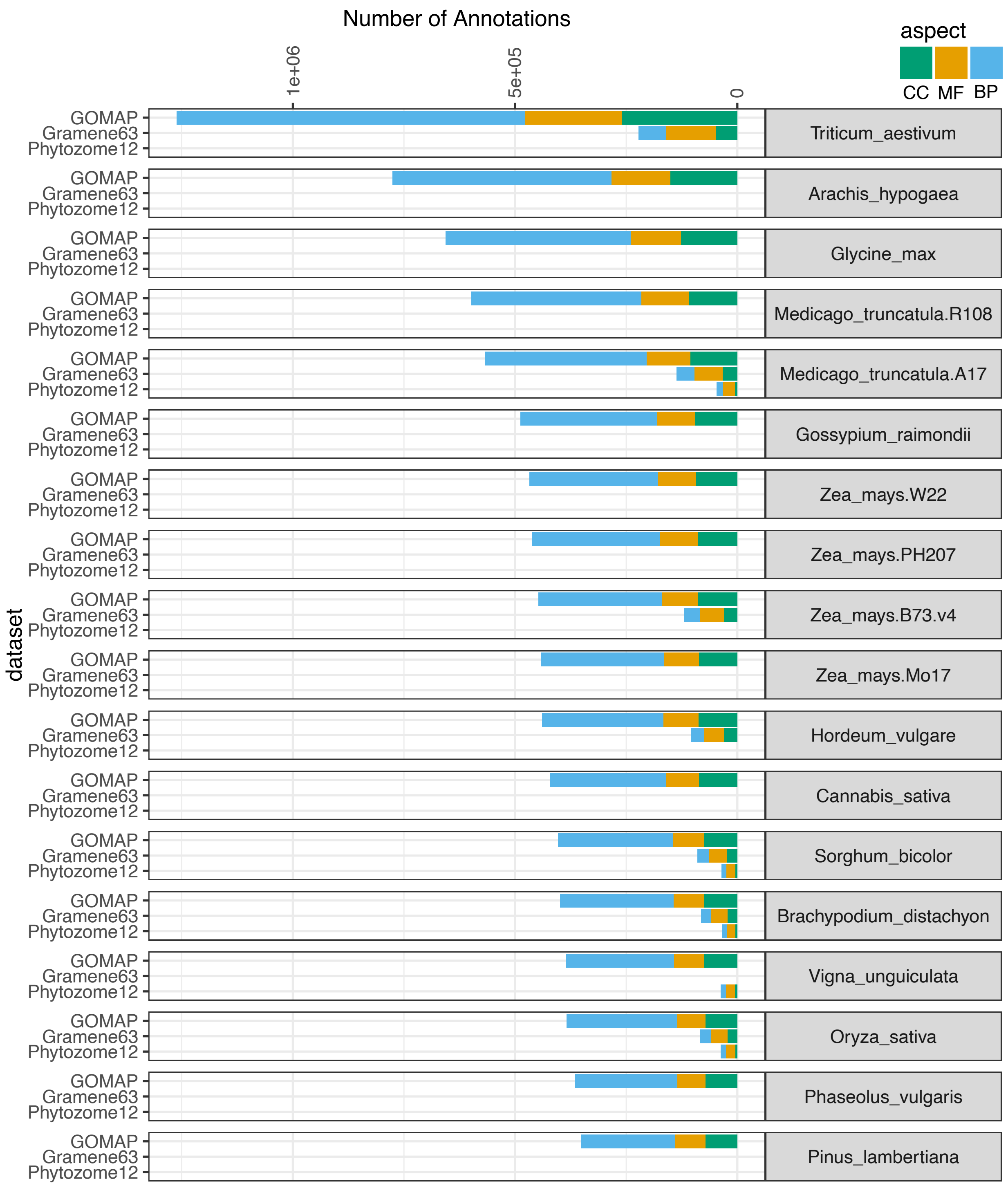

Supplementary Figure S1: Number of total annotations in each GO IEA dataset analyzed, colored by GO aspect (Cellular Component in green, Molecular Function in orange, and Biological Process in blue). Species are ordered based on the number of GO terms in the GOMAP dataset, with the species producing the most GO terms (*Triticum*) on top and the least (*Pinus*) on bottom.

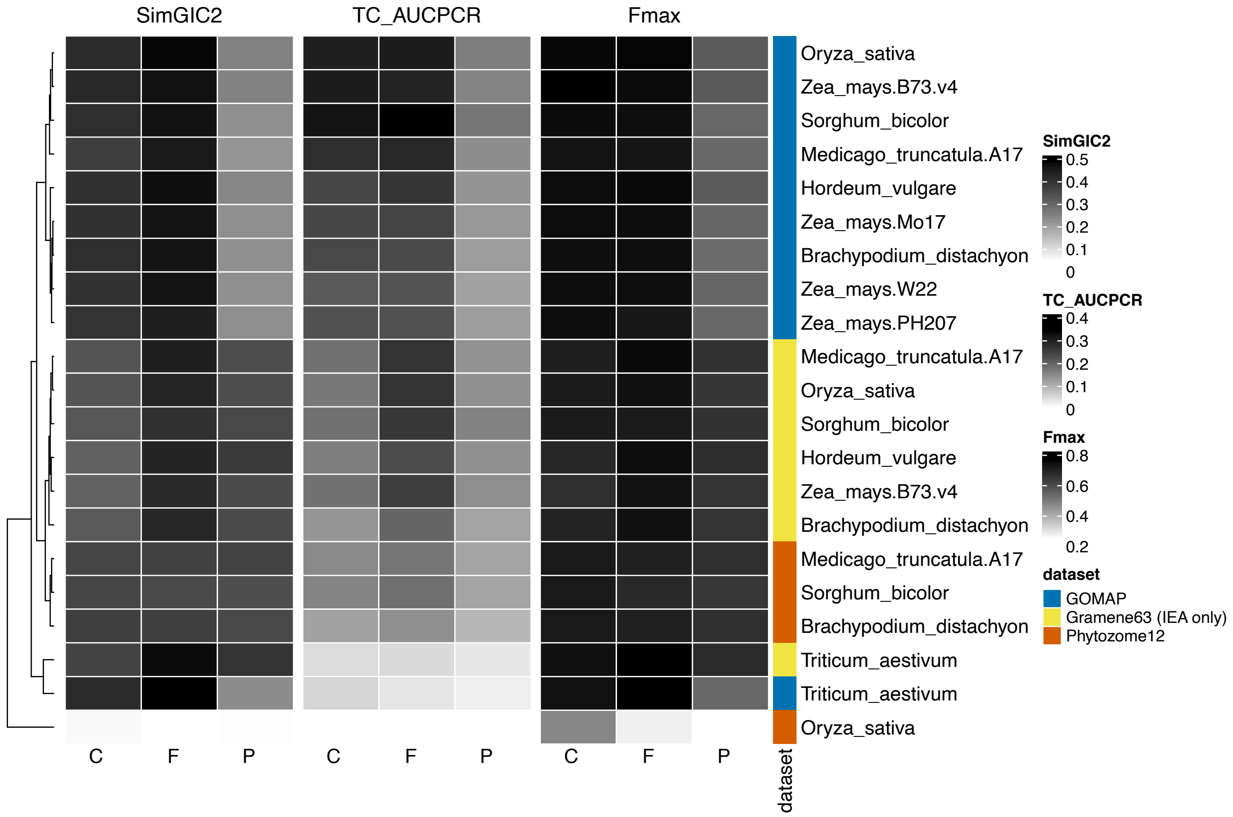

Supplementary Figure S2: Quality scores of the predicted annotation visualized as a grayscale heatmap. Each row represents a single species from a single data source. Input gene model dataset origin is indicated - blue for GOMAP, yellow for Gramene63, and orange for Phytozome12. Across the top are the three metric types used to assess the annotation quality. Across the bottom are subgraph indicators: C is Cellular Component, F is Molecular Function, and P is Biological Process. Darker cells indicate a higher (better) score whereas lighter cells indicate a lower score. Note that scales are different for each metric type (meaning that comparisons across the three metric types are not meaningful). Rows are clustered by pairwise correlation/similarity across all metrics. The dendrogram at left retraces clustering.

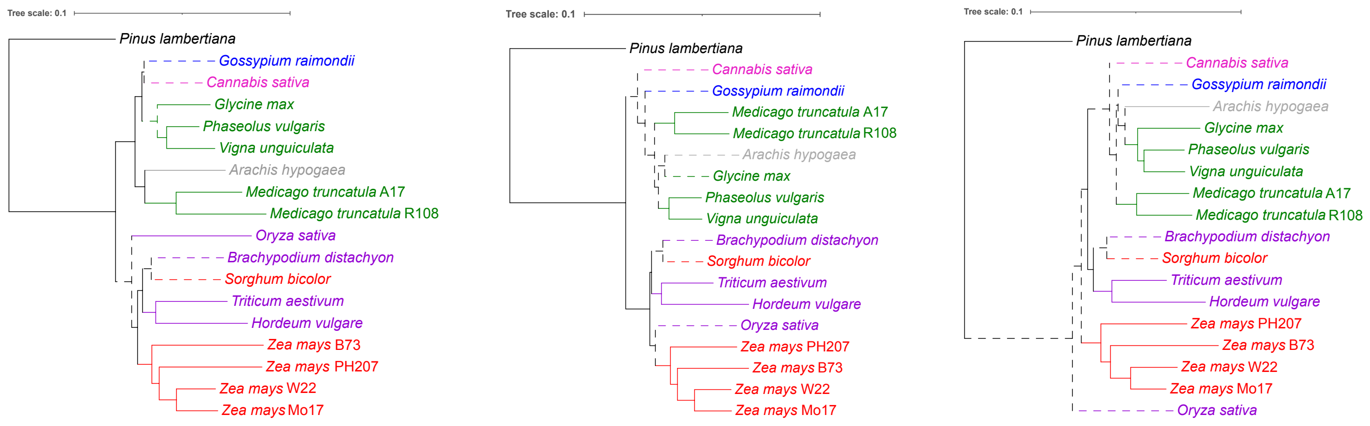

(a) Cellular Component (b) Molecular Function (c) Biological Process

Supplementary Figure S3: Neighbor-joining trees built on annotation subsets per GO aspect. Phylograms are colored and rooted as described in Figure 2. For each tree, only the annotations from the respective aspect of the Gene Ontology were used.

Figure 1 [Click here to access/download;Figure;Figure1.pdf](#)

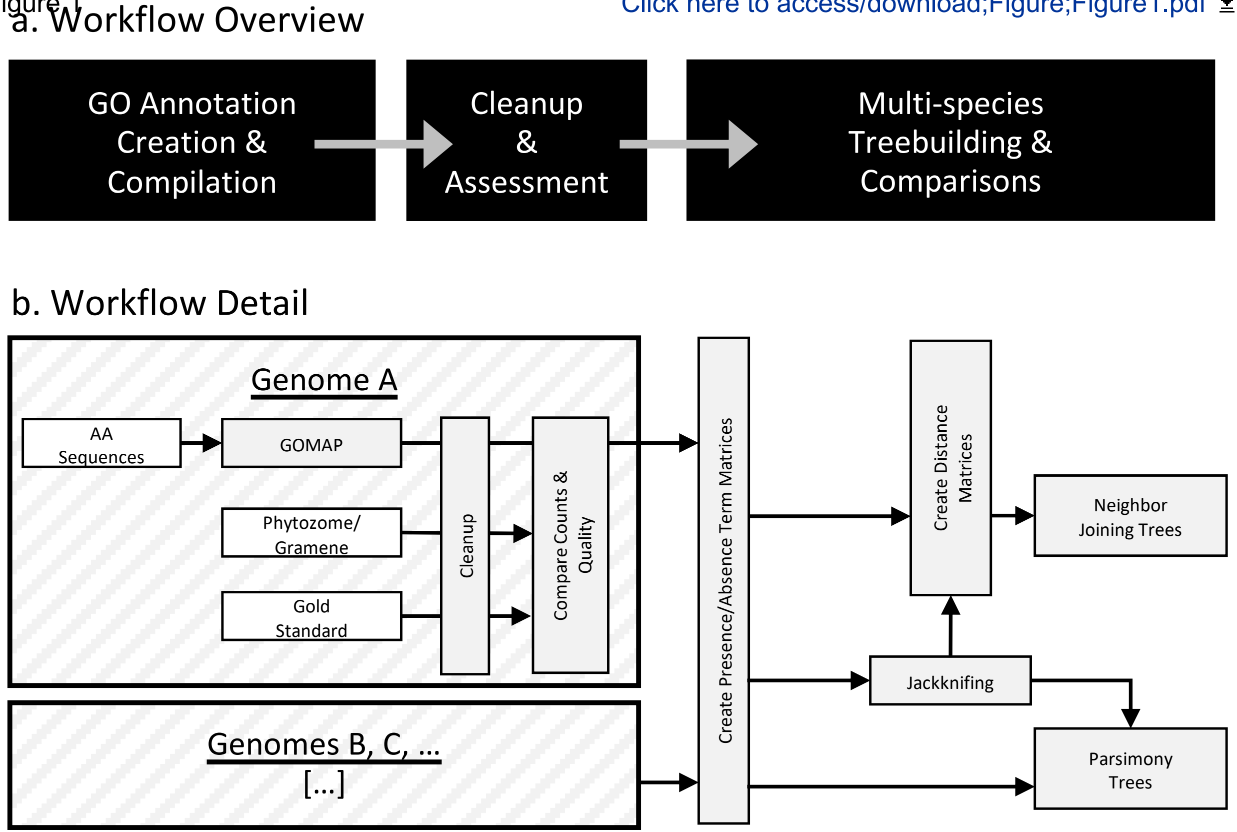

Figure 2

Click here to access/download;Figure;Figure2.pdf

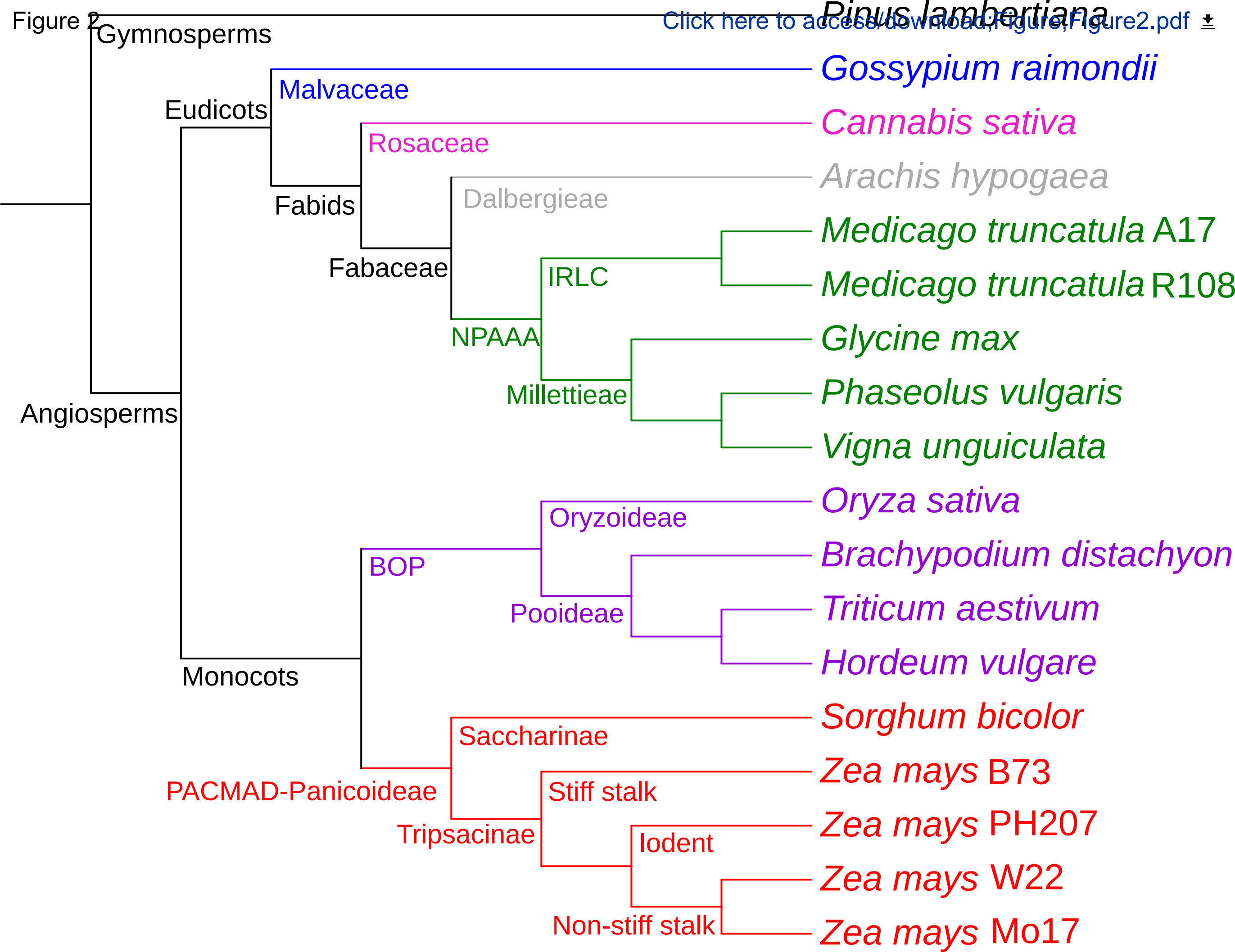

Figure 3a

Click here to access/download;Figure;Figure3a.pdf

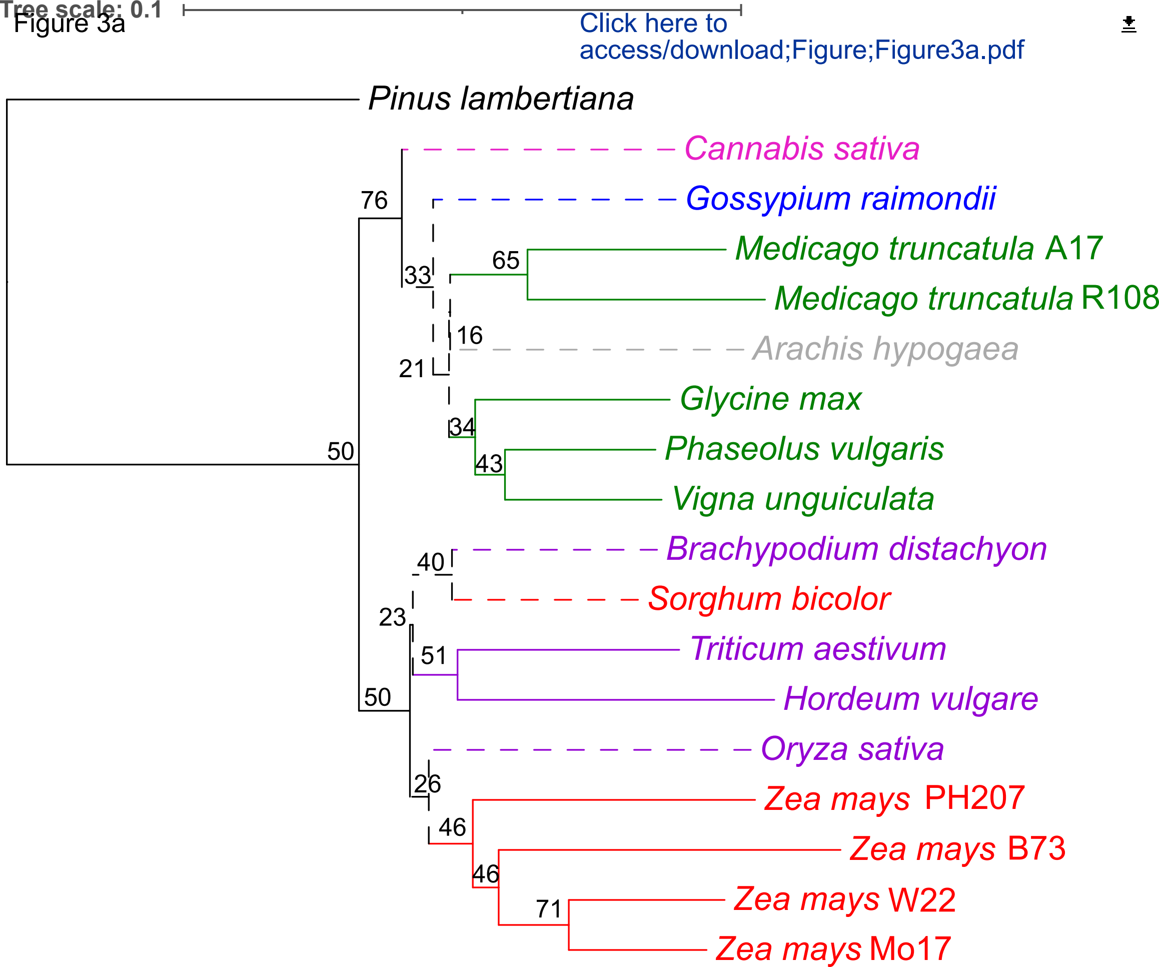

Figure 3b

Click here to access/download;Figure;Figure3b.pdf

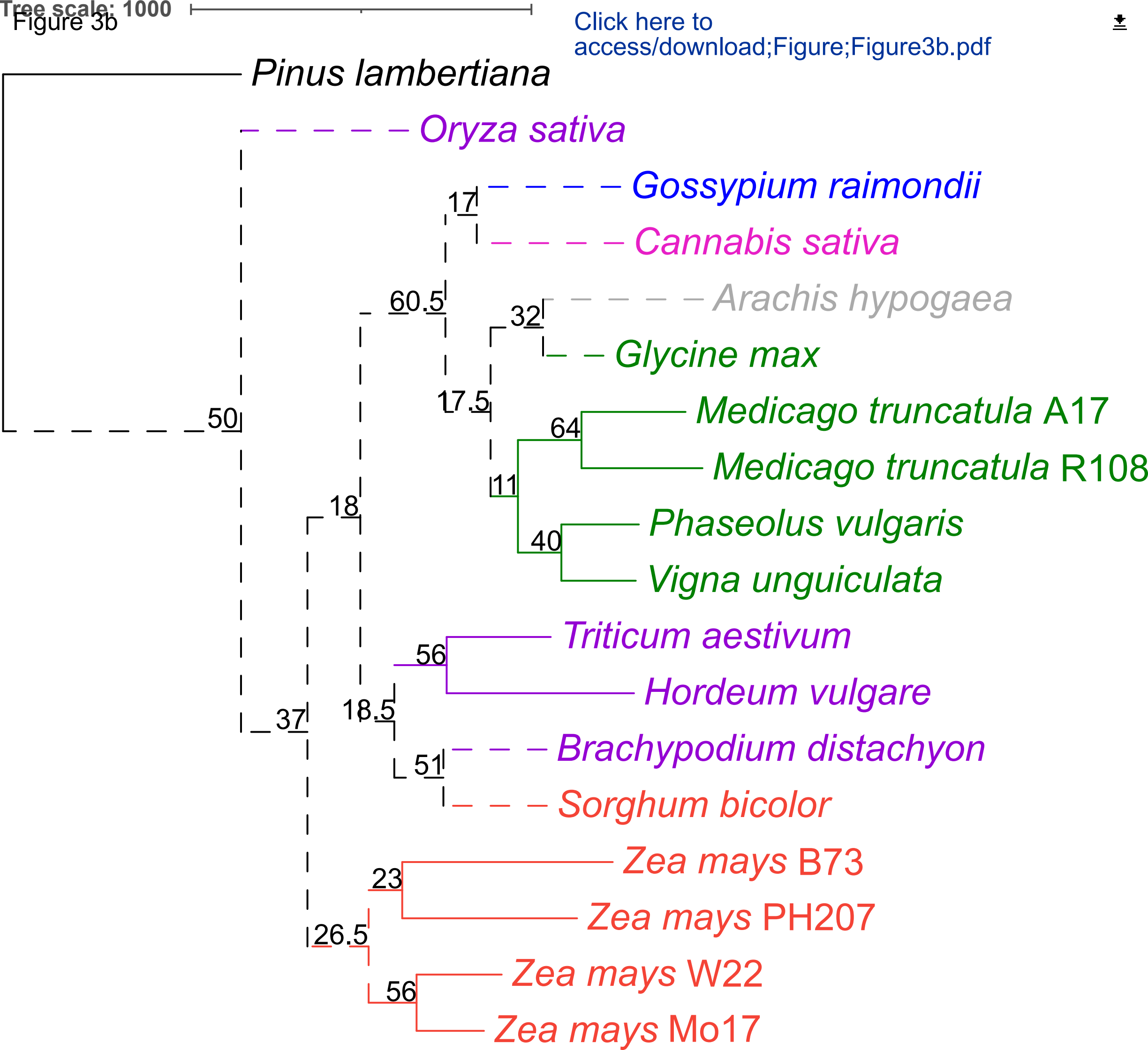

Figure 4a

Click here to access/download;Figure;Figure4_Oryz

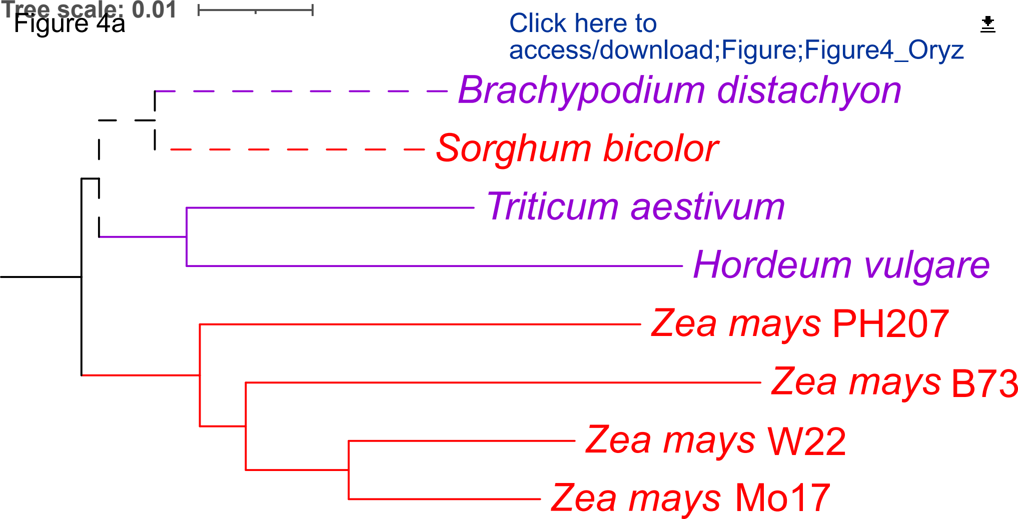

Figure 4b

Click here to access/download;Figure;Figure4_Sorg

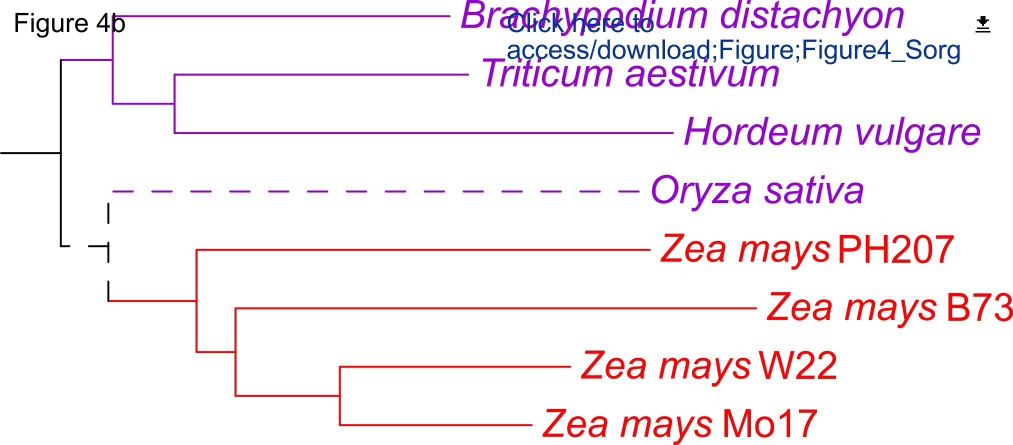

Figure 4c

Click here to access/download;Figure;Figure4_Brach

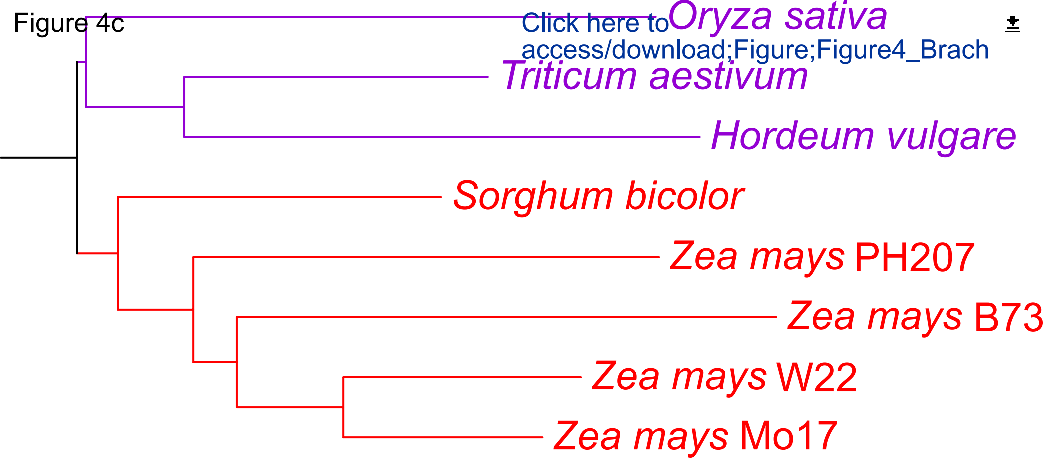

Figure 5

Click here to access/download;Figure;Figure5.pdf

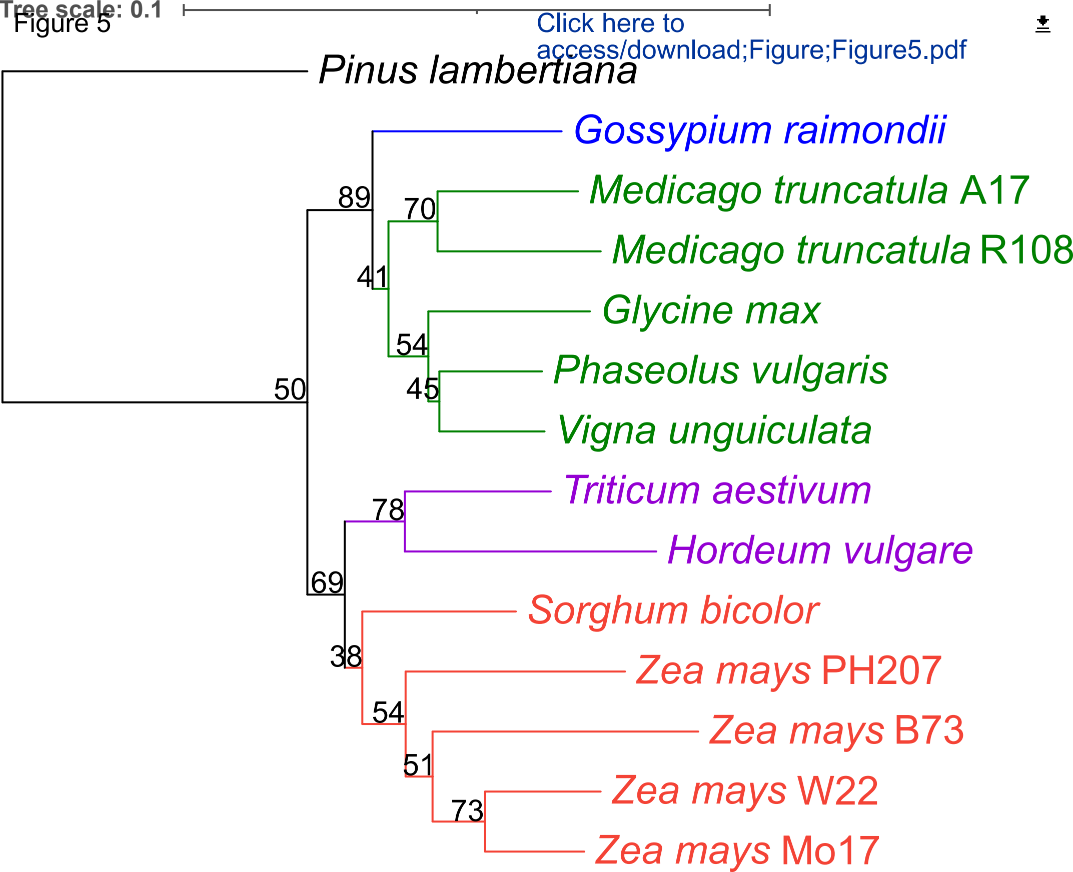

Figure 6

Click here to access/download;Figure;BUSCO_assemblyR.pdf

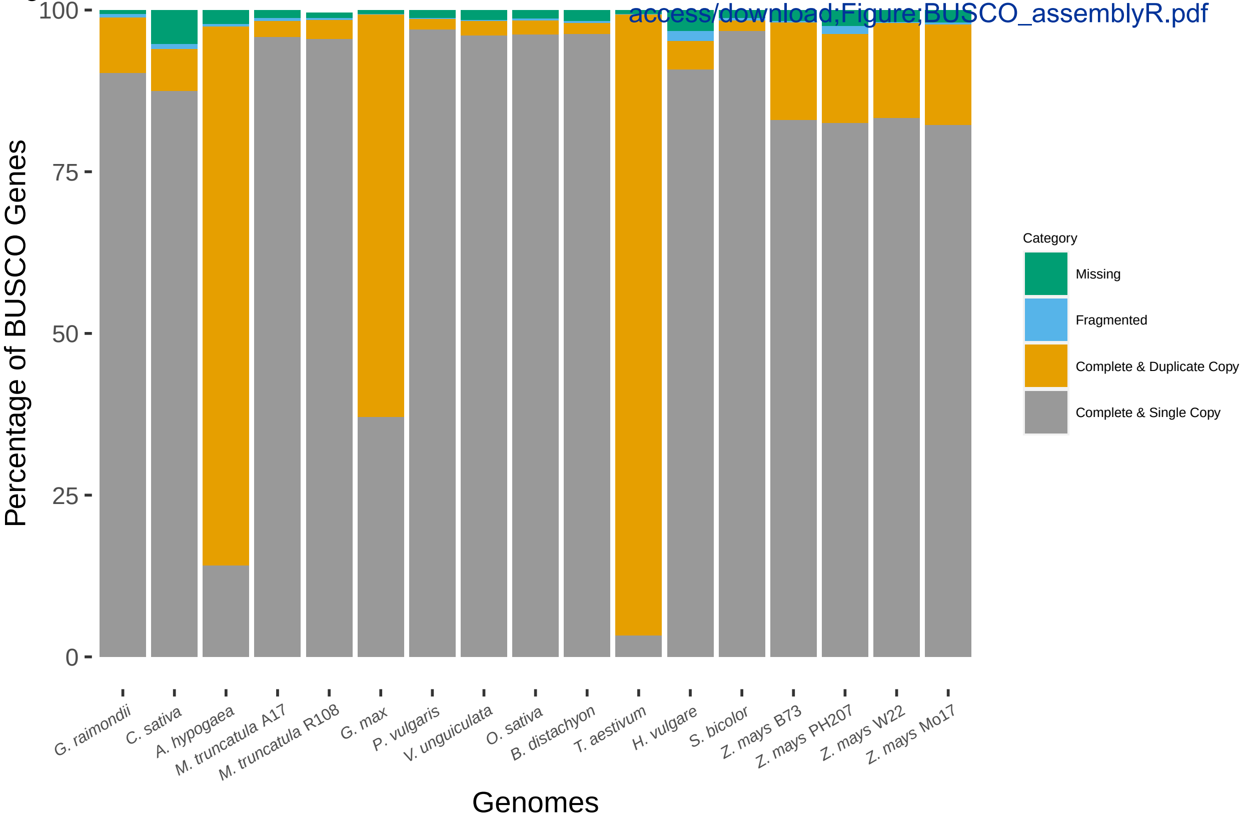

Figure 7

Click here to access/download;Figure;BUSCO_annotationR.pd

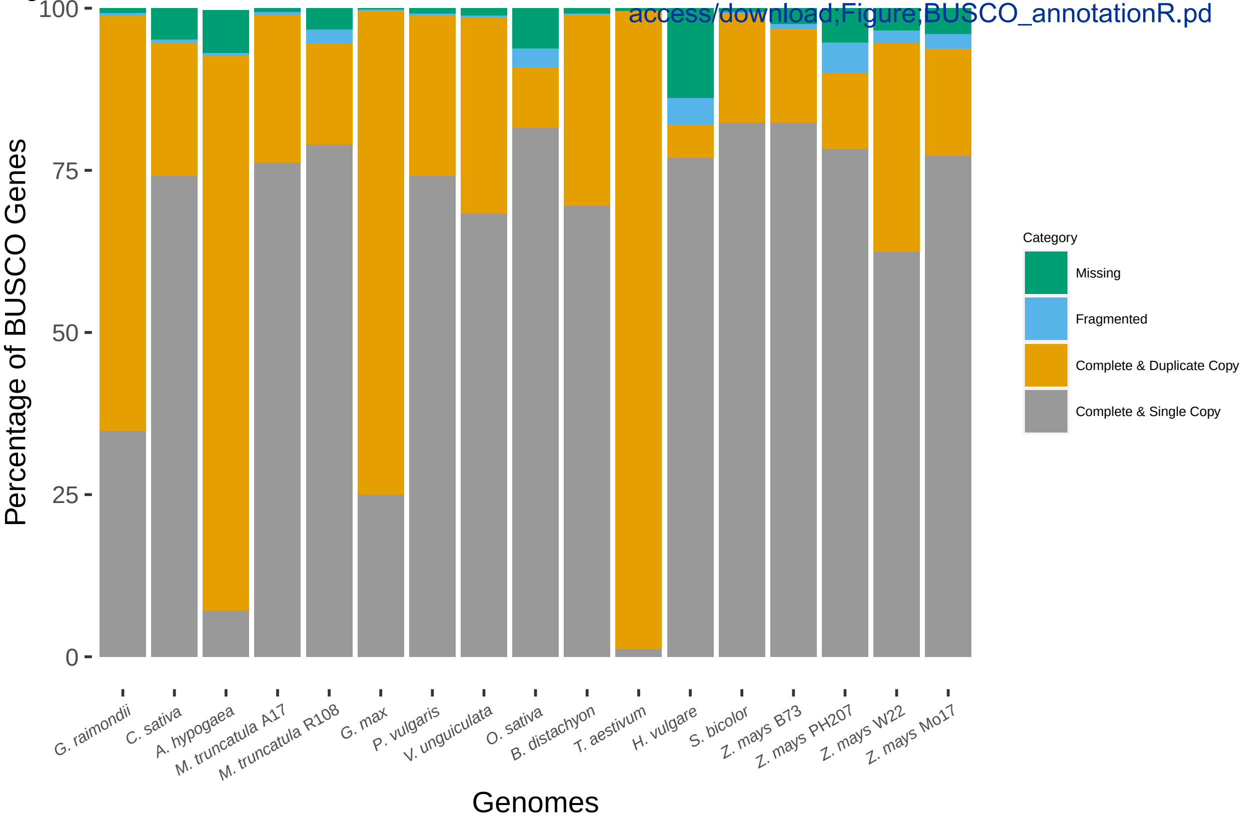

Supplemental Figure 1

Click here to access/download

Supplementary Material

FigureS1.pdf

Supplemental Figure 2

Click here to access/download
Supplementary Material
FigureS2.pdf

Supplemental Figure 3a

Click here to access/download
Supplementary Material
FigureS3a.pdf

Supplemental Figure 3b

Click here to access/download
Supplementary Material
FigureS3b.pdf

Supplemental Figure 3c

Click here to access/download
Supplementary Material
FigureS3c.pdf

Click here to access/download;Personal Cover;GOMAP Trees Reviewer Response.pdf

Thank you for giving us the opportunity to revise our submission.

Please find below responses to reviews, with reviewer comments in green and responses in black. Note that where changes to the manuscript are described, those are shown in a version of the manuscript pdf with highlighting as follows: text in blue has been moved, text in green has been added, and text in the figures can be ignored (these highlights are because the figures contain text now and in the previous version the figure-text was an image (pixels) not actual text).

Reviewer #1

The idea of recapitulation of phylogenetic relationships based on GO annotations is an interesting one and while authors do a good of addressing some of the caveats and limitations of their analysis I do wonder if there are other things that they may want to consider. For example a lot of plant annotations are based off of Arabidopsis experimental annotations, which means that some aspects of plant biology that are unique to specific clades may not be well represented in the ontology because those processes have not been annotated or the terms may not even exist in the ontologies yet. Also, at least for Arabidopsis, many of the included annotations come from PAINT which is a phylogenetic based annotation method (the IBA annotations) so transferring IBA annotations from Arabidopsis to other plant species might add a certain phylogenetic flavor the GOMAP results.

This discussion of how the the GO graph is influenced by presence of processes in Arabidopsis is an excellent suggestion. A paragraph to this effect has been added to the manuscript so that readers also understand this limitation to the GO graph itself for plants. See also the answer to Reviewer #1 point 4.

1.Please clarify what the sets of terms used were and what is meant by ancestors? The MS states, granular terms were mapped to higher order terms used for comparison- how were those ancestor terms selected? is there a list of these common (S) terms that were used to generate the trees available somewhere? If so, that subset should be made available (or maybe it is but I could not tell.) I think this selection of terms for use in the analysis is really important but could not find any data for this- if the data is available it is not obvious.

By ancestral terms, we mean all terms that are proximal to a given term, i.e., all parental terms (connected via *is_a* in the ontology) as well as their respective parents, up until the very root of the ontology (e.g. GO:0008150, biological process, for the biological process graph). For example, if “GO:0044711, Single-organism biosynthetic process” is the term present in an annotation set T, all the terms shown below would be added as ancestral terms:

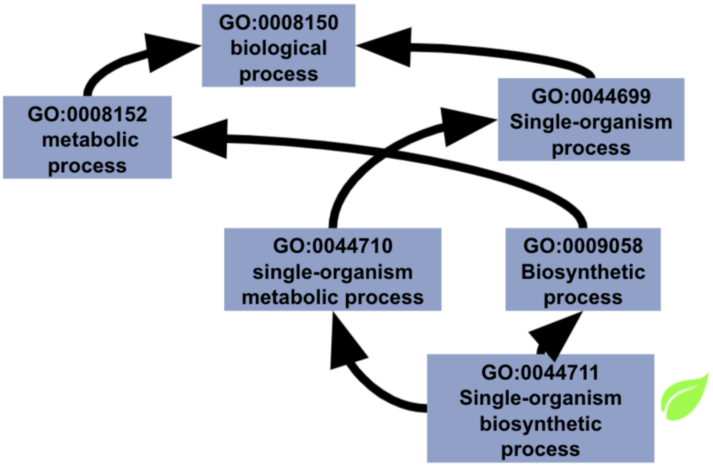

We have now clarified this in the manuscript in the first paragraph of part C of Methods and have made the sets with added ancestors available on our GitHub repository (https://github.com/Dill-PICL/GOMAP-Paper-2019.1/tree/master/analyses/treebuilding/results/sets).

2.Annotations with modifiers that were removed- can you clarify what is meant by that , are those 'Not' annotations?

Yes, annotations with NOT, contributes_to, or colocalizes_with modifiers were removed. We have added this clarification in the manuscript revision to the first paragraph of part A in the Methods section.

3.One expects a high level of granularity for manually curated gene functions (that is very specific terms) how are annotations harmonized across the different prediction methods used for GOMAP since presumably some of the methods employed provide less specificity in their annotations?

This has been added to the first paragraph of the Methods section: "Details of how GOMAP annotations are derived including the specificity of component datasets and which terms are retained are described elsewhere (2018; https://doi.org/10.1002/pld3.52 and Wimalanathan and Lawrence-Dill 2021;https://doi.org/10.1186/s13007-021-00754-1). In brief, GOMAP annotations are a combination of the annotations from multiple sources. GOMAP combines the annotations from all the sources and removes the less specific annotations that could be inferred from the more specific annotations, keeping only the most specific terms for each gene that cannot be inferred from other terms (i.e., only leaf terms)."

4. For the comparison, was there any manual inspection of presence or absence of terms? Was there any correspondance with anything known biologically? That is for certain term character states, were there any unexpected or inconsistent with biology?

That is a great question. We did inspect the uniquely present/absent GO terms by clade and by species, both computationally and manually. We even hypothesized that we should be able to extract biologically meaningful terms for specific cases. For example we hypothesized that we could find unique terms for C4 vs C3 plants or monocots vs dicots and that those terms might have biological meaning for those traits. While we were able to find differences, it was difficult to identify and interpret any biological significance, likely because of a point you mention yourself in your first comments: there is a lack of good representation

of GO terms for diverse plants processes because plant-specific terms in the GO graph itself are largely based on functions derived from Arabidopsis. It has proven to be challenging for us to identify biologically meaningful terms that are specific to certain clades in our trees. We should also mention here that we have not limited our use of the gene ontology directed acyclic graph to plant specific terms or removed any non-plant terms (e.g., terms related to blood, nerves, etc.), because certain GO terms that may seem unrelated to plant processes could inspire understanding of plant processes (e.g., terms that are relevant to nerve cell branching could be relevant to trichome branching in plants given that each is a single-celled complex structure). We have added these observations and caveats to the Discussion section of the manuscript.

5.The phylogenetic analysis seems to factor in all 3 GO aspects, have the authors compared results using just a single aspect (process function or component?) Process is notoriously noisy and annotations can be subject to a lot of interpretation. It is also probably the most incomplete data set,

That is a very good point and thank you for this suggestion. Based on your suggestion we went ahead and generated phylogenetic trees based on each of the three graphs. In the figure below, we generated the neighbor-joining trees using (a) cellular component GO terms, (b) molecular function GO terms, and (c) biological process GO terms. Of the 14,303 total GO terms, 1,524 are cellular component terms, 3,926 are molecular function terms, and 8,853 are biological process terms. Out of the three phylogenies below, the one built using molecular function terms (b) is the closest to our neighbor-joining tree obtained using all GO terms in our datasets. The only difference is that *A. hypogaea* and *G. max* are clustered in (b), while they were not in our NJ tree (Fig. 3a) . In (a), *G. raimondii* and *C. sativa* were clustered together when they were not in the NJ tree. Also, *O. sativa* was at the base of the monocots just like in the expected tree, but not in the NJ tree. In (c), *O. sativa* was at the base of the angiosperms and there was no clear separation between monocots and dicots. In all the three phylogenies presented below and our NJ tree, *A. hypogaea* was never placed at the base of the NPAAA clade as would be expected. Also, *B. distachyon* and *S. bicolor* were always clustered together. Like the trees generated from the full GO dataset, the topologies constructed using one GO term aspect at a time failed to restore the topology of the expected tree. We added this to our manuscript in part C "Phylogenetic Tree Analyses" of the Results section.

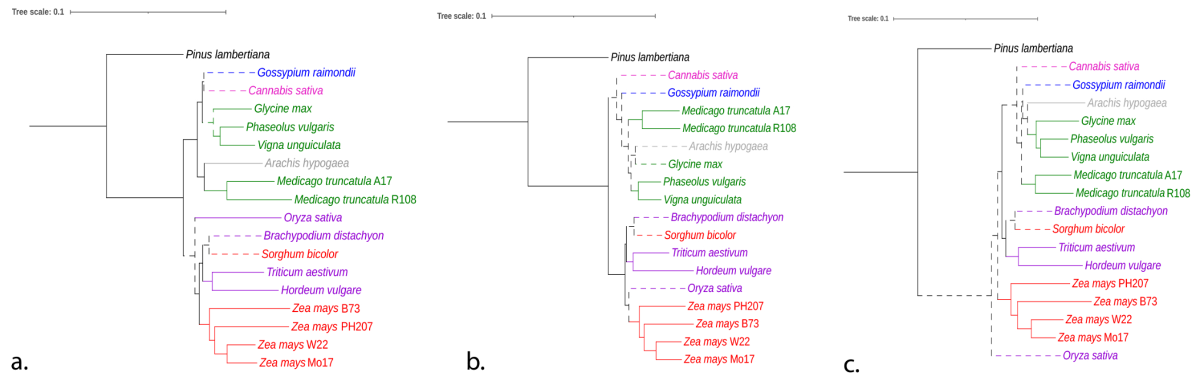

Specific comments on the figures.

1. Panel b. Gramene -IEA is confusing here in the figure and when described elsewhere. I suggest that in the figure, and the text, using less confusing nomenclature such as Gramene

(IEA only) and Gramene (no IEA) for gold standard. To me I read Gramene-IEA as Gramene minus IEA annotations and not Gramene's IEA annotations only.

Thank you for pointing this out. We have renamed Gramene63-IEA to "Gramene63 (IEA only)" and for the Gold Standard we now call it "Gold Standard Gramene 63 (no IEA)" throughout the text and in tables.

2.Supplementary Figure S1.

I wonder if there is a more effective way to visualize this data. I think there is a lot of interesting information here but it is hard to follow , especially the third graph. Another improvement to readability would be to make the text font darker (not sure why it is light grey.)

Thank you for pointing this out and suggesting changes. We replaced that figure with the new Supplementary Figure S2 heatmap shown below. We hope this figure is easier to follow.

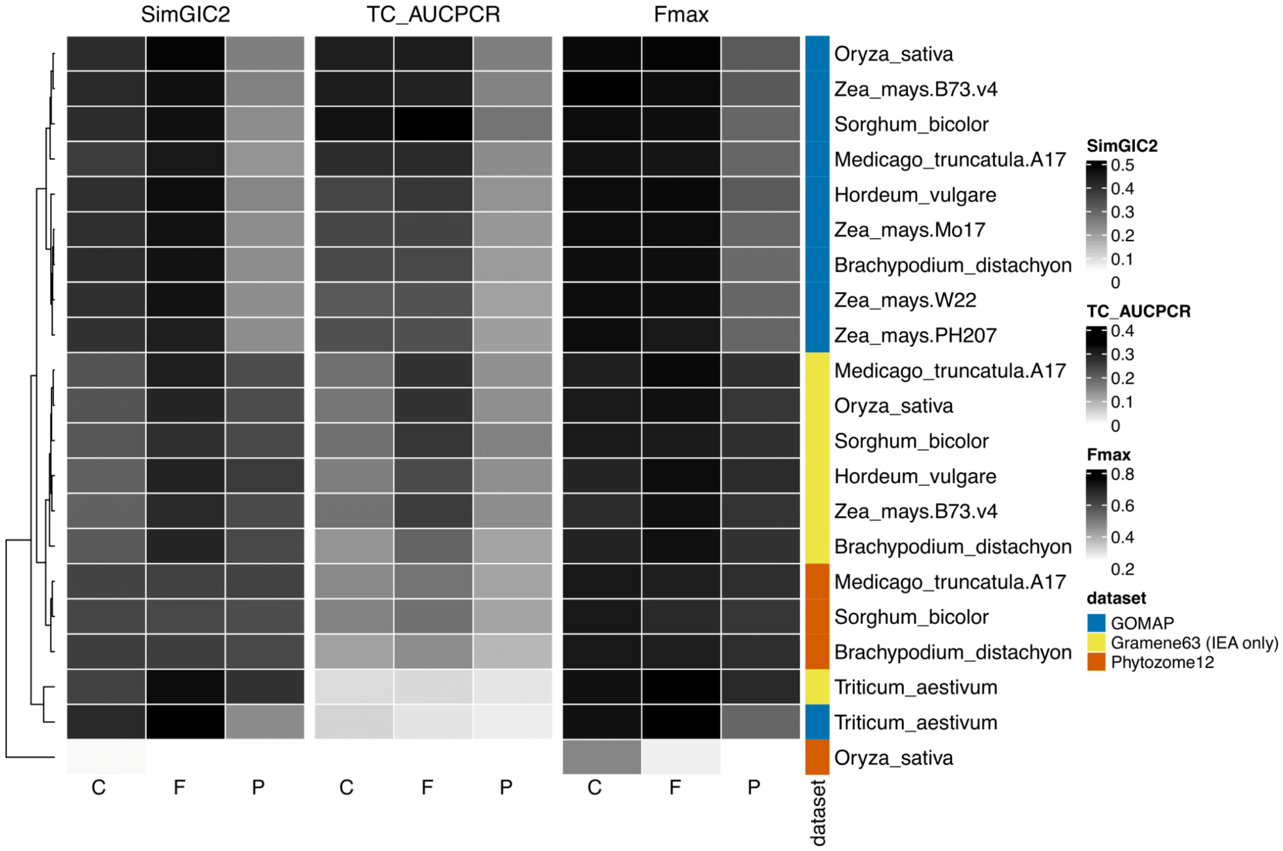

Supplementary Figure 2: Quality scores of the predicted annotation visualized as a grayscale heatmap. Each row represents a single species from a single data source. Input gene model dataset origin is indicated - blue for GOMAP, yellow for Gramene63, and orange for Phytozome12. Across the top are the three metric types used to assess the annotation quality. Across the bottom are subgraph indicators: C is Cellular Component, F is Molecular Function, and P is Biological Process. Darker cells indicate a higher (better) score whereas lighter cells indicate a lower score. Note that scales are different for each metric type (meaning that comparisons across the three metric types are not meaningful). Rows are clustered by pairwise correlation/similarity across all metrics. The dendrogram at left retraces clustering.

Reviewer #2

The authors present a "Standardized genome-wide function prediction enables comparative functional genomics: a new application area for Gene Ontologies in plants". It seems to be an application of the GOMAP pipeline in 14 species which sounds to be interesting. However, it lacks polish, thats why I list some suggestions to help the authors improve it.

* Major:

1-) It is not clear the state-of-art tools in this topic (this is not detailed in introduction, which is a serious gap in this work), including similar or same tools/methods for the same purpose. Keep this in mind, please, compare against tools from literature in the same issue please. I am not an expert in the GO topic, but as far as I know, there is Blast2GO, and I found others:
https://www.mdpi.com/1999-5903/13/7/172
https://academic.oup.com/nar/article/49/D1/D394/6027812?login=true
https://onlinelibrary.wiley.com/doi/full/10.1111/1755-0998.13285
https://david.ncifcrf.gov/
blast2go
Etc
1.2.) Please, compare against these tools, or those that make sense, if not, why not? Clarify and make clear this, please.

PS: Re-write the introduction to address these points.

These points and comparisons are covered in two previous publications that describe the GAMER/GOMAP system. To draw attention to that work we have edited the third paragraph of the introduction as follows. “We previously annotated gene functions for the maize B73 genome and demonstrated that GOMAP's predicted functions were closer to curated gene-term associations from the literature than those of other community functional annotation datasets, including those produced by Gramene (Ensembl pipeline) and Phytozome (Interpro2GO pipeline) (Wimalanathan et al., 2018). Using the newly containerized GOMAP system (Wimalanathan and Lawrence-Dill 2021), we report here the functional annotation of 18 plant genomes across the 14 crop plant species shown in Table I and report comparisons of performance based on comparison to a Gold Standard gene function datasets, where possible.”

2-) The idea is to do a large-scale analysis of several plant species (in case 14) using the GOMAP pipeline. Is it?
2.1-) Please make clear how many and each of the contributions in the abstract and introduction.
It is not clear to us what this comment means. If it refers to contributions of individuals who are authors, those are listed in part C “Author’s Contributions” of the Declarations section.

2.2-) We now have more than 70 plants species in Ensembl Plants, for example. Why do not use all of them as much as possible for a real large-scale analysis?

The aim of this paper is not to do a large-scale analysis of plants, but rather to describe a proof-of-concept methodology and some caveats for performing comparative functional genomics using GO for plants more generally.

We did not state that the aim of this study was to do a large-scale analysis of plant species. This study was posited as a proof of concept aiming to demonstrate that Gene Ontology datasets are now so complete that comparative functional genomics using gene functions is possible. We chose to use GOMAP rather than datasets derived from Ensembl or other pipelines based on the fact that the datasets are higher coverage, they are of comparable quality, and the pipeline is fully reproducible by other people who wish to sequence and annotate plant genomes. Indeed, the comparative quality of genome functional annotations produced by GOMAP and the Ensembl pipeline (as used to produce the Gramene plant datasets) are directly assessed by our work. The example set of analyses here based on phylogenetics are described as a proof-of-concept and a starting point for future, more sophisticated methods for GO-based functional comparative genomics of plants.

Concerning the use of a larger number of plant species (i.e., 70 for Ensembl) it should be noted that we were performing comparative functional genomics of crop species and our goal was to demonstrate comparative gene functions with high-quality, large-scale datasets.

3-) If I understood, the GitHub (https://github.com/Dill-PICL/GOMAP-Paper-2019.1) and the https://dill-picl.org/projects/gomap/ gomap-datasets/ contains all the data results from this report, is it?
Correct.

3.1.) Both (and mainly GitHub) are far from being user-friendly. It seems the author put the information and that it. Be more clear, what, how, why this information is there, and how to use it. Also, is it clear all commands used (maybe provide a manual on what you have done in a tech aspect)?
Thank you for your comments, the documentation was indeed incomplete. We have now substantially improved it and added README files for all the provided data and analyses. More information about how to run the GOMAP pipeline itself can be found at the corresponding repository/documentation page (https://bioinformapping.com/gomap/master/index.html). This is described in sufficient detail that undergraduate bioinformatics students have annotated functions to plant species genome datasets directly (indeed that aspect is described in the paper itself in the “VIII. Authors’ Information” section).

3.2.) Is there any visualization table, I mean an easy output produced by this analysis? If I want to use this data for my new genome etc, real case, how to use it? how to compare? where? sequence? GO terms, etc? Clarify this, please.
PS: Imagine that is a biologist that wants to use your approach.
We have also made and added the following new figure in the Supplementary material as Supplementary Figure 1, to help in visualizing the number of total annotations in each dataset by GO term aspect.

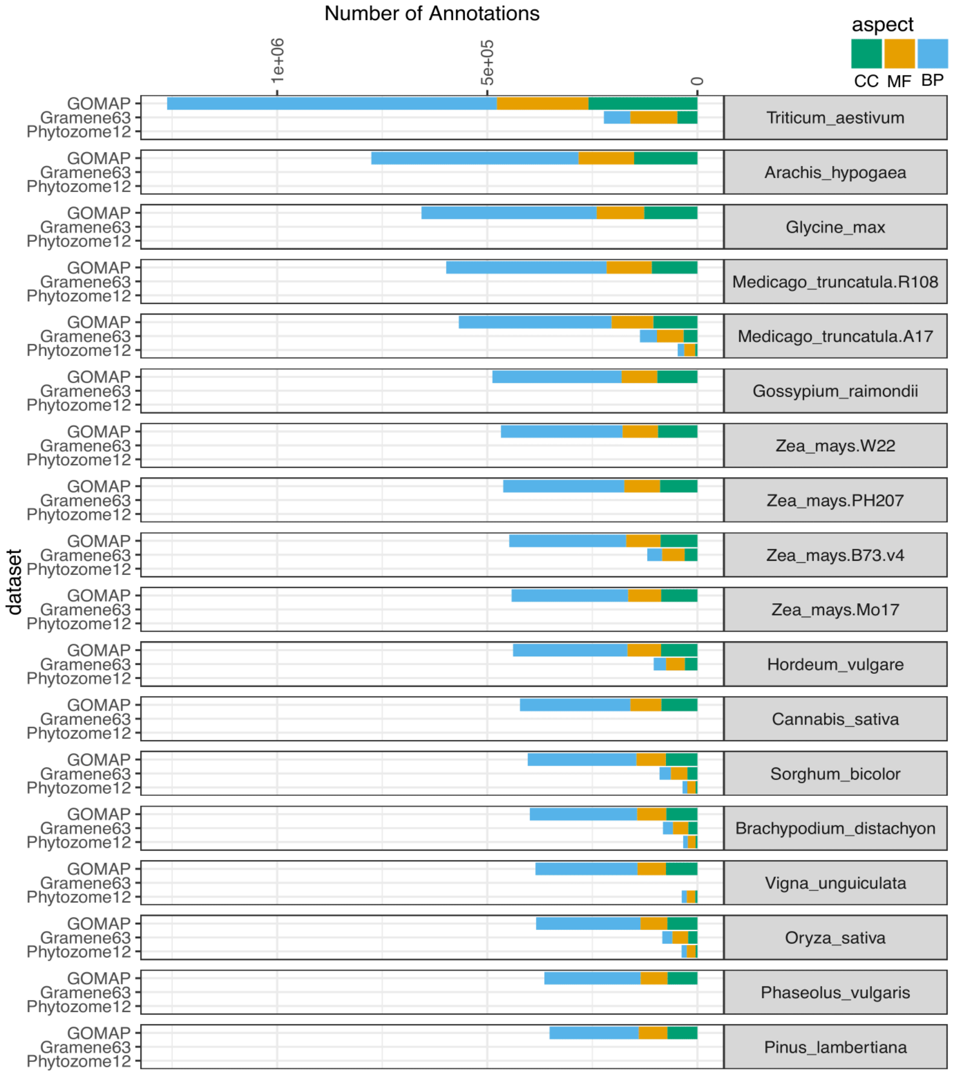

4-) For me is not clear why do not also put Ensembl Plants in this report analysis, and only Phytozome and Gramene. Please, include and compare all these databases.

As described in previous publications and now specified in our introduction, the Gramene annotations are produced by the Ensembl pipeline (indeed, Gramene is the source of data for Ensembl Plants).

5-) Authors mention that they will make available the final results in Zenodo after this revision. Please, make all data, FASTA, trees etc available.

Thank you for mentioning this. All data are already available on our github (https://github.com/Dill-PICL/GOMAP-Paper-2019.1), this link was mentioned in the “Availability of Source Data Code and Supporting Data Section”, found towards the end of the manuscript. The DOI for Zenodo will be provided after the revision process, since Zenodo mirrors the github repository and changes to a published DOI are not possible. Therefore, we will provide the DOI at the end of the revision process, so that any corrections required for the manuscript can be made in our git repository before replicating it onto the final Zenodo for DOI.

* Minor:

- How often do you expect to update this tool? Make clear this point, please

Thank you for asking. GOMAP is actually frequently updated. Author Kokulapalan Wimalanathan (the creator of GOMAP), actively addresses bug fixes and new versions are released as needed (multiple times per year). We added a statement to this effect to the first paragraph of the Methods section.

- Could you clarify all the diff. among your work and Zhu et al. work?

The biggest difference between our work and Zhu et al., 2015 is that their work is on microorganisms, while ours is on plants. In Zhu et al, they were interested in classifying microorganisms on methods that are non-phylogenetic. Since horizontal gene transfer can occur between prokaryotes that are not phylogenetically related, they wanted to classify groups that are metabolically, environmentally, and phenotypically diverse based on function. Unlike us, they did not annotate through one tool that generates a high coverage gene ontology dataset. Instead, they used 3 annotation tools (COG, RAST, Pfam). Also, our distance metric was based on Jaccard distance, while they used HSSP (Homology-derived Secondary Structure of Proteins). The method we chose to use here was phylogenetics, while they used fusion (functional-repertoire similarity-based organism network). While their choice of network is more sophisticated, ours was more of a proof-of-concept to demonstrate that GO-based comparative functional analysis of plants is possible, since it seems that sufficient biological signal is now present in these datasets to reconstruct known phylogenetic relationships. This indicates that the datasets may be useful for comparative functional genomics, not just analyses within a single species. Our preliminary work serves to push us in the direction of finding more sophisticated representations of function-based comparative analyses for plants.

- Did you expect to have any significance (bootstrap) on the trees fig.?

In phylogenetics, jackknife values are not directly related to any sort of biological or statistical significance. Also, as Felsenstein stated, a big obstacle for making statistical interpretation is present when jackknifing or bootstrapping across species and when interpreting the effect of removing one species at a time from a phylogenetic tree [1]. Therefore, we were careful not to state any conclusions based on the values, but rather to simply report the values to show comparative support from the dataset for each node. Some nodes are well-supported by the dataset, while other nodes are more poorly supported. Put another way, the support values report the comparative confidence of each branch during reconstruction of the topologies. We did not expect to see any specific value on the trees, but we did expect to see an increase of those support values when individual "problematic" species were removed, which we did observe. In so doing, we could identify datasets that were putatively inconsistent with others. As such, jackknifing analyses of both the tree containing all species and the tree with the restored topology show an expected result: the species with datasets consistent with evolutionary relatedness have higher branch support based on bootstrapping analyses.

[1] Felsenstein, J., & Felenstein, J. (2004). *Inferring phylogenies* (Vol. 2, p. 664). Sunderland, MA: Sinauer associates.

- Page 5/6, there are zero space lines in section D., and some ?? reference in fig and reference, please, correct this issue.

Thank you for bringing this to our attention. We checked both our manuscript and the publicly available bioRxiv version, but we do not see these issues in either version. This may be an error that happened during the typesetting stage at GigaScience and should be followed up by GigaScience copy editors in next steps.
