# Supplementary material for: Standardized genome-wide function prediction enables comparative functional genomics: a new application area for Gene Ontologies in plants
Source: Gigascience. 2022 Apr 15;11:giac023. doi: 10.1093/gigascience/giac023 (PMC9012101; doi:10.1093/gigascience/giac023)
Supplement: giac023_Supplemental_Figures_and_Tables [file giac023_supplemental_figures_and_tables.zip › Supplemental/TableS1.pdf]

Supplementary Table SI: Additional assembly statistics from GenomeQC.

	Longest Scaffold (bp)	Shortest Scaffold (bp)	Scaffolds >1K nt	Scaffolds >10K nt	Scaffolds >100K nt	Scaffolds >1M nt	Scaffolds >10M nt	N50	L50	NG50	LG50	%A	%C	%G	%T
A. hypogaea	1.61E+08	1000	382	299	42	23	20	1.35E+08	9	1.35E+08	10	31.79	18.14	18.13	31.79
B. distachyon	7.55E+07	2.88E+04	11	11	5	5	5	5.89E+07	3	5.89E+07	3	26.76	23.16	23.14	26.76
C. sativa	1.05E+08	6741	221	218	52	15	10	9.19E+07	5	9.19E+07	5	27.94	14.07	14.11	27.95
G. max	5.83E+07	885	281	271	63	20	20	4.99E+07	10	4.78E+07	11	31.77	16.91	16.91	31.77
G. raimondii	7.07E+07	1001	1033	200	20	14	13	6.22E+07	6	6.10E+07	7	32.81	16.32	16.32	32.81
H. vulgare	7.68E+08	2.50E+08	8	8	8	8	8	6.57E+08	4	5.83E+08	6	26.27	21.02	21.01	26.26
M. truncatula A17	5.66E+07	1004	2186	605	51	8	8	4.92E+07	4	4.57E+07	5	31.60	15.66	15.65	31.57
M. truncatula R108	3.23E+07	1007	909	481	72	49	18	1.28E+07	12	1.23E+07	14	33.25	16.41	16.41	33.25
O. sativa	4.33E+07	4236	63	51	17	12	12	3.00E+07	6	2.90E+07	8	28.21	21.77	21.78	28.21
P. vulgaris	6.30E+07	1005	478	290	61	13	11	4.97E+07	5	4.80E+07	6	31.84	17.61	17.64	31.86
S. bicolor	8.09E+07	1005	870	232	69	12	10	6.87E+07	5	6.87E+07	9	26.74	20.90	20.91	26.73
T. aestivum	8.31E+08	4.74E+08	22	22	22	22	22	7.10E+08	10	6.74E+08	12	26.46	22.59	22.59	26.46
V. unguiculata	6.53E+07	2922	686	679	103	13	11	4.17E+07	6	4.13E+07	7	33.31	16.40	16.42	33.34
Z. mays B73	3.07E+08	5568	266	261	48	12	10	2.24E+08	5	1.82E+08	6	26.18	23.09	23.10	26.19
Z. mays Mo17	3.06E+08	1007	2208	1864	129	11	10	2.20E+08	5	1.83E+08	6	26.14	23.03	23.05	26.17
Z. mays PH207	3.03E+08	500	16840	1115	61	11	10	2.15E+08	5	1.76E+08	6	21.34	18.40	18.40	21.36
Z. mays W22	3.11E+08	1.25E+07	11	11	11	11	11	2.23E+08	5	1.83E+08	6	26.11	22.92	22.94	26.13

Download this table (CSV)

N50: The length of the shortest scaffold/contig in the list of L50 sequences.

L50: The number of sequences whose sum of lengths make up 50% or more of the total assembly length.

NG50: The length of the shortest scaffold/contig calculated in the same manner as N50 but based on estimated genome size rather than total assembly length.

LG50: The number of sequences whose sum of lengths make up 50% or more of the estimated genome size.
