# Supplementary material for: Standardized genome-wide function prediction enables comparative functional genomics: a new application area for Gene Ontologies in plants
Source: Gigascience. 2022 Apr 15;11:giac023. doi: 10.1093/gigascience/giac023 (PMC9012101; doi:10.1093/gigascience/giac023)
Supplement: giac023_Supplemental_Figures_and_Tables [file giac023_supplemental_figures_and_tables.zip › Supplemental/TableS2.pdf]

Supplementary Table SII: Number of removed annotations during cleanup.

Genome	Dataset	Obsolete Annotations	Duplicates	Annotations with Modifiers
Arachis hypogaea	GOMAP	3437	13	912
Brachypodium distachyon	GOMAP	2512	49	789
	Gold Standard Gramene 63 (no IEA)	21	204	0
	Gramene63 (IEA only)	166	114	0
	Phytozome12	99	18	0
Cannabis sativa	GOMAP	1714	6	757
Glycine max	GOMAP	3333	10	930
Gossypium raimondii	GOMAP	1781	7	822
Hordeum vulgare	GOMAP	1877	8	815
	Gold Standard Gramene 63 (no IEA)	1	9	0
	Gramene63 (IEA only)	282	147	0
Medicago truncatula A17	GOMAP	2673	10	798
	Gold Standard Gramene 63 (no IEA)	2	23	0
	Gramene63 (IEA only)	309	243	0
	Phytozome12	132	17	0
Medicago truncatula R108	GOMAP	4168	7	803
Oryza sativa	GOMAP	1642	7	869
	Gold Standard Gramene 63 (no IEA)	37	833	0
	Gramene63 (IEA only)	238	64	0
	Phytozome12	119	19	0
Phaseolus vulgaris	GOMAP	1190	6	783
Pinus lambertiana	GOMAP	1839	4	587
Sorghum bicolor	GOMAP	2384	66	783
	Gold Standard Gramene 63 (no IEA)	178	219	0
	Gramene63 (IEA only)	278	198	0
	Phytozome12	131	12	0
Triticum aestivum	GOMAP	9624	17	1132
	Gold Standard Gramene 63 (no IEA)	1	5	0
	Gramene63 (IEA only)	584	319	0
Vigna unguiculata	GOMAP	1269	6	811
	Phytozome12	122	27	0
Zea mays B73.v4	GOMAP	2077	89	848
	Gold Standard Gramene 63 (no IEA)	50	633	0
	Gramene63 (IEA only)	306	140	0
Zea mays Mo17	GOMAP	2346	83	823
	Gold Standard Gramene 63 (no IEA)	36	1489	0
Zea mays PH207	GOMAP	2676	82	830
	Gold Standard Gramene 63 (no IEA)	37	2702	0
Zea mays W22	GOMAP	2681	88	840
	Gold Standard Gramene 63 (no IEA)	30	499	0

Download this table (CSV)
