# Supplementary figures and images for: Standardized genome-wide function prediction enables comparative functional genomics: a new application area for Gene Ontologies in plants
Source: Gigascience. 2022 Apr 15;11:giac023. doi: 10.1093/gigascience/giac023 (PMC9012101; doi:10.1093/gigascience/giac023)

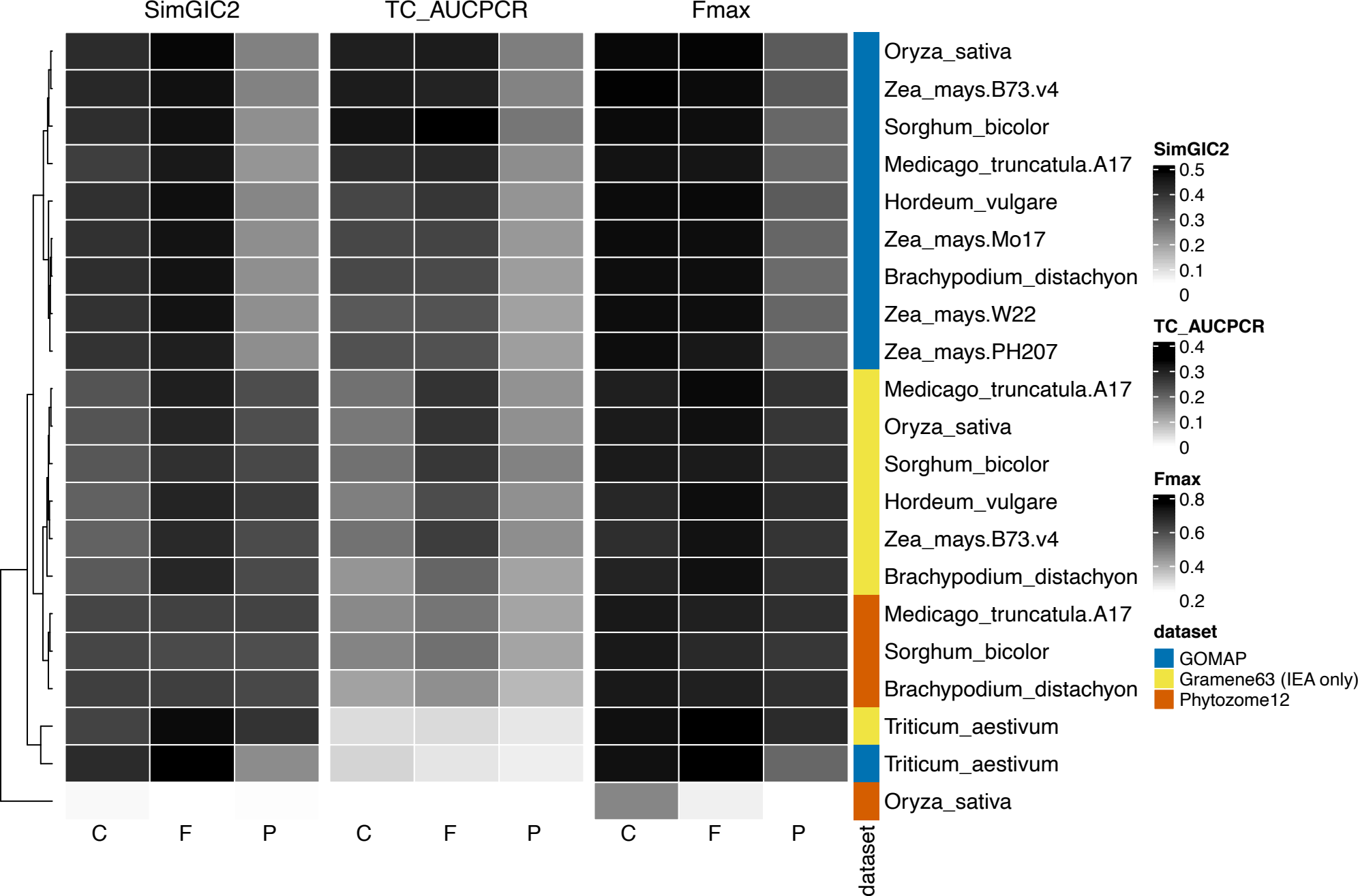

Supplement: giac023_Supplemental_Figures_and_Tables [file giac023_supplemental_figures_and_tables.zip › Supplemental/FigureS2.pdf]

# Number of Annotations

aspect

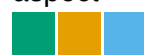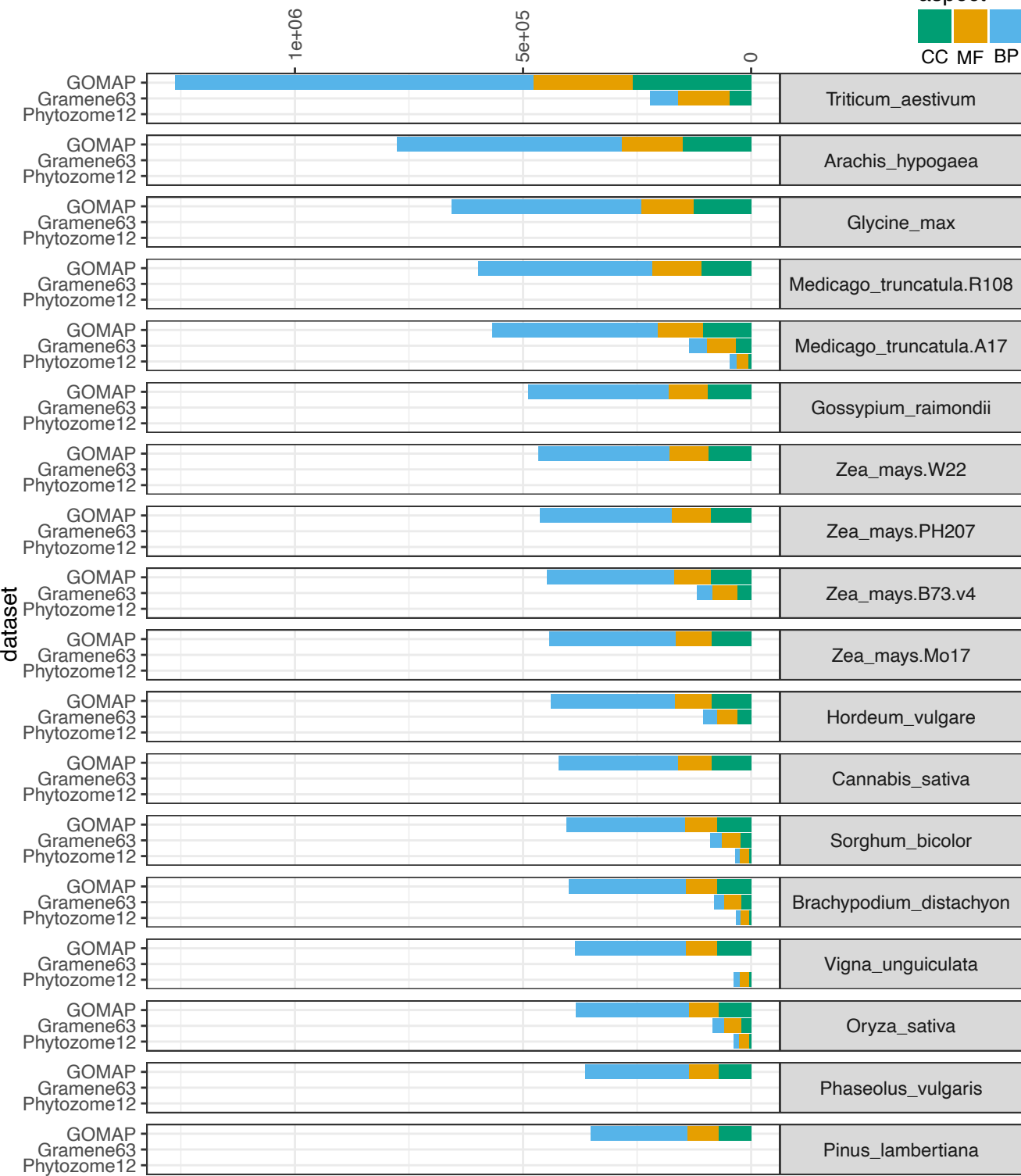

Supplement: giac023_Supplemental_Figures_and_Tables [file giac023_supplemental_figures_and_tables.zip › Supplemental/FigureS1.pdf]
