# Supplementary material for: Standardized genome-wide function prediction enables comparative functional genomics: a new application area for Gene Ontologies in plants
Source: Gigascience. 2022 Apr 15;11:giac023. doi: 10.1093/gigascience/giac023 (PMC9012101; doi:10.1093/gigascience/giac023)
Supplement: giac023_Response_to_Reviewer_Comments_Revision_1 [file giac023_response_to_reviewer_comments_revision_1.pdf]

Author's Response To Reviewer Comments

Close

Please see cover file entitled, Response to Reviewers at end of file group.

Close
