# Supplementary material for: Standardized genome-wide function prediction enables comparative functional genomics: a new application area for Gene Ontologies in plants
Source: Gigascience. 2022 Apr 15;11:giac023. doi: 10.1093/gigascience/giac023 (PMC9012101; doi:10.1093/gigascience/giac023)
Supplement: giac023_Reviewer_1_Report_Original_Submission — Leonore Reiser -- 10/11/2021 Reviewed [file giac023_reviewer_1_report_original_submission.pdf]

Reviewer Report

Title: Standardized genome-wide function prediction enables comparative functional genomics: a new application area for Gene Ontologies in plants

Version: Original Submission **Date:** 10/11/2021

Reviewer name: Leonore Reiser

Reviewer Comments to Author:

The authors present a detailed assessment and creative analysis of computationally predicted functional annotations for 18 plant genomes. First they applied their GOMAP pipeline to annotate the genomes, and compared those outputs against a 'Gold Standard' of Gramene annotations (minus those inferred from Electronic Annotation) and electronically inferred annotations from Gramene or Phytozome. They then used the GOMAP annotation set in an interesting way to perform a sort of phylogenetic reconstruction.

First, I applaud the authors for presenting a manuscript that is a paragon of data FAIRness. The data is findable, accessible, well annotated with metadata and certainly looks reusable (what a pleasure to have the option to download as a CSV.) Bravo! Brava!

The idea of recapitulation of phylogenetic relationships based on GO annotations is an interesting one and while authors do a good of addressing some of the caveats and limitations of their analysis I do wonder if there are other things that they may want to consider. For example a lot of plant annotations are based off of Arabidopsis experimental annotations, which means that some aspects of plant biology that are unique to specific clades may not be well represented in the ontology because those processes have not been annotated or the terms may not even exist in the ontologies yet. Also, at least for Arabidopsis, many of the included annotations come from PAINT which is a phylogenetic based annotation method (the IBA annotations) so transferring IBA annotations from Arabidopsis to other plant species might add a certain phylogenetic flavor the GO MAP results.

Specific comments on the text.

1.Please clarify what the sets of terms used were and what is meant by ancestors? The MS states, granular terms were mapped to higher order terms used for comparison- how were those ancestor terms selected? is there a list of these common (S) terms that were used to generate the trees available somewhere? If so, that subset should be made available (or maybe it is but I could not tell.) I think this selection of terms for use in the analysis is really important but could not find any data for this- if the data is available it is not obvious.

2.Annotations with modifiers that were removed- can you clarify what is meant by that , are those 'Not' annotations?

3.One expects a high level of granularity for manually curated gene functions (that is very specific terms) how are annotations harmonized across the different prediction methods used for GO MAP since presumably some of the methods employed provide less specificity in their annotations?

4.For the comparison, was there any manual inspection of presence or absence of terms? Was there any correspondance with anything known biologically? That is for certain term character states, were there

any unexpected or inconsistent with biology?
5.The phylogenetic analysis seems to factor in all 3 GO aspects, have the authors compared results using just a single aspect (process function or component?) Process is notoriously noisy and annotations can be subject to a lot of interpretation. It is also probably the most incomplete data set,
Specific comments on the figures.
1. Panel b. Gramene -IEA is confusing here in the figure and when described elsewhere. I suggest that in the figure ,and the text, using less confusing nomenclature such as Gramene (IEA only) and Gramene (no IEA) for gold standard. To me I read Gramene-IEA as Gramene minus IEA annotations and not Gramene's IEA annotations only.
2.Supplementary Figure S1.
I wonder if there is a more effective way to visualize this data. I think there is a lot of interesting information here but it is hard to follow , especially the third graph. Another improvement to readability would be to make the text font darker (not sure why it is light grey.)

Methods

Are the methods appropriate to the aims of the study, are they well described, and are necessary controls included? Choose an item.

Conclusions

Are the conclusions adequately supported by the data shown? Choose an item.

Reporting Standards

Does the manuscript adhere to the journal's guidelines on minimum standards of reporting? Choose an item.

Choose an item.

Statistics

Are you able to assess all statistics in the manuscript, including the appropriateness of statistical tests used? Choose an item.

Quality of Written English

Please indicate the quality of language in the manuscript: Choose an item.

Declaration of Competing Interests

Please complete a declaration of competing interests, considering the following questions:

- Have you in the past five years received reimbursements, fees, funding, or salary from an organisation that may in any way gain or lose financially from the publication of this manuscript, either now or in the future?
- Do you hold any stocks or shares in an organisation that may in any way gain or lose financially from the publication of this manuscript, either now or in the future?
- Do you hold or are you currently applying for any patents relating to the content of the manuscript?
- Have you received reimbursements, fees, funding, or salary from an organization that holds or has applied for patents relating to the content of the manuscript?
- Do you have any other financial competing interests?
- Do you have any non-financial competing interests in relation to this paper?

If you can answer no to all of the above, write 'I declare that I have no competing interests' below. If your reply is yes to any, please give details below.

I declare that I have no competing interests

I agree to the open peer review policy of the journal. I understand that my name will be included on my report to the authors and, if the manuscript is accepted for publication, my named report including any attachments I upload will be posted on the website along with the authors' responses. I agree for my report to be made available under an Open Access Creative Commons CC-BY license (http://creativecommons.org/licenses/by/4.0/). I understand that any comments which I do not wish to be included in my named report can be included as confidential comments to the editors, which will not be published.

Choose an item.

To further support our reviewers, we have joined with Publons, where you can gain additional credit to further highlight your hard work (see: https://publons.com/journal/530/gigascience). On publication of this paper, your review will be automatically added to Publons, you can then choose whether or not to claim your Publons credit. I understand this statement.

Yes Choose an item.
