# Supplementary material for: Standardized genome-wide function prediction enables comparative functional genomics: a new application area for Gene Ontologies in plants
Source: Gigascience. 2022 Apr 15;11:giac023. doi: 10.1093/gigascience/giac023 (PMC9012101; doi:10.1093/gigascience/giac023)
Supplement: giac023_Reviewer_2_Report_Original_Submission — Alexandre R. Paschoal -- 10/12/2021 Reviewed [file giac023_reviewer_2_report_original_submission.pdf]

Reviewer Report

Title: Standardized genome-wide function prediction enables comparative functional genomics: a new application area for Gene Ontologies in plants

Version: Original Submission **Date:** 10/12/2021

Reviewer name: Alexandre R. Paschoal

Reviewer Comments to Author:

The authors present a "Standardized genome-wide function prediction enables comparative functional genomics: a new application area for Gene Ontologies in plants". It seems to be an application of the GOMAP pipeline in 14 species which sounds to be interesting. However, it lacks polish, thats why I list some suggestions to help the authors improve it.
* Major:
1-) It is not clear the state-of-art tools in this topic (this is not detailed in introduction, which is a serious gap in this work), including similar or same tools/methods for the same purpose. Keep this in mind, please, compare against tools from literature in the same issue please.
I am not an expert in the GO topic, but as far as I know, there is Blast2GO, and I found others:
https://www.mdpi.com/1999-5903/13/7/172
https://academic.oup.com/nar/article/49/D1/D394/6027812?login=true
https://onlinelibrary.wiley.com/doi/full/10.1111/1755-0998.13285
https://david.ncifcrf.gov/
blast2go
etc
1.2.) Please, compare against these tools, or those that make sense, if not, why not? Clarify and make clear this, please.
PS: Re-write the introduction to address these points.
2-) The idea is to do a large-scale analysis of several plant species (in case 14) using the GOMAP pipeline. Is it?
2.1-) Please make clear how many and each of the contributions in the abstract and introduction.
2.2-) We now have more than 70 plants species in Ensembl Plants, for example. Why do not use all of them as much as possible for a real large-scale analysis?
3-) If I understood, the GitHub (https://github.com/Dill-PICL/GOMAP-Paper-2019.1) and the https://dill-picl.org/projects/gomap/ gomap-datasets/ contains all the data results from this report, is it?
3.1.) Both (and mainly GitHub) are far from being user-friendly. It seems the author put the information and that it. Be more clear, what, how, why this information is there, and how to use it. Also, is it clear all commands used (maybe provide a manual on what you have done in a tech aspect)?
3.2.) Is there any visualization table, I mean an easy output produced by this analysis? If I want to use this data for my new genome etc, real case, how to use it? how to compare? where? sequence? GO terms, etc? Clarify this, please.
PS: Imagine that is a biologist that wants to use your approach.

4-) For me is not clear why do not also put Ensembl Plants in this report analysis, and only Phytozome and Gramene. Please, include and compare all these databases.
5-) Authors mention that they will make available the final results in Zenodo after this revision. Please, make all data, FASTA, trees etc available.
* Minor:
- How often do you expect to update this tool? Make clear this point, please
- Could you clarify all the diff. among your work and Zhu et al. work?
- Did you expect to have any significance (bootstrap) on the trees fig.?
- Page 5/6, there are zero space lines in section D., and some ?? reference in fig and reference, please, correct this issue.

Methods

Are the methods appropriate to the aims of the study, are they well described, and are necessary controls included? Choose an item.

Conclusions

Are the conclusions adequately supported by the data shown? Choose an item.

Reporting Standards

Does the manuscript adhere to the journal's guidelines on minimum standards of reporting? Choose an item.

Choose an item.

Statistics

Are you able to assess all statistics in the manuscript, including the appropriateness of statistical tests used? Choose an item.

Quality of Written English

Please indicate the quality of language in the manuscript: Choose an item.

Declaration of Competing Interests

Please complete a declaration of competing interests, considering the following questions:

- Have you in the past five years received reimbursements, fees, funding, or salary from an organisation that may in any way gain or lose financially from the publication of this manuscript, either now or in the future?
- Do you hold any stocks or shares in an organisation that may in any way gain or lose financially from the publication of this manuscript, either now or in the future?

- Do you hold or are you currently applying for any patents relating to the content of the manuscript?
- Have you received reimbursements, fees, funding, or salary from an organization that holds or has applied for patents relating to the content of the manuscript?
- Do you have any other financial competing interests?
- Do you have any non-financial competing interests in relation to this paper?

If you can answer no to all of the above, write 'I declare that I have no competing interests' below. If your reply is yes to any, please give details below.

I declare that I have no competing interests

I agree to the open peer review policy of the journal. I understand that my name will be included on my report to the authors and, if the manuscript is accepted for publication, my named report including any attachments I upload will be posted on the website along with the authors' responses. I agree for my report to be made available under an Open Access Creative Commons CC-BY license (http://creativecommons.org/licenses/by/4.0/). I understand that any comments which I do not wish to be included in my named report can be included as confidential comments to the editors, which will not be published.

Choose an item.

To further support our reviewers, we have joined with Publons, where you can gain additional credit to further highlight your hard work (see: https://publons.com/journal/530/gigascience). On publication of this paper, your review will be automatically added to Publons, you can then choose whether or not to claim your Publons credit. I understand this statement.

Yes Choose an item.
